# Somatic, Psychiatric and Neurodevelopmental Comorbidities in People with Autism Spectrum Disorder (ASD): A Narrative Review

**DOI:** 10.3390/healthcare14162492

**Published:** 2026-08-11

**Authors:** Emma Dando, Juergen Hahn, David A. Geier, Athena Whiteley, Paul Whiteley

**Affiliations:** 1 Department of Biomedical Engineering, Rensselaer Polytechnic Institute, Troy, NY 12180, USA; dandoe@rpi.edu (E.D.); hahnj@rpi.edu (J.H.); 2 Institute of Chronic Illnesses, Inc., 14 Redgate Ct, Silver Spring, MD 20905, USA; 3 Independent Researcher, Sunderland SR5 3RL, UK; 4 ESPA Research (Education & Services for People with Autism), North House, Ferryboat Lane, Castletown, Sunderland SR5 3RL, UK

**Keywords:** autism spectrum disorder, comorbidity, multimorbidity, behavioural, psychiatric, somatic, risk, sex, age, screening

## Abstract

**Highlights:**

**What are the main findings?**

- A diagnosis of autism spectrum disorder (ASD) carries elevated risk(s) for multiple comorbidities across behavioural, psychiatric, and somatic domains.
- Data on the frequency of various comorbidities are presented alongside their profiles according to sex and age.

**What is the implication of the main findings?**

- Such data forms the starting point for investigations into screening programmes for the early detection and intervention for various comorbidities which can impact quality of life.

**Abstract:**

**Background/Objectives**: Autism spectrum disorder (ASD) is currently singularly described as a behaviourally defined neurodevelopmental diagnosis with symptoms affecting social communication and social understanding alongside the presence of restricted patterns of behaviour. Wide, and often very personal, variations in symptom intensities and differing developmental trajectories, reflective of large heterogeneity, are an important feature of the label. ASD carries enhanced risks for multiple over-represented, sometimes overlapping, comorbidities spanning behavioural, psychiatric and somatic domains. Said comorbidities often have numerous and far-reaching effects on quality of life by way of their impacts and, in extreme cases, their potential effects on life expectancy. It is of paramount importance that data on the risks of such comorbidities are available and, where possible, screening, preventive and/or treatment options implemented. **Methods**: In this narrative review, the authors searched databases (PubMed/Medline, ScienceDirect, Google Scholar) for papers published between 1 January 2015 and 16 February 2026 that were pertinent to comorbidity and autism. **Results**: We present data on the frequency of various somatic (gastrointestinal, immunological, metabolic, neurological, motor-sensory disorders), psychiatric (anxiety, mood, schizophrenia spectrum, personality, substance abuse, trauma, feeding and eating, sleeping, self-harm, neurocognitive disorders) and neurodevelopmental comorbidities (intellectual, attentional, speech and language, motor disorders) that can present alongside a diagnosis of ASD. We provide accompanying data on the magnitude of potential risk and, where available, information on comorbidity risks according to sex and age. **Conclusions**: This review paper deals with an extremely broad field. Limitations of the narrative review strategy are discussed alongside how the variable risk of comorbidity mimics the heterogeneity present in autism, thus inviting further investigations.

## 1. Introduction

**Figure 1:**
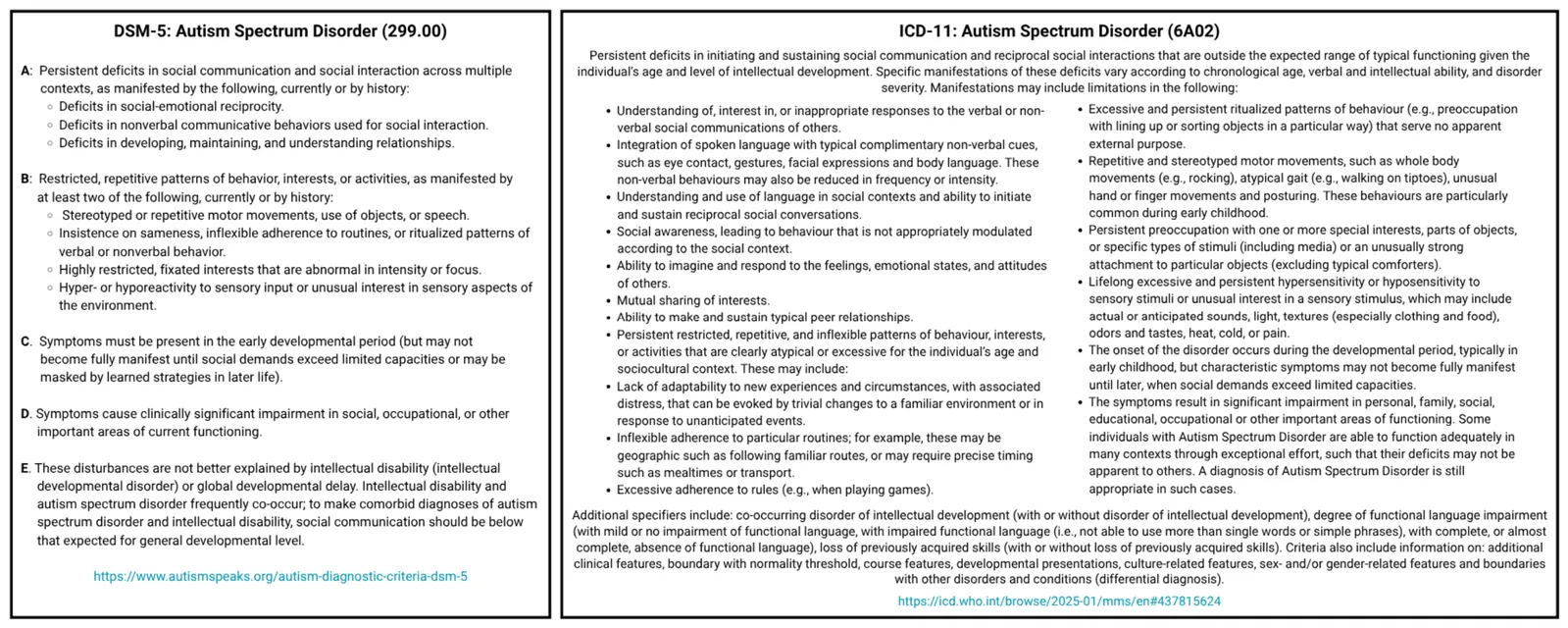
Diagnostic criteria for autism based on DSM-5 and ICD-11.

Autism spectrum disorder (ASD) or autism is currently listed as a singular neurodevelopmental disorder in contemporary DSM (version 5) and ICD (version 11) diagnostic schemes [1,2] (see Figure 1). Core features include (i) clinically relevant issues with social communication and social understanding—potentially affecting both verbal and non-verbal communication—alongside (ii) the presence of repetitive and/or restricted patterns of behaviour. The presence of such characteristics, outside the boundaries of typical presentations in frequency and severity, cumulatively merits a clinical diagnosis alongside judgement of their significant and sustained disabling effects on daily functioning. The onset of behaviours associated with autism is traditionally present in early infancy. There is, however, increasing recognition that clinically relevant symptoms can, for some, manifest in later childhood. Specifically, either as part of maturation, when social demands increase, or following regression, where previously acquired developmental milestones were met and skills gained but plateaued and/or were subsequently lost.

As part of, or alongside, the core dyad of features that define autism, various other characteristics and traits can be present. Much has been traditionally made about how the social communication element of the diagnosis can influence the development of skills such as some forms of empathy, sharing, turn-taking and reciprocity behaviours. Whilst evident and potentially disabling for some, such features are, however, typically amenable to various forms of learning and intervention. More disabling and, currently, less treatable, are the various sensory issues that typically occur alongside autism, where hypersensitivity and/or hyposensitivity to sensory stimuli can provoke challenges for the individual and those around them. Whether a seeming hyposensitivity to pain or hypersensitivity to lights, textures and tastes, the knock-on effects of such symptoms can have serious impact, particularly in relation to restricted eating patterns and onwards risk of various nutritional deficiencies. Such symptoms can similarly also lead to sensory-seeking patterns of behaviour which can, combined with a lack of danger awareness and the presence of wandering/elopement behaviours, place someone at considerable risk when seeking out particularly sources of water. Various other notable traits can similarly be present but are not necessarily covered by the core defining features of autism. These include emotional dysregulation manifesting as irritability and frustration (particularly in childhood), which can also lead to episodes of reactive and explosive aggression towards self or others under specific circumstances.

Autism is a heterogeneous label which encapsulates wide variations in symptom(s) expressions and intensities. Although modern descriptions of autism have always to some degree ‘compartmentalised’ the label, there is an increasing emphasis on such intra-label distinctions according to either differing support needs (as highlighted in DSM-5) and/or differing expressions of verbal language use and/or the presence of intellectual (learning) disability (as highlighted in ICD-11). More recent moves to define a ‘profound autism’ phenotype [3] have, in part, led to suggestions that a more plural ‘autisms’ description [4] may be better reflective of such diversity in presentation. This, coupled with data suggesting different aetiological routes towards autism and differing longer-term developmental trajectories, implies that autism for some may not be a lifelong condition [5] and/or may be a prodromal state to other subsequent diagnoses [6].

Factors increasing the likelihood of autism are as heterogeneous as symptom presentations. Several well-defined variables are known to culminate in a diagnosis of autism, covering syndromic presentations accompanying known genetic conditions, post-infectious or immune-related insults (e.g., encephalitis) [2] and specific in-utero pharmaceutical exposures (e.g., sodium valproate) [4]. But for many expressions of autism, a distinct aetiology or aetiological pattern has yet to be properly elucidated, particularly where potentially multiple variables are working synergistically to elevate the risk of autism and stretching across different time periods (pre-conception, in-utero, postpartum).

Discussions on the aetiologies of autism are also particularly relevant to the prevalence of autism. Multiple data show a consistent increase in both the number of children presenting with features of autism and those in receipt of a formal diagnoses [7] across decades. The potential reasons for such an increase are complex. They will include, but are not limited to, increasing awareness of autism, modification and expansion of the diagnostic criteria to include less severe or less prototypical presentations, diagnosis being linked to healthcare and educational resource access and sociocultural influences (e.g., reduced stigma around the label alongside Looping effects). The scale of the increase, however, particularly across more severe presentations that are unlikely to have been missed, also implies a partly real increase in childhood autism [8]. The idea of ‘acquired autism’, as per the example provided in the ICD-11 diagnostic criteria for autism where autistic traits become manifest post-encephalitis, represents one example of how aetiology and prevalence may well be linked, albeit still lacking clear mechanistic and operational definitions and criteria.

### 1.1. The (Co-Occurring) Boundaries of Autism

**Figure 2:**
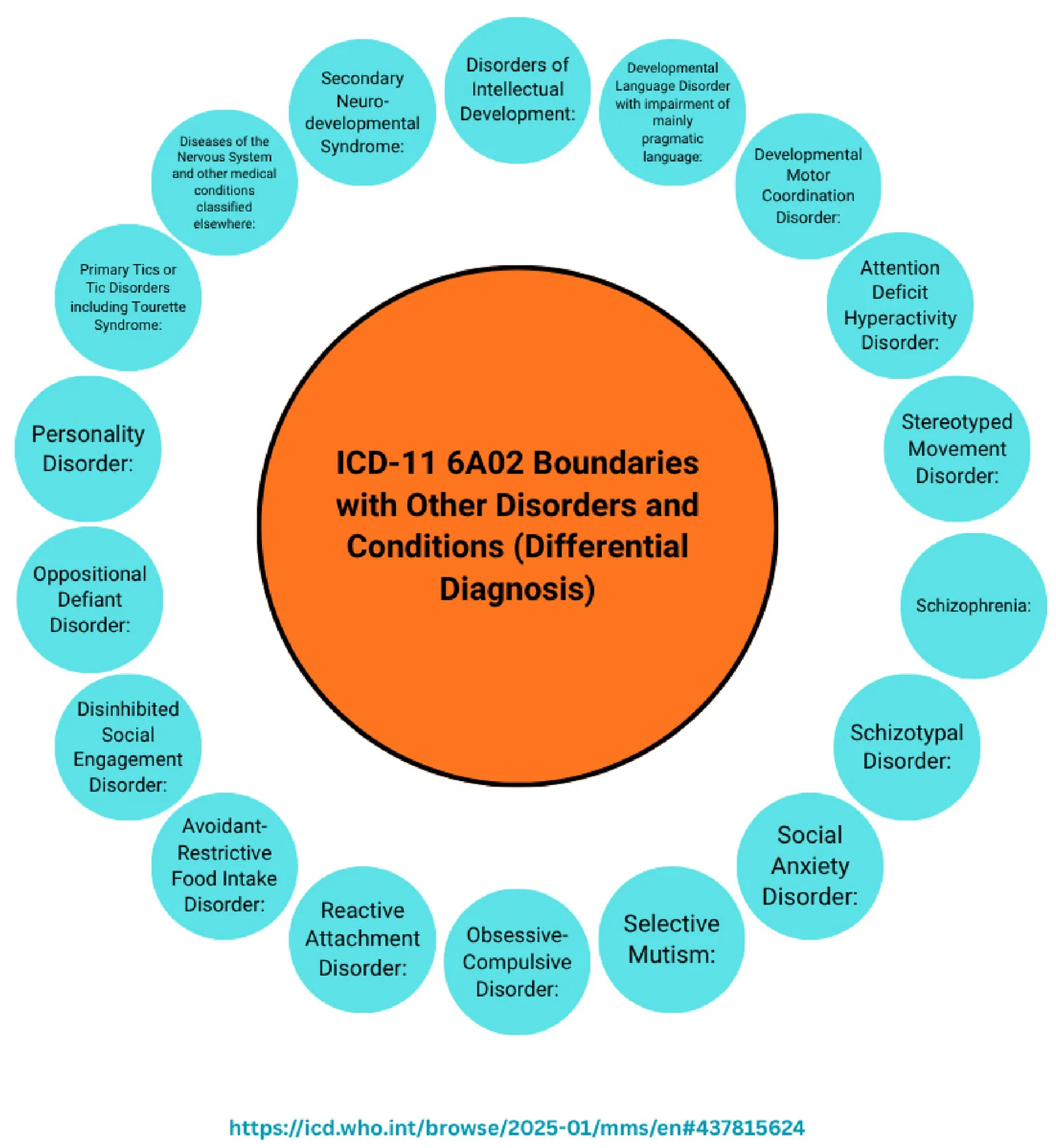
ICD-11 autism boundary conditions.

There is increasing recognition that whilst the significant and life-altering presence of autistic traits is implicit to awarding a diagnosis of autism, such traits are (a) present along a continuum in terms of severity and persistence and (b) are not necessarily the sole domain of an autism diagnosis. Point (a) has its roots in various historic events including acknowledgement of the sub-diagnostic ‘broader autism phenotype’ (BAP) and the inclusion of the separate diagnosis of ‘social (pragmatic) communication disorder’ (SCD) in the DSM-5 [1]. Point (b) is an emerging idea presented in the ICD-11. Whilst not yet as popular as the DSM-5 diagnostic criteria (particularly in the United States where it is not yet used), the ICD-11 definition of autism extends this focus on autistic symptoms not necessarily implying a diagnosis of autism, with its description of various boundary conditions (see Figure 2).

Multiple boundary conditions are described, including diagnoses such as attention-deficit hyperactivity disorder (ADHD), social anxiety disorder, Avoidant-Restrictive Food Intake Disorder (ARFID) and schizophrenia. The ICD-11 autism boundary conditions also now—and seemingly for the first time for any version of such diagnostic manuals—include somatic diseases that may also present with autistic traits, suggesting for example, that such traits may become ‘manifest’ post-encephalitis or can be similarly acquired following acquired epileptic aphasia, Landau–Kleffner syndrome, autoimmune encephalitis, or Rett Syndrome [2].

Collectively, such descriptions have multiple potential consequences for our understanding of autism. They imply that diagnostic assessments for autism, or indeed any one of the various boundary conditions highlighted, need to encompass a greater level of detail such that autism and those boundary conditions are at least screened for whether as stand-alone conditions or as some diagnostic combination as potential comorbidity. They also imply that such transdiagnostic autistic features may well be important for intervention outcomes for conditions outside of autism. Given that some of those comorbid conditions also appear later than autism, i.e., outside of early infancy or childhood, they similarly imply the possibility of movement from autism to one or more of those boundary conditions in keeping with the ‘progression’ evidence available so far [6].

### 1.2. Autism Is Not a Static Entity

Within the vast heterogeneities of autism presentations, there is typically ‘movement’ in symptoms presentations across both the short- and long-term. Much of the short-term heterogeneity in symptoms is reactive; relating to environmental factors such as changes which typically accompany daily living and how such changes are perceived. A child with autism may well experience significant distress when important routines are altered either with purpose or by accident, which will typically be reflected in overt behaviours. Similarly, accommodations to help that child manage such changes, either in planning or distraction or other learned response, may likewise avert some more negative behaviours.

Across the longer-term, reactive changes may also be accompanied by more fundamental changes apparent across multiple presentations of autism. For a few, this potentially implies a ‘loss of autism diagnosis’ [5] (or at least not reaching diagnostic thresholds for autism having previously done so) and various accompanying issues too [9]. Whether this loss of diagnosis is universally accurate in terms of genuine neurodevelopmental ‘normalisation’ or merely reflective of reduced symptom severity and/or the increasing use of social skills linked to impression management skills requires further study. Further investigations are required to ascertain the precise prevalence of any such diagnostic loss and the longer-term stability of changes to diagnostic status. At the other extreme, it means a continuing journey into more disabling presentations of autism, where increasing behaviours and traits linked to maturing physiology (e.g., puberty) and more, translate into increasing dependency on others and for some, a lifelong dependency on others for everyday wants and needs, whether with parents or other caregivers. In between these trajectories, a mass of varied presentations and different behavioural severities across different behavioural indices are present. Whilst still reactive to day-to-day circumstances, more individual longitudinal behavioural trajectories wax and wane, interwoven with the effects of similarly fluctuating comorbidity trajectories.

### 1.3. Autism Is Not Typically a Stand-Alone Condition

Alongside the large heterogeneity in symptoms presentation in autism, a further layer of complexity is present with the addition of various comorbidities that are over-represented in autism [10,11]. The idea of ‘autism plus’ [12] is by no means a new one but has gathered significant momentum as the scope, nature and prevalence of such comorbidities has gained prominence. It implies that although autism may be the primary diagnosis, other transdiagnostic signs and symptoms will inevitably be present and present at various times, under different circumstances across different people and, sometimes, in different intensities.

**Figure 3:**
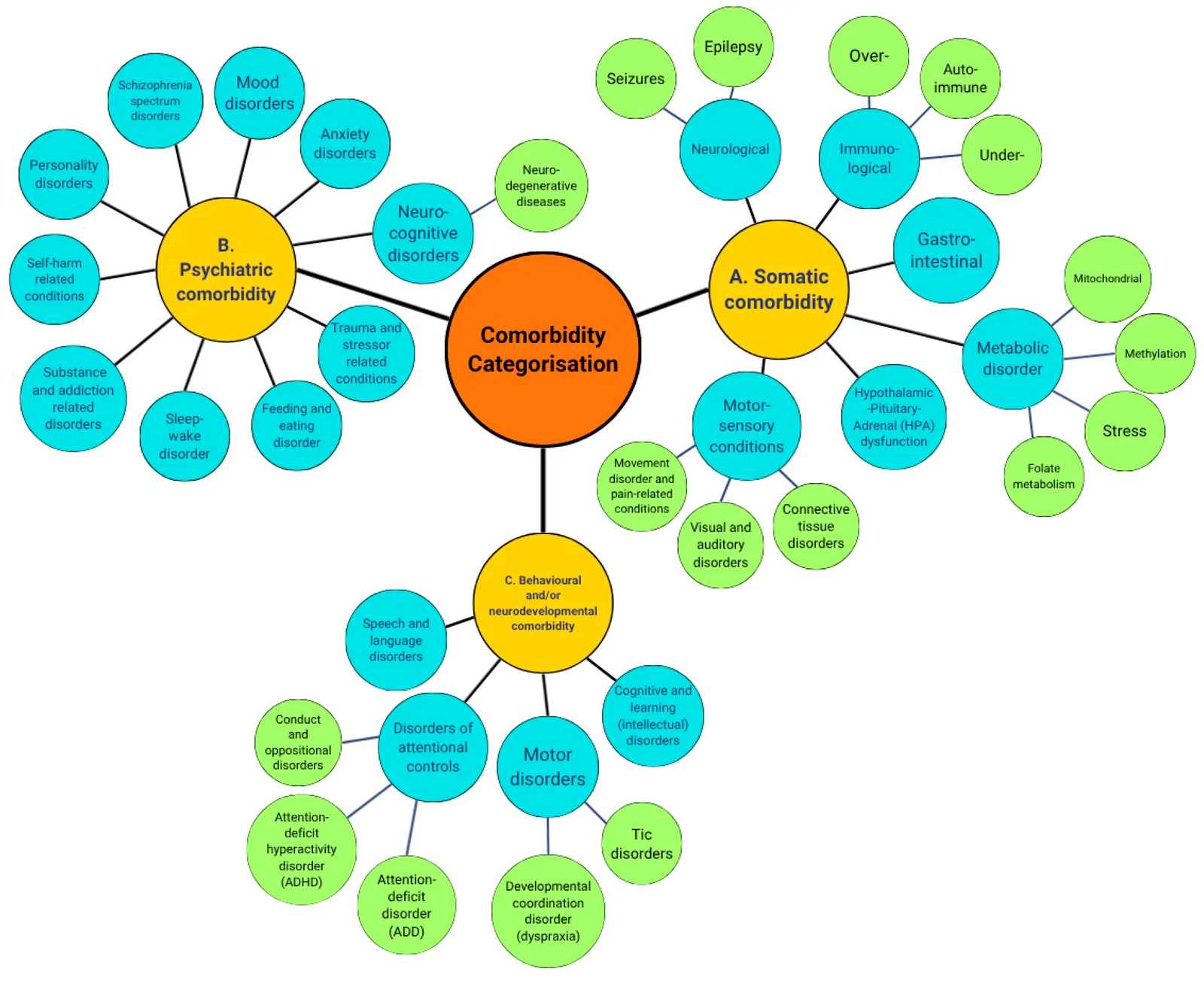
Categories for comorbidities including sub-categorisations.

The numbers and types of comorbidities that can follow a diagnosis of autism are considerable [13] but typically fall into one of three categories: (i) somatic, denoting conditions that act on biology and/or biochemistry and are typically diagnosed on the basis of symptom presentation and/or objective medical and/or biological test(s), (ii) psychiatric, denoting behavioural symptoms or clusters of symptoms that act on or are part of a person’s psychology, and (iii) neurodevelopmental, denoting behaviourally defined symptoms or clusters of symptoms that can intersect with psychiatric features but are typically first present and indeed, most severe, in infancy, childhood and into early adulthood (see Figure 3).

Such demarcations, whilst important, do not rule out the possibility of cross-definition presentations, where most if not all of the conditions headed under ‘psychiatric’ and ‘neurodevelopment’ will have accompanying biology and biochemistry that are, as yet, not completely known to science.

Comorbidities present alongside autism will have varying effects on quality of life, ostensibly negative effects. By their very nature, such comorbidity in the form of symptoms, disease or disorder, will either increase the level of disability present and/or reduce quality of life, whether by some direct effect or, typically, because some combination of treatment or management strategy will also be required as a result. In some cases, comorbidities can also be potentially life-limiting either as a function of disease or condition progression or where management of said conditions is not adequately or substantially undertaken.

Herein we provide a narrative review of the various comorbidities, spanning somatic, psychiatric, and neurodevelopmental domains, that have been indicated as being frequent and/or overrepresented alongside a diagnosis of autism, whether singularly or clustering. We provide accompanying data on the magnitude of the potential risk(s) and, where available, information on comorbidity risks according to sex, chronological age, and as a function of trajectory information.

## 2. Methods

We chose a narrative review methodology incorporating three aspects: analysis of current understanding, how we arrived at the status quo and implications for future directions [14]. This follows the use of a similar methodology by study authors in previous pertinent studies [15,16]. Inclusion criteria comprised (i) research studies including cohort or cross-sectional studies; (ii) research that provided prevalence rates on comorbid or co-occurring symptoms, conditions or diagnoses appearing alongside autism or autism spectrum disorder (ASD); (iii) original peer-reviewed studies published in English. Exclusion criteria comprised (i) case studies or studies including fewer than fifty participants and (ii) papers published before 1 January 2015. The search criteria consisted of authors searching databases (PubMed/Medline, ScienceDirect, Google Scholar) from 1 January 2015 to 16 February 2026, using the keywords autism OR autism spectrum disorder, either individually or in combination, AND prevalence, co-occurring/co-occurrence, comorbid/comorbidity, epidemiology relating to all discussed category names (medical comorbidity, psychiatric comorbidity, neurodevelopmental comorbidity). Keywords were successively added to the search parameters as the review progressed. This was completed on 16 February 2026. The search strategy was enhanced through hand searching reference sections of included articles to identify any additional eligible papers. Following completion of the searches, duplicates were removed, and authors screened the title and abstracts of all results obtained for eligibility. Data from the included papers were extracted in line with the categorical placements highlighted in Figure 3. The specific studies pertinent to each categorisation are shown in Supplements S1–S3, showing (i) Source (authors and reference), (ii) Sample (including ASD and/or non-ASD status, sample sizes, age ranges, sex, country of study, data type), (iii) Prevalence and (iv) Other Notes. A narrative method was chosen over a more structured review due to the breadth and comparative lack of depth of alternatives. There are few studies examining the prevalence of individual conditions, particularly for rarer conditions or conditions less discussed in combination with ASD. Additionally, naming and keyword conventions vary broadly between papers. The combination of such issues created the concern that a more structured review may miss substantial portions of the relevant literature.

## 3. Results

Following our categorisation of ASD comorbidity shown in Figure 3, several trends were observed.

**A. Somatic conditions**

Details of the various studies included under the heading ‘somatic comorbidity’ are shown in Supplement S1. Given the volume of data generated from searches, the main findings are summarised with key references.

*i. Gastrointestinal*

Gastrointestinal (GI) symptoms and disorders were more common in people with ASD compared with non-ASD control participants. Among the numerous studies included (see Supplement S1), the prevalence ranged from 0 to 67% of cases presenting with GI issues, including both symptoms and disorders [17]. Most studies reported a percentage frequency in the range 10–40% of cases [18,19]. Females with ASD were more likely to be diagnosed with a GI condition or disorder than males with ASD [20]. The best estimate for the presence of any GI symptoms in ASD, based on a meta-analysis study [21], was 48%. Analysis of specific GI symptoms again showed wide variations across the different studies. The frequency of constipation ranged between 1 and 65% across studies (see Supplement S1 Section S1.1.2). The best estimate for the presence of constipation in ASD, based on a meta-analysis study, was 26% [21]. The frequency of diarrhoea ranged between ~2–64% across studies (see Supplement S1 Section S1.1.3). The best estimate for the presence of diarrhoea in ASD, based on a meta-analysis study [21], was 20%. The frequency of reflux ranged between 1 and 19% across studies (see Supplement S1 Section S1.1.4). The best estimate for the presence of reflux in ASD, based on a meta-analysis study [21], was 8%. Frequency data for various other GI symptoms or related issues were also extracted (see Supplement S1 Section S1.1.5), suggesting that food selectivity was common (59%), alongside experiences of nausea (25%), bloating and distension (22%) and abdominal pain or discomfort (21%) [21]. Rates of faecal and urinary incontinence across age ranges were also elevated compared to non-ASD controls [22].

Extracted data on more functional pathological GI conditions also revealed some important trends. The frequency of irritable bowel syndrome (IBS) was significantly more common in ASD in one survey [22] (25% ASD vs. 11% non-ASD). Various diseases of the upper and lower GI tract were also present in ASD, including diseases of the oesophagus, stomach and duodenum, including inflammatory bowel diseases, diseases of the liver, and peptic ulcer disease, albeit with a mixed profile of the presence or absence of additional risk (see Supplement S1 Section S1.1.7).

*ii. Immunological*

Various specific immunological-related issues and diseases were more common in individuals with ASD compared with non-ASD control participants (see Supplement S1 Section S1.2), although the overall trend in relation to the over-representation of immune-related conditions was mixed. Grouping immunological issues into autoimmune and allergy (atopy), several results are pertinent. Several studies highlighted an increased risk of autoimmune disease in ASD [18,23]. The reported prevalence of autoimmune conditions in ASD varied widely from 1 to 24% across different studies [24,25]. Various types of autoimmune disease were highlighted, including type-1 diabetes (T1D), psoriasis, alopecia, and vitiligo. Coeliac disease (CD), an autoimmune condition characterised by GI symptoms related to the ingestion of the dietary protein gluten, was shown to be present more frequently in ASD compared with data from non-ASD participants. One study [19] based on meta-analysis estimated a prevalence of CD between 1 and 9% in ASD. This compares with general population estimates typically around 1%. Allergic or atopic disease similarly revealed an over-representation in cases of ASD vs. non-ASD participants (see Supplement S1 Section S1.2.2). The overall estimated frequency of any allergy, based on data from multiple studies [23,26], in ASD was between 15 and 62% of cases. Food allergy (to include both IgE and non-IgE-mediated food allergies and/or intolerances) was a prominent issue that was reported as being more frequent in ASD across studies included for inspection (see Supplement S1 Section S1.2.2). The prevalence of food allergies in ASD ranged between ~1 and 29% [19,24] compared with a range between 0.5 and 10.3% in non-ASD cohorts included for analysis [23,24]. Various other types of allergies were also more frequently present in ASD, including rhinitis, various skin allergies, and asthma (see Supplement S1 Section S1.2.2 for further details).

*iii. Metabolic*

Metabolic disorders, including endocrine, nutritional, and metabolic disorders, were reported to be more frequently present in ASD compared to non-ASD across several studies based on large database analyses (see Supplement S1 Section S1.3). This elevated risk was notable across the lifespan [27] and, in some studies, showed differing risk profiles in ASD between the sexes [28]. Intersecting and adding to the data already discussed on diabetes in the context of potential autoimmune issues accompanying autism, multiple studies reported a higher prevalence of diabetes in ASD across different chronological ages [29]. Type-2 diabetes (T2D) is a particularly important metabolic disease, the risk of which was shown to be elevated across multiple studies comparing ASD and non-ASD population rates. The disparity between ASD and non-ASD frequency of T2D was dependent on age, sex and ethnicity but typically showed a frequency of between 7 and 36% for ASD [30,31] compared with 4–27% for non-ASD [31,32] depending on data source and study inclusion parameters, including any differentiation or not between T2D and T1D. A potential signal suggesting that females with ASD were more likely than males with ASD to develop T2D [33] adds to the potential importance of this data. This trend also seemed to be apparent in those studies looking at prediabetes too.

*iv. Neurological*

Epilepsy and/or seizure-related disorders take greatest prominence when it comes to this specific sub-category. Unsurprisingly, nearly all comparative studies analysed showed an elevated risk of epilepsy and seizures in ASD compared to non-ASD participants (see Supplement S1 Section S1.4.1). The prevalence of epilepsy/seizures ranged between 0 and 77% in the data included for analysis [17], where chronological age and the presence of intellectual disabilities also seemingly influenced risk. A pooled prevalence of 16% [19] was reported. A general prevalence estimate of epilepsy/seizures in children ranges between 4 and 13% [34,35]. Various data also suggested that epilepsy may be more common in females with ASD than males with ASD [28], albeit with cautions around generalisation.

*v. Motor-sensory conditions*

Connective tissue disorders, incorporating joint hypermobility and movement and/or pain-related disorders, were identified in several studies as being over-represented in ASD (see Supplement S1 Section S1.5.1). Various reviews incorporating individual surveys and patient register data cumulatively suggested that around 1 in 5 people with ASD showed joint hypermobility (22%) [36]. Specific diseases that include joint hypermobility, such as Ehlers–Danlos syndrome, were reported in 6.3% of females with ASD compared with 0.8% in non-ASD females based on survey data [22]. Similar disparities in the frequency of pain-related disorders such as Fibromyalgia were also noted [22]. Many of these studies were based on adult populations, so caution is required when generalising to younger age ranges. Auditory disorders such as hearing loss and deafness were reported more frequently in ASD compared to non-ASD cohorts (see Supplement S1 Section S1.5.3). Again, considering the interfering variable of chronological age, significant differences in the rates of auditory disorders in ASD vs. non-ASD, favouring ASD, were found. The differing types and definitions of auditory disorders (e.g., hearing loss, hearing impairment) make estimating difficult, but figures between 2 and 94% were frequently cited in ASD [18,30]. Alongside, several other auditory issues were noted more frequently in the ASD group(s), including hyperacusis estimated at 69% in one study [37]. Visual disorders (see Supplement S1 Section S1.5.3), including blindness and specific physiological eye conditions, showed a similar pattern to auditory issues as far as multiple issues being overrepresented in ASD [22,30], considering variables such as ageing effects. A review paper [17] reported vision impairment in children with autism to be present in between 0–15%. Older adults (over 65) with ASD were typically more likely to present with low vision or blindness than non-ASD cohorts. The research in this area typically did not consider eye and vision problems derived from self-injurious behaviour or following nutritional deficiencies, for example, which can also affect functioning.

*vi. Hypothalamic–Pituitary–Adrenal (HPA) dysfunction.*

Sex hormone disturbances were noted in cases of ASD (see Supplement S1 Section S1.6). Endocrine issues were typically over-represented in cases of autism [18,22] across various age ranges. Specific gynaecological conditions including polycystic ovary syndrome (PCOS) were more frequently described or diagnosed in women with autism [22]. Puberty disorders such as precocious puberty were also more frequently reported [22,38].

**B. Psychiatric conditions**

Details of the numerous studies included under the heading ‘psychiatric comorbidity’ are shown in Supplement S2. Given the volume of data generated from searches, the main findings are summarised with key references.

*i. Anxiety*

Anxiety disorders and related states, including panic disorder, agoraphobia, social phobia, specific phobia, post-traumatic stress disorder (PTSD), obsessive–compulsive disorder (OCD), and generalised anxiety disorder (GAD), were more frequent across all age ranges in ASD compared to non-ASD participants (see Supplement S2, Section S1.1). Prevalence data from an umbrella review showed that anxiety disorders were present in between 1 and 54% [39], with a pooled prevalence estimate of 35% of people with ASD presenting with an anxiety disorder [19]. Larger population-based studies based on insurance claims data, for example, suggested that people with ASD were 6 times more likely to present with an anxiety disorder than non-ASD cohorts [29].

*ii. Mood disorders including depressive disorders*

Various mood disorders including depression and bipolar disorder (BD) were elevated in ASD [18,33]. Prevalence rates for the general categorisation of mood disorder were particularly elevated in adults with ASD, typically in the region of 20–50% of ASD participants (see Supplement S2, Section S1.2). Data in one study of older adults (aged above 65 years) based on health insurance claims reported a mood disorder rate of 35% in those with ASD compared with 9% of those without [31]. A pooled prevalence of 19% for mood disorder (ranging between 11 and 28%) was reported [19]. Specific data on rates of depression, including major depressive disorder was also consistently higher in the ASD group [30]. Over 16% of children aged 3–17 years reported depression based on a national survey method [34]. Survey data on depression when progressing into adulthood revealed upwards of 20–35% of adults with ASD reported depression [40], with an estimated pooled prevalence of 18% (range 15–21%) [19]. Women with ASD were particularly prone to mood disorders and depression compared with men with ASD and non-ASD women [41]. The presence of bipolar disorder (BD) was also more frequently present in ASD. Reported prevalence rates for BD range from 1.3% to 25% [25,42] compared to non-ASD rates typically around 1–2% in comparison. A pooled prevalence of 7% (ranging between 4 and 9%) for BD was reported [19]. Given the potential for diagnostic overlap between various anxiety and mood disorders with ASD (see Figure 2), disentangling autistic traits from specific psychiatric conditions, and vice versa, is a complicating variable potentially affecting the data presented.

*iii. Schizophrenia spectrum disorders (SSD)*

The presence of SSD, including schizophrenia and other psychosis related conditions were often highly elevated in those with ASD compared to non-ASD participants [6] (see Supplement S2, Section S1.3). In some studies, rates were 25–30 times those recorded in non-ASD participants [31,35], with an umbrella review reporting any SSD as being present in 4–67% of cases [39]. A pooled prevalence rate of 10% (ranging between 7 and 13%) was reported for schizophrenia [19]. Adult rates of psychotic disorder in one insurance-based study reported a prevalence of 6.3% in the ASD group versus 0.5% in the non-ASD group [30]. Studies looking at children with ASD also reported an increased frequency of SSD [35].

*iv. Personality disorders*

Including several types of personality disorder (PD), the collected data suggest that PD may be over-represented in ASD (see Supplement S2, Section S1.4). Although still a topic requiring further study, the current best estimate range for PD in ASD is between 4 and 10%, with a pooled prevalence estimated at 7% [19].

*v. Substance and addiction related disorders*

Data on substance use and addiction disorders (SUAD), including but not limited to alcohol, tobacco and illicit drug use, showed mixed results in relation to any universally elevated risks appearing alongside ASD (see Supplement S2, Section S1.5). In general, rates of substance use disorder (SUD) were equivalent or lower in ASD compared to non-ASD participants [29], with an estimated pooled prevalence of 5% (ranging between 2 and 11%) [19]. Given the spectrum of abilities and disabilities that ASD encompasses and how, particularly for more severe presentations, the opportunities to engage in SUAD are more limited, the data probably reflect this, albeit with further studies required. Specific patterns of alcohol abuse were noted in various studies [30].

*vi. Trauma- and stressor-related conditions*

Post-traumatic stress disorder (PTSD) alongside observations on the number of significant trauma events were the primary focus in this area (see Supplement S2, Section S1.6). Interpretation of the data was hindered by the lack of control groups in many studies, despite showing an estimated PTSD prevalence rate anywhere between 0 and 45% [43,44]. An estimated 1% of people with autism were diagnosed with current PTSD [40] (alongside a 5% lifetime rate). Study recruitment processes, whereby participants with ASD would be aware of the trauma focus of studies, may also be an influencing variable, potentially attracting more people who have experienced trauma than those who have not.

*vii. Feeding and eating disorder*

Multiple feeding and eating issues were identified in our review (see Supplement S2, Section S1.7). This manifests not only as a risk for nutritional deficiencies such as scurvy and other functional issues such as pica but also as eating disorders including anorexia nervosa (AN) and Avoidant/Restrictive Food Intake Disorder (ARFID). The overall estimated frequency of any eating disorder, based on data from multiple studies in ASD, was between 4 and 52% of cases [45], with an estimated pooled prevalence of 5% [19]. Importantly, various feeding and eating disorders were present across chronological ages. There were signals of an over-representation of such issues in girls and women with autism [41].

*viii. Sleep–wake disorders*

Sleep–wake disorders, including dyssomnia, sleep-disordered breathing and more general categorisations of sleep disorders, were frequent across the studies analysed (see Supplement S2, Section S1.8). Frequency estimates from the collected data provide wide ranges in relation to overall sleep issues due to the types of data collected and the various sleep issues detailed. One meta-analysis of observational studies estimated between 36 and 50% of people with ASD have sleep–wake issues with a pooled prevalence of 43% [19].

*ix. Self-harm-related conditions*

The prevalence estimates for self-harm behaviours and conditions, including non-suicidal self-injury and suicidality, were notable (see Supplement S2, Section S1.9). Around 25% of children aged between 4 and 8 years were reported to present with at least some forms of self-injurious behaviours [11]. Data for adults were typically more focused on suicidality, including suicide ideation—estimated at between 10 and 66%—and/or suicide attempts (1–35%) [39] based on an umbrella review. Caution is required in this area, given the potential focus on high-risk populations and overlap with other psychiatric comorbidities.

*x. Neurocognitive disorders*

Parkinson’s disease (PD) and the various conditions headed under the term dementia predominated the research literature under this heading (see Supplement S2, Section S1.10). All the collected literature analysed on PD observed it to be more frequent in people with ASD compared to non-ASD cohorts [25,30,31]. The magnitude of the increased risk based on comparative risk and odds ratio data across different studies suggested PD to be between 1.5 and 6 times more likely in ASD [25,31]. Chronological age was also an important variable. Data on dementia, including Alzheimer’s disease, showed a similar pattern of enhanced risk albeit not with the same magnitude as that noted with PD. One study, based on insurance records, reported a diagnosis of dementia in around 2% of cases compared with under 1% of non-autistic controls [30]. Worryingly, however, early-onset dementia was highlighted in one study as being elevated in ASD [46].

**C. Behavioural and/or neurodevelopmental comorbidity**

Details of the numerous studies included under the heading ‘Behavioural and/or neurodevelopmental comorbidity’ are shown in Supplement S3. Given the volume of data generated from searches, the main findings are summarised with key references.

*i. Cognitive and learning (intellectual) disorders*

Cognitive disorders, including delirium, dementia, and amnesia (overlapping with previous section results), were all more frequently elevated in people with ASD compared to non-ASD cohorts (see Supplement S3, Section S1.1). Based on medical insurance data [29,31], the prevalence risk in adults (18–64) and older adults (65 years+) with ASD ranged between 3 and 8 times more common than in non-ASD individuals. Intellectual or learning disability (ID) is one of the most common comorbid conditions appearing alongside ASD. The prevalence range across various studies was between 0 and 90% depending on criteria used and the specific ASD population studied [17]. The largest meta-analysis of pooled observational studies showed an estimated prevalence of 33% [19] and a range between 26 and 41% frequency. Mild ID was typically the most common severity level based on data from 2006 to 2010 [47] among those with comorbid autism and ID. The data on sex differences was slightly mixed, although potentially skewed towards a greater risk of ID in females compared with males [28]. Distinct from ID but influenced by the presence of ID, learning disorders, which incorporate specific impairments in speaking, reading, writing and arithmetic skills, were also reported to be more frequent in ASD cohorts (13%, range 8–20%) [19]. Such issues require caution in their interpretation considering how ASD can manifest in early age cohorts.

*ii. Disorders of attentional controls*

Disorders of attentional controls including attention-deficit disorder (ADD)/attention-deficit hyperactivity disorder (ADHD), conduct and oppositional disorders are all over-represented and frequently comorbid with ASD (see Supplement S3, Section S1.2). ADD and ADHD, incorporating the various manifestations and subgroupings included in these conditions, were present in an estimated 37% of people with ASD [48] (ranging from 28 to 46% [19]). Studies comparing ASD and non-ASD cohorts showed some variability across different datasets but typically reported over-representation of such attentional disorders in ASD [30]. Such conditions were more frequently present in boys than girls [28], but this may be indicative of any under-representation of assessment in girls. Rates of conduct, oppositional and disruptive disorders (see Supplement S3, Section S1.2.2) outside of attentional problems were also frequently more prevalent in ASD compared with non-ASD populations [18]. They ranged from 21 to 36% with a pooled prevalence estimate of 28% [19].

*iii. Speech and language disorders*

Language disorders are a common comorbidity in ASD, with prevalence rates varying based on the age of the population studied and the diagnostic methods used (see Supplement S3, Section S1.3). Many studies focused on language disorders in young children with ASD, highlighting a pooled frequency estimate of 16% [19] (range 0–53%) based on meta-analysis data. Other studies based on administrative data observed that the prevalence of language disorder in the ASD group was 26% at age 4, increasing to 34% at age 8 [11], suggesting that increasing age in childhood may highlight increasing disparities in this area compared with non-ASD populations. Other, smaller-scale data on the presence of social communication disorder (often termed social pragmatic communication disorder, SCD, in the current DSM iteration) is scarce but suggests an over-representation in ASD [49] albeit with comparative data in non-ASD populations currently lacking.

*iv. Motor disorders*

Encompassing developmental coordination disorder (DCD) (also known as dyspraxia), tic and movement disorders and somatic conditions affecting motor control, including cerebral palsy (CP), various motor disorders are more frequently present in ASD compared to non-ASD populations (see Supplement S3, Section S1.4). The estimated prevalence of diagnosed DCD in childhood ASD is 15% [50], with a larger percentage of children showing motor impairments and thus at heightened risk of a diagnosis of DCD. Motor issues overall, based on pooled prevalence, were observed in around 40% of children but with wide boundaries for potential risk (range: 19–64%) [19]. Tic disorders, including those overlapping with Gilles de la Tourette syndrome, were similarly over-represented when compared with non-ASD cohorts; typically present in 8–13% of people with ASD [19] (with a pooled prevalence of 10%) depending on specific criteria used. Estimates on CP in cases of ASD ranged between 1 and 13% [34,51].

The data included here represent only a sample of the plethora of comorbidities that can potentially present alongside a diagnosis of autism. Our focus on specific categories and specific conditions does not imply that other comorbid conditions cannot be present and cannot exert an equally important singular or cumulative effect on the individual experiences of people with autism.

## 4. Discussion

Multiple traits, states, and conditions spread across the overlapping domains of somatic, psychiatric and neurodevelopment disorders are potentially over-represented when a diagnosis of autism is present (see Table 1). The variable frequency and magnitude of the over-representation highlighted in our narrative review provide important evidence for the potential need for further preferential screening when a diagnosis of autism is given (see Figure 3). Caution is, however, needed around how age, presence and level of intellectual disability, diagnostic methods, data source, and other study sample characteristics may influence such over-representation as detailed in the current literature.

Such preferential screening at various time points post-diagnosis is similarly warranted because of differing onset patterns of specific age-related comorbidities into adolescence, adulthood and beyond. Initial evidence for the clustering of specific conditions is also present, albeit with the requirement for further study. This implies that screening for specific categories of potential comorbidity clustering may well be required whether in a similar discipline (e.g., immunological) or across different disciplines where different comorbidities may intersect. Further study is also required on how such connections are present and related to autism, particularly in relation to any overlapping genetic and biochemical mechanisms.

Several intra-autism variables were also identified as modifiers of risk of such comorbidity. Children with autism, girls, and women with autism and those presenting with more serious and persisting autism severity seem to be particularly prone to increased likelihood of various comorbidities and their clustering(s) in keeping with other reviews [52]. While comorbidity screening is important for individuals diagnosed with autism, we recognise that the magnitude of the risk for developing a comorbidity is not evenly distributed among the very heterogeneous label of autism. Further research is required on this issue.

The wide array and often serious impacts of overlapping comorbidities included in our review suggest that additional screening should be a priority. Moves towards formulating and rolling out a systematic ‘roadmap’ for the identification of any comorbidity would lead to earlier identification and treatment/management strategies where available. This will have important effects on quality-of-life indicators on the basis that many of the identified comorbidities already have their own important, often negative, effects which further impact on the various disabilities inherent to a diagnosis of autism. That many of the over-represented conditions linked to autism also have either an element of physical and/or psychological pain or serious discomfort and/or, in the extreme, the potential to affect patterns of mortality, adds to the urgency in devising such a roadmap.

Whilst our narrative review provides an up-to-date analysis of the autism comorbidities, we are conscious that our findings have previously been individually reported. Issues with GI functions, whether in state or trait, have, for example, a long scientific and clinical history in relation to autism. For whatever reasons, such findings have not received the wider attention that is often merited. Such an issue has also contributed to various health inequalities which are, unfortunately, still a feature of many experiences of autism.

Given the multiple conditions that are over-represented when autism is diagnosed, an important question is why? What potential mechanisms are at work to elevate such risks, and can they provide clues to the underlying biological processes that enable the manifestations of some autisms? The answers are likely to be complicated and not universal. Where, for example, autism presents as part of a genetic syndrome—often included as part of a suite of different issues—it is likely that some of the genetic processes which control a relationship with autism are similarly likely to overlap with other conditions. So, for example, autism appearing alongside DiGeorge syndrome (22q11.2 deletion syndrome), where congenital heart issues, immune system weaknesses and heightened risk for diagnostic progression to schizophrenia are also present, potentially highlights a role for the specific deletion on chromosome 22q11.2 that characterises the condition. Where autism, for example, appears in the context of an acquired insult such as that related to prenatal valproate exposure, one might assume that additional comorbidity in that context summed under the description ‘foetal valproate syndrome’, for example, may also be linked to said exposure. This is particularly important in the context that there are known biological actions attributed to valproate chemistry linked to its role as an HDAC (histone deacetylase) inhibitor (an epigenetic medicine) and its interference with folate transport and metabolism among other biological processes. Other comorbidity patterns around distinct types of autism (e.g., as part of a suite of neurological and psychiatric symptoms with regressive onset following autoimmune encephalitis) may well provide additional information on how comorbidity aetiology and biochemistry might also inform autism aetiology and biochemistry too. Mention of immune functions, particularly a trend towards ‘overactive’ immune functions in the context of hyper- or autoimmunity, likewise offers potentially valuable information about underlying pathology for some [15]. Whether there is, however, a unified mechanistic model which unites both the heterogeneity of autism and the variable range and presence of different comorbidities remains unanswered at the present time.

A related perspective is how treating certain comorbidities in the context of autism can sometimes have important effects on the presentation of the core symptoms of autism. There are numerous examples of such outcomes in relation to epilepsy and seizure disorders, GI conditions (both functional and pathological) and across various biological parameters included under the headings of mitochondrial and neuroinflammatory comorbidities, for example. The so-called ‘gut–brain axis’ provides probably the most well-researched link for this process, where treating particularly functional bowel issues such as constipation, bloating and related issues, via pharmacological and/or dietary means, has, in some studies [53,54], shown a connection with a reduction in core autistic features. Part of this issue is likely to centre around the experience of pain and discomfort which accompanies many, if not all, GI conditions. Part of it is, however, likely to be more central to the biology of some autisms. We caution, however, that further research is needed on the extent of effects of any intervention options for the wide range of potential comorbidities present alongside autism and any effects on core feature presentations.

Several other prominent issues are relevant to our findings. The presence of intellectual (learning) disabilities (ID) as well as being an important comorbidity for autism may also complicate the screening for certain comorbidities. Whether directly, through effects associated with reduced cognitive and adaptive skills and/or potentially due to heightened risk for diagnostic overshadowing, even potentially mild ID has the potential to frustrate efforts of comorbidity screening. In such cases, a lower threshold of suspicion for various comorbidities may be required, alongside efforts to increase communicative and self-report skills to ensure important symptoms are not missed or misdiagnosed.

It is also highly likely that within the context that some autisms may be ‘non-persisting’ in terms of symptoms continually reaching diagnostic thresholds [5,55], particularly behavioural and/or psychiatric comorbidity, for some this will potentially mean that a childhood diagnosis of autism may well be eventually viewed as a ‘prodromal’ diagnosis. Having already highlighted such a preliminary issue in the context of schizophrenia [6] and acknowledging that a dual diagnosis is possible across multiple areas, work remains to be done on what potential prodromal means for comorbidity as a future primary diagnosis and any shared overlapping genetics and biology as potential clues to such events. Again, further studies are warranted around specific phenotypes of autism where such conditions may be more pertinent.

Whilst we assume that rates of many of the various comorbidities highlighted are relatively fixed in terms of their risk following autism, we prioritise continuing research on prevalence and detection rates of the multiple issues identified. Where environmental and lifestyle issues, for example, can influence risk of comorbidity (e.g., T2D), more regular monitoring may be required considering potentially more dynamic comorbidity rates. Such continued monitoring may also be required for other, more delicate reasons related to social vulnerability, in the context of data observing a potential connection between some autism presentations and functional disorders such as functional tics [56] present in part due to social contagion and enhanced vulnerability to such phenomena. Additional demographic variables in relation to sexual and gender diversity into late childhood and adulthood [57] may also be relevant to further study on comorbidity profiles.

Several important limitations of our study and analysis require mention. Our search strategy was broad, reflective of the huge array of comorbidities that can and do present alongside autism. We have provided details of our search strategy but, in keeping with our narrative review, realise the limitations compared with other more formalised systematic review methods. Heterogeneity in the range, type and risk of various comorbidities seemingly follows the heterogeneity present in autism. Many of the reported prevalence estimates we included, for example, span wide ranges, likely influenced by differences in diagnostic criteria, study populations, age distribution, study setting, methods of case ascertainment, and healthcare systems. We did not provide any framework for synthesising such heterogeneity according to age, diagnostic criteria, healthcare systems, and/or ascertainment methods. Additionally, we combined evidence from administrative claims databases, ICD-based registries, electronic health records, population-based cohorts, and clinically confirmed cohorts, all of which differ in case ascertainment, diagnostic accuracy, and susceptibility to misclassification. Although we have provided extensive prevalence data, we did not formally operationalise the clinical screening utility nor provide any framework for stratification, subgrouping, precision screening, or phenotype clustering outside of our mention of age- and sex-based risks. We have not provided any data in relation to practical screening algorithms, priority screening tiers, age-specific recommendations (outside of what is presented) or clear ‘red-flag’ indicators. Finally, there is the concern that individuals with autism may interact differently with healthcare systems than those without, given that individuals with autism are potentially more likely to have more frequent doctor’s appointments than their peers and such more frequent interactions with their doctor make it less likely that a comorbidity is missed. Consequently, some of the observed differences in prevalence may partly reflect increased diagnostic opportunity rather than true differences in disease frequency. This is something that requires further study.

## 5. Conclusions

As inherently disabling as the core features of a diagnosis are, the experience of autism is often compounded by the presence of multiple potential comorbidities with similar life-affecting levels of disability working in an additive fashion to reduce quality of life. We identified a myriad of different over-represented conditions, states, diseases, and disorders that can follow a diagnosis of autism based on the extant scientific literature and in keeping with previous scientific reviews. Differing onset and risk profiles across different comorbidities were also present intra-autism, with children, girls, and women with autism, and those with more severe presentations of autism at particularly heightened risk for various comorbidities and comorbidity combinations. Many, if not all, the comorbidities described will impact on functioning and are additive to the disability that is already implied by a diagnosis of autism. Some comorbidities are treatable assuming timely and correct identification. Said identification will also eventually need to be formally systematised via screening protocols, something which represents a next important stage to ensure health inequalities are minimised in the context of the wide array of potential comorbidities and their effects on quality of life for people with autism.

**Additional notes:** We use ‘autism spectrum disorder (ASD)’ and ‘autism’ interchangeably in this manuscript. We use the terms ‘person or people with autism or ASD’ and ‘autistic person or people’ interchangeably in this manuscript.

## Figures and Tables

**Table 1:** Summary of comorbidities.

Condition	Estimated ASD Prevalence (%) (and/or Range)	Estimated Non-ASD Prevalence (%) (and/or Range)
**A. Somatic [Supplement S1]**		
(i) Gastrointestinal (GI) (any)	48 (0–67)	(3–27)
Constipation	26 (1–65)	(1–11)
Diarrhoea	20 (2–64)	(1–8)
Reflux	8 (1–19)	(1–12)
(ii) Immunological [Supplement S1, Section S1.2]		
Autoimmune condition (any)	(1–24)	(1–23)
Celiac (coeliac) disease	4 (1–9)	
Allergy (any)	(15–62)	(17–29)
Food allergy	13 (1–29)	5 (1–10)
(iii) Metabolic issues [Supplement S1, Section S1.3]		
Metabolic disorder	3 (1–5)	
Type-2 diabetes	(7–36)	(4–27)
(iv) Neurological [Supplement S1, Section S1.4]		
Epilepsy/seizure disorder (all)	16 (0–77)	(1–14)
(v) Motor-sensory conditions [Supplement S1, Section S1.5]		
Joint hypermobility	22	
Ehlers-Danlos syndrome (women)	6	~1
Auditory disorder(s)	(2–94)	
Visual disorder(s) (children)	(0–14)	
**B. Psychiatric [Supplement S2]**		
(i) Anxiety (any) [Supplement S2, Section S1.1]	35 (1–54)	(4–22)
(ii) Mood disorders [Supplement S2, Section S1.2]	19 (11–28)	(1–18)
Depression	18 (15–21)	(4–28)
Bipolar disorder	7 (1–25)	(1–14)
(iii) Schizophrenia spectrum disorders (all) [Supplement S2, Section S1.3]		
	(4–67)	
Schizophrenia	10 (7–13)	(1–11)
(iv) Personality disorder [Supplement S2, Section S1.4]		
	7 (4–10)	(1–2)
(v) Substance and addiction related disorders [Supplement S2, Section S1.5]		
	5 (2–11)	
(vi) Trauma and stressor related conditions [Supplement S2, Section S1.6]		
Post-traumatic stress disorder (PTSD)	1	
(vii) Feeding and eating disorder [Supplement S2, Section S1.7]		
	5 (4–52)	
(viii) Sleep-wake disorders [Supplement S2, Section S1.8]		
	43 (36–50)	
(ix) Self-harm related conditions [Supplement S2, Section S1.9]		
	24	
Suicidality (ideation)	(10–66)	
Suicidality (attempts)	(1–35)	
(x) Neurocognitive disorders [Supplement S2, Section S1.10]		
Parkinson’s disease (PD)	(~1–6)	~1
Dementia	2	~1
**C. Behavioural and/or neurodevelopmental comorbidity [Supplement S3]**		
(i) Cognitive and learning (intellectual) disorders		
Intellectual/learning disability [Supplement S3, Section S1.1]		
	33 (26–41)	(1–2)
Learning disorder	13 (8–20)	
(ii) Disorders of attentional controls [Supplement S3, Section S1.2]		
	37 (28–46)	(1–16)
(iii) Speech and language disorders [Supplement S3, Section S1.3]		
	16 (0–53)	(1–2)
(iv) Motor disorders [Supplement S3, Section S1.4]		
Developmental coordination disorder (DCD)	15	
Motor issues	40 (19–64)	−3
Tic disorders	10 (8–13)	(1–2)
Cerebral Palsy	(1–13)	

## Data Availability

No new data were created or analysed in this study. Data sharing is not applicable to this article.

## Supplementary Materials

The following supporting information can be downloaded at: https://www.mdpi.com/article/10.3390/healthcare14162492/s1, Supplement S1: Somatic; Supplement S2: Psychiatric; Supplement S3: Behavioral and Neurodevelopmental.
