# Supplementary material for: Somatic, Psychiatric and Neurodevelopmental Comorbidities in People with Autism Spectrum Disorder (ASD): A Narrative Review
Source: Healthcare (Basel). 2026 Aug 11;14(16):2492. doi: 10.3390/healthcare14162492 (PMC13512144; doi:10.3390/healthcare14162492)
Supplement: Supplementary file 1 [file healthcare-14-02492-s001.zip › 100d Suppl S1 Comorbidity_Papers___Somatic___Clean.pdf]

# Supplement S1: Somatic

# 1 Somatic Comorbidity

## 1.1 Gastrointestinal (GI)

Table 1: Prevalence of GI in those with ASD, table 1.

| Source | Sample | Prevalence | Other Notes |
|---|---|---|---|
| Croen et al. (2015) [25] | n=1507 (ASD), n=15070 (age, sex matches controls); Adults; Kaiser Permanente Northern California; 2008-12 | GI disorders, any: 34.70% (ASD), 27.47% (non-ASD) | Chi-square p value: <0.001 |
| Cawthorpe (2016) [17] | n = 763,449 (total); n=583 (ASD, female), n=1457 (ASD, male), n=414185 (non-ASD, female), n=348,506 (non-ASD, male); 1994-2009; Canada; ICD-9 | Disease of the digestive system: 60.15% (ASD), 49.46% (non-ASD) [calculated by me from OR table - not reported directly] | Female: OR: 2.04 (95% CI: 1.71-2.43), Male: OR: 1.5 (95% CI: 1.35-1.66) |
| Vohra et al. (2017) [123] | n=1772 (ASD), n=5316 (non-ASD); Age: 22-64; three state Medicaid Analytic eXtract; 2000-08 | GI disorder: 12.6% (ASD), 11.0% (non-ASD) | AOR (95% CI): 1.17 (0.99, 1.38), p: <0.001 |
| Cawthorpe (2018) [18] | n=609 (ASD, male), n=111 (ASD, female), n=28588 (non-ASD, male), n=17,787 (non-ASD, female); Age: <3 between April 1, 1993 and December 31, 1994; 16 year study, max age: 18; ICD-9 | | Digestive system: OR: 1.56, p:<0.05 (males), OR: 1.92, p:<0.05 (females) |
| Davignon, et al. (2018) [29] | n=4123 (ASD), n=20615 (non-ASD, non-ADHD); Age: 14-25; Kaiser Permanente Northern California, 2013-15 | GI: 10.8% (ASD), 8.4% (non-ASD) | aOR (95% CI): 1.29 (1.16 - 1.45) |
| Vargason et al. (2019) [121] | n=3253 (ASD), n=278370 (non-ASD); Children; OptumLabs Data Warehouse, medical claims; 2000-15 | GI diagnosis: 37.0% (ASD), 20.0% (non-ASD) | |

Table 2: Prevalence of GI in those with ASD, table 2.

| Source | Sample | Prevalence | Other Notes |
|---|---|---|---|
| Smith DaWalt et al. (2021) [30] | n=2187 (ASD) [n=1716 (male), n=471 (female)], n=21870 (non-ASD, age and sex matched) [n=17160 (male), n=4710 (female)]; Age:>18; EHRs - Marshfield Clinic - Wisconsin; ICD-9 codes | Gastrointestinal disorders: 0.69 (female, ASD), 0.61 (male,ASD), 0.42 (female, control), 0.36 (male, control) [predicted probability] | OR: 2.78 (95% CI: 2.51-3.08), p: <0.001 [ASD status], OR: 1.06 (95% CI: 0.85-1.33) [ASD X Sex] |
| Bougeard et al. (2021) [11] | Review: N=7 | GI, Range: 0% - 67.8% | |
| Angell et al. (2021) [4] | n=18355 (ASD, girls), n=65145 (ASD, boys); Age: 1-21; healthcare claims data; integrates ICD-9 and ICD-10 codes | Gastrointestinal disorders: 54.76% (ASD, girls), 48.99% (ASD, boys) | aOR: 1.28 (95% CI: 1.24-1.33), p:<0.001 - adjusted for age, race, ethnicity, urbanicity, and insurance |
| Micai et al. (2023) [79] | Meta-analysis of observational studies, N =18 [N = 11 (clinical studies from community samples), N=7 (population/registry based)], n=27249 | Gastrointestinal disorder: Pooled prevalence: 21%, Range: 11% - 33% | |
| Micai et al. (2023) [79] | Meta-analysis of observational studies, N=43 [N = 37 (clinical studies from community samples), N=6 (population/registry based)], n=23908 | Gastrointestinal problem: Pooled prevalence: 39%, Range: 32% - 46% | |
| Ward et al. (2023) [127] | n=1129 (ASD), n=1176 (non-ASD); Adults; Online survey | Any GI: 38.56% (ASD), 20.25% (non-ASD) | p-value: <0.0001 |

Table 3: Prevalence of GI in those with ASD, table 2.

| Source | Sample | Prevalence | Other Notes |
|---|---|---|---|
| Steinhausen et al. (2024) [108] | n=12063 (ASD), n=41251 (non-ASD); Danish register; birth cohort 1984-95; ICD-8 and ICD-10 | | Digestive system diseases (HR, 95% CI): 1.42 (1.32–1.54) (Age: 0–4), 1.71 (1.57–1.88) (Age: 5–10), 1.33 (1.27–1.40) (Age: 11 +) [ASD vs ref, total]; 1.40 (1.28–1.52) (Age: 0–4), 1.72 (1.55–1.92) (Age: 5–10), 1.28 (1.20–1.36) (Age: 11 +) [Males]; 1.53 (1.29–1.82) (Age: 0–4), 1.69 (1.43–2.00) (Age: 5–10), 1.42 (1.32–1.54) (Age: 11 +) [Females]; 0.54 (0.45–0.64) (Age: 0–4), 0.80 (0.67–0.95) (Age: 5–10), 1.49 (1.37–1.63) (Age: 11 +) [Female vs Male, ASD]; 0.49 (0.45–0.54) (Age: 0–4), 0.81 (0.73–0.90) (Age: 5–10), 1.34 (1.28–1.41) (Age: 11 +) [Female vs Male, non-ASD] |
| Al-Ayadhi et al. (2025) [2] | n=363 [n=189 (ASD), n=174 (non-ASD)]; Online questionnaire (self or caregiver); Saudi Arabia; August 2022 - January 2023 | GI disease: 11.1% (ASD), 3.4% (non-ASD) | p: 0.005 |

### 1.1.1 GI Symptoms

Table 4: Prevalence of GI symptoms in those with ASD.

| Source | Sample | Prevalence | Other Notes |
|---|---|---|---|
| Aldinger et al. (2015) [3] | n=728 (ASD, children), n=694 (parents), n=184 (non-ASD, siblings); Autism Genetic Resource Exchange | GI disturbances: 42.4% (ASD, children), 26.5% (parents), 12.2% (non-ASD, siblings) | |
| Fulceri et al. (2016) [39] | n=115 (ASD), n=115 (control); Age: Preschool; Parental report | GI symptoms: 37.4% (ASD), 14.8% (non-ASD) | |
| Lai et al. (2020) [67] | n=107 (ASD), n=249 (non-ASD), Children; China | Any GI symptom: 26.2% (ASD), 10.4% (non-ASD) | p-value:$<$0.001 |
| Wang et al. (2022) [124] | Meta-analysis, N= 63, n=131,416 ASD | GI symptom: 48.67% | (95% CI: 43.50–53.86, $I^2$ = 99.51%) |
| Leader et al. (2022) [69] | Review, N=30 | GI symptoms: Range: 30% - 37.4%, 82.4% | |
| Lasheras et al. (2023) [68] | Review, N=8; Pediatric | GI symptoms: 33%, Range: 0% - 69% | (95% CI, 13%–57%) |

### 1.1.2 Constipation

Table 5: Prevalence of constipation in those with ASD

| Source | Sample | Prevalence | Other Notes |
|---|---|---|---|
| Croen et al. (2015) [25] | n=1507 (ASD), n=15070 (age, sex matches controls); Adults; Kaiser Permanente Northern California; 2008-12 | 4.45% (ASD), 1.39% (non-ASD) | Chi-square p value: <0.001 |
| Davignon, et al. (2018) [29] | n=4123 (ASD), n=20615 (non-ASD, non-ADHD); Age: 14-25; Kaiser Permanente Northern California, 2013-15 | 1.7% (ASD), 0.5% (non-ASD) | aOR (95% CI): 3.47 (2.53- 4.75) |
| Schott et al. (2022) [100] | n=155,617 (ASD), n=466,851 (non-ASD); Adults; Medicaid; 2008–12 | Constipation: 15.11% (ASD), 11.10% (non-ASD) | aOR: 1.35 (1.32 1.38) |
| Wang et al. (2022) [124] | Meta-analysis; N=63, n=131,416 (ASD) | 26.17% | 95% CI: 22.10–30.45, $I^2$ = 97.22% |
| Leader et al. (2022) [69] | Review, N=30 | 22.1%-65% | |
| Koceski and Trajkovski (2022) [65] | n=72 (ASD), n=75 (non-ASD); Age: 3-24; Rochester Health Status Survey IV, Macedonia; 2019 | Constipation: 31.9% (ASD), 10.6% (non-ASD) | p: 0.02 |
| Ward et al. (2023) [127] | n=1129 (ASD), n=1176 (non-ASD); Adults; Online survey | Chronic constipation: 10.11% (ASD), 3.75% (non-ASD) | p-value: <0.0001 |
| Kennedy et al. (2024) [62] | n=246 (ASD); Connecticut Children's Medical Center ASD Assessment Program electronic records; 2018-19 | 14.6% | |
| Austin et al. (2025) [7] | Review, N=44, n=17924 (ASD), Children, 18 countries | Constipation: Mean: 29.8%, Range: 3.5%-79.5% | |

### 1.1.3 Diarrhea

Table 6: Prevalence of diarrhea in those with ASD.

| Source | Sample | Prevalence | Other Notes |
|---|---|---|---|
| Croen et al. (2015) [25] | n=1507 (ASD), n=15070 (age, sex matches controls); Adults; Kaiser Permanente Northern California; 2008-12 | 5.24% (ASD), 3.38% (non-ASD) | Chi-square p value: 0.002 |
| Davignon, et al. (2018) [29] | n=4123 (ASD), n=20615 (non-ASD, non-ADHD); Age: 14-25; Kaiser Permanente Northern California, 2013-15 | 1.7% (ASD), 0.9% (non-ASD) | aOR (95% CI): 1.68 (1.27 - 2.23) |
| Schott et al. (2022) [100] | n=155,617 (ASD), n=466,851 (non-ASD); Adults; Medicaid; 2008–12 | Diarrhea: 9.8% (ASD), 8.49% (non-ASD) | aOR: 1.06 (1.03 1.09) |
| Wang et al. (2022) [124] | Meta-analysis; N=63, n=131,416 (ASD) | 19.92% | (95% CI: 15.97–24.18, $I^2$ = 97.47%) |
| Leader et al. (2022) [69] | Review, N=30 | 10.6% - 64.7% | |
| Ward et al. (2023) [127] | n=1129 (ASD), n=1176 (non-ASD); Adults; Online survey | Chronic diarrhea: 7.80% (ASD), 1.87% (non-ASD) | p-value: <0.0001 |

### 1.1.4 Reflux

Table 7: Prevalence of reflux in those with ASD, table 1.

| Source | Sample | Prevalence | Other Notes |
|---|---|---|---|
| Croen et al. (2015) [25] | n=1507 (ASD), n=15070 (age, sex matches controls); Adults; Kaiser Permanente Northern California; 2008-12 | GERD: 12.81% (ASD), 7.70% (non-ASD) | Chi-square p value: $<0.001$ |
| Davignon, et al. (2018) [29] | n=4123 (ASD), n=20615 (non-ASD, non-ADHD); Age: 14-25; Kaiser Permanente Northern California, 2013-15 | GERD: 1.9% (ASD), 1.1% (non-ASD) | aOR (95% CI): 1.77 (1.36 - 2.32) |
| Kamionkowski et al. (2022) [59] | n=4430 (ASD+GERD), n=33110 (ASD, non-GERD), n=2405750 (non-ASD + GERD), n=38244190 (non-ASD, non-GERD); Age: >18; National cohort - IBMs Explorys database; 2000-19 | GERD: 11.20% (ASD), 5.92% (non-ASD) | p:$<0.0001$ |
| Schott et al. (2022) [100] | n=155,617 (ASD), n=466,851 (non-ASD); Adults; Medicaid; 2008–12 | GERD: 11.63% (ASD), 12.44% (non-ASD) | aOR: 0.85 (0.83 0.87) |
| Wang et al. (2022) [124] | Meta-analysis, 63 studies, n=131,416 (ASD) | GI Reflux: 8.58% | 95% CI: 5.51–12.20, $I^2$ = 93.04% |

Table 8: Prevalence of reflux in those with ASD, table 2.

| Source | Sample | Prevalence | Other Notes |
|---|---|---|---|
| Koceski and Trajkovski (2022) [65] | n=72 (ASD), n=75 (non-ASD); Age: 3-24; Rochester Health Status Survey IV, Macedonia; 2019 | Gastroesophageal reflux: 12.5% (ASD), 9.3% (non-ASD) | p: 0.538 |
| Ward et al. (2023) [127] | n=1129 (ASD), n=1176 (non-ASD); Adults; Online survey | Gastric reflux: 19.77% (ASD), 8.43% (non-ASD) | p-value: p <0.0001 |
| Kennedy et al. (2024) [62] | n=246 (ASD); Connecticut Children's Medical Center ASD Assessment Program electronic records; 2018-19 | GERD: 8.5% | |

### 1.1.5 Other GI Symptoms

Table 9: Prevalence of GI symptoms not listed above in those with ASD, table 1.

| Source | Sample | Prevalence | Other Notes |
|---|---|---|---|
| Wang et al. (2022) [124] | Meta-analysis; N=63, n=131,416 (ASD) | Abdominal pain: 21.38% | 95%CI: 16.07–27.20, $I^2$ = 97.87% |
| Wang et al. (2022) [124] | Meta-analysis; N=63, n=131,416 (ASD) | Bloating: 22.52% | 95% CI: 15.87–29.93, $I^2$ = 97.44% |
| Wang et al. (2022) [124] | Meta-analysis; N=63, n=131,416 (ASD) | Nausea: 25.06% | 95% CI: 10.72–42.85, $I^2$= 98.36% |
| Leader et al. (2022) [69] | Review, N=30 | Nausea: 23.2% - 27.9% | |
| Leader et al. (2022) [69] | Review, N=30 | Vomiting: 4.2% - 11.4% | |
| Wang et al. (2022) [124] | Meta-analysis; N=63, n=131,416 (ASD) | Vomiting: 5.99% | 95% CI: 3.65–8.82, $I^2$ = 93.39% |
| Wang et al. (2022) [124] | Meta-analysis; N=63, n=131,416 (ASD) | Food selectivity: 59.14% | 95% CI: 44.77–72.76, $I^2$ = 95.58% |
| Niemczyk et al. (2018) [83] | Review, N=19 | Nocturnal enuresis: 2%-90%; Daytime urinary incontinence: 4%-55%; Fecal incontinence: 2%-70.6% | |
| Ivanović (2021) [54] | n=152 (ASD); Mean age: 8.02 (4.26); Autism Center, Montenegro | Enuresis/encopresis: 3.29% | |
| Ward et al. (2023) [127] | n=1129 (ASD), n=1176 (non-ASD); Adults; Online survey | Fecal incontinence: 3.72% (ASD), 1.11% (non-ASD) | p-value: <0.0001 |
| Ward et al. (2023) [127] | n=1129 (ASD), n=1176 (non-ASD); Adults; Online survey | Urinary incontinence: 3.99% (ASD), 1.02% (non-ASD) | p-value: <0.0001 |

Table 10: Prevalence of GI symptoms not listed above in those with ASD, table 2.

| Source | Sample | Prevalence | Other Notes |
|---|---|---|---|
| Khachadourian et al. (2023) [63] | n=42569 (ASD), n=11389 (non-ASD siblings); SPARK; Age: <18 at time of registration; 1999-2019 | Encopresis, estimated: 9% (ASD), 1% (non-ASD) | p: <0.05 |
| Khachadourian et al. (2023) [63] | n=42569 (ASD), n=11389 (non-ASD siblings); SPARK; Age: <18 at time of registration; 1999-2019 | Enuresis, estimated: 9% (ASD), 2% (non-ASD) | p: <0.05 |

#### 1.1.6 Functional GI Issues

Table 11: Prevalence of functional GI issues in those with ASD, table 1.

| Source | Sample | Prevalence | Other Notes |
|---|---|---|---|
| Croen et al. (2015) [25] | n=1507 (ASD), n=15070 (age, sex matches controls); Adults; Kaiser Permanente Northern California; 2008-12 | Functional disorders: 13.80% (ASD), 13.77% (non-ASD) | Chi-square p value: 0.97 |
| Davignon, et al. (2018) [29] | n=4123 (ASD), n=20615 (non-ASD, non-ADHD); Age: 14-25; Kaiser Permanente Northern California, 2013-15 | Functional disorders: 4.1% (ASD), 4.7% (non-ASD) | aOR (95% CI): 0.84 (0.71 - 1.00) |
| Schott et al. (2022) [100] | n=155,617 (ASD), n=466,851 (non-ASD); Adults; Medicaid; 2008–12 | Functional disorders: 22.90% (ASD), 25.79% (non-ASD) | aOR: 0.74 (0.73 0.76) |
| Ward et al. (2023) [127] | n=1129 (ASD), n=1176 (non-ASD); Adults; Online survey | Functional pain: 3.55% (ASD), 0.94% (non-ASD) | p-value: 0.00021 |
| Ward et al. (2023) [127] | n=1129 (ASD), n=1176 (non-ASD); Adults; Online survey | IBS: 25.0% (ASD), 11.75% (non-ASD) | p-value: <0.0001 |
| Ward et al. (2023) [127] | n=1129 (ASD), n=1176 (non-ASD); Adults; Online survey | Gastroparesis: 2.22% (ASD), 0.43% (non-ASD) | p-value: 0.0014 |
| Croen et al. (2015) [25] | n=1507 (ASD), n=15070 (age, sex matches controls); Adults; Kaiser Permanente Northern California; 2008-12 | Upper GI motility: 7.56% (ASD), 3.93% (non-ASD) | Chi-square p value: <0.001 |
| Davignon, et al. (2018) [29] | n=4123 (ASD), n=20615 (non-ASD, non-ADHD); Age: 14-25; Kaiser Permanente Northern California, 2013-15 | Upper GI motility problems: 1.8% (ASD), 1.3% (non-ASD) | aOR (95% CI): 1.30 (1.00 - 1.70) |

Table 12: Prevalence of functional GI issues in those with ASD, table 2.

| Source | Sample | Prevalence | Other Notes |
|---|---|---|---|
| Schott et al. (2022) [100] | n=155,617 (ASD), n=466,851 (non-ASD); Adults; Medicaid; 2008–12 | Upper GI motility: 15.28% (ASD), 15.76% (non-ASD) | aOR: 0.85 (0.83 0.86) |
| Hunt et al. (2024) [53] | n=813 (ASD), n=2781 (non-ASD); Age: 6-97; National Center for Mental Health database and volunteers; Wales and UK | IBS: 7.2% (ASD), 3.1% (non-ASD) | p:<0.001 (ASD vs non-ASD); ASD vs non-ASD: aOR: 2.10 (95% 1.06-4.17), p: 0.035 (adjusted for age, gender, antipsychotic use, mood stabilizer, smoking status, COPD, chronic obstructive pulmonary disease); ASD (no ID) vs non-ASD: aOR: 2.05 (95% 1.03-4.09), p: 0.042 (adjusted for COPD, chronic obstructive pulmonary disease) ASD+ID vs non-ASD: aOR: 3.78 (95% 0.70-20.39), p: 0.122 (adjusted for COPD, chronic obstructive pulmonary disease) |
| Austin et al. (2025) [7] | Review, N=44, n=17924 (ASD), Children, 18 countries | Any functional GI disorder: Range: 22.7%-93.2%; Functional abdominal Pain: 14.5%, Range: 0%-59.1%; IBS: 0.3%-11.9% | |

### 1.1.7 Upper and Lower GI

Table 13: Prevalence of upper and lower GI issues in those with ASD, table 1.

| Source | Sample | Prevalence | Other Notes |
|---|---|---|---|
| Hand et al. (2020) [46] | n=4685 (ASD), n=46850 (non-ASD); Age: ≥65; Medicare; 2016-17 | Upper and lower GI disorders: 48.6% (ASD), 26.4% (non-ASD) | aOR: 2.7 (2.5 - 2.9) |
| Longo et al. (2024) [75] | n=30258 (ASD), n=60516 (non-ASD); Age: 18-64; IBM Market Scan Commercial Claims and Encounters Database; 2019-20 | Upper and Lower GI Disorders: 13.7% (ASD), 8.6% (non-ASD) | aOR: 1.7 (95% CI: 1.6, 1.8) |
| Croen et al. (2015) [25] | n=1507 (ASD), n=15070 (age, sex matches controls); Adults; Kaiser Permanente Northern California; 2008-12 | Other diseases of esophagus: 4.58% (ASD), 2.79% (non-ASD) | Chi-square p value: <0.001 |
| Schott et al. (2022) [100] | Adults enrolled in Medicaid in 2008–12 (1:3 ASD to non-ASD) n=155,617 ASD, n=466,851 non-ASD | Other disease of esophagus: 12.71% (ASD), 13.88% (non-ASD) | aOR: 0.87 (0.85 0.89) |
| Liu et al. (2023) [74] | n=5291 (ASD), n=4195596 (non-ASD); Age: >45; Swedish nationwide Total Population Register and National Patient Register; born 1932-67; cumulative incidence uses 1:10 sex and birth year matched cohort | Diseases of esophagus, stomach and duodenum: 5.7% (ASD), 6.5% (non-ASD) [prevalence over follow up period]; 10.5% (ASD), 8.5% (non-ASD) [estimated cumulative incidence by age 70] | aOR: 1.49 (95% CI: 1.33-1.67), p:<0.0001 (ASD vs non-ASD); aOR: 1.52 (95% CI: 1.34-1.73), p:<0.0001 (ASD without ID vs non-ASD); aOR: 1.39 (95% CI: 1.09-1.77), p: 0.0069 (ASD+ID vs non-ASD); p: 0.53 (ASD without ID vs ASD+ID); aOR: 1.50 (95% CI: 1.30-1.73), p:<0.0001 (men with ASD vs men without ASD); aOR: 1.52 (95% CI: 1.27-1.83), p:<0.0001 (women with ASD vs women without ASD); p: 0.90 (men with ASD vs women with ASD) [AORs from cumulative incidence; adjusted for sex and birth year] |

Table 14: Prevalence of upper and lower GI issues in those with ASD, table 2.

| Source | Sample | Prevalence | Other Notes |
|---|---|---|---|
| Croen et al. (2015) [25] | n=1507 (ASD), n=15070 (age, sex matches controls); Adults; Kaiser Permanente Northern California; 2008-12 | Disorders of stomach or duodenum: 1.33% (ASD), 1.46% (non-ASD) | Chi-square p value: 0.68 |
| Schott et al. (2022) [100] | n=155,617 (ASD), n=466,851 (non-ASD); Adults; Medicaid; 2008–12 | Disorders of stomach or duodenum: 9.45% (ASD), 10.72% (non-ASD) | aOR: 0.81 (0.79 0.83) |
| Bishop-Fitzpatrick et al. (2018) [10] | n=91 (ASD), n=6186 (sex, birth year matched controls); Age: 0-89; EHR from Marshfield clinic - decedents - Wisconsin | Liver disease: 5.5% (ASD), 5.5% (non-ASD) | aOR: 0.80 (adjusted for age and sex), p: 0.64 |
| Liu et al. (2023) [74] | n=5291 (ASD), n=4195596 (non-ASD); Age: >45; Swedish nationwide Total Population Register and National Patient Register; born 1932-67; cumulative incidence uses 1:10 sex and birth year matched cohort | Diseases of liver: 1.0% (ASD), 1.3% (non-ASD) [prevalence over follow up period]; 1.3% (ASD), 1.2% (non-ASD) [estimated cumulative incidence by age 70] | aOR: 1.23 (95% CI: 0.94-1.61), p: 0.13 (ASD vs non-ASD); aOR: 1.60 (95% CI: 1.22-2.10), p: 0.0007 (ASD without ID vs non-ASD); aOR: NA, p: NA (ASD+ID vs non-ASD); p: NA (ASD without ID vs ASD+ID); aOR: 1.32 (95% CI: 0.97-1.81), p: 0.076 (men with ASD vs men without ASD); aOR: 1.03 (95% CI: 0.60-1.77), p: 0.92 (women with ASD vs women without ASD); p: 0.43 (men with ASD vs women with ASD) [all aORs based on cumulative incidence; adjusted for sex and birth year] |

Table 15: Prevalence of upper and lower GI issues in those with ASD, table 3.

| Source | Sample | Prevalence | Other Notes |
|---|---|---|---|
| Shedlock et al. (2016) [103] | n=48762 (ASD), n=243810 (non-ASD); children; military healthcare system database, 2000-13 | Nonalcoholic fatty liver disease and nonalcoholic steatohepatitis (NASH): 0.2% (ASD), 0.1% (non-ASD) | OR: 2.74 (95% CI 2.06 - 3.65), p<0.001 |
| Ward et al. (2023) [127] | n=1129 (ASD), n=1176 (non-ASD); Adults; Online survey | Non-alcoholic fatty liver disease: 1.51% (ASD), 0.51% (non-ASD) | p-value: 0.03 |
| Hunt et al. (2024) [53] | n=813 (ASD), n=2781 (non-ASD); Age: 6-97; National Center for Mental Health database and volunteers; Wales and UK | Liver disease: 1.9% (ASD), 0.4% (non-ASD) | p:<0.001 (ASD vs non-ASD); ASD vs non-ASD: aOR: 10.96 (95% 3.72-32.25), p:<0.001 (adjusted for age, gender, antipsychotic use, mood stabilizer, smoking status, COPD, chronic obstructive pulmonary disease); ASD (no ID) vs non-ASD: aOR: 10.42 (95% 3.48-31.17), p:<0.001 (adjusted for COPD, chronic obstructive pulmonary disease) ASD+ID vs non-ASD: aOR: 22.28 (95% 3.75-132.5), p:<0.001 (adjusted for COPD, chronic obstructive pulmonary disease) |
| Bishop-Fitzpatrick et al. (2018) [10] | n=91 (ASD), n=6186 (sex, birth year matched controls); Age: 0-89; EHR from Marshfield clinic - decedents - Wisconsin | Peptic ulcer disease: 9.9% (ASD), 8.0% (non-ASD) | aOR: 1.34 (adjusted for age and sex), p: 0.41 |
| Ward et al. (2023) [127] | n=1129 (ASD), n=1176 (non-ASD); Adults; Online survey | Peptic ulcers: 1.95% (ASD), 1.11% (non-ASD) | p-value: 0.13 |

Table 16: Prevalence of upper and lower GI issues in those with ASD, table 4.

| Source | Sample | Prevalence | Other Notes |
|---|---|---|---|
| Liu et al. (2023) [74] | n=5291 (ASD), n=4195596 (non-ASD); Age: >45; Swedish nationwide Total Population Register and National Patient Register; born 1932-67; cumulative incidence uses 1:10 sex and birth year matched cohort | Intestinal diseases: 1.6% (ASD), 3.3% (non-ASD) [prevalence over follow up period]; 3.4% (ASD), 5.0% (non-ASD) [estimated cumulative incidence by age 70] | aOR: 0.91 (95% CI: 0.74-1.13), p: 0.39 (ASD vs non-ASD); aOR: 1.04 (95% CI: 0.82-1.30), p: 0.76 (ASD without ID vs non-ASD); aOR: 0.52 (95% CI: 0.30-0.92), p: 0.025 (ASD+ID vs non-ASD); p: 0.03 (ASD without ID vs ASD+ID); aOR: 0.79 (95% CI: 0.59-1.07), p: 0.13 (men with ASD vs men without ASD); aOR: 1.07(95% CI: 0.79-1.44), p: 0.67 (women with ASD vs women without ASD); p: 0.17 (men with ASD vs women with ASD) [AORs from cumulative incidence; adjusted for sex and birth year] |
| Liu et al. (2023) [74] | n=5291 (ASD), n=4195596 (non-ASD); Age: >45; Swedish nationwide Total Population Register and National Patient Register; born 1932-67; cumulative incidence uses 1:10 sex and birth year matched cohort | Appendix disease: 0.7% (ASD), 1.2% (non-ASD) [prevalence over follow up period]; 1.3% (ASD), 1.9% (non-ASD) [estimated cumulative incidence by age 70] | aOR: 0.89 (95% CI: 0.65-1.22), p: 0.46 (ASD vs non-ASD); aOR: 1.07 (95% CI: 0.77-1.49), p: 0.68 (ASD without ID vs non-ASD); aOR: NA, p:NA (ASD+ID vs non-ASD); p:NA (ASD without ID vs ASD+ID); aOR: 0.84 (95% CI: 0.56-1.25), p: 0.38 (men with ASD vs men without ASD); aOR: 1.00 (95% CI: 0.60-1.65), p: 0.97 (women with ASD vs women without ASD); p: 0.61 (men with ASD vs women with ASD) [AORs from cumulative incidence; adjusted for sex and birth year] |

Table 17: Prevalence of upper and lower GI issues in those with ASD, table 5.

| Source | Sample | Prevalence | Other Notes |
|---|---|---|---|
| Croen et al. (2015) [25] | n=1507 (ASD), n=15070 (age, sex matches controls); Adults; Kaiser Permanente Northern California; 2008-12 | Lower GI: 3.32% (ASD), 1.61% (non-ASD) | Chi-square p value: <0.001 |
| Schott et al. (2022) [100] | n=155,617 (ASD), n=466,851 (non-ASD); Adults; Medicaid; 2008–12 | Lower GI: 7.94% (ASD), 8.41% (non-ASD) | aOR: 0.97 (0.94 1.00) |
| Ward et al. (2023) [127] | n=1129 (ASD), n=1176 (non-ASD); Adults; Online survey | Diverticulosis: 1.51% (ASD), 0.85% (non-ASD) | p-value: 0.19 |
| Croen et al. (2015) [25] | n=1507 (ASD), n=15070 (age, sex matches controls); Adults; Kaiser Permanente Northern California; 2008-12 | Disease of rectum and anus: 2.59% (ASD), 1.96% (non-ASD) | Chi-square p value: 0.09 |
| Schott et al. (2022) [100] | n=155,617 (ASD), n=466,851 (non-ASD); Adults; Medicaid; 2008–12 | Diseases of rectum and anus: 4.80% (ASD), 4.34% (non-ASD) | aOR: 1.05 (1.02 1.09) |
| Ward et al. (2023) [127] | n=1129 (ASD), n=1176 (non-ASD); Adults; Online survey | Hemorrhoids: 9.66% (ASD), 4.77% (non-ASD) | p-value: <0.0001 |

Table 18: Prevalence of upper and lower GI issues in those with ASD, table 6.

| Source | Sample | Prevalence | Other Notes |
|---|---|---|---|
| Hunt et al. (2024) [53] | n=813 (ASD), n=2781 (non-ASD); Age: 6-97; National Center for Mental Health database and volunteers; Wales and UK | Gastric/duodenal ulcers: 3.5% (ASD), 2.4% (non-ASD) | p: 0.092 (ASD vs non-ASD); ASD vs non-ASD: aOR: 2.37 (95% 1.18-4.76), p: 0.016 (adjusted for age, gender, antipsychotic use, mood stabilizer, smoking status, COPD, chronic obstructive pulmonary disease); ASD (no ID) vs non-ASD: aOR: 2.42 (95% 1.20-4.88), p: 0.013 (adjusted for COPD, chronic obstructive pulmonary disease) ASD+ID vs non-ASD: aOR: 1.45 (95% 0.18-11.75), p: 0.729 (adjusted for COPD, chronic obstructive pulmonary disease) |
| Liu et al. (2023) [74] | n=5291 (ASD), n=4195596 (non-ASD); Age: >45; Swedish nationwide Total Population Register and National Patient Register; born 1932-67; cumulative incidence uses 1:10 sex and birth year matched cohort | Pancreatitis: 0.4% (ASD), 0.9% (non-ASD) [prevalence over follow up period]; 0.8% (ASD), 0.9% (non-ASD) [estimated cumulative incidence by age 70] | aOR: 0.73 (95% CI: 0.48-1.12), p: 0.15 (ASD vs non-ASD); aOR: 0.87 (95% CI: 0.56-1.37), p: 0.55 (ASD without ID vs non-ASD); aOR: NA, p:NA (ASD+ID vs non-ASD); p:NA (ASD without ID vs ASD+ID); aOR: 0.72 (95% CI: 0.43-1.19), p: 0.20 (men with ASD vs men without ASD); aOR: 0.84 (95% CI: 0.27-2.59), p: 0.53 (women with ASD vs women without ASD); p: 0.88 (men with ASD vs women with ASD) [AORs from cumulative incidence; adjusted for sex and birth year] |

Table 19: Prevalence of upper and lower GI issues in those with ASD, table 7.

| Source | Sample | Prevalence | Other Notes |
|---|---|---|---|
| Liu et al. (2023) [74] | n=5291 (ASD), n=4195596 (non-ASD); Age: >45; Swedish nationwide Total Population Register and National Patient Register; born 1932-67; cumulative incidence uses 1:10 sex and birth year matched cohort | Peritonitis: 0.2% (ASD), 0.4% (non-ASD) [prevalence over follow up period]; 0.3% (ASD), 0.4% (non-ASD) [estimated cumulative incidence by age 70] | aOR: 1.18 (95% CI: 0.67-2.07), p: 0.58 (ASD vs non-ASD); aOR: 1.17 (95% CI: 0.61-2.25), p: 0.64 (ASD without ID vs non-ASD); aOR: NA, p: NA (ASD+ID vs non-ASD); p: NA (ASD without ID vs ASD+ID); aOR: 1.39 (95% CI: 0.72-2.67), p: 0.32 (men with ASD vs men without ASD); aOR: NA, p: NA (women with ASD vs women without ASD); p: NA (men with ASD vs women with ASD) [all aORs based on cumulative incidence; adjusted for sex and birth year] |
| Koceski and Trajkovski (2022) [65] | n=72 (ASD), n=75 (non-ASD); Age: 3-24; Rochester Health Status Survey IV, Macedonia; 2019 | Intestinal inflammation: 19.4% (ASD), 8% (non-ASD) | p: 0.043 |

#### 1.1.8 Other GI Conditions

Table 20: Prevalence of other GI issues in those with ASD, table 1.

| Source | Sample | Prevalence | Other Notes |
|---|---|---|---|
| Croen et al. (2015) [25] | n=1507 (ASD), n=15070 (age, sex matches controls); Adults; Kaiser Permanente Northern California; 2008-12 | Hepatic disease: 2.19% (ASD), 1.51% (non-ASD) | Chi-square p value: 0.04 |
| Hand et al. (2020) [46] | n=4685 (ASD), n=46850 (non-ASD); Age: $\geq$65; Medicare; 2016-17 | Non- upper or lower GI condition: 51.2% (ASD), 17.6% (non-ASD) | aOR: 5.2 (4.9-5.5) |
| Schott et al. (2022) [100] | n=155,617 (ASD), n=466,851 (non-ASD); Adults; Medicaid; 2008–12 | Gallbladder disorders: 2.59% (ASD), 3.51% (non-ASD) | aOR: 0.72 (0.68 0.75) |
| Schott et al. (2022) [100] | n=155,617 (ASD), n=466,851 (non-ASD); Adults; Medicaid; 2008–12 | Hepatic disease: 2.81% (ASD), 3.28% (non-ASD) | aOR: 0.87 (0.83 0.91) |
| Croen et al. (2015) [25] | n=1507 (ASD), n=15070 (age, sex matches controls); Adults; Kaiser Permanente Northern California; 2008-12 | Gallbladder disorders: 1.86% (ASD), 1.41% (non-ASD) | Chi-square p value: 0.17 |
| Ward et al. (2023) [127] | n=1129 (ASD), n=1176 (non-ASD); Adults; Online survey | Gallbladder disease: 2.22% (ASD), 0.68% (non-ASD) | p-value: 0.0059 |
| Ward et al. (2023) [127] | n=1129 (ASD), n=1176 (non-ASD); Adults; Online survey | Gallstones: 3.99% (ASD), 1.96% (non-ASD) | p-value: 0.0077 |

Table 21: Prevalence of other GI issues in those with ASD, table 2.

| Source | Sample | Prevalence | Other Notes |
|---|---|---|---|
| Liu et al. (2023) [74] | n=5291 (ASD), n=4195596 (non-ASD); Age: >45; Swedish nationwide Total Population Register and National Patient Register; born 1932-67; cumulative incidence uses 1:10 sex and birth year matched cohort | Cholelithiasis (gallstone): 2.7% (ASD), 4.2% (non-ASD) [prevalence over follow up period]; 5.3% (ASD), 5.1% (non-ASD) [estimated cumulative incidence by age 70] | aOR: 1.12 (95% CI: 0.95-1.32), p: 0.17 (ASD vs non-ASD); aOR: 1.10 (95% CI: 0.92-1.33), p: 0.30 (ASD without ID vs non-ASD); aOR: 1.17 (95% CI: 0.85-1.63), p: 0.33 (ASD+ID vs non-ASD); p: 0.75 (ASD without ID vs ASD+ID); aOR: 1.05 (95% CI: 0.82-1.33), p: 0.72 (men with ASD vs men without ASD); aOR: 1.22 (95% CI: 0.98-1.52), p: 0.081 (women with ASD vs women without ASD); p: 0.36 (men with ASD vs women with ASD) [AORs from cumulative incidence; adjusted for sex and birth year] |
| Liu et al. (2023) [74] | n=5291 (ASD), n=4195596 (non-ASD); Age: >45; Swedish nationwide Total Population Register and National Patient Register; born 1932-67; cumulative incidence uses 1:10 sex and birth year matched cohort | Cholecystitis: 0.6% (ASD), 1.1% (non-ASD) [prevalence over follow up period]; 1.1% (ASD), 1.1% (non-ASD) [estimated cumulative incidence by age 70] | aOR: 1.09 (95% CI: 0.77-1.53), p: 0.63 (ASD vs non-ASD); aOR: 1.09 (95% CI: 0.73-1.61), p: 0.68 (ASD without ID vs non-ASD); aOR: 1.10 (95% CI: 0.55-2.19), p: 0.80 (ASD+ID vs non-ASD); p: 0.98 (ASD without ID vs ASD+ID); aOR: 1.04 (95% CI: 0.66-1.63), p: 0.87 (men with ASD vs men without ASD); aOR: 1.20 (95% CI: 0.71-2.04), p: 0.49 (women with ASD vs women without ASD); p: 0.68 (men with ASD vs women with ASD) [AORs from cumulative incidence; adjusted for sex and birth year] |

Table 22: Prevalence of other GI issues in those with ASD, table 3.

| Source | Sample | Prevalence | Other Notes |
|---|---|---|---|
| Longo et al. (2024) [75] | n=30258 (ASD), n=60516 (non-ASD); Age: 18-64; IBM Market Scan Commercial Claims and Encounters Database; 2019-20 | Other GI: 18.6% (ASD), 10.9% (non-ASD) | aOR: 1.9 (95% CI: 1.8, 1.9) |
| Ward et al. (2023) [127] | n=1129 (ASD), n=1176 (non-ASD); Adults; Online survey | Hernia: 5.14% (ASD), 1.19% (non-ASD) | p-value: p <0.0001 |
| Ward et al. (2023) [127] | n=1129 (ASD), n=1176 (non-ASD); Adults; Online survey | GI polyps: 3.64% (ASD), 1.70% (non-ASD) | p-value: 0.0077 |
| Tint et al. (2023) [113] | n=3220 (ASD, women), n=12920 (no DD, women), n=3217 (other DD, women), n=7426 (ASD, men), n=29687 (no DD, men), n=7398 (other DD, men); Canada; administrative and social services data - ICES | Crohn's disease/colitis: 0.6% (ASD, women), 0.6% (other DD, women), 0.7% (no DD, women), 0.6% (ASD, men), 0.6% (no DD, men), 0.6% (other DD, men) | aRR: 0.99 (95% 0.59-1.64) [ASD vs no DD, women], aRR: 0.78 (95% 0.41-1.45) [ASD vs other DD, women], aRR: 1.03 (95% 0.74-1.43) [ASD vs no DD, men], aRR: 1.02 (95% 0.67-1.57) [ASD vs other DD, men] |

## 1.2 Immunological

Table 23: Prevalence of immune issues in those with ASD.

| Source | Sample | Prevalence | Other Notes |
|---|---|---|---|
| Zerbo et al. (2015) [132] | n=5565 (ASD), n=27825 (non-ASD); Age: 3-26; Kaiser Permanente Northern California; ≥12 months; 1995–2006 | Any immune condition: 28.61% (ASD), 27.57% (non-ASD) | OR (95% CI): 1.05 (0.98 - 1.11) |
| Schott et al. (2022) [101] | n=31101 (ASD, no ID), n=52049 (ASD+ID), n=563558 (ID, no ASD), n=372807 (any mental health condition, no ASD), n=683778 (no MHC, no ASD); Age: 20-64; Medicaid Analytic eXtract, 2008-12 | Immunocompromised: 0.43% (ASD, no ID), 0.41% (ASD+ID), 0.34% (ID, no ASD), 0.49% (MHC, no ASD), 0.14% (no MHC, no ASD) | OR: 1.17 (99% CI: 0.88-1.54) (ASD, no ID), OR: 0.97 (99% CI: 0.76-1.22) (ASD+ID), OR: 0.86 (99% CI: 0.75-0.98) (ID, no ASD), OR: 2.00 (99% CI: 1.79-2.23) (MHC, no ASD) |

### 1.2.1 Autoimmune

Table 24: Prevalence of autoimmune issues in those with ASD.

| Source | Sample | Prevalence | Other Notes |
|---|---|---|---|
| Croen et al. (2015) [25] | n=1507 (ASD), n=15070 (age, sex matches controls); Adults; Kaiser Permanente Northern California; 2008-12 | Autoimmune: 13.87% (ASD), 10.78% (non-ASD) | Chi-square p value: <0.001 |
| Zerbo et al. (2015) [132] | n=5565 (ASD), n=27825 (non-ASD); Age: 3-26; Kaiser Permanente Northern California; ≥12 months; 1995–2006 | Autoimmune: 1.02% (ASD), 0.76% (non-ASD) | OR (95% CI): 1.36 (1.01 - 1.83) |
| Davignon, et al. (2018) [29] | n=4123 (ASD), n=20615 (non-ASD, non-ADHD); Age: 14-25; Kaiser Permanente Northern California, 2013-15 | Autoimmune: 3.6% (ASD), 2.2% (non-ASD) | aOR (95% CI): 1.57 (1.30-1.90) |
| Schott et al. (2022) [100] | n=155,617 (ASD), n=466,851 (non-ASD); Adults; Medicaid; 2008–12 | Autoimmune: 24.06% (ASD), 23.92% (non-ASD) | aOR: 0.93 (0.92 0.95) |
| Al-Ayadhi et al. (2025) [2] | n=363 [n=189 (ASD), n=174 (non-ASD)]; Online questionnaire (self or caregiver); Saudi Arabia; August 2022 - January 2023 | Autoimmune: 21.7% (ASD), 3.4% (non-ASD) | e |
| Micai et al. (2023) [79] | Meta-analysis of observational studies, N=6 [N=4 (clinical studies from community samples), N=2 (population/registry based)], n=8419 | Celiac Disease: Pooled prevalence: 4%, Range: 1% - 9% | |
| Ward et al. (2023) [127] | n=1129 (ASD), n=1176 (non-ASD); Adults; Online survey | Coeliac Disease: 2.13% (ASD), 0.85% (non-ASD) | p-value: 0.02 |

### 1.2.2 Allergy

Table 25: Prevalence of allergies in those with ASD.

| Source | Sample | Prevalence | Other Notes |
|---|---|---|---|
| Aldinger et al. (2015) [3] | n=728 (ASD, children), n=694 (parents), n=184 (non-ASD, siblings); Autism Genetic Resource Exchange | Allergies: 40.0% (ASD, children), 39.9% (parents), 30.4% (non-ASD, siblings) | |
| Croen et al. (2015) [25] | n=1507 (ASD), n=15070 (age, sex matches controls); Adults; Kaiser Permanente Northern California; 2008-12 | Allergy: 36.30% (ASD), 28.71% (non-ASD) | Chi-square p value: <0.001 |
| Zerbo et al. (2015) [132] | n=5565 (ASD), n=27825 (non-ASD); Age: 3-26; Kaiser Permanente Northern California; ≥12 months; 1995–2006 | Any allergy: 20.63% (ASD), 17.66% (non-ASD) | OR (95% CI): 1.22 (1.13 - 1.31) |
| Dizitzer et al. (2020) [33] | n=459 (ASD), n=2285 (non-ASD); Children, southern Israel, Negev Autism Center database, born 2009-16, controls age, sex, ethnicity matched | Allergy or hypersensitivity: 15.7% (ASD), 10.7% (non-ASD) | OR: 1.56 (95% CI: 1.17-2.07), p: 0.002 |
| Schott et al. (2022) [100] | n=155,617 (ASD), n=466,851 (non-ASD); Adults; Medicaid; 2008–12 | Allergy: 36.88% (ASD), 29.70% (non-ASD) | aOR: 1.16 (1.14 1.17) |
| Salehi et al. (2025) [98] | n=2568 (ASD); Age: 3-17; The National Survey of Children's Health; 20–2021, USA - survey | Allergies: 32.4% | |
| Kennedy et al. (2024) [62] | n=246 (ASD); Connecticut Children's Medical Center ASD Assessment Program electronic records; 2018-19 | Allergies/sinus: 19.9% | |
| Al-Ayadhi et al. (2025) [2] | n=363 [n=189 (ASD), n=174 (non-ASD)]; Online questionnaire (self or caregiver); Saudi Arabia; August 2022 - January 2023 | No known allergy: 37.6% (ASD), 62.6% (non-ASD) | p: 0.000 |

**Food Allergy**

Table 26: Prevalence of food allergies in those with ASD.

| Source | Sample | Prevalence | Other Notes |
|---|---|---|---|
| Miyazaki et al. (2015) [81] | Meta-analysis, N=3 | Food allergy: OR: 1.23 (95% CI: 0.76, 2.01) | |
| Zerbo et al. (2015) [132] | n=5565 (ASD), n=27825 (non-ASD); Age: 3-26; Kaiser Permanente Northern California; ≥12 months; 1995–2006 | Food allergy: 0.90% (ASD), 0.50% (non-ASD) | OR (95% CI): 1.62 (1.17 - 2.23) |
| Xu et al. (2018) [131] | n=1868 (ASD), n=197652 (non-ASD); Age 3-17; US National Health Interview Survey; 1997 to 2016 | Food allergy: 11.25% (ASD), 4.25% (non-ASD) | 2.29 (1.87-2.81) adjusted for age, sex, race/ethnicity, family highest education level, ratio of family income to federal poverty level, geographic region, and other allergic conditions |
| Wang et al. (2021) [125] | Meta-analysis, children, N=13, n=14,486 (ASD), n=459,016 (non-ASD) | Food allergy, pooled prevalence: 13% (ASD), 5% (non-ASD) | Pooled OR: FA in individuals with ASD: 2.45 (95% CI: 2.25–2.67) |
| Micai et al. (2023) [79] | Meta-analysis of observational studies, N=7 [N=4 (clinical studies from community samples), N=3 (population/registry based)], n=7094 | Food Intolerance: Pooled prevalence: 13%, Range: 3% - 29% | |
| Micai et al. (2023) [79] | Meta-analysis of observational studies, N=2 [N=2 (clinical studies from community samples), N=0 (population/registry based)], n=338 | Gluten Intolerance: Pooled prevalence: 1%, Range: 0% - 3% | |
| Saad et al. (2024) [97] | n=150 (ASD), n=100 (non-ASD), Egyptian children | Food Allergy: 10% (ASD), 9% (non-ASD) | p-value: 0.12 |
| Al-Ayadhi et al. (2025) [2] | n=363 [n=189 (ASD), n=174 (non-ASD)]; Online questionnaire (self or caregiver); Saudi Arabia; August 2022 - January 2023 | Food allergy: 22.2% (ASD), 10.3% (non-ASD) | |

Table 27: Prevalence of ASD in those with food allergies.

| Source | Sample | Prevalence | Other Notes |
|---|---|---|---|
| Wang et al. (2021) [125] | Meta-analysis, N=5 | ASD: 3.5% (food allergy), 1.6% (no food allergy) | Pooled OR: ASD: 1.95 (95% CI: 1.73–2.19) [Food allergy] |
| Li et al. (2021) [72] | Meta-analysis, N=12, n=434809; Children | | ASD: OR = 2.792, 95% CI: 2.081-3.746 (food hypersensitivity) |

**Other Allergies**

Table 28: Prevalence of other allergies in those with ASD.

| Source | Sample | Prevalence | Other Notes |
|---|---|---|---|
| Zerbo et al. (2015) [132] | n=5565 (ASD), n=27825 (non-ASD); Age: 3-26; Kaiser Permanente Northern California; ≥12 months; 1995–2006 | Rhinitis: 16.0% (ASD), 12.9% (non-ASD) | OR (95% CI): 1.30 (1.20 - 1.41) |
| Davignon, et al. (2018) [29] | n=4123 (ASD), n=20615 (non-ASD, non-ADHD); Age: 14-25; Kaiser Permanente Northern California, 2013-15 | Allergy rhinitis: 3.7% (ASD), 2.4% (non-ASD) | aOR (95% CI): 1.62 (1.35 - 1.96) |
| Saad et al. (2024) [97] | n=150 (ASD), n=100 (non-ASD), Egyptian children | Allergic rhinitis: 25% (ASD), 8% (non-ASD) | p value : $\leq$ 0.01 |
| Davignon, et al. (2018) [29] | n=4123 (ASD), n=20615 (non-ASD, non-ADHD); Age: 14-25; Kaiser Permanente Northern California, 2013-15 | Drug allergy: 2.0% (ASD), 1.1% (non-ASD) | aOR (95% CI): 1.69 (1.30 - 2.18) |
| Al-Ayadhi et al. (2025) [2] | n=363 [n=189 (ASD), n=174 (non-ASD)]; Online questionnaire (self or caregiver); Saudi Arabia; August 2022 - January 2023 | Skin allergy: 50.3% (ASD), 26.4% (non-ASD); Respiratory allergy: 14.8% (ASD), 7.5% (non-ASD); Medication allergy: 3.2% (ASD), 1.7% (non-ASD) | p: 0.000; p: 0.027; p:0.375 |
| Xu et al. (2018) [131] | n=1868 (ASD), n=197652 (non-ASD); Age 3-17; US National Health Interview Survey; 1997 to 2016 | Respiratory allergy: 18.73% (ASD), 12.08% (non-ASD); Skin allergy: 16.81% (ASD), 9.84% (non-ASD) | 1.28 (1.10-1.50); 1.50 (1.28-1.77) adjusted for age, sex, race/ethnicity, family highest education level, ratio of family income to federal poverty level, geographic region, and other allergic conditions |

## 1.3 Metabolic

Table 29: Prevalence of metabolic conditions in those with ASD, table 1.

| Source | Sample | Prevalence | Other Notes |
|---|---|---|---|
| Cawthorpe (2016) [17] | n = 763,449 (total); n=583 (ASD, female), n=1457 (ASD, male), n=414185 (non-ASD, female), n=348,506 (non-ASD, male); 1994-2009; Canada; ICD-9 | Endocrine, nutritional, metabolic disorders, and immunity disorders: 30.98% (ASD), 36.78% (non-ASD) | OR: 1.47 (95% CI: 1.25-1.73) [Female], OR: 0.63 (95% CI: 0.56-0.71) [Male] |
| Steinhausen et al. (2024) [108] | n=12063 (ASD), n=41251 (non-ASD); Danish register; birth cohort 1984-95; ICD-8 and ICD-10 | | Endocrine, nutritional, and metabolic diseases (HR, 95% CI): 2.08 (1.83–2.36) (age: 0-6), 2.63 (2.38–2.90) (age: 7-17), 1.74 (1.58–1.91) (age: 18-24), 1.25 (1.10–1.44) (age: 25+) [ASD vs ref, total]; 1.86 (1.58–2.19) (age: 0-6), 2.45 (2.15–2.80) (age: 7-17), 2.55 (2.17–3.00) (age: 18-24), 2.51 (2.00–3.16) (age: 25+) [Males]; 2.48 (2.03–3.03) (age: 0-6), 2.87 (2.48–3.32) (age: 7-17), 1.42 (1.26–1.61) (age: 18-24), 0.90 (0.75–1.08) (age: 25+) [Females]; 1.39 (1.13–1.72) (age: 0-6), 1.65 (1.42–1.93) (age: 7-17), 3.21 (2.73–3.77) (age: 18-24), 3.47 (2.71–4.45) (age: 25+) [Female vs Male, ASD]; 1.04 (0.90–1.21) (age: 0-6), 1.41 (1.25–1.60) (age: 7-17), 5.76 (5.08–6.53) (age: 18-24), 9.71 (8.33–11.3) (age: 25+) [Female vs Male, non-ASD] |
| Davignon, et al. (2018) [29] | n=4123 (ASD), n=20615 (non-ASD, non-ADHD); Age: 14-25; Kaiser Permanente Northern California, 2013-15 | Metabolic (any): 3.5% (ASD), 1.0% (non-ASD); Lipoid metabolism: 2.4% (ASD), 0.5% (non-ASD) | aOR (95% CI): 3.71 (2.97-4.64); 5.35 (4.02-7.13) |

Table 30: Prevalence of metabolic conditions in those with ASD, table 2.

| Source | Sample | Prevalence | Other Notes |
|---|---|---|---|
| Angell et al. (2021) [4] | n=18355 (ASD, girls), n=65145 (ASD, boys); Age: 1-21; healthcare claims data; integrates ICD-9 and ICD-10 codes | Metabolic disorders: 8.49% (ASD, girls), 7.01% (ASD, boys) | aOR: 1.21 (95% CI: 1.14-1.29), p:<0.001 - adjusted for age, race, ethnicity, urbanicity, and insurance |
| Micai et al. (2023) [79] | Meta-analysis of observational studies, N=5 [N=4 (clinical studies from community samples), N=1 (population/registry based)], n=5014 | Metabolic Disorder: Pooled prevalence: 3%, Range: 1% - 5% | |

### 1.3.1 Mitochondrial

Table 31: Prevalence of mitochondrial conditions and biomarkers in those with ASD.

| Source | Sample | Prevalence | Other Notes |
|---|---|---|---|
| Goldenthal et al. (2015) [42] | n=92 (ASD), n=68 (non-ASD); Age: 3-22; ASD recruited from neurology clinic; St. Christopher's Hospital for Children and Drexel University College of Medicine. | RC-I defective: 12% (ASD); RC-IV defective: 27.2% (ASD); Combined RC-I + RC-IV defective: 3.2% (ASD); Hyperactive RC-I: 6.5% (ASD); Hyperactive RC-IV: 3.2% (ASD) | |
| Aoki and Cortese (2016) [5] | N=12, Systematic Review and Meta-analysis | (n datasets=, Pooled OR [95% CI]) rs2292813-G: n=10, 1.190 [1.052-1.346] (All), n=7, 1.216 [1.075–1.376] (Family based), n=3, 0.899 [0.594–1.360] (case-control); rs2056202-G: n=11, 1.206 [1.035–1.405] (All), n=6, 1.267 [1.041–1.542] (Family based), n=5, 1.071 [0.854–1.343] (Case-control) | p: 0.006, 0.002, 0.615, 0.016, 0.018, 0.552 |
| Wang et al. (2016) [126] | n=903 (ASD child, mother, sibling trios); National Database for Autism Research, CSHL only | 1+ high-confidence heteroplasmic or homoplasmic mutation with derived allele fraction (DAF) 5% [mutation]: 21.2% (ASD), 20.2% (non-ASD siblings); Nonsynonymous mutations: 5.6% (ASD), 3.1% (non-ASD sibling); CADD >15: 3.3% (ASD), 1.3% (non-ASD sibling); CADD >20: 2.4% (ASD), 1.0% (non-ASD sibling) | OR [95% CI], p: 1.87 [1.14–3.11], p: 0.0055 (Nonsynonymous mutations); 2.55 [1.26–5.51], p: 0.0036 (CADD >15); 2.48 [1.09–6.15], p: 0.014 (CADD >20) |

Table 32: Prevalence of mitochondrial conditions and biomarkers in those with ASD, table 2.

| Source | Sample | Prevalence | Other Notes |
|---|---|---|---|
| Delhey et al. (2017) [31] | n=76 (ASD); Age: 3-14; University of Arkansas for Medical Sciences | Enzyme activity outside normal range: Citrate Synthase: 24% (ASD), Complex I: 39% (ASD), Complex IV: 11% (ASD) | |
| Varga et al. (2018) [120] | n=60 (ASD) [n=6 (girls), n=54 (boys); Median Age=7], n=60 (non-ASD) [n=26 (women), n=34 (men), Median Age=28] | MtDNA deletions: 16.6% (ASD) | |
| Valiente-Pallejà (2018) [119] | n=122 (ASD+ID), n=115 (ID), n=112 (non-ASD controls); Sequenced: n=98 (ASD), n=95 (ID); Age: ≥18; Spain | 1+ putative pathogenic mtDNA mutation: 28.6% (ASD), 30.5% (ID) | |
| Frye et al. (2024) [38] | N=187, Systematic Review and Meta-analysis | Pooled prevalence: Lactate elevation: 17% (ASD, N=7); Pyruvate elevation: 41% (ASD, N=4); Lactate-to-Pyruvate Ratio elevation: 28% (ASD, N=1); Alanine elevation: 15% (ASD, N=3); Alanine-to-Lysine Ratio elevation: 8.2% (ASD, N=1); Acyl-Carnitine elevation: 19% (ASD, N=3); Creatine Kinase elevation: 9% (ASD, N=2) | [95% CI]: Lactate elevation: [8%, 29%] (ASD, N=7); Pyruvate elevation: [18%, 67%] (ASD, N=4); Lactate-to-Pyruvate Ratio elevation: [22%, 34%] (ASD, N=1); Alanine elevation: [0%, 42%] (ASD, N=3); Alanine-to-Lysine Ratio elevation: [3.4%, 14.5%] (ASD, N=1); Acyl-Carnitine elevation:[26%, 13%] (ASD, N=3); Creatine Kinase elevation: [4%, 15%] (ASD, N=2) |

### 1.3.2 Methylation and Oxidative Stress

Table 33: Prevalence of oxidative stress markers in those with ASD.

| Source | Sample | Prevalence | Other Notes |
|---|---|---|---|
| Ramaekers et al. (2020) [91] | n=38 (ASD), n=24 (non-ASD); Age: 3.6-18; NY, USA | 1+ elevated oxidative stress biomarker: 66% (ASD); Low vitamin A: 64% (ASD); Low vitamin D: 70% (ASD) | |
| Chen et al. (2021) [19] | N=87, n=9109 [n=4928 (ASD), n=4181 (non-ASD)]; Systematic review and Meta-analysis | Hedges g (95% CI), p: GSSG: 1.053 (0.865 to 1.241), <0.001; Homocysteine: 0.806 (0.431 to 1.181), <0.001; SAH: 0.730 (0.362 to 1.098), <0.001; Copper: 0.370 (0.154 to 0.586), 0.001; Nitric oxide: 3.118 (0.682 to 5.555), 0.012; MDA: 1.435 (0.622 to 2.248), <0.001; tGSH: -1.905 (-2.722 to -1.087), <0.001; GSH: -0.885 (-1.224 to -0.546), <0.001; GSH/GSSG: -1.991 (-2.792 to -1.189), <0.001; tGSH/GSSG: -1.822 (-2.279 to -1.365), <0.001; Methionine: -0.580 (-0.894 to -0.265), <0.001; Cysteine: -0.718 (-1.214 to -0.221), 0.005; SAM/SAH: -1.248 (-1.839 to -0.658), <0.001; Vitamin B9: -0.914 (-1.529 to -0.298), 0.004; Vitamin B12: -0.470 (-0.853 to -0.087), 0.016; Vitamin D: -0.874 (-1.218 to -0.529), <0.001; Vitamin E: -1.580 (-3.144 to -0.016), 0.048; Calcium: -1.138 (-2.007 to -0.269), 0.010 | |

**Cancer**

Table 34: Prevalence of having cancer in those with ASD, table 1.

| Source | Sample | Prevalence | Other Notes |
|---|---|---|---|
| Chiang et al. (2015) [21] | n=8438 (ASD); Age: $\leq$20; Cancer confirmed via histology; Taiwan National Health Insurance database, 1997-2011 | All cancers: n=20 (ASD), n=10.31 (exp), standardized incidence ration (SIR) 1.94(95% CI: 1.18-2.99) [total]; n=16 (ASD), n=8.21 (exp), (SIR) 1.95(95% CI: 1.11-3.16) [male], n=4 (ASD), n=2.10 (exp), (SIR) 1.91(95% CI: 0.52-4.88) [female]; Hematologic (all): SIR: 1.55(95% CI: 0.57-3.37) [total]; SIR: 1.51(95% CI: 0.49-3.52) [male]; SIR: 1.81(95% CI: 0.05-10.07) [female] Leukemia: SIR: 2.05(95% CI: 0.67-4.79) [total]; SIR: 1.91(95% CI: 0.52-4.89) [male]; SIR: 2.90(95% CI: 0.07-16.16) [female] Lymphoma: SIR: 0.71(95% CI: 0.02-3.93) [total]; SIR: 0.83(95% CI: 0.02-4.61) [male]; SIR: 0(95% CI: 0-17.71) [female] CNS: SIR: 1.77(95% CI: 0.36-5.17) [total]; SIR: 2.05(95% CI: 0.42-6.00) [male]; SIR: 0(95% CI: 0-15.80) [female] Eye: SIR: 9.44(95% CI: 0.24-52.29) [total]; SIR: 10.61(95% CI: 0.27-59.13) [male]; SIR: 0(95% CI: 0-315.08) [female] Head and neck: SIR: 1.77(95% CI: 0.04-9.85) [total]; SIR: 0(95% CI: 0-7.75) [male]; SIR: 11.17(95% CI: 0.28-62.26) [female] Bone and soft tissue: SIR: 2.09(95% CI: 0.25-7.56) [total]; SIR: 2.52(95% CI: 0.31-9.12) [male]; SIR: 0(95% CI: 0-22.55) [female] Genitourinary (all): SIR: 4.16(95% CI: 1.13-10.65) [total]; SIR: 0(95% CI: 0-19.43) [male]; SIR: 6.38(95% CI: 0.77-23.06) [female] Ovary: SIR: 9.22(95% CI: 1.12-33.29) [total]; NA [male]; SIR: 9.22(95% CI: 1.12-33.29) [female] Testis: SIR: 4.33(95% CI: 0.52-15.65) [total]; SIR: 4.33(95% CI: 0.52-15.65) [male]; NA [female] Digestive (all): SIR: 2.99(95% CI: 0.36-10.8) [total]; SIR: 3.67(95% CI: 0.44-13.25) [male]; SIR: 0(95% CI: 0-29.76) [female] Liver and biliary tract: SIR: 5.89(95% CI: 0.71-21.27) [total]; SIR: 6.67(95% CI: 0.81-24.09) [male]; SIR: 0(95% CI: 0-92.84) [female] Lung and mediastinum: SIR: 4.53(95% CI: 0.11-25.26) [total]; SIR: 5.15(95% CI: 0.13-28.68) [male]; SIR: 0(95% CI: 0-140.17) [female] | p:<0.05: genitourinary (total), ovary (total), all cancers (male), age 15-19 (male); p:<0.01: all cancers (total), age 15-19 (total) |

Table 35: Prevalence of having cancer in those with ASD, table 2.

| Source | Sample | Prevalence | Other Notes |
|---|---|---|---|
| Croen et al. (2015) [25] | n=1507 (ASD), n=15070 (age, sex matches controls); Adults; Kaiser Permanente Northern California; 2008-12 | Cancer: 1.73% (ASD), 2.21% (non-ASD) | Chi-square p value: 0.22 |
| Darbro et al. (2016) [28] | University of Iowa Hospitals and Clinics records, 2009-15, n=1837 (ASD), n=9336 (non-ASD) | Neoplasm: 1.3% (ASD), 3.9% (non-ASD) | p-value: <0.001 |
| Cawthorpe (2016) [17] | n = 763,449 (total); n=583 (ASD, female), n=1457 (ASD, male), n=414185 (non-ASD, female), n=348,506 (non-ASD, male); 1994-2009; Canada; ICD-9 | Neoplasms: 22.50% (ASD), 33.95% (non-ASD) | Female: OR: 0.86 (95% CI: 0.73-1.02), Male: OR: 0.55 (95% CI: 0.48-0.63) |
| Cawthorpe (2018) [18] | n=609 (ASD, male), n=111 (ASD, female), n=28588 (non-ASD, male), n=17,787 (non-ASD, female); Regional cohort - <3 between April 1, 1993 and December 31, 1994 and had a diagnosis by that time (any kind); 16 year study, max age: 18; ICD-9 | | Neoplasm: OR: 1.29, p: <0.05 (males), OR: 1.66, p:<0.05 (females) |
| Vohra et al. (2017) [123] | n=1772 (ASD), n=5316 (non-ASD); Age: 22-64; three state Medicaid Analytic eXtract; 2000-08 | Cancer: 1.2% (ASD), 2.8% (non-ASD) | aOR (95% CI): 0.41 (0.26, 0.66), p:<0.001 |
| Bishop-Fitzpatrick et al. (2018) [10] | n=91 (ASD), n=6186 (sex, birth year matched controls); Age: 0-89; EHR from Marshfield clinic - decedents - Wisconsin | Lymphoma: 1.1% (ASD), 3.9% (non-ASD); Metastatic cancer: 4.4% (ASD), 19.4% (non-ASD); Solid tumor without metastasis: 15.4% (ASD), 18.0% (non-ASD) | aOR: 0.27 (adjusted for age and sex), p: 0.19; aOR: 0.19, p:<0.001; aOR: 0.88, p: 0.65 |

Table 36: Prevalence of having cancer in those with ASD, table 3.

| Source | Sample | Prevalence | Other Notes |
|---|---|---|---|
| Hand et al. (2020) [46] | n=4685 (ASD), n=46850 (non-ASD); Age: ≥65; Medicare; 2016-17 | Cancer: 30.7% (ASD), 26.3% (non-ASD) | aOR: 1.2 (1.2-1.3) |
| Weir et al. (2021) [128] | n=1156 (ASD), n=1212 (non-ASD); Age: >16; online recruitment - survey; 2018-19 | Cancer: 4% (ASD, female), 5% (ASD, male), 4.5% (non-ASD, female), 5.5% (non-ASD, male) | OR: 1.068 (95% CI: 0.605-1.872) [female], OR: 0.739 (95% CI: 0.367-1.473) [male] - controlled for lifestyle factors - BMI, alcohol use, smoking |
| Schott et al. (2022) [100] | n=155,617 (ASD), n=466,851 (non-ASD); Adults; Medicaid; 2008–12 | Cancer: 4.53% (ASD), 4.74% (non-ASD) | aOR: 0.94 (0.90 0.97) |
| Liu et al. (2022) [73] | n=40,334 (ASD), n=2,278,100 (non-ASD); Age: <30; Swedish National Patient Register | Cancer: 0.51% (ASD), 0.35% (control) | OR 1.3, (95% CI: 1.2-1.5) |
| Schott et al. (2022) [101] | n=31101 (ASD, no ID), n=52049 (ASD+ID), n=563558 (ID, no ASD), n=372807 (any mental health condition, no ASD), n=683778 (no MHC, no ASD); Age: 20-64; Medicaid Analytic eXtract, 2008-12 | Cancer: 4.87% (ASD, no ID), 5.82% (ASD+ID), 7.95% (ID, no ASD), 8.09% (MHC, no ASD), 2.63% (no MHC, no ASD) | OR: 1.54 (99% CI: 1.42-1.67) (ASD, no ID), OR: 1.59 (99% CI 1.49-1.69) (ASD+ID), OR: 1.44 (99% CI 1.39-1.49) (ID, no ASD), OR: 2.03(99% CI 1.97-2.08) (MHC, no ASD). All p:<0.01 |

Table 37: Prevalence of having cancer in those with ASD, table 4.

| Source | Sample | Prevalence | Other Notes |
|---|---|---|---|
| Tint et al. (2023) [113] | n=3220 (ASD, women), n=12920 (no DD, women), n=3217 (other DD, women), n=7426 (ASD, men), n=29687 (no DD, men), n=7398 (other DD, men); Canada; administrative and social services data - ICES | Cancer: 2.6% (ASD, women), 2.1% (other DD, women), 2.0% (no DD, women), 1.1% (ASD, men), 0.7% (no DD, men), 1.4% (other DD, men) | aRR: 1.30 (95% 1.02-1.65) [ASD vs no DD, women], aRR: 1.27 (95% 0.93-1.74) [ASD vs other DD, women], aRR: 1.55 (95% 1.22-1.97) [ASD vs no DD, men], aRR: 0.97 (95% 0.59-1.05) [ASD vs other DD, men] |
| Steinhausen et al. (2024) [108] | n=12063 (ASD), n=41251 (non-ASD); Danish register; birth cohort 1984-95; ICD-8 and ICD-10 | | Neoplasm (HR, 95% CI): 0.95 (0.70-1.28) (age:0-19), 0.92 (0.66-1.27) (age: 20+) [ASD vs ref, total]; 0.79 (0.55–1.14) (age:0-19), 0.89 (0.58–1.36) (age: 20+)[Males]; 1.44 (0.87–2.37) (age:0-19), 0.96 (0.58–1.59) (age: 20+) [Females]; 1.37 (0.79–2.37) (age:0-19), 1.68 (0.92–3.06) (age: 20+) [Female vs Male, ASD]; 0.75 (0.56–1.00) (age:0-19), 1.55 (1.19–2.03) (age: 20+) [Female vs Male, non-ASD] |

**Cardiovascular**

Table 38: Prevalence of cardiovascular problems in those with ASD, table 1.

| Source | Sample | Prevalence | Other Notes |
|---|---|---|---|
| Croen et al. (2015) [25] | n=1507 (ASD), n=15070 (age, sex matches controls); Adults; Kaiser Permanente Northern California; 2008-12 | Cardiovascular disease, any: 36.96% (ASD), 23.03% (non-ASD) | Chi-square p value: $<0.001$ |
| Croen et al. (2015) [25] | n=1507 (ASD), n=15070 (age, sex matches controls); Adults; Kaiser Permanente Northern California; 2008-12 | Any hospitalized CVD; 1.73% (ASD), 1.00% (non-ASD) | Chi-square p value: 0.008 |
| Vohra et al. (2017) [123] | n=1772 (ASD), n=5316 (non-ASD); Age: 22-64; three state Medicaid Analytic eXtract; 2000-08 | Cardiovascular or cerebrovascular: 13.7% (ASD), 16.9% (non-ASD) | aOR (95% CI): 0.77 (0.66, 0.90), $0.001 \leq p < 0.01$; |
| Davignon, et al. (2018) [29] | n=4123 (ASD), n=20615 (non-ASD, non-ADHD); Age: 14-25; Kaiser Permanente Northern California, 2013-15 | Cardiovascular: 6.0% (ASD), 2.6% (non-ASD) | aOR (95% CI): 2.32 (1.98 - 2.71) |
| Hand et al. (2020) [46] | n=4685 (ASD), n=46850 (non-ASD); Age: $\geq$65; Medicare; 2016-17 | Heart disease: 54.2% (ASD), 37.1% (non-ASD) | aOR: 2.1 (2.0-2.3) |
| Hand et al. (2020) [46] | n=4685 (ASD), n=46850 (non-ASD); Age: $\geq$65; Medicare; 2016-17 | Cerebrovascular disease: 12.1% (ASD), 8.3% (non-ASD) | aOR: 1.6 (1.4-1.7) |
| Weir et al. (2021) [128] | n=1156 (ASD), n=1212 (non-ASD); Age: >16; online recruitment - survey; 2018-19 | Cardiovascular, estimated: 19% (ASD, female), 28% (ASD, male), 15% (non-ASD, female), 21% (non-ASD, male) | OR: 1.383 (95% CI: 1.033-1.854), p:<0.05 [female], OR: 1.542 (95% CI: 1.056-2.262) [male] - controlled for lifestyle factors - BMI, alcohol use, smoking |

Table 39: Prevalence of cardiovascular problems in those with ASD, table 2.

| Source | Sample | Prevalence | Other Notes |
|---|---|---|---|
| Schott et al. (2022) [100] | n=155,617 (ASD), n=466,851 (non-ASD); Adults; Medicaid; 2008–12 | Cardiovascular diseases: 40.02% (ASD), 40.08% (non-ASD) | aOR: 1.04 (1.02 1.06) |
| Schott et al. (2022) [100] | n=155,617 (ASD), n=466,851 (non-ASD); Adults; Medicaid; 2008–12 | Any hospitalized CVD: 31.49% (ASD), 34.08% (non-ASD) | aOR: 0.92 (0.90 0.94) |
| Schott et al. (2022) [101] | n=31101 (ASD, no ID), n=52049 (ASD+ID), n=563558 (ID, no ASD), n=372807 (any mental health condition, no ASD), n=683778 (no MHC, no ASD); Age: 20-64; Medicaid Analytic eXtract, 2008-12 | Serious heart conditions: 2.75% (ASD, no ID), 3.15% (ASD+ID), 6.17% (ID, no ASD), 6.41% (MHC, no ASD), 1.82% (no MHC, no ASD) | OR: 0.98(99% CI: 0.89-1.09) (ASD, no ID), OR: 0.80(99% CI: 0.74-0.87) (ASD+ID), OR: 1.30(99% CI: 1.25-1.34) (ID, no ASD), OR: 1.95(99% CI: 1.89-2.02) (MHC, no ASD) |
| Smith DaWalt et al. (2021) [30] | n=2187 (ASD) [n=1716 (male), n=471 (female)], n=21870 (non-ASD, age and sex matched) [n=17160 (male), n=4710 (female)]; EHRs - Marshfield Clinic - Wisconsin; ICD-9 codes | Cardiovascular disease: 0.28 (female, ASD), 0.25 (male,ASD), 0.13 (female, control), 0.10 (male, control) [predicted probability] | OR: 2.89 (95% CI: 2.55-3.28), p:<0.001 [ASD status], OR: 0.94 (95% CI: 0.72-1.21) [ASD X Sex] |
| Liu et al. (2023) [74] | n=5291 (ASD), n=4195596 (non-ASD); Age: >45; Swedish nationwide Total Population Register and National Patient Register; born 1932-67; cumulative incidence uses 1:10 sex and birth year matched cohort | Heart failure: 2.9% (ASD), 3.7% (non-ASD) [prevalence over follow up period]; 4.4% (ASD), 3.6% (non-ASD) [estimated cumulative incidence by age 70] | aOR: 1.89 (95% CI: 1.61-2.22), p:<0.0001 (ASD vs non-ASD); aOR: 1.91 (95% CI: 1.59-2.29), p:<0.0001 (ASD without ID vs non-ASD); aOR: 1.84 (95% CI: 1.34-2.53), p: 0.0002 (ASD+ID vs non-ASD); p: 0.85 (ASD without ID vs ASD+ID); aOR: 1.52 (95% CI: 1.25-1.85), p:<0.0001 (men with ASD vs men without ASD); aOR: 3.30 (95% CI: 2.52-4.32), p:<0.0001 (women with ASD vs women without ASD); p:<0.0001 (men with ASD vs women with ASD) [AORs from cumulative incidence; adjusted for sex and birth year] |

Table 40: Prevalence of cardiovascular problems in those with ASD, table 3.

| Source | Sample | Prevalence | Other Notes |
|---|---|---|---|
| Liu et al. (2023) [74] | n=5291 (ASD), n=4195596 (non-ASD); Age: >45; Swedish nationwide Total Population Register and National Patient Register; born 1932-67; cumulative incidence uses 1:10 sex and birth year matched cohort | Cerebrovascular disease: 4.5% (ASD), 6.2% (non-ASD) [prevalence over follow up period]; 8.4% (ASD), 8.2% (non-ASD) [estimated cumulative incidence by age 70] | aOR: 1.55 (95% CI: 1.37-1.77), p:<0.0001 (ASD vs non-ASD); aOR: 1.50 (95% CI: 1.29-1.74), p:<0.0001 (ASD without ID vs non-ASD); aOR: 1.73 (95% CI: 1.36-2.20), p:<0.0001 (ASD+ID vs non-ASD); p: 0.33 (ASD without ID vs ASD+ID); aOR: 1.45 (95% CI: 1.24-1.69), p:<0.0001 (men with ASD vs men without ASD); aOR: 1.86 (95% CI: 1.49-2.32), p:<0.0001 (women with ASD vs women without ASD); p: 0.065 (men with ASD vs women with ASD) [all aORs based on cumulative incidence; adjusted for sex and birth year] |
| Weir et al. (2021) [128] | n=1156 (ASD), n=1212 (non-ASD); Age: >16; online recruitment - survey; 2018-19 | Heart disease, estimated: NA% (ASD, female), 4% (ASD, male), NA% (non-ASD, female), 3% (non-ASD, male) | OR: NA [female], OR: 1.121 (95% CI: 0.501-2.562), p: ns [male] - controlled for lifestyle factors - BMI, alcohol use, smoking |
| Longo et al. (2024) [75] | n=30258 (ASD), n=60516 (non-ASD); Age: 18-64; IBM Market Scan Commercial Claims and Encounters Database; 2019-20 | Heart disease: 15.6% (ASD), 10.8% (non-ASD) | aOR: 1.5 (95% CI: 1.5, 1.6) |

Table 41: Prevalence of cardiovascular problems in those with ASD, table 4.

| Source | Sample | Prevalence | Other Notes |
|---|---|---|---|
| Longo et al. (2024) [75] | n=30258 (ASD), n=60516 (non-ASD); Age: 18-64; IBM Market Scan Commercial Claims and Encounters Database; 2019-20 | Cerebrovascular: 1.1% (ASD), 0.4% (non-ASD) | aOR: 2.4 (95% CI: 2.1, 2.9) |
| Hunt et al. (2024) [53] | n=813 (ASD), n=2781 (non-ASD); Age: 6-97; National Center for Mental Health database and volunteers; Wales and UK | Heart disease: 1.5% (ASD), 4.0% (non-ASD) | p:<0.001 (ASD vs non-ASD); ASD vs non-ASD: aOR: 0.57 (95% 0.17-1.94), p: 0.367 (adjusted for age, gender, antipsychotic use, mood stabilizer, smoking status, COPD, chronic obstructive pulmonary disease); ASD (no ID) vs non-ASD: aOR: 0.60 (95% 0.17-2.04), p: 0.409 (adjusted for COPD, chronic obstructive pulmonary disease) ASD+ID vs non-ASD: aOR: 0.0 (95% 0.0-0.0), p: 0.997 (adjusted for COPD, chronic obstructive pulmonary disease) |
| Salehi et al. (2025) [98] | n=2568 (ASD); Age: 3-17; The National Survey of Children's Health; USA; 2020-21 | Heart Disorder: 3.5% | |

*Congenital Heart Disease*

Table 42: Prevalence of congenital heart disease in those with ASD.

| Source | Sample | Prevalence | Other Notes |
|---|---|---|---|
| Sigmon et al. (2019) [105] | n=8760 (ASD), n=26280 (non-ASD) ; Children; USA; Military Health System; 2001-13 | 4.6% (ASD), 2.5% (non-ASD) | p-value: <0.001, OR: 1.85 (95% CI 1.63–2.10; n = 1063) |

Table 43: Prevalence of ASD in those with congenital heart disease.

| Source | Sample | Prevalence | Other Notes |
|---|---|---|---|
| Tsao et al. (2017)[117] | n=3552(CHD), n=14,208 (non-CHD); Age: <18; Taiwan; National Health Insurance | 0.99/1000 person-year (CHD), 0.20/1000 person-year | p-vale: <0.001, CHD vs control - adjusted for co-occurring conditions (aHR 1.97, 95% CI 1.11–3.52) |
| Sigmon et al. (2019) [105] | n=8760 (ASD), n=26280 (non-ASD) ; Children; USA; Military Health System; 2001-13 | | ASD: OR (95% CI): 1.32, (1.10–1.59) [CHD] |
| Jenabi et al. (2022) [56] | N=6; Meta-analysis | ASD risk: OR=1.35 (95% CI: 1.17, 1.52; $I^2$=88.1% | |
| Gu et al. (2023) [44] | Review, N=7, n=3984 (CHD), n=9829 (ASD), n=236,798 (controls); Mixed studies examining ASD prevalence in ASD and reverse | | ASD: OR: 1.99, 95% CI: 1.77–2.24 [CHD vs non-CHD] |

*Hypertension*

Table 44: Prevalence of hypertension in those with ASD, table 1.

| Source | Sample | Prevalence | Other Notes |
|---|---|---|---|
| Croen et al. (2015) [25] | n=1507 (ASD), n=15070 (age, sex matches controls); Adults; Kaiser Permanente Northern California; 2008-12 | 25.61% (ASD), 15.63% (non-ASD) | Chi-square p value: $<0.001$ |
| Shedlock et al. (2016) [103] | n=48762 (ASD), n=243810 (non-ASD); children; military healthcare system database, 2000-13 | Hypertension: 1.0% (ASD), 0.5% (non-ASD) | (OR: 2.04, 95% CI 1.84-2.27), $p<0.001$ |
| Weiss, et al. (2017) [129] | n=5085 (ASD), n=393,263 (no developmental disorder); Age: 18-24; Administrative health data, Canada | 1.67% (ASD), 0.83% (non-ASD) | aOR: 2.03 (1.63-2.52) |
| Bishop-Fitzpatrick et al. (2018) [10] | n=91 (ASD), n=6186 (sex, birth year matched controls); Age: 0-89; EHR from Marshfield clinic - decedents - Wisconsin | Hypertension: 38.5% (ASD), 51.9% (non-ASD) | aOR: 0.60 (adjusted for age and sex), p: 0.02 |
| Hand et al. (2020) [46] | n=4685 (ASD), n=46850 (non-ASD); Age: $\geq$65; Medicare; 2016-17 | Hypertension: 66.5% (ASD), 51.2% (non-ASD) | aOR: 2.0 (1.9-2.2) |
| Tint et al. (2021) [112] | n=6870 (ASD), n=2686160 (non-ASD); Age: 15-44; Administrative health data, Canada, 2017-18 | 2.1% (ASD), 2.8% (non-ASD) | Standard difference: 0.05 |
| Schott et al. (2022) [100] | n=155,617 (ASD), n=466,851 (non-ASD); Adults; Medicaid; 2008–12 | 14.31% (ASD), 16.69% (non-ASD) | aOR: 0.86 (0.84 0.88) |
| Chieh et al. (2022) [22] | N=6, Review | Hypertension: N=4 studies found increased odds or prevalence, N=2 found no significant difference | |

Table 45: Prevalence of hypertension in those with ASD, table 2.

| Source | Sample | Prevalence | Other Notes |
|---|---|---|---|
| Liu et al. (2023) [74] | n=5291 (ASD), n=4195596 (non-ASD); Age: >45; Swedish nationwide Total Population Register and National Patient Register; born 1932-67; cumulative incidence uses 1:10 sex and birth year matched cohort | Hypertension and related disease: 10.2% (ASD), 18.5% (non-ASD) [prevalence over follow up period]; 19.1% (ASD), 29.0% (non-ASD) [estimated cumulative incidence by age 70] | aOR: 0.96 (95% CI: 0.88-1.05), p: 0.35 (ASD vs non-ASD); aOR: 1.11 (95% CI: 1.01-1.22), p: 0.024 (ASD without ID vs non-ASD); aOR: 0.52 (95% CI: 0.41-0.65), p:<0.0001 (ASD+ID vs non-ASD); p:<0.0001 (ASD without ID vs ASD+ID); aOR: 0.93 (95% CI: 0.84-1.03), p: 0.16 (men with ASD vs men without ASD); aOR: 1.02 (95% CI: 0.88-1.19), p: 0.75 (women with ASD vs women without ASD); p: 0.29 (men with ASD vs women with ASD) [all aORs based on cumulative incidence; adjusted for sex and birth year] |
| Tint et al. (2023) [113] | n=3220 (ASD, women), n=12920 (no DD, women), n=3217 (other DD, women), n=7426 (ASD, men), n=29687 (no DD, men), n=7398 (other DD, men); Canada; administrative and social services data - ICES | Hypertension: 13.1% (ASD, women), 9.7% (other DD, women), 13.1% (no DD, women), 7.4% (ASD, men), 6.2% (no DD, men), 7.6% (other DD, men) | aRR: 1.35 (95% 1.23-1.48) [ASD vs no DD, women], aRR: 1.03 (95% 0.92-1.15) [ASD vs other DD, women], aRR: 1.20 (95% 1.10-1.31) [ASD vs no DD, men], aRR: 0.98 (95% 0.88-1.09) [ASD vs other DD, men] |
| Longo et al. (2024) [75] | n=30258 (ASD), n=60516 (non-ASD); Age: 18-64; IBM Market Scan Commercial Claims and Encounters Database; 2019-20 | Hypertension: 9.1% (ASD), 6.5% (non-ASD) | aOR: 1.6 (95% CI: 1.5, 1.7) |

Table 46: Prevalence of hypertension in those with ASD, table 3.

| Source | Sample | Prevalence | Other Notes |
|---|---|---|---|
| Hunt et al. (2024) [53] | n=813 (ASD), n=2781 (non-ASD); Age: 6-97; National Center for Mental Health database and volunteers; Wales and UK | Hypertension: 12.0% (ASD), 16.7% (non-ASD) | p:<0.001 (ASD vs non-ASD); ASD vs non-ASD: aOR: 2.11 (95% 1.44-3.09), p:<0.001 (adjusted for age, gender, antipsychotic use, mood stabilizer, smoking status, COPD, chronic obstructive pulmonary disease); ASD (no ID) vs non-ASD: aOR: 2.11 (95% 1.44-3.11), p:<0.001 (adjusted for COPD, chronic obstructive pulmonary disease) ASD+ID vs non-ASD: aOR: 2.13 (95% 0.81-5.60), p: 0.124 (adjusted for COPD, chronic obstructive pulmonary disease) |

**Diabetes**

Table 47: Prevalence of diabetes in those with ASD, table 1.

| Source | Sample | Prevalence | Other Notes |
|---|---|---|---|
| Croen et al. (2015) [25] | n=1507 (ASD), n=15070 (age, sex matches controls); Adults; Kaiser Permanente Northern California; 2008-12 | Diabetes: 7.56% (ASD), 4.33% (non-ASD) | Chi-square p value: <0.001 |
| Zerbo et al. (2015) [132] | n=5565 (ASD), n=27825 (non-ASD); Age: 3-26; Kaiser Permanente Northern California; ≥12 months; 1995–2006 | Type 1 diabetes: 0.22% (ASD), 0.19% (non-ASD) | OR (95% CI): 1.15 (0.62 - 2.17) |
| Shedlock et al. (2016) [103] | n=48762 (ASD), n=243810 (non-ASD); children; military healthcare system database, 2000-13 | Type 2 diabetes: 1.1% (ASD), 0.4% (non-ASD) | (OR: 2.68, 95% CI 2.41 - 2.99), p<0.001 |
| Weiss, et al. (2017) [129] | n=5085 (ASD), n=393,263 (no developmental disorder); Age: 18-24; Administrative health data, Canada | Diabetes: 1.67% (ASD), 0.65% (non-ASD) | aOR: 2.78 (2.23-3.45) |
| Vohra et al. (2017) [123] | n=1772 (ASD), n=5316 (non-ASD); Age: 22-64; three state Medicaid Analytic eXtract; 2000-08 | Diabetes: 3.6% (ASD), 4.7% (non-ASD) | aOR (95% CI): 0.74 (0.56, 0.99), p: 0.01 ≤ p <0.05 |
| Davignon, et al. (2018) [29] | Medical records - Kaiser Permanente Northern California, 2013-2015, age 14-25, n=4123 (ASD), n=20615 (non-ASD, non-ADHD, non-DM) | 0.6% (ASD), N/A% (non-ASD) | |
| Bishop-Fitzpatrick et al. (2018) [10] | n=91 (ASD), n=6186 (sex, birth year matched controls); Age: 0-89; EHR from Marshfield clinic - decedents - Wisconsin | Diabetes, complicated: 7.7% (ASD), 12.0% (non-ASD); Diabetes, uncomplicated: 8.8% (ASD), 14.2% (non-ASD) | aOR: 0.63 (adjusted for age and sex), p: 0.24 ; aOR: 0.60, p: 0.17 |

Table 48: Prevalence of diabetes in those with ASD, table 2.

| Source | Sample | Prevalence | Other Notes |
|---|---|---|---|
| Hand et al. (2020) [46] | n=4685 (ASD), n=46850 (non-ASD); Age: ≥65; Medicare; 2016-17 | Diabetes; 36.6% (ASD), 27.4% (non-ASD) | aOR: 1.6 (1.5-1.7) |
| Tromans et al. (2020) [115] | Review, N=19 | 0.22% - 0.79% (T1,ASD), 0.1% - 0.59% (T1,non-ASD); 0.5%-1.06% (T2,ASD); 0.66% - 13.6% (Diabetes, ASD) | |
| Tint et al. (2021) [112] | n=6870 (ASD), n=2686160 (non-ASD); Age: 15-44; Administrative health data, Canada, 2017-18 | Diabetes: 4.3% (ASD), 2.3% (non-ASD) | Standard difference: 0.11 |
| Brooks et al. (2021) [12] | n=1062 (ASD), n=79175 Age: 1-24; Canada; Electronic Medical Record Primary Care database | Diabetes: 0.6% (ASD), 0.4% (non-ASD) | Prevalence ratio: 1.29 (95% CI: 0.58, 2.87) - adjusted for age, sex, neighborhood income, geographic location |
| Weir et al. (2021) [128] | n=1156 (ASD), n=1212 (non-ASD); Age: >16; online recruitment - survey; 2018-19 | Diabetes, estimated: 10% (ASD, female), 10% (ASD, male), 6% (non-ASD, female), 5% (non-ASD, male) | OR: 1.692 (95% CI: 1.109-2.599), p:<0.05 [female], OR: 1.509 (95% CI: 0.822-2.830), p:ns [male] - controlled for lifestyle factors - BMI, alcohol use, smoking |
| Weir et al. (2021) [128] | n=1156 (ASD), n=1212 (non-ASD); Age: >16; online recruitment - survey; 2018-19 | Type II diabetes, estimated: 3% (ASD, female), 5% (ASD, male), 4% (non-ASD, female), 2% (non-ASD, male) | OR: 1.028 (95% CI: 0.510-2.055), p: ns [female], OR: 1.799 (95% CI: 0.688-5.266), p: ns [male] - controlled for lifestyle factors - BMI, alcohol use, smoking |

Table 49: Prevalence of diabetes in those with ASD, table 3.

| Source | Sample | Prevalence | Other Notes |
|---|---|---|---|
| Weir et al. (2021) [128] | n=1156 (ASD), n=1212 (non-ASD); Age: >16; online recruitment - survey; 2018-19 | Prediabetes, estimated: 5% (ASD, female), 3% (ASD, male), 2% (non-ASD, female), 4% (non-ASD, male) | OR: 4.379 (95% CI: 2.102-9.772), p:<0.001 [female], OR: 1.020 (95% CI: 0.419-2.474), p: ns [male] - controlled for lifestyle factors - BMI, alcohol use, smoking |
| Schott et al. (2022) [100] | n=155,617 (ASD), n=466,851 (non-ASD); Adults; Medicaid; 2008–12 | 8.61% (ASD), 9.40% (non-ASD) | aOR: 0.93 (0.91 0.96) |
| Chieh et al. (2022) [22] | N =8, Review | T2/Hyperglycemia: 6/8 studies found higher blood sugar, shorter onset, or higher prevalence. 1 found no significant difference in diabetes, 1 found lower fasting blood glucose | |
| Schott et al. (2022) [101] | n=31101 (ASD, no ID), n=52049 (ASD+ID), n=563558 (ID, no ASD), n=372807 (any mental health condition, no ASD), n=683778 (no MHC, no ASD); Age: 20-64; Medicaid Analytic eXtract, 2008-12 | Diabetes: 11.82% (ASD, no ID), 12.57% (ASD+ID), 16.69% (ID, no ASD), 17.78% (MHC, no ASD), 7.65% (no MHC, no ASD) | OR: 1.48(99% CI: 1.40-1.56) (ASD, no ID), OR: 1.26(99% CI: 1.21-1.32) (ASD+ID), OR: 1.25(99% CI: 1.23-1.28) (ID, no ASD), OR: 1.64(99% CI: 1.61-1.67) (MHC, no ASD). All p:<0.01 |

Table 50: Prevalence of diabetes in those with ASD, table 4.

| Source | Sample | Prevalence | Other Notes |
|---|---|---|---|
| Liu et al. (2023) [74] | n=5291 (ASD), n=4195596 (non-ASD); Age: >45; Swedish nationwide Total Population Register and National Patient Register; born 1932-67; cumulative incidence uses 1:10 sex and birth year matched cohort | Type 2 diabetes: 6.4% (ASD), 6.9% (non-ASD) [prevalence over follow up period]; 12.3% (ASD), 9.7% (non-ASD) [estimated cumulative incidence by age 70] | aOR: 1.65 (95% CI: 1.48-1.83), p:<0.0001 (ASD vs non-ASD); aOR: 1.74 (95% CI: 1.54-1.96), p:<0.0001 (ASD without ID vs non-ASD); aOR: 1.37 (95% CI: 1.09-1.73), p: 0.0082 (ASD+ID vs non-ASD); p: 0.078 (ASD without ID vs ASD+ID); aOR: 1.54 (95% CI: 1.36-1.75), p:<0.0001 (men with ASD vs men without ASD); aOR: 1.97 (95% CI: 1.62-2.39), p:<0.0001 (women with ASD vs women without ASD); p: 0.038 (men with ASD vs women with ASD) [AORs from cumulative incidence; adjusted for sex and birth year] |
| Liu et al. (2023) [74] | n=5291 (ASD), n=4195596 (non-ASD); Age: >45; Swedish nationwide Total Population Register and National Patient Register; born 1932-67; cumulative incidence uses 1:10 sex and birth year matched cohort | Glucose dysregulation: 0.5% (ASD), 0.3% (non-ASD) [prevalence over follow up period]; 1.0% (ASD), 0.4% (non-ASD) [estimated cumulative incidence by age 70] | aOR: 2.96 (95% CI: 2.04-4.29), p:<0.0001 (ASD vs non-ASD); aOR: 2.79 (95% CI: 1.80-4.33), p:<0.0001 (ASD without ID vs non-ASD); aOR: 3.51 (95% CI: 1.75-7.01), p: 0.0004 (ASD+ID vs non-ASD); p: 0.59 (ASD without ID vs ASD+ID); aOR: 2.75 (95% CI: 1.75-4.31), p:<0.0001 (men with ASD vs men without ASD); aOR: 3.65 (95% CI: 1.90-7.03), p: 0.0001 (women with ASD vs women without ASD); p: 0.48 (men with ASD vs women with ASD) [AORs from cumulative incidence; adjusted for sex and birth year] |

Table 51: Prevalence of diabetes in those with ASD, table 5.

| Source | Sample | Prevalence | Other Notes |
|---|---|---|---|
| Tint et al. (2023) [113] | n=3220 (ASD, women), n=12920 (no DD, women), n=3217 (other DD, women), n=7426 (ASD, men), n=29687 (no DD, men), n=7398 (other DD, men); Canada; administrative and social services data - ICES | Diabetes: 8.2% (ASD, women), 4.1% (other DD, women), 10.3% (no DD, women), 5.0% (ASD, men), 2.9% (no DD, men), 5.8% (other DD, men) | aRR: 2.00 (95% 1.74-2.30) [ASD vs no DD, women], aRR: 0.84 (95% 0.72-0.98) [ASD vs other DD, women], aRR: 1.69 (95% 1.50-1.89) [ASD vs no DD, men], aRR: 0.88 (95% 0.77-1.00) [ASD vs other DD, men] |
| Semovski et al. (2023) [102] | n=2020 (ASD); Age: 12-18; records and interviews; Canada | Diabetes mellitus: 1.3% (ASD) | |
| Longo et al. (2024) [75] | n=30258 (ASD), n=60516 (non-ASD); Age: 18-64; IBM Market Scan Commercial Claims and Encounters Database; 2019-20 | Diabetes: 9.7% (ASD), 5.1% (non-ASD) | aOR: 2.1 (95% CI: 2.0, 2.2 ) |
| Hunt et al. (2024) [53] | n=813 (ASD), n=2781 (non-ASD); Age: 6-97; National Center for Mental Health database and volunteers; Wales and UK | Type 1 Diabetes: 0.9% (ASD), 0.7% (non-ASD) | p: 0.656(ASD vs non-ASD); ASD vs non-ASD: aOR: 0.64 (95% 0.14-3.00), p: 0.573 (adjusted for age, gender, antipsychotic use, mood stabilizer, smoking status, COPD, chronic obstructive pulmonary disease); ASD (no ID) vs non-ASD: aOR: 0.59 (95% 0.12-2.91), p: 0.516 (adjusted for COPD, chronic obstructive pulmonary disease) ASD+ID vs non-ASD: aOR: 1.07 (95% 0.10-11.43), p: 0.956 (adjusted for COPD, chronic obstructive pulmonary disease) |

Table 52: Prevalence of diabetes in those with ASD, table 6.

| Source | Sample | Prevalence | Other Notes |
|---|---|---|---|
| Hunt et al. (2024) [53] | n=813 (ASD), n=2781 (non-ASD); Age: 6-97; National Center for Mental Health database and volunteers; Wales and UK | Type 2 Diabetes: 4.4% (ASD), 4.6% (non-ASD) | p: 0.775 (ASD vs non-ASD); ASD vs non-ASD: aOR: 2.32 (1.28-4.21), p: 0.573 (adjusted for age, gender, antipsychotic use, mood stabilizer, smoking status, COPD, chronic obstructive pulmonary disease); ASD (no ID) vs non-ASD: aOR: 2.27 (95% 1.24-4.15), p: 0.008 (adjusted for COPD, chronic obstructive pulmonary disease) ASD+ID vs non-ASD: aOR: 3.17 (95% 0.86-11.76), p: 0.084 (adjusted for COPD, chronic obstructive pulmonary disease) |
| Salehi et al. (2025) [98] | n=2568 (ASD); Age: 3-17; The National Survey of Children's Health; USA; 2020-21 | 0.9% | |

**Asthma**

Table 53: Prevalence of asthma in those with ASD, table 1.

| Source | Sample | Prevalence | Other Notes |
|---|---|---|---|
| Aldinger et al. (2015) [3] | n=728 (ASD, children), n=694 (parents), n=184 (non-ASD, siblings); Autism Genetic Resource Exchange | Asthma: 16.3% (ASD, children), 15.2% (parents), 18.2% (non-ASD, siblings) | |
| Croen et al. (2015) [25] | n=1507 (ASD), n=15070 (age, sex matches controls); Adults; Kaiser Permanente Northern California; 2008-12 | 12.54% (ASD), 10.27% (non-ASD) | Chi-square p value: 0.006 |
| Miyazaki et al. (2015) [81] | Meta-analysis, N=9 | | OR: 1.69 (95% CI: 1.11, 2.59) |
| Zerbo et al. (2015) [132] | n=5565 (ASD), n=27825 (non-ASD); Age: 3-26; Kaiser Permanente Northern California; $\geq$12 months; 1995–2006 | Asthma: 13.66% (ASD), 15.93% (non-ASD) | OR (95% CI): 0.83 (0.76 - 0.90) |
| Weiss, et al. (2017) [129] | n=5085 (ASD), n=393,263 (no developmental disorder); Age: 18-24; Administrative health data, Canada | 24.42% (ASD), 20.59% (non-ASD) | aOR: 1.13 (1.06-1.21) |
| Davignon, et al. (2018) [29] | n=4123 (ASD), n=20615 (non-ASD, non-ADHD); Age: 14-25; Kaiser Permanente Northern California, 2013-15 | 7.7% (ASD), 6.3% (non-ASD) | aOR (95% CI): 1.19 (1.04-1.35) |
| Hand et al. (2020) [46] | n=4685 (ASD), n=46850 (non-ASD); Age: $\geq$65; Medicare; 2016-17 | Asthma: 9.1% (ASD), 4.8% (non-ASD) | aOR: 2.0 (1.8-2.2) |
| Tint et al. (2021) [112] | n=6870 (ASD), n=2686160 (non-ASD); Age: 15-44; Administrative health data, Canada, 2017-18 | 26.2% (ASD), 17.5% (non-ASD) | Standard difference: 0.21 |

Table 54: Prevalence of asthma in those with ASD, table 2.

| Source | Sample | Prevalence | Other Notes |
|---|---|---|---|
| Kaas et al. (2021) [58] | Review, N=17 (14 for meta-analysis) | OR 1.12 (0.93-1.34) | P = 0.24, I2 = 89% |
| Brooks et al. (2021) [12] | n=1062 (ASD), n=79175 Age: 1-24; Canada; Electronic Medical Record Primary Care database | Asthma: 20.8% (ASD), 15.2% (non-ASD) | Prevalence ratio: 1.36 (95% CI: 1.21, 1.53)-adjusted for age, sex, neighborhood income, geographic location |
| Schott et al. (2022) [100] | n=155,617 (ASD), n=466,851 (non-ASD); Adults; Medicaid; 2008–12 | 12.47% (ASD), 14.44% (non-ASD) | aOR: 0.77 (0.75 0.78) |
| Liu et al. (2023) [74] | n=5291 (ASD), n=4195596 (non-ASD); Age: >45; Swedish nationwide Total Population Register and National Patient Register; born 1932-67; cumulative incidence uses 1:10 sex and birth year matched cohort | Asthma: 3.9% (ASD), 3.0% (non-ASD) [prevalence over follow up period]; 6.3% (ASD), 4.8% (non-ASD) [estimated cumulative incidence by age 70] | aOR: 1.85 (95% CI: 1.62-2.13), p:<0.0001 (ASD vs non-ASD); aOR: 1.92 (95% CI: 1.65-2.24), p:<0.0001 (ASD without ID vs non-ASD); aOR: 1.61 (95% CI: 1.18-2.19), p: 0.0026 (ASD+ID vs non-ASD); p: 0.31 (ASD without ID vs ASD+ID); aOR: 1.40 (95% CI: 1.13-1.74), p: 0.0022 (men with ASD vs men without ASD); aOR: 2.38 (95% CI: 1.99-2.84), p:<0.0001 (women with ASD vs women without ASD); p: 0.0002 (men with ASD vs women with ASD) [all aORs based on cumulative incidence; adjusted for sex and birth year] |
| Tint et al. (2023) [113] | n=3220 (ASD, women), n=12920 (no DD, women), n=3217 (other DD, women), n=7426 (ASD, men), n=29687 (no DD, men), n=7398 (other DD, men); Canada; administrative and social services data - ICES | Asthma: 19.0% (ASD, women), 16.1% (other DD, women), 21.4% (no DD, women), 19.0% (ASD, men), 17.0% (no DD, men), 21.3% (other DD, men) | aRR: 1.17 (95% 1.08-1.27) [ASD vs no DD, women], aRR: 0.91 (95% 0.82-1.00) [ASD vs other DD, women], aRR: 1.12 (95% 1.06-1.18) [ASD vs no DD, men], aRR: 0.90 (95% 0.85-0.96) [ASD vs other DD, men] |

Table 55: Prevalence of asthma in those with ASD, table 3.

| Source | Sample | Prevalence | Other Notes |
|---|---|---|---|
| Semovski et al. (2023) [102] | n=2020 (ASD); Age: 12-18; records + interviews; Canada | Asthma: 7.9% (ASD) | |
| Weir et al. (2021) [128] | n=1156 (ASD), n=1212 (non-ASD); Age: >16; online recruitment - survey; 2018-19 | Asthma, estimated: 22% (ASD, female), 12% (ASD, male), 13% (non-ASD, female), 13% (non-ASD, male) | OR: 2.092 (95% CI: 1.576-2.789), p:<0.001 [female], OR: 0.792 (95% CI: 0.499-1.267), p: ns [male] - controlled for lifestyle factors - BMI, alcohol use, smoking |
| Kennedy et al. (2024) [62] | n=246 (ASD); Connecticut Children's Medical Center ASD Assessment Program, EHR, n=246 (ASD) | Asthma: 12.6% | |
| Saad et al. (2024) [97] | n=150 (ASD), n=100 (non-ASD), Egyptian children | 23% (ASD), 7% (non-ASD) | p-values: $\leq 0.01$ |
| Longo et al. (2024) [75] | n=30258 (ASD), n=60516 (non-ASD); Age: 18-64; IBM Market Scan Commercial Claims and Encounters Database; 2019-20 | Asthma: 8.9% (ASD), 6.3% (non-ASD) | aOR: 1.4 (95% CI: 1.3, 1.5) |
| Hunt et al. (2024) [53] | n=813 (ASD), n=2781 (non-ASD); Age: 6-97; National Center for Mental Health database and volunteers; Wales and UK | Asthma: 33.2% (ASD), 16.3% (non-ASD) | p:<0.001 (ASD vs non-ASD); ASD vs non-ASD: aOR: 2.36 (95% 1.84-3.02), p:<0.001 (adjusted for age, gender, antipsychotic use, mood stabilizer, smoking status, COPD, chronic obstructive pulmonary disease); ASD (no ID) vs non-ASD: aOR: 2.32 (95% 1.80-2.98), p:<0.001 (adjusted for COPD, chronic obstructive pulmonary disease) ASD+ID vs non-ASD: aOR: 2.94 (95% 1.60-5.42), p:<0.001 (adjusted for COPD, chronic obstructive pulmonary disease) |

Table 56: Prevalence of asthma in those with ASD, table 4.

| **Source** | **Sample** | **Prevalence** | **Other Notes** |
|---|---|---|---|
| Al-Ayadhi et al. (2025) [2] | n=363 [n=189 (ASD), n=174 (non-ASD)]; Online questionnaire (self or caregiver); Saudi Arabia; August 2022 - January 2023 | 33.3% (ASD), 9.2% (non-ASD) | p: 0.000 |
| Salehi et al. (2025) [98] | n=2568 (ASD); Age: 3-17; The National Survey of Children's Health; USA; 2020-21 | 12.6% | |

Table 57: Prevalence of ASD in those with asthma.

| **Source** | **Sample** | **Prevalence** | **Other Notes** |
|---|---|---|---|
| Gong et al. (2023) [43] | n=1569944 (individuals), n=1704388 (siblings, 356544 pairs), n=3921890 (cousins); Swedish register; 1992-2007 | ASD: (OR 1.39, 95% CI 1.35–1.44) [asthma] | |

**Migraine/Headaches**

Table 58: Prevalence of migraines/headaches in those with ASD, table 1.

| Source | Sample | Prevalence | Other Notes |
|---|---|---|---|
| Croen et al. (2015) [25] | n=1507 (ASD), n=15070 (age, sex matches controls); Adults; Kaiser Permanente Northern California; 2008-12 | Headache: 5.57% (ASD), 5.34% (non-ASD); Migraines: 4.98% (ASD), 5.83% (non-ASD) | Chi-square p value: 0.70; p value: 0.18 |
| Vohra et al. (2017) [123] | n=1772 (ASD), n=5316 (non-ASD); Age: 22-64; three state Medicaid Analytic eXtract; 2000-08 | Headache/ migraine: 1.0% (ASD), 3.3% (non-ASD) | aOR (95% CI): 0.30 (0.18, 0.49), p: <0.001 |
| Davignon, et al. (2018) [29] | n=4123 (ASD), n=20615 (non-ASD, non-ADHD); Age: 14-25; Kaiser Permanente Northern California, 2013-15 | Migraine: 2.6% (ASD), 2.9% (non-ASD) | aOR (95% CI): 0.79 (0.64-0.98) |
| Vetri (2020) [122] | Review, N=3. Study 1: 105 adults (76 controls) - no ID. Study 2: n=18 ASD. Study 3: n=81 ASD | Migraine: Study 1: 42.7% (ASD), 20.5% (controls). Study 2: 61%. Study 3; 28.4% | |
| Pan et al. (2021) [87] | Review, N=6 [N=5 (case control), N=1 (prevalence study)], n=5240 | Migraine/ headache: Range: 0% - 28.4% | Pooled estimate of proportion: 7.2 (1.7-16.1) |
| Schott et al. (2022) [100] | n=155,617 (ASD), n=466,851 (non-ASD); Adults; Medicaid; 2008–12 | Headaches: 13.32% (ASD), 15.18% (non-ASD) | aOR: 0.70 (0.68 0.71) |

Table 59: Prevalence of migraines/headaches in those with ASD, table 2.

| Source | Sample | Prevalence | Other Notes |
|---|---|---|---|
| Schott et al. (2022) [100] | n=155,617 (ASD), n=466,851 (non-ASD); Adults; Medicaid; 2008–12 | Migraines: 3.51% (ASD), 4.07% (non-ASD) | aOR: 0.65 (0.62 0.68) |
| Underwood et al. (2022) [118] | n=7943 (core ASD), n=25941 (control); Age: ≥18; UK; SAIL Databank, 2001-16 | Tension headache: 0.8% (ASD), 1.20% (non-ASD) | |
| Underwood et al. (2022) [118] | n=7943 (core ASD), n=25941 (control); Age: ≥18; UK; SAIL Databank, 2001-16 | Migraine: 3.90% (ASD), 3.80% (non-ASD) | |
| Ward et al. (2023) [127] | n=1129 (ASD), n=1176 (non-ASD); Adults; Online survey | Migraine: 22.70% (ASD), 14.81% (non-ASD) | p-value: <0.0001 |
| Semovski et al. (2023) [102] | n=2020 (ASD); Age: 12-18; records + interviews; Canada | Migraine: 2.3% (ASD) | |
| Kennedy et al. (2024) [62] | n=246 (ASD); Connecticut Children's Medical Center ASD Assessment Program electronic records; 2018-19 | Headache/migraine: 1.2% | |
| Hunt et al. (2024) [53] | n=813 (ASD), n=2781 (non-ASD); Age: 6-97; National Center for Mental Health database and volunteers; Wales and UK | Migraine headaches: 33.2% (ASD), 15.5% (non-ASD) | p:<0.001 (ASD vs non-ASD); ASD vs non-ASD: aOR: 3.45 (95% 2.66-4.48), p:<0.001 (adjusted for age, gender, antipsychotic use, mood stabilizer, smoking status, COPD, chronic obstructive pulmonary disease); ASD (no ID) vs non-ASD: aOR: 3.52 (95% 2.70-4.58), p:<0.001 (adjusted for COPD, chronic obstructive pulmonary disease) ASD+ID vs non-ASD: aOR: 2.63 (95% 1.33-5.23), p: 0.006 (adjusted for COPD, chronic obstructive pulmonary disease) |

Table 60: Prevalence of migraines/headaches in those with ASD, table 3.

| **Source** | **Sample** | **Prevalence** | **Other Notes** |
|---|---|---|---|
| O'Nions et al. (2024) [84] | n=15675 (ASD, no ID), n=156750 (comparison), n=6437 (ASD+ID), n=64370 (comparison); Age: ≥18; UK; IQVIA Medical research data (electronic health records from multiple hospitals); comparison matched by age, sex, location | Migraine: 5.61% (ASD, no ID), 5.04% (comparison), 2.61% (ASD+ID), 5.35% (comparison) [baseline]; crude incidence based on observation period: 33.91(28.81-39.65) (ASD, no ID), 33.87 (32.29-35.51) (comparison), 18.76(13.93-24.76) (ASD+ID), 32.76(30.61-35.02) (comparison) | p:<0.05 (ASD no ID vs comparison), p:<0.001 (ASD+ID vs comparison) [baseline]; ASD no ID vs comparison; ASD+ID vs comparison, incidence rate ratio: 0.55(0.42-0.74) (ASD+ID), 0.99 (0.84-1.17) (ASD no ID) |
| Salehi et al. (2025) [98] | n=2568 (ASD); Age: 3-17; The National Survey of Children's Health; USA; 2020-21 | Frequent/Severe Headaches: 6.2% | OR: 1.7, p = 0.002) [Female vs Male] |

### 1.3.3 Folate Metabolism

Table 61: Prevalence of variations in the folate metabolism in those with ASD.

| Source | Sample | Prevalence | Other Notes |
|---|---|---|---|
| Quadros et al. (2018) [89] | n= 81 (ASD) [n=17 girls, n=64 boys], n=135 (non-ASD Parents) [n=70 Mothers, n=65 Fathers], n=53 (non-ASD siblings) [n=30 sisters, n=23 brothers], n=52 (non-ASD controls) [n=28 female, n=24 male] | Blocking antibodies: 40.7% (ASD, all); 52.9% (ASD, girls), 37.5% (ASD, boys); 37.8% (non-ASD Parents, all); 31.4% (non-ASD Mothers), 44.6% (non-ASD Fathers); 43.4% (non-ASD siblings, all); 40.0% (non-ASD sisters), 47.8% (non-ASD brothers); 3.8% (non-ASD controls, all); 3.6% (non-ASD controls, female), 4.2% (non-ASD controls, male); Binding antibodies: 53.7% (ASD, all); 52.9% (ASD, girls), 53.8% (ASD, boys); 40.0% (non-ASD Parents, all); 35.7% (non-ASD Mothers), 44.6% (non-ASD Fathers); 54.7% (non-ASD siblings, all); 63.3% (non-ASD sisters), 43.5% (non-ASD brothers); 26.9% (non-ASD controls, all); 21.4% (non-ASD controls, female), 33.3% (non-ASD controls, male) | |
| Zhou et al. (2018) [134] | n=40 (ASD), n= 42 (non-ASD); Age: 2-6; Department of Psychology at Shenzhen Children's Hospital | Serum FRAA: 77.5% (ASD) 54.8% (non-ASD) | |
| Ramaekers et al. (2019) [90] | Untreated group: n=84 (ASD) and their parents; mean Age: 4.45; Treatment group: n=82 (ASD) and their parents; mean Age: 4.4; Controls: Age: 1-18 | FRA antibody: 71.4% (ASD, untreated), 30.8% (mothers, untreated), 27.4% (fathers, untreated), 75.6% (ASD, treatment), 34.1% (mothers, treatment), 29.4% (fathers, treatment), 3% (controls) | ) |
| Ramaekers et al. (2020) [91] | n=38 (ASD), n=24 (non-ASD); Age: 3.6-18; NY, USA | Blocking FRAA: 68% (ASD), 3.3% (non-ASD) | |

Table 62: Prevalence of variations in the folate metabolism in those with ASD, table 2.

| Source | Sample | Prevalence | Other Notes |
|---|---|---|---|
| Rossignol and Frye (2021) [94] | Systematic review and Meta-Analysis; N=21 (total, for all categories) | CFD: 38% (ASD, N=8); Blocking FRAA: 46% (ASD, N=5), 30% (parent of ASD, N=3), 38% (non-ASD sibling, N=2), 4% (non-ASD non-relation, N=2), 5% (developmental delay, non-ASD, N=1); Binding FRAA: 49% (ASD, N=4), 23% (parent of ASD, N=2), 40% (non-ASD sibling, N=2), 10% (non-ASD non-relation, N=2); Either FRAA: 71% (ASD, N=5), 45% (parent of ASD, N=4), 61% (non-ASD sibling, N=2), 15% (non-ASD non-relation, N=2) | 95% CI: 11-71%, Q: 85.50. Etiology of CFD [prevalence, (95% CI)]: 83% (69-94%) [Either FRAA, N=5], 43% (0-100%) [Mitochondrial dysfunction, N=2], 14% (0-39%) [Genetic abnormalities, N=6]. 95% CI and Q available for all antibody values. Odds Ratio [95% CI], ASD vs group: Blocking FRAA: 2.10 [1.05-4.21] (parent of ASD), 2.0 [0.26-15.21] (non-ASD sibling), 26.84 [7.84-91.86] (non-ASD non-relation), 25.38 [3.29-196.02] (non-ASD developmental disability); Binding FRAA: 3.62 [0.70-18.84] (parent of ASD), 1.32 [0.44-3.99] (non-ASD sibling), 7.90 [0.70-89.13] (non-ASD non-relation); Either FRAA: 3.56 [1.62-7.79] (parent of ASD), 2.12 [0.40-11.30] (non-ASD sibling), 19.03 [2.36-153.58] (non-ASD non-relation) |
| Phunsawat et al. (2022) [88] | n=89 (ASD) [n=69 males, n=20 females]; Age: 2-15 years; Ramathibodi Hospital, Bangkok, Thailand; 2020-21 | Serum FRAA: 33.7% (ASD) | |
| Stefanyshyn et al. (2025) [107] | n=227 (ASD); Age: 2-13; “Vivere” clinic; 2021-24 | Folate Receptor Alpha Autoantibodies (FRAA), binding: 39.2% (ASD); FRAA, blocking: 3.5%; FRAA, specific soluble folate receptor: 13.2% | |

Table 63: Prevalence of ASD in those with variations in the folate metabolism.

| Source | Sample | Prevalence | Other Notes |
|---|---|---|---|
| Rossignol and Frye (2021) [94] | Systematic review and Meta-Analysis; N=21 (total, for all categories) | ASD: 44% (CFD, N=5) | 95% CI: 21-70%, Q: 16.14 |

**Birth Defects**

Table 64: Prevalence of having a birth defect in those with ASD.

| Source | Sample | Prevalence | Other Notes |
|---|---|---|---|
| Cawthorpe (2018) [18] | n=609 (ASD, male), n=111 (ASD, female), n=28588 (non-ASD, male), n=17,787 (non-ASD, female); Regional cohort - <3 between April 1, 1993 and December 31, 1994 and had a diagnosis by that time (any kind); 16 year study, max age: 18; ICD-9 | | Congenital abnormalities: OR: 1.69, p:<0.05 (males), OR: 2.58, p:<0.05 (females) |
| Dizitzer et al. (2020) [33] | n=459 (ASD), n=2285 (non-ASD); Children, southern Israel, Negev Autism Center database, born 2009-16, controls age, sex, ethnicity matched | Congenital diagnosis: 11.8% (ASD), 6.2% (non-ASD) | OR: 2.01 (95% CI: 1.45-2.80), p:<0.001 |
| Pan et al. (2021) [87] | Review, N=6 [N=4 (case control), N=2 (prevalence study)], n=31063 | Congenital nerve system abnormality: Range: 0.2%-12.4% | Pooled estimate of proportion: 3.8 (1.1-8.1) |
| Jadav and Bal (2022) [55] | n=4657 (ASD); Adults; online survey, SPARK; 2022 | Birth defect: 4.2% | |
| Hasler et al. (2023) [48] | n=8760 (ASD), n=26280 (non-ASD); Age: 2-18; Military Health System Data Repository | Neural tube defect: 0.64% (ASD), 0.11% (non-ASD) | OR: 6.00 (3.81–9.44) |
| Micai et al. (2023) [79] | Meta-analysis of observational studies, N=12 [N=5 (clinical studies from community samples), N=7 (population/registry based)], n=21682 | Neurocutaneous Disorder: Pooled prevalence: 0%, Range: 0% - 1% | |

Table 65: Prevalence of having ASD in those with a birth defect.

| Source | Sample | Prevalence | Other Notes |
|---|---|---|---|
| Tillman et al. (2018) [111] | n=7842 (non-syndromic orofacial clefts), n=78409 (non-ASD), n=9637 (non-ASD siblings); Children; Registers; Sweden; 1973-2012 | | OFC vs non: (aHR = 1.96, 95% CI: 1.65-2.33) [ASD] |
| Junaid et al. (2022) [57] | n=1421 (craniofacial anomolies), n=564,804 (controls); Children, Age: >5; Australian; Population Datasets; born 1983-2005 | 0.8% (craniofacial anomalies), 0.4% (controls) | ASD: (adjusted PR 2.11, 95% CI 1.16, 3.80) [children with CFA vs without] |
| Kassa et al. (2023) [60] | n=614 (esophageal atresia), n=3040 (controls); Age: 5-45; Sweden, born 1973–2018 | ASD: 3.9% (EA), 2.4% (non-EA) | ASD: 1.66 (95% CI, 1.05–2.64) [EA] |
| Cook et al. (2025) [24] | N=6, Review | ASD: Mean: 2.87%, Range: 0% - 50.00% (syndromic and non-syndromic orofacial clefts) | |
| Arora et al. (2025) [6] | n=185 (isolated nonsyndromic cleft palate), n=52 (isolated cleft lip), n=29147 (control); Age: 5-21; 1998-2014 | 4.3% (INCP), 11.5% (isolated cleft lip), 5.5% (control) | |

**Congenital Diaphragmatic Hernia**

Table 66: Prevalence of having ASD in those with congenital diaphragmatic hernia.

| Source | Sample | Prevalence | Other Notes |
|---|---|---|---|
| Danzer et al. (2018) [27] | n=110 CDH survivors; Pulmonary Hypoplasia Program; 2004-15; Compared to TD rates from ADDMN | 13.6% (CDH) | 9.3x greater risk, p=0.0002 |
| Kutasy et al. (2024) [66] | n=641 (non-syndromic CDH), n=3080 (controls); Age: $\geq$2; Sweden; Nationwide, Population; 1982-2015 | ASD: 3.9% (CDH), 2.1% (controls) | p-value: 0.011 |

**Down Syndrome**

Table 67: Prevalence of Down syndrome in those with ASD.

| Source | Sample | Prevalence | Other Notes |
|---|---|---|---|
| Croen et al. (2015) [25] | n=1507 (ASD), n=15070 (age, sex matches controls); Adults; Kaiser Permanente Northern California; 2008-12 | Downs syndrome: 1.39% (ASD), 0.10% (non-ASD) | Chi-square p value: <0.001 |
| Bishop-Fitzpatrick et al. (2018) [10] | n=91 (ASD), n=6186 (sex, birth year matched controls); Age: 0-89; EHR from Marshfield clinic - decedents - Wisconsin | Down syndrome: 7.7% (ASD), 0% (non-ASD) | p:<0.001 |
| Schott et al. (2022) [100] | n=155,617 (ASD), n=466,851 (non-ASD); Adults; Medicaid; 2008–12 | Downs syndrome: 2.00% (ASD), 4.58% (non-ASD) | aOR: 0.44 (0.41 0.46) |
| Micai et al. (2023) [79] | Meta-analysis of observational studies, N=16 [N=6 (clinical studies from community samples), N=10 (population/registry based)], n=74916 | Down Syndrome: Pooled prevalence: 1%, Range: 1% - 1% | |
| Salehi et al. (2025) [98] | n=2568 (ASD); Age: 3-17; The National Survey of Children's Health; USA; 2020-21 | Down Syndrome: 0.9% | |

Table 68: Prevalence of ASD in those with Down syndrome.

| Source | Sample | Prevalence | Other Notes |
|---|---|---|---|
| Diniz et al. (2021) [32] | Review, N=15, individual's with Down syndrome, age 2-40 | ASD: 12% to 41% | |

**Other Genetic Conditions**

Table 69: Prevalence of other genetic conditions in those with ASD.

| Source | Sample | Prevalence | Other Notes |
|---|---|---|---|
| Pan et al. (2021) [87] | Review, N=19 [N=10 (case control), N=9 (prevalence study)], n=7413 | Macrocephaly: Range: 0.3% - 31.1% | Pooled estimate of proportion: 13.7 (9.4-18.5) |
| Jadav and Bal (2022) [55] | n=4657 (ASD); Adults; online survey, SPARK; 2022 | Macrocephaly: 1.1% | |
| Khachadourian et al. (2023) [63] | n=42569 (ASD), n=11389 (non-ASD siblings); SPARK; Age: <18; 1999-2019 | Macrocephaly, estimated: 4% (ASD), 1% (non-ASD) | p: <0.05 |
| Jadav and Bal (2022) [55] | n=4657 (ASD); Adults; online survey, SPARK; 2022 | Microcephaly: 0.4% | |
| Khachadourian et al. (2023) [63] | n=42569 (ASD), n=11389 (non-ASD siblings); SPARK; Age: <18 at time of registration; 1999-2019 | Microcephaly, estimated: 1% (ASD), <1% (non-ASD); Short stature, estimated: 3% (ASD), 2% (non-ASD); Other growth condition, estimated: 2% (ASD), 1% (non-ASD) | p: <0.05; p: <0.05; p: <0.05 |
| Marlborough et al. (2021) [77] | N=34; females only; Review | Fragile X Syndrome: 14% | 95% CI: 13–18% |
| Micai et al. (2023) [79] | Meta-analysis of observational studies, N=20 [N=12 (clinical studies from community samples), N=8 (population/registry based)], n=20802 | Fragile X Syndrome: Pooled prevalence: 2%, Range: 1% - 3% | |
| Micai et al. (2023) [79] | Meta-analysis of observational studies, N=9 [N=4 (clinical studies from community samples), N=5 (population/registry based)], n=56118 | Chromosomal abnormality: Pooled prevalence: 2%, Range: 1% - 4% | |
| Micai et al. (2023) [79] | Meta-analysis of observational studies, N=1 [N=1 (clinical studies from community samples), N=0 (population/registry based)], n=92 | Rett syndrome: Pooled prevalence: 2%, Range: 1% - 8% | |

Table 70: Prevalence of ASD in those with other genetic conditions, table 1.

| Source | Sample | Prevalence | Other Notes |
|---|---|---|---|
| Eijk et al. (2018) [36] | n=128 (Neurofibromatosis type 1); Age: 2-10; ENCORE patients; 2011-16 | ASD: 10.9% (using DSM-IV), 25.2% (using SRS), 18.8% (using ADOS) | |
| Morotti et al. (2021) [82] | n=968 (neurofibromatosis type 1); children; Oregon Health & Science University Electronic Medical Records database | ASD: 2.07% (ICD-10) | (2.07%; 95% CI: 1.3–3.2%) |
| Wheeler et al. (2015) [130] | n=758 (fragile X syndrome) [n=639 (male), n=119 (female)]; Age: 2-67; Large family survey, US CDC | ASD: 38.7% (ASD, male, parent reported DSM-IV-TR), 24.7% (ASD, female, parent reported DSM-IV-TR); 27.8% (ASD, male, DSM-5), 11.3% (ASD, female, DSM-5) | |
| Hartley et al. (2015) [47] | n=70 (age 18-21), n=116 (age 22-29), n=70 (age 30-39), n=34 (40-49) [fragile X syndrome - some may not have full mutation], men | ASD criteria: 43.6% (18-21), 37.5% (22-29), 35.8% (30-39), 29.8% (40-49) [DSM-IV TR]; 23.7% (18-21), 21.1% (22-29), 18.7% (30-39), 25.4% (40-49) [DSM-5] | |
| Lee et al. (2016) [70] | n=65 [n=31 (male), n=34 (female)](fragile X syndrome), n=19 (ASD, male); Children; Recruited from genetics clinics and physicians offices | ASD: 41.7% (FXS, total); 54.8% (ASD, male), 41.5% (ASD, female) | |
| Kaufmann (2017) [61] | n=713 (fragile X syndrome); Age; 3-21; FORWARD Dataset; 2012-14 | ASD: 51% (males), 18% (females) (DSM-5 criteria) | p:<0.001 (male vs female) |
| Abbeduto et al. (2019) [1] | n=44 (FXS, male); Age: 15-22; ADOS-2 | ASD: 75% (ADOS and ADI-R) | |

Table 71: Prevalence of ASD in those with other genetic conditions, table 1.

| Source | Sample | Prevalence | Other Notes |
|---|---|---|---|
| Haebig et al. (2020) [45] | n=33 (fragile X syndrome); Age; 9-16.4 | ASD: 96.97% (ADOS), 90.91% (Autism Diagnostic Interview, Revised), 51.52% (Childhood Autism Rating Scale, 2nd edition), 48.48% (all three) | |
| Roberts et al. (2020) [93] | n=51 (fragile X syndrome); Age: preschool | ASD: 60.7% (DSM-5) | 92% (developmental delay), 45.4% (psychiatric disorder) |
| Capal et al. (2021) [15] | n=138 (tuberous sclerosis complex); Age: 3-36 months; Tuberous Sclerosis Complex Autism Center of Excellence Research Network | ASD: 25% (at 36 months) | |
| Mitchell et al. (2021) [80] | Systematic review and meta-analysis, N=40, n=3387; children; tuberous sclerosis complex | ASD: 28%, range: 15% - 70% | |
| Cao et al. (2025) [14] | n=121 (tuberous sclerosis complex), children, Hangzhou Children's Hospital, 2021-23 | ASD: 42.15% | |
| Ramani et al. (2025) [92] | Review, individual's tuberous sclerosis complex | ASD: 17% to 63% | |
| Dykens et al. (2017) [35] | n=146 (Prader-Willi syndrome); Age: 4-21; ADOS-2 + histories and observations | ASD: 12.3% (comprehensive best guess diagnosis), 21.9% (ADOS-2 only) | |

## 1.4 Neurological

### 1.4.1 Epilepsy/Seizures

Table 72: Prevalence of epilepsy in those with ASD, table 1.

| Source | Sample | Prevalence | Other Notes |
|---|---|---|---|
| Croen et al. (2015) [25] | n=1507 (ASD), n=15070 (age, sex matches controls); Adults; Kaiser Permanente Northern California; 2008-12 | 11.94% (ASD), 0.73% (non-ASD) | Chi-square p value: <0.001 |
| Aldinger et al. (2015) [3] | n=728 (ASD, children), n=694 (parents), n=184 (non-ASD, siblings); Autism Genetic Resource Exchange | Seizures: 12.2% (ASD, children), 4.1% (parents), 2.7% (non-ASD, siblings) | |
| Houghton et al. (2017) [50] | n=46943 (ASD, commercial), Truven Health MarketScan Commercial Database, 2014; n=46696 (ASD, Medicaid), Truven Health MarketScan Multi-State Medicaid Database; Age: >3 | Epilepsy: 7.10% (commercial), 10.76% (medicaid) | |
| Vohra et al. (2017) [123] | n=1772 (ASD), n=5316 (non-ASD); Age: 22-64; three state Medicaid Analytic eXtract; 2000-08 | Epilepsy: 22.7% (ASD), 4.8% (non-ASD) | aOR (95% CI): 5.84 (4.94, 6.91), p:<0.001 |
| Davignon, et al. (2018) [29] | n=4123 (ASD), n=20615 (non-ASD, non-ADHD); Age: 14-25; Kaiser Permanente Northern California, 2013-15 | 8.5% (ASD), 0.8% (non-ASD) | aOR (95% CI): 11.88 (9.78-14.43) |
| Soke et al. (2018) [106] | n=783 (ASD, age: 4), n=1091 (ASD, age; 8), ADDMN - 5 sites (Arizona, Missouri, New Jersey, Utah, and Wisconsin), 2010 | Epilepsy/seizure disorder: 2.81% (ASD, age: 4), 3.02% (ASD, age: 8) | Adjusted prevalence, 95% CI: 0.99 (0.54, 1.84) [8 vs 4 years] - adjusted for sex, ethnicity, maternal education, location |

Table 73: Prevalence of epilepsy in those with ASD, table 2.

| Source | Sample | Prevalence | Other Notes |
|---|---|---|---|
| Zhang et al. (2019) [133] | n=192 (ASD), n=74059 (non-ASD); Age: 3-12; Epidemiological study, special kindergartens and primary schools; China | Epilepsy: 11.5% (ASD), 0.2% (non-ASD) | p: <0.01 |
| Pacheva et al. (2019) [86] | n=59 (ASD); Children; Medical files | Epilepsy: 44% (ASD) | |
| Lukmanji et al. (2019) [76] | Meta-analysis, N=74, n=283,549 | Epilepsy: 12.1% (median overall period prevalence) | |
| Dizitzer et al. (2020) [33] | n=459 (ASD), n=2285 (non-ASD); Children, southern Israel, Negev Autism Center database, born 2009-16, controls age, sex, ethnicity matched | Epilepsy: 2.4% (ASD), 1.2% (non-ASD) | OR: 2.05 (95% CI: 1.01-4.17), p: 0.042 |
| Dizitzer et al. (2020) [33] | n=459 (ASD), n=2285 (non-ASD); Children, southern Israel, Negev Autism Center database, born 2009-16, controls age, sex, ethnicity matched | Neurological - convulsion related: 2.4% (ASD), 1.2% (non-ASD) | OR: 2.05 (95% CI: 1.01-4.17), p: 0.042 |
| Hand et al. (2020) [46] | n=4685 (ASD), n=46850 (non-ASD); Age: ≥65; Medicare; 2016-17 | Epilepsy: 26.4% (ASD), 1.9% (non-ASD) | aOR: 18.9 (17.2 - 20.7) |
| Huang et al. (2021) [51] | n=6599 (ASD), n=26396 (non-ASD); controls age, sex, date matched 1:4; Taiwan National Health Insurance Research Database; ICD-9; 2000-15 | Epilepsy: 9.8% (ASD), 0.8% (non-ASD) | p: <0.01 |

Table 74: Prevalence of epilepsy in those with ASD, table 3.

| Source | Sample | Prevalence | Other Notes |
|---|---|---|---|
| Bougeard et al. (2021) [11] | Review: N=12 | Epilepsy/Seizures, Range: 0% - 77.50% | |
| Chien et al. (2021) [23] | n=3837 (ASD), n=38370 (non-ASD); Children; Taiwan's claims database; 2000, 2005, 2010 | 13% (ASD), 1.9% (non-ASD) | |
| Pan et al. (2021) [87] | Review, N=51 [N=16 (case control), N=35 (prevalence study)], n=94539 | Range: 2.3% - 72.5% | Pooled estimate of proportion: 14.2 (11.3-17.2) |
| Rødgaard et al. (2021) [96] | n=11,914 (ASD, men), n=4212 (ASD, women); Age $\leq$16; Danish National Patient Registry | Epilepsy: 6% (men with ASD), 7% (women with ASD) | |
| Bishop et al. (2021) [9] | n=2738 (ASD, ID), n=4775 (ASD, no ID), n=18429 (ID, no ASD); Age: $\geq$21; Wisconsin, Medicaid data | 11.1% (ASD, no ID), 34.6% (ASD, ID), 27% (ID, no ASD) | |
| Angell et al. (2021) [4] | n=18355 (ASD, girls), n=65145 (ASD, boys); Age: 1-21; healthcare claims data; integrates ICD-9 and ICD-10 codes | Epilepsy: 31.26% (ASD, girls), 25.44% (ASD, boys) | aOR: 1.33 (95% CI: 1.28-1.38), p:<0.001 - adjusted for age, race, ethnicity, urbanicity, and insurance |
| Jadav and Bal (2022) [55] | n=4657 (ASD); Adults; online survey, SPARK; 2022 | 6.4% | |
| Schott et al. (2022) [100] | n=155,617 (ASD), n=466,851 (non-ASD); Adults; Medicaid; 2008–12 | Epilepsy and recurrent seizures: 18.40% (ASD), 14.27% (non-ASD) | aOR: 1.39 (1.36 1.42) |

Table 75: Prevalence of epilepsy in those with ASD, table 4.

| Source | Sample | Prevalence | Other Notes |
|---|---|---|---|
| Shoaib et al. (2022) [104] | n=29694 (ASD, medicaid), IBM Marketscan Multi-State Medicaid Database; n=6719 (ASD, Optum), Optum De-Identified Clinformatics Data Mart Database, Date of Death; US; 2015-16 | Seizure and seizure disorder: 6.41% (Optum), 8.44% (Medicaid) | |
| Underwood et al. (2022) [118] | n=7943 (core ASD), n=25941 (control); Age: $\geq$18; UK; SAIL Databank, 2001-16 | 8.90% (ASD), 1.10% (non-ASD) | |
| Koceski and Trajkovski (2022) [65] | n=72 (ASD), n=75 (non-ASD); Age: 3-24; Rochester Health Status Survey IV, Macedonia; 2019 | Epilepsy: 19.4% (ASD), 2.7% (non-ASD) | p: 0.001 |
| Hughes et al. (2023) [52] | n=1846 (ASD); ADDM Network (5 sites, USA); 2018 | 5% | |
| Micai et al. (2023) [79] | Meta-analysis of observational studies, N=84 [N=55 (clinical studies from community samples), N=29 (population/registry based)], n=250,800 | Pooled prevalence: 16%, Range: 14% - 18% | |
| Tint et al. (2023) [113] | n=3220 (ASD, women), n=12920 (no DD, women), n=3217 (other DD, women), n=7426 (ASD, men), n=29687 (no DD, men), n=7398 (other DD, men); Canada; administrative and social services data - ICES | Epilepsy: 11.8% (ASD, women), 0.7% (other DD, women), 15.6% (no DD, women), 11.9% (ASD, men), 0.9% (no DD, men), 12.7% (other DD, men) | aRR: 17.10 (95% 13.66-21.40) [ASD vs no DD, women], aRR: 0.75 (95% 0.66-0.85) [ASD vs other DD, women], aRR: 13.72 (95% 11.97-15.72) [ASD vs no DD, men], aRR: 0.93 (95% 0.85-1.01) [ASD vs other DD, men] |
| Khachadourian et al. (2023) [63] | n=42569 (ASD), n=11389 (non-ASD siblings); SPARK; Age: <18 at time of registration; 1999-2019 | Seizure disorder or epilepsy, estimated: 5% (ASD), 1% (non-ASD) | p: <0.05 |

Table 76: Prevalence of epilepsy in those with ASD, table 5.

| Source | Sample | Prevalence | Other Notes |
|---|---|---|---|
| Semovski et al. (2023) [102] | n=2020 (ASD); Age: 12-18; records, interviews; Canada | Epilepsy or seizure disorder: 4.8% (ASD) | |
| O'Nions et al. (2024) [85] | n=13,172 (ASD, men, no ID), n=3958 (ASD, women, no ID) n=4850 (ASD+ID, men) n=1600 (ASD+ID, women); comparison are 1:10 matched by age, sex, ID status, general practice registered to; mortality study; UK; 1989-2019 | Epilepsy: Men: 5.1% (ASD, no ID); 0.97% (comparison). Women: 5.94% (ASD, no ID); 0.94% (comparison). Men: 20.31% (ASD + ID); 1.05% (comparison). Women: 24.88% (ASD + ID); 1.07% (comparison) | |
| Kennedy et al. (2024) [62] | n=246 (ASD); Connecticut Children's Medical Center ASD Assessment Program electronic records; 2018-19 | Epilepsy: 8.9% | |
| Longo et al. (2024) [75] | n=30258 (ASD), n=60516 (non-ASD); Age: 18-64; IBM Market Scan Commercial Claims and Encounters Database; 2019-20 | Epilepsy: 12.1% (ASD), 1.7% (non-ASD) | aOR: 6.6 (95% CI: 6.2, 7.1) |
| Hunt et al. (2024) [53] | n=813 (ASD), n=2781 (non-ASD); Age: 6-97; National Center for Mental Health database and volunteers; Wales and UK | Epilepsy/Seizure disorder: 8.0% (ASD), 1.8% (non-ASD) | p:<0.001 (ASD vs non-ASD); ASD vs non-ASD: aOR: 3.44 (95% 1.95-6.04), p:<0.001 (adjusted for age, gender, antipsychotic use, mood stabilizer, smoking status, COPD, chronic obstructive pulmonary disease); ASD (no ID) vs non-ASD: aOR: 3.36 (95% 1.89-5.96), p:<0.001 (adjusted for COPD, chronic obstructive pulmonary disease) ASD+ID vs non-ASD: aOR: 4.41 (95% 1.41-13.82), p: 0.011 (adjusted for COPD, chronic obstructive pulmonary disease) |

Table 77: Prevalence of epilepsy in those with ASD, table 6.

| Source | Sample | Prevalence | Other Notes |
|---|---|---|---|
| Salehi et al. (2025) [98] | n=2568 (ASD); Age: 3-17; The National Survey of Children's Health; USA; 2020-21 | 4.2% | OR: 1.7, p = 0.014 [Female vs Male] |
| Toman et al. (2025) [114] | n=240 (ASD); Age: 4-7 (IQR); Al-Subtain Academy, Imam Hussein Centre, Baghdad's National Centre; 2024 | Epilepsy: 14.2% | |
| Kielty et al. (2025) [64] | n=1354 (ASD); Age: 4-21; Autism Inpatient Collection; caregiver-reported information | Epilepsy: 18.9% (ASD) | |

Table 78: Prevalence of ASD in those with epilepsy.

| Source | Sample | Prevalence | Other Notes |
|---|---|---|---|
| Strasser et al. (2017) [109] | Review, N=19 | ASD: 6.3% (epilepsy, all); 4.7% (general epilepsy), 19.9% (infantile spasms), 41.9% (focal seizures), 47.4% (Dravet syndrome) | |
| Lukmanji et al. (2019) [76] | Meta-analysis, N=74, n=283,549 | | ASD: 9.0% (epilepsy population) |
| Holmes et al. (2021) [49] | n=662 (epilepsy); Children; Developmental Epilepsy Clinic; 2016-19 | ASD: 31.35% | |

## 1.5 Motor-Sensory Conditions

### 1.5.1 Connective Tissue Disorders

Table 79: Prevalence of hypermobility in those with ASD, table 1.

| Source | Sample | Prevalence | Other Notes |
|---|---|---|---|
| Glans et al. (2022) [41] | n=199 (ASD), n=419 (controls); Age: 18-65; Sweden; BSS criteria | Generalized joint hypermobility: 28.0% (ASD, female), 10.7% (ASD, male), 11.0% (non-ASD, female), 4.7% (non-ASD, male) | |
| Glans et al. (2022) [41] | n=199 (ASD), n=419 (controls); Age: 18-65; Sweden; BSS criteria | Symptomatic generalized joint hypermobility: 26.1% (ASD, female), 24.0% (ASD, male), 25.0% (non-ASD, female), 12.8% (non-ASD, male) | |
| Ward et al. (2023) [127] | n=716 (ASD, female), n=804 (non-ASD, female); Adults; Online survey | Hypermobility: 4.48% (ASD, female), 0.62% (non-ASD, female); Ehler-Danlos: 6.29% (ASD, female), 0.87% (non-ASD, female) | p-value: 0.00011; p-value: <0.0001 |
| Liu et al. (2023) [74] | n=5291 (ASD), n=4195596 (non-ASD); Age: >45; Swedish nationwide Total Population Register and National Patient Register; born 1932-67; cumulative incidence uses 1:10 sex and birth year matched cohort | : 1.7% (ASD), 0.8% (non-ASD) [prevalence over follow up period]; 1.2% (ASD), 1.9% (non-ASD) [estimated cumulative incidence by age 70] | aOR: 1.04 (95% CI: 0.77-1.40), p: 0.79 (ASD vs non-ASD); aOR: 1.24 (95% CI: 0.91-1.70), p: 0.17 (ASD without ID vs non-ASD); aOR: NA, p:NA (ASD+ID vs non-ASD); p:NA (ASD without ID vs ASD+ID); aOR: 0.80 (95% CI: 0.46-1.37), p: 0.41 (men with ASD vs men without ASD); aOR: 1.26 (95% CI: 0.88-1.80), p: 0.21 (women with ASD vs women without ASD); p: 0.17 (men with ASD vs women with ASD) [AORs from cumulative incidence; adjusted for sex and birth year] |

Table 80: Prevalence of hypermobility in those with ASD, table 2.

| Source | Sample | Prevalence | Other Notes |
|---|---|---|---|
| Liu et al. (2023) [74] | n=5291 (ASD), n=4195596 (non-ASD); Age: >45; Swedish nationwide Total Population Register and National Patient Register; born 1932-67; cumulative incidence uses 1:10 sex and birth year matched cohort | Dorsopathies: 7.9% (ASD), 10.3% (non-ASD) [prevalence over follow up period]; 12.8% (ASD), 16.6% (non-ASD) [estimated cumulative incidence by age 70] | aOR: 0.98 (95% CI: 0.89-1.08), p: 0.71 (ASD vs non-ASD); aOR: 1.13 (95% CI: 1.02-1.26), p: 0.016 (ASD without ID vs non-ASD); aOR: 0.47 (95% CI: 0.35-0.62), p:<0.0001 (ASD+ID vs non-ASD); p:<0.0001 (ASD without ID vs ASD+ID); aOR: 0.86 (95% CI: 0.75-0.98), p: 0.020 (men with ASD vs men without ASD); aOR: 1.18 (95% CI: 1.03-1.36), p: 0.019 (women with ASD vs women without ASD); p: 0.0010 (men with ASD vs women with ASD) [AORs from cumulative incidence; adjusted for sex and birth year] |
| Baeza-Velasco et al. (2025) [8] | Review, N=15 | Pooled prevalence, joint hypermobility: 22.3% | |

### 1.5.2 Movement and Pain Related Conditions

Table 81: Prevalence of other pain related conditions in those with ASD.

| Source | Sample | Prevalence | Other Notes |
|---|---|---|---|
| Ward et al. (2023) [127] | n=1129 (ASD), n=1176 (non-ASD); Adults; Online survey | Fibromyalgia: 6.65% (ASD), 1.79% (non-ASD) | p-value: <0.0001 |
| Lever and Geurts (2016) [71] | n=138 (ASD), n=170 (non-ASD); Age: 19-79; Mental Health Institutions and online recruitment; Netherlands | Somatoform disorder: 5.8% (ASD), 1.8% (non-ASD) | p-value:<0.1 |
| Schendel et al. (2016) [99] | n=20492 (ASD), 1892412 (non-ASD); Age: >1.5; Danish Civil Registration Service and the Danish Medical Birth Register; born 1980-2010 | Neurotic, stress-related, or somatoform disorder: 16.9% (ASD), 3.4% (non-ASD) | |
| Micai et al. (2023) [79] | Meta-analysis of observational studies, N=4 [N=3 (clinical studies from community samples), N=1 (population/registry based)], n=186 | Somatic Symptom and related disorders: Pooled prevalence: 28%, Range: 5% - 58% | |
| Carpita et al. (2024) [16] | N=4, Review | No specific numbers reported in most studies, but generally ASD individuals had more chronic pain and somatic symptoms | |

### 1.5.3 Visual and Auditory Disorders

#### Hearing

Table 82: Prevalence of hearing issues in those with ASD, table 1.

| Source | Sample | Prevalence | Other Notes |
|---|---|---|---|
| Danesh et al. (2015) [26] | n=55 (asperger's); Age: 4-42; mailing list if center for autism and related disabilities; case-history survey and questionnaire | Tinnitus: 35%, Hyperacusis: 69%, Both: 31% | |
| Croen et al. (2015) [25] | n=1507 (ASD), n=15070 (age, sex matches controls); Adults; Kaiser Permanente Northern California; 2008-12 | Hearing impairment: 4.71% (ASD), 2.02% (non-ASD) | Chi-square p value: <0.001 |
| Vohra et al. (2017) [123] | n=1772 (ASD), n=5316 (non-ASD); Age: 22-64; three state Medicaid Analytic eXtract; 2000-08 | Hearing impairments: 18.5% (ASD), 3.9% (non-ASD) | aOR (95% CI): 5.54 (4.62, 6.66), p:<0.001 |
| Rydzewska et al. (2018) [95] | n=6649 (ASD), n=3739935 (non-ASD); Age: ≥25; Scotland Census data; 2011 | Deafness/partial hearing loss: 14.1% (ASD), 9.1% (non-ASD) | |
| Davignon, et al. (2018) [29] | n=4123 (ASD), n=20615 (non-ASD, non-ADHD); Age: 14-25; Kaiser Permanente Northern California, 2013-15 | Hearing impairment: 1.9% (ASD), 0.8% (non-ASD) | aOR (95% CI): 2.54 (1.93 - 3.35) |
| Dizitzer et al. (2020) [33] | n=459 (ASD), n=2285 (non-ASD), [controls age, sex, ethnicity matched]; Children, southern Israel, Negev Autism Center database; Born 2009-16 | Hearing impairment: 2.8% (ASD), 0.6% (non-ASD) | OR: 4.73 (95% CI: 2.21-10.13), p:<0.001 |
| Bougeard et al. (2021) [11] | Review: N=3 | Hearing impairment, Range: 0% - 4.90% | |

Table 83: Prevalence of hearing issues in those with ASD, table 2.

| Source | Sample | Prevalence | Other Notes |
|---|---|---|---|
| Trudeau et al. (2021) [116] | n=39 (ASD), n=15 (Down syndrome), n=10 (Global developmental delay), n=11 (cerebral palsy); Age: 6 mnth-12 yr | Any hearing loss: 12.8% (ASD), 46.1% (DS), 10% (GDD), 36% (CP) | |
| Jadav and Bal (2022) [55] | n=4657 (ASD); Adults; online survey, SPARK; 2022 | Deafness/hearing loss: 6.2% | |
| Schott et al. (2022) [100] | Adults; Medicaid; 2008–12 (1:3 ASD to non-ASD) n=155,617 ASD, n=466,851 non-ASD | Hearing impairment: 9.53% (ASD), 8.00% (non-ASD) | aOR: 1.20 (1.17 1.23) |
| Ward et al. (2023) [127] | n=1129 (ASD), n=1176 (non-ASD); Adults; Online survey | Deafness: 2.93% (ASD), 1.02% (non-ASD); Tinnitus: 7.89% (ASD), 2.13% (non-ASD) | p-value: 0.00293; p-value: <0.0001 |
| Micai et al. (2023) [79] | Meta-analysis; observational studies, N=14 [N=8 (clinical studies from community samples), N=6 (population/registry based)], n=22213 | Hearing Disorder: Pooled prevalence: 4%, Range: 2% - 7% | |
| Liu et al. (2023) [74] | n=5291 (ASD), n=4195596 (non-ASD); Age: >45; Swedish nationwide Total Population Register and National Patient Register; born 1932-67; cumulative incidence uses 1:10 sex and birth year matched cohort | Hearing loss: 4.0% (ASD), 5.1% (non-ASD) [prevalence over follow up period]; 8.6% (ASD), 9.3% (non-ASD) [estimated cumulative incidence by age 70] | aOR: 1.22 (95% CI: 1.06-1.39), p: 0.0046 (ASD vs non-ASD); aOR: 1.24 (95% CI: 1.06-1.44), p: 0.0063 (ASD without ID vs non-ASD); aOR: 1.14 (95% CI: 0.86-1.52), p: 0.35 (ASD+ID vs non-ASD); p: 0.63 (ASD without ID vs ASD+ID); aOR: 1.13 (95% CI: 0.95-1.34), p: 0.17 (men with ASD vs men without ASD); aOR: 1.41 (95% CI: 1.13-1.75), p: 0.0020 (women with ASD vs women without ASD); p: 0.12 (men with ASD vs women with ASD) [AORs from cumulative incidence; adjusted for sex and birth year] |

Table 84: Prevalence of hearing issues in those with ASD, table 3.

| Source | Sample | Prevalence | Other Notes |
|---|---|---|---|
| Khachadourian et al. (2023) [63] | n=42569 (ASD), n=11389 (non-ASD siblings); SPARK; Age: <18 at time of registration; 1999-2019 | Deafness/hearing loss, estimated: 2% (ASD), 1% (non-ASD) | p: <0.05 |
| O'Nions et al. (2024) [85] | n=13,172 (ASD, men, no ID), n=3958 (ASD, women, no ID) n=4850 (ASD+ID, men) n=1600 (ASD+ID, women); comparison are 1:10 matched by age, sex, ID status, general practice registered to; mortality study; UK; 1989-2019 | Severe hearing impairment: Men: 2.03% (ASD, no ID); 1.73% (comparison). Women: 2.78% (ASD, no ID); 1.50% (comparison). Men: 3.15% (ASD + ID); 1.77% (comparison). Women: 4.25% (ASD + ID); 1.64% (comparison) | |
| Salehi et al. (2025) [98] | n=2568 (ASD); Age: 3-17; The National Survey of Children's Health; USA; 2020-21 | Hearing Problems: 3.4% | |

Table 85: Prevalence of ASD in those with hearing issues, table 1.

| Source | Sample | Prevalence | Other Notes |
|---|---|---|---|
| Do et al. (2017) [34] | Review, N=7 (excluded joint vision and hearing as well as some forms of correction) | ASD: 9% (hearing impaired) | |

**Vision**

Table 86: Prevalence of vision issues in those with ASD.

| Source | Sample | Prevalence | Other Notes |
|---|---|---|---|
| Vohra et al. (2017) [123] | n=1772 (ASD), n=5316 (non-ASD); Age: 22-64; three state Medicaid Analytic eXtract; 2000-08 | Blindness/vision: 5.4% (ASD), 3.7% (non-ASD) | aOR (95% CI): 1.49 (1.16, 1.92), $0.001 \leq p < 0.01$; |
| Davignon, et al. (2018) [29] | n=4123 (ASD), n=20615 (non-ASD, non-ADHD); Age: 14-25; Kaiser Permanente Northern California, 2013-15 | Opthamology: 4.0% (ASD), 3.1% (non-ASD) | aOR (95% CI): 1.33 (1.12 - 1.59) |
| Dizitzer et al. (2020) [33] | n=459 (ASD), n=2285 (non-ASD); Children, southern Israel, Negev Autism Center database, born 2009-16, controls age, sex, ethnicity matched | Visual impairment: 1.1% (ASD), 0.4% (non-ASD) | OR: 2.79 (95% CI: 0.93-8.35), p: 0.056 |
| Ward et al. (2023) [127] | n=1129 (ASD), n=1176 (non-ASD); Adults; Online survey | Vision: 44.24% (ASD), 35.83% (non-ASD) | p-value: 0.00012 |
| Liu et al. (2023) [74] | n=5291 (ASD), n=4195596 (non-ASD); Age: >45; Swedish nationwide Total Population Register and National Patient Register; born 1932-67; cumulative incidence uses 1:10 sex and birth year matched cohort | Visual impairment: 2.3% (ASD), 2.0% (non-ASD) [prevalence over follow up period]; 4.7% (ASD), 3.8% (non-ASD) [estimated cumulative incidence by age 70] | aOR: 1.74 (95% CI: 1.46-2.08), p:<0.0001 (ASD vs non-ASD); aOR: 1.56 (95% CI: 1.26-1.94), p:<0.0001 (ASD without ID vs non-ASD); aOR: 2.35 (95% CI: 1.70-3.24), p:<0.0001 (ASD+ID vs non-ASD); p: 0.039 (ASD without ID vs ASD+ID); aOR: 1.56 (95% CI: 1.22-1.99), p: 0.0004 (men with ASD vs men without ASD); aOR: 2.06 (95% CI: 1.59-2.68), p:<0.0001 (women with ASD vs women without ASD); p: 0.13 (men with ASD vs women with ASD) [AORs from cumulative incidence; adjusted for sex and birth year] |
| Salehi et al. (2025) [98] | n=2568 (ASD); Age: 3-17; The National Survey of Children's Health; USA; 2020-21 | Vision Problems: 4.6% | (OR: 2.3, p <0.001 [Female] |

**Blindness and Low vision**

Table 87: Prevalence of blindness and low vision specifically in those with ASD.

| Source | Sample | Prevalence | Other Notes |
|---|---|---|---|
| Croen et al. (2015) [25] | n=1507 (ASD), n=15070 (age, sex matches controls); Adults; Kaiser Permanente Northern California; 2008-12 | Low vision and blindness: 1.06% (ASD), 0.13% (non-ASD) | Chi-square p value: <0.001 |
| Rydzewska et al. (2018) [95] | n=6649 (ASD), n=3739935 (non-ASD); Age: ≥25; Scotland Census data; 2011 | Blindness/partial sight loss: 12.1% (ASD), 3.1% (non-ASD) | |
| Bougeard et al. (2021) [11] | Review: N=3 | Vision impairment, Range: 0% - 14.90% | |
| Jadav and Bal (2022) [55] | n=4657 (ASD); Adults; online survey, SPARK; 2022 | Blindness: 0.8% | |
| Schott et al. (2022) [100] | n=155,617 (ASD), n=466,851 (non-ASD); Adults; Medicaid; 2008–12 | Low vision and blindness: 2.67% (ASD), 2.75% (non-ASD) | aOR: 0.88 (0.84 0.93) |
| Ward et al. (2023) [127] | n=1129 (ASD), n=1176 (non-ASD); Adults; Online survey | Blindness: 2.75% (ASD), 1.87% (non-ASD) | p-value: 0.19 |
| O'Nions et al. (2024) [85] | n=13,172 (ASD, men, no ID), n=3958 (ASD, women, no ID) n=4850 (ASD+ID, men) n=1600 (ASD+ID, women); comparison are 1:10 matched by age, sex, ID status, general practice registered to; mortality study; UK; 1989-2019 | Severe visual impairment: Men: 0.65% (ASD, no ID); 0.26% (comparison). Women: 0.86% (ASD, no ID); 0.31% (comparison). Men: 1.61% (ASD + ID); 0.28% (comparison). Women: 3.06% (ASD + ID); 0.31% (comparison) | |

Table 88: Prevalence of ASD in those with vision issues.

| Source | Sample | Prevalence | Other Notes |
|---|---|---|---|
| Do et al. (2017) [34] | Review, N=10 studies; Clinical studies | ASD: 19% | |
| Fazzi et al. (2019) [37] | n=273 (vision impairment, total), n=214 (cerebral causes), n=59 (peripheral causes); Children; Centre of Child Neuro-opthalmology, Civil Hospital of Brescia, Italy; 2017 | ASD: 2.8% (cerebral causes), 8.4% (peripheral causes) | |

### Specific Eye Conditions

Table 89: Prevalence of other eye conditions in those with ASD, table 1.

| Source | Sample | Prevalence | Other Notes |
|---|---|---|---|
| Butchart et al. (2017) [13] | Review, N=7 | 8.3% (strabismus) | |
| Jadav and Bal (2022) [55] | n=4657 (ASD); Adults; online survey, SPARK; 2022 | Strabismus: 4.0% | |
| Ward et al. (2023) [127] | n=1129 (ASD), n=1176 (non-ASD); Adults; Online survey | Strabismus: 2.57% (ASD), 0.94% (non-ASD) | p-value: 0.0067 |
| Khachadourian et al. (2023) [63] | n=42569 (ASD), n=11389 (non-ASD siblings); SPARK; Age: <18 at time of registration; 1999-2019 | Strabismus, estimated: 3% (ASD), 1% (non-ASD) | p: <0.05 |
| Jadav and Bal (2022) [55] | n=4657 (ASD); Adults; online survey, SPARK; 2022 | Cataracts: 1.8% | e |
| Ward et al. (2023) [127] | n=1129 (ASD), n=1176 (non-ASD); Adults; Online survey | Cataract: 3.46% (ASD), 3.83% (non-ASD) | p-value: 0.66 |
| Ward et al. (2023) [127] | n=1129 (ASD), n=1176 (non-ASD); Adults; Online survey | Near sightedness: 31.10% (ASD), 28.40% (non-ASD) | p-value: 0.19 |
| Ward et al. (2023) [127] | n=1129 (ASD), n=1176 (non-ASD); Adults; Online survey | Farsightedness: 11.53% (ASD), 8.51% (non-ASD) | p-value: 0.03 |
| Ward et al. (2023) [127] | n=1129 (ASD), n=1176 (non-ASD); Adults; Online survey | Astigmatism: 25.10% (ASD), 18.30% (non-ASD) | p-value: 0.00022 |
| Ward et al. (2023) [127] | n=1129 (ASD), n=1176 (non-ASD); Adults; Online survey | Colorblindness: 1.60% (ASD), 1.19% (non-ASD) | p-value: 0.43 |
| Ward et al. (2023) [127] | n=1129 (ASD), n=1176 (non-ASD); Adults; Online survey | Amblyopia: 6.29% (ASD), 3.15% (non-ASD) | p-value: 0.0097 |

Table 90: Prevalence of other eye conditions in those with ASD, table 2.

| Source | Sample | Prevalence | Other Notes |
|---|---|---|---|
| Liu et al. (2023) [74] | n=5291 (ASD), n=4195596 (non-ASD); Age: >45; Swedish nationwide Total Population Register and National Patient Register; born 1932-67; cumulative incidence uses 1:10 sex and birth year matched cohort | Degeneration of macular and posterior pole: 1.3% (ASD), 3.4% (non-ASD) [prevalence over follow up period]; 3.9% (ASD), 4.6% (non-ASD) [estimated cumulative incidence by age 70] | aOR: 1.09 (95% CI: 0.86-1.36), p: 0.46 (ASD vs non-ASD); aOR: 1.25 (95% CI: 0.97-1.61), p: 0.091 (ASD without ID vs non-ASD); aOR: 0.66 (95% CI: 0.36-1.19), p: 0.16 (ASD+ID vs non-ASD); p: 0.051 (ASD without ID vs ASD+ID); aOR: 1.03 (95% CI: 0.76-1.40), p: 0.83 (men with ASD vs men without ASD); aOR: 1.16 (95% CI: 0.81-1.67), p: 0.42 (women with ASD vs women without ASD); p: 0.63 (men with ASD vs women with ASD) [all aORs based on cumulative incidence; adjusted for sex and birth year] |
| Liu et al. (2023) [74] | n=5291 (ASD), n=4195596 (non-ASD); Age: >45; Swedish nationwide Total Population Register and National Patient Register; born 1932-67; cumulative incidence uses 1:10 sex and birth year matched cohort | Peripheral retinal degeneration: 0% (ASD), 0.1% (non-ASD) [prevalence over follow up period]; 0.1% (ASD), 0.1% (non-ASD) [estimated cumulative incidence by age 70] | aOR: 1.09 (95% CI: .86-1.36), p: 0.46 (ASD vs non-ASD); aOR: NA, p:NA (ASD without ID vs non-ASD); aOR: NA, p:NA (ASD+ID vs non-ASD); p:NA (ASD without ID vs ASD+ID); aOR: 1.01 (95% CI: 0.14-7.22), p: 0.99 (men with ASD vs men without ASD); aOR: NA, p:NA (women with ASD vs women without ASD); p:NA (men with ASD vs women with ASD) [all aORs based on cumulative incidence; adjusted for sex and birth year] |

Table 91: Prevalence of other eye conditions in those with ASD, table 3.

| Source | Sample | Prevalence | Other Notes |
|---|---|---|---|
| Liu et al. (2023) [74] | n=5291 (ASD), n=4195596 (non-ASD); Age: >45; Swedish nationwide Total Population Register and National Patient Register; born 1932-67; cumulative incidence uses 1:10 sex and birth year matched cohort | Glaucoma: 2.1% (ASD), 4.0% (non-ASD) [prevalence over follow up period]; 5.2% (ASD), 6.7% (non-ASD) [estimated cumulative incidence by age 70] | aOR: 1.10 (95% CI: 0.91-1.32), p: 0.34 (ASD vs non-ASD); aOR: 1.10 (95% CI: 0.89-1.37), p: 0.37 (ASD without ID vs non-ASD); aOR: 1.07 (95% CI: 0.74-1.56), p: 0.72 (ASD+ID vs non-ASD); p: 0.90 (ASD without ID vs ASD+ID); aOR: 1.01 (95% CI: 0.79-1.30), p: 0.93 (men with ASD vs men without ASD); aOR: 1.21 (95% CI: 0.91-1.61), p: 0.18 (women with ASD vs women without ASD); p: 0.34 (men with ASD vs women with ASD) [AORs from cumulative incidence; adjusted for sex and birth year] |
| Steinhausen et al. (2024) [108] | n=12063 (ASD), n=41251 (non-ASD); Danish register; birth cohort 1984-95; ICD-8 and ICD-10 | | Disease of the eye and adnexa (HR, 95% CI): 2.65 (2.44–2.88) (Age: 0–11), 1.53 (1.36–1.72) (Age: 12–20), 1.22 (1.07–1.39) (Age: 21 +) [ASD vs ref, total]; 2.33 (2.10–2.58) (Age: 0–11), 1.41 (1.23–1.63) (Age: 12–20), 1.05 (0.89–1.25) (Age: 21 +) [Males]; 3.38 (2.94–3.87) (Age: 0–11), 1.79 (1.47–2.17) (Age: 12–20), 1.56 (1.27–1.92) (Age: 21 +) [Females]; 1.22 (1.07–1.39) (Age: 0–11), 1.23 (1.00–1.51) (Age: 12–20), 1.67 (1.32–2.13) (Age: 21 +) [Female vs Male, ASD]; 0.84 (0.75–0.93) (Age: 0–11), 0.97 (0.86–1.10) (Age: 12–20), 1.13 (1.01–1.27) (Age: 21 +) [Female vs Male, non-ASD] |

## 1.6 Hypothalamic-Pituitary-Adrenal (HPA) Dysfunction

### Endocrine

Table 92: Prevalence of having an endocrine issue in those with ASD.

| Source | Sample | Prevalence | Other Notes |
|---|---|---|---|
| Vohra et al. (2017) [123] | n=1772 (ASD), n=5316 (non-ASD); Age: 22-64; three state Medicaid Analytic eXtract; 2000-08 | Non-thyroid endocrine condition: 10.8% (ASD), 4.0% (non-ASD) | aOR (95% CI): 2.92 (2.38, 3.57), p:<0.001 |
| Davignon, et al. (2018) [29] | n=4123 (ASD), n=20615 (non-ASD, non-ADHD); Age: 14-25; Kaiser Permanente Northern California, 2013-15 | Endocrine: 2.5% (ASD), 1.0% (non-ASD) | aOR (95% CI): 2.61 (2.04 - 3.34) |
| Cawthorpe (2018) [18] | n=609 (ASD, male), n=111 (ASD, female), n=28588 (non-ASD, male), n=17,787 (non-ASD, female); Regional cohort - <3 between April 1, 1993 and December 31, 1994 and had a diagnosis by that time (any kind); 16 year study, max age: 18; ICD-9 | | Endocrine: OR: 1.58, p:<0.05 (males), OR: 2.15, p:<0.05 (females) |
| Smith DaWalt et al. (2021) [30] | n=2187 (ASD) [n=1716 (male), n=471 (female)], n=21870 (non-ASD, age and sex matched) [n=17160 (male), n=4710 (female)]; Age: >18; EHRs - Marshfield Clinic - Wisconsin; ICD-9 codes | Endocrine disease: 0.25 (female, ASD), 0.10 (male,ASD), 0.08 (female, control), 0.02 (male, control) [predicted probability] | OR: 4.67 (95% CI: 3.88-5.61), p: <0.001 [ASD status], OR: 0.82 (95% CI: 0.61-1.10) [ASD X Sex] |
| Ward et al. (2023) [127] | n=1129 (ASD), n=1176 (non-ASD); Adults; Online survey | Endocrine, any: 20.57% (ASD), 17.02% (non-ASD) | p-value: 0.04 |

### 1.6.1 Gynecological Conditions

Table 93: Prevalence of having a uterine condition in those with ASD, table 1.

| Source | Sample | Prevalence | Other Notes |
|---|---|---|---|
| Cherskov et al. (2018) [20] | n=971 (women, ASD), n=4855 (controls); Age: ≥21; EHR; Clinical Practice Research Datalink; UK; 1990-2014 | Polycystic ovarian syndrome: 2.3% (ASD), 1.1% (non-ASD) | OR: 2.01 95% CI: 1.22-3.30 |
| Hand et al. (2020) [46] | n=4685 (ASD), n=46850 (non-ASD); Age: ≥65; Medicare; 2016-17 | Menopausal disorders: 1.3% (ASD), 1.1% (non-ASD) | aOR: 1.2 (0.9-1.5) |
| May et al. (2021) [110] | Review, N=3 | PCOS, pooled prevalence: 7.2% (ASD) | |
| Ward et al. (2023) [127] | n=716 (ASD, female), n=804 (non-ASD, female); Adults; Online survey | Polycystic ovarian syndrome: 8.81% (ASD, female), 5.23% (non-ASD, female) | p-value: 0.01 |
| Ward et al. (2023) [127] | n=716 (ASD, female), n=804 (non-ASD, female); Adults; Online survey | Premenstrual tension: 13.99% (ASD, female), 8.59% (non-ASD, female) | p-value: 0.0018 |
| Ward et al. (2023) [127] | n=716 (ASD, female), n=804 (non-ASD, female); Adults; Online survey | Endometriosis: 6.57% (ASD, female), 3.74% (non-ASD, female) | p-value: 0.02 |
| Ward et al. (2023) [127] | n=716 (ASD, female), n=804 (non-ASD, female); Adults; Online survey | Fibroids: 2.79% (ASD, female), 3.61% (non-ASD, female) | p-value: 0.40 |
| Longo et al. (2024) [75] | n=30258 (ASD), n=60516 (non-ASD); Age: 18-64; IBM Market Scan Commercial Claims and Encounters Database; 2019-20 | Menopausal disorders: 0.5% (ASD), 0.4% (non-ASD) | aOR: 1.5 (95% CI: 1.2, 1.9) |

Table 94: Prevalence of having a uterine condition in those with ASD, table 2.

| Source | Sample | Prevalence | Other Notes |
|---|---|---|---|
| O'Nions et al. (2024) [84] | n=15675 (ASD, no ID), n=156750 (comparison), n=6437 (ASD+ID), n=64370 (comparison); Female subgroup; Age: ≥18; UK; IQVIA Medical research data (electronic health records from multiple hospitals); comparison matched by age, sex, location | Gynecological conditions: 8.64% (ASD, no ID), 6.60% (comparison), 9.83% (ASD+ID), 8.50% (comparison) [baseline]; crude incidence based on observation period: 94.27 (75.19-116.71) (ASD, no ID), 79.89(74.47-85.60) (comparison), 93.48 (70.42-121.68) (ASD+ID), 75.91 (69.15-83.16) (comparison) | p:<0.05 (ASD no ID vs comparison), p: not significant (ASD+ID vs comparison) [baseline]; both groups, incidence rate ratio: 1.22 (0.93-1.47) (ASD+ID), 1.19 (0.95-1.49) (ASD no ID) |

Table 95: Prevalence of those with a uterine condition having ASD.

| Source | Sample | Prevalence | Other Notes |
|---|---|---|---|
| Cherskov et al. (2018) [20] | n=26,263 (women, PCOS or PCOS phenotype), n=130,717 (controls); Age: ≥21; Electronic health records; Clinical Practice Research Datalink; UK; 1990-2014 | ASD: 0.17% (PCOS), 0.09% (non-PCOS) | OR: 1.94 95% CI: 1.37-2.76 |
| Tamara May et al. (2021) [110] | Review, N=3 | ASD, pooled prevalence: 0.2% (PCOS) | OR: 1.48 [95% CI: 1.21–1.80] |

### 1.6.2 Thyroid

Table 96: Prevalence of having a thyroid issue in those with ASD, table 1.

| Source | Sample | Prevalence | Other Notes |
|---|---|---|---|
| Croen et al. (2015) [25] | n=1507 (ASD), n=15070 (age, sex matches controls); Adults; Kaiser Permanente Northern California; 2008-12 | Thyroid diseases: 7.03% (ASD), 3.10% (non-ASD) | Chi-square p value: <0.001 |
| Vohra et al. (2017) [123] | n=1772 (ASD), n=5316 (non-ASD); Age: 22-64; three state Medicaid Analytic eXtract; 2000-08 | Thyroid diagnosis: 4.7% (ASD), 1.3% (non-ASD) | 3.66 (2.65, 5.05), p:<0.001 |
| Davignon, et al. (2018) [29] | n=4123 (ASD), n=20615 (non-ASD, non-ADHD); Age: 14-25; Kaiser Permanente Northern California, 2013-15 | Thyroid disease: 1.6% (ASD), 0.6% (non-ASD) | aOR (95% CI): 2.70 (1.98 - 3.69) |
| Bishop-Fitzpatrick et al. (2018) [10] | n=91 (ASD), n=6186 (sex, birth year matched controls); Age: 0-89; EHR from Marshfield clinic - decedents - Wisconsin | Hypothyroidism: 35.2% (ASD), 11.6% (non-ASD) | aOR: 4.76 (adjusted for age and sex), p: <0.001 |
| Hand et al. (2020) [46] | n=4685 (ASD), n=46850 (non-ASD); Age: $\geq$65; Medicare; 2016-17 | Thyroid disorder: 31.7% (ASD), 13.8% (non-ASD) | aOR: 3.1 (2.9-3.3) |
| Schott et al. (2022) [100] | n=155,617 (ASD), n=466,851 (non-ASD); Adults; Medicaid; 2008–12 | Thyroid disease: 11.91% (ASD), 9.86% (non-ASD) | aOR: 1.21 (1.18 1.23) |
| Ward et al. (2023) [127] | n=1129 (ASD), n=1176 (non-ASD); Adults; Online survey | Hypothyroidism: 6.11% (ASD), 6.13% (non-ASD) | p-value: 0.99 |
| Ward et al. (2023) [127] | n=1129 (ASD), n=1176 (non-ASD); Adults; Online survey | Hyperthyroidism: 1.41% (ASD), 1.45% (non-ASD) | p-value: 0.97 |

Table 97: Prevalence of having a thyroid issue in those with ASD, table 2.

| Source | Sample | Prevalence | Other Notes |
|---|---|---|---|
| Liu et al. (2023) [74] | n=5291 (ASD), n=4195596 (non-ASD); Age: >45; Swedish nationwide Total Population Register and National Patient Register; born 1932-67; cumulative incidence uses 1:10 sex and birth year matched cohort | Thyroid disorders: 3.2% (ASD), 3.3% (non-ASD) [prevalence over follow up period]; 5.9% (ASD), 4.0% (non-ASD) [estimated cumulative incidence by age 70] | aOR: 1.81 (95% CI: 1.56-2.11), p:<0.0001 (ASD vs non-ASD); aOR: 1.87 (95% CI: 1.58-2.22), p:<0.0001 (ASD without ID vs non-ASD); aOR: 1.62 (95% CI: 1.16-2.26), p: 0.0042 (ASD+ID vs non-ASD); p: 0.46 (ASD without ID vs ASD+ID); aOR: 2.42 (95% CI: 1.88-3.11), p:<0.0001 (men with ASD vs men without ASD); aOR: 1.60 (95% CI: 1.33-1.94), p:<0.0001 (women with ASD vs women without ASD); p: 0.010 (men with ASD vs women with ASD) [AORs from cumulative incidence; adjusted for sex and birth year] |
| Longo et al. (2024) [75] | n=30258 (ASD), n=60516 (non-ASD); Age: 18-64; IBM Market Scan Commercial Claims and Encounters Database; 2019-20 | Thyroid disorders: 8.3% (ASD), 4.1% (non-ASD) | aOR: 2.1 (95% CI: 2.0, 2.1) |
| Hunt et al. (2024) [53] | n=813 (ASD), n=2781 (non-ASD); Age: 6-97; National Center for Mental Health database and volunteers; Wales and UK | Overactive thyroid/hyperthyroid: 0.8% (ASD), 1.7% (non-ASD) | p: 0.063 (ASD vs non-ASD); ASD vs non-ASD: aOR: 2.11 (95% 0.68-6.55), p: 0.196 (adjusted for age, gender, antipsychotic use, mood stabilizer, smoking status, COPD, chronic obstructive pulmonary disease); ASD (no ID) vs non-ASD: aOR: 1.62 (95% 0.47-5.56), p: 0.444 (adjusted for COPD, chronic obstructive pulmonary disease) ASD+ID vs non-ASD: aOR: 13.89 (95% 2.36-81.99), p: 0.004 (adjusted for COPD, chronic obstructive pulmonary disease) |

Table 98: Prevalence of having a thyroid issue in those with ASD, table 3.

| Source | Sample | Prevalence | Other Notes |
|---|---|---|---|
| Hunt et al. (2024) [53] | n=813 (ASD), n=2781 (non-ASD); Age: 6-97; National Center for Mental Health database and volunteers; Wales and UK | Underactive thyroid/ hypothyroid: 5.0% (ASD), 5.3% (non-ASD) | p: 0.758 (ASD vs non-ASD); ASD vs non-ASD: aOR: 2.18 (95% 1.28-3.70), p: 0.004 (adjusted for age, gender, antipsychotic use, mood stabilizer, smoking status, COPD, chronic obstructive pulmonary disease); ASD (no ID) vs non-ASD: aOR: 2.15 (95% 1.26-3.66), p: 0.005 (adjusted for COPD, chronic obstructive pulmonary disease) ASD+ID vs non-ASD: aOR: 2.81 (95% 0.77-10.33), p: 0.120 (adjusted for COPD, chronic obstructive pulmonary disease) |
| Al-Ayadhi et al. (2025) [2] | n=363 [n=189 (ASD), n=174 (non-ASD)]; Online questionnaire (self or caregiver); Saudi Arabia; August 2022 - January 2023 | Thyroiditis: 3.7% (ASD), 0.6% (non-ASD) | p: 0.042 |

### 1.6.3 Other Endocrine Disorders

Table 99: Prevalence of having other endocrine issue in those with ASD.

| Source | Sample | Prevalence | Other Notes |
|---|---|---|---|
| Croen et al. (2015) [25] | n=1507 (ASD), n=15070 (age, sex matches controls); Adults; Kaiser Permanente Northern California; 2008-12 | Pituitary gland and hypothalamic control: 1.33% (ASD), 0.21% (non-ASD) | Chi-square p value: <0.001 |
| Croen et al. (2015) [25] | n=1507 (ASD), n=15070 (age, sex matches controls); Adults; Kaiser Permanente Northern California; 2008-12 | Puberty and gonadal disorders: 1.26% (ASD), 0.79% (non-ASD) | Chi-square p value: 0.05 |
| Geier and Geier (2021) [40] | n=1593 (ASD), 100143 (non-ASD) [15738 person-years (ASD), 996835 person-years (non-ASD)]; Age: ≤10; Independent Healthcare Research Database; 1990-2009; ICD-9; USA | Precocious puberty: 43.2 per 10000 person-years (ASD), 13.7 per 10000 person-years (non-ASD) | RR: 3.15, p:<0.0001 (frequency modeling), aHR: 4.64, p:<0.0001 (Cox proportional HR modeling) |
| May et al. (2021) [78] | Review, N=2 | Congenital adrenal hyperplasia: 0.1% (ASD) | |
| Schott et al. (2022) [100] | n=155,617 (ASD), n=466,851 (non-ASD); Adults; Medicaid; 2008–12 | Pituitary gland and hypothalamic control: 1.79% (ASD), 1.14% (non-ASD) | aOR: 1.50 (1.42 1.60) |
| Schott et al. (2022) [100] | n=155,617 (ASD), n=466,851 (non-ASD); Adults; Medicaid; 2008–12 | Puberty and gonadal disorders: 2.15% (ASD), 1.72% (non-ASD) | aOR: 1.03 (0.97 1.08) |
| Ward et al. (2023) [127] | n=1129 (ASD), n=1176 (non-ASD); Adults; Online survey | Precocious puberty: 1.77% (ASD), 0.60% (non-ASD) | p-value: 0.02 |

Table 100: Prevalence of having ASD for those with other endocrine issue.

| Source | Sample | Prevalence | Other Notes |
|---|---|---|---|
| May et al. (2021) [78] | Review, N=3 | ASD: 0.9% (Congenital adrenal hyperplasia) 0.1% (ASD) | |

# References


[1] L. Abbeduto, A. John Thurman, A. McDuffie, J. Klusek, R. Tempero Feigles, W. Ted Brown, D. J. Harvey, T. Adayev, G. LaFauci, C. Dobkins, and J. E. Roberts. ASD Comorbidity in Fragile X Syndrome: Symptom Profile and Predictors of Symptom Severity in Adolescent and Young Adult Males. *Journal of autism and developmental disorders*, 49(3):960–977, Mar. 2019. ISSN 0162-3257. doi: 10.1007/s10803-018-3796-2. URL `https://www.ncbi.nlm.nih.gov/pmc/articles/PMC6556533/`.

[2] L. Y. Al-Ayadhi, N. E. Elamin, A. Madani, F. Al-Ghamdi, H. A. Al-Ghamdi, and D. M. Halepoto. Examining the Prevalence, Characteristics, and Potential Links Between Skin Disorders and Autism Spectrum Disorder (ASD). *Journal of Clinical Medicine*, 14(2):469, Jan. 2025. ISSN 2077-0383. doi: 10.3390/jcm14020469. URL `https://www.ncbi.nlm.nih.gov/pmc/articles/PMC11765873/`.

[3] K. A. Aldinger, C. J. Lane, J. Veenstra-VanderWeele, and P. Levitt. Patterns of Risk for Multiple Co-Occurring Medical Conditions Replicate Across Distinct Cohorts of Children with Autism Spectrum Disorder. *Autism Research*, 8(6):771–781, 2015. ISSN 1939-3806. doi: 10.1002/aur.1492. URL `https://onlinelibrary.wiley.com/doi/abs/10.1002/aur.1492`. _eprint: https://onlinelibrary.wiley.com/doi/pdf/10.1002/aur.1492.

[4] A. M. Angell, A. Deavenport-Saman, L. Yin, B. Zou, C. Bai, D. Varma, and O. Solomon. Sex Differences in Co-occurring Conditions among Autistic Children and Youth in Florida: A Retrospective Cohort Study (2012-2019). *Journal of autism and developmental disorders*, 51(10):3759–3765, Oct. 2021. ISSN 0162-3257. doi: 10.1007/s10803-020-04841-5. URL `https://www.ncbi.nlm.nih.gov/pmc/articles/PMC8254818/`.

[5] Y. Aoki and S. Cortese. Mitochondrial Aspartate/Glutamate Carrier SLC25A12 and Autism Spectrum Disorder: a Meta-Analysis. *Molecular Neurobiology*, 53(3):1579–1588, Apr. 2016. ISSN 1559-1182. doi: 10.1007/s12035-015-9116-3. URL `https://doi.org/10.1007/s12035-015-9116-3`.

[6] J. S. Arora, N. Khoshab, M. Kanack, L. Chase, N. Kadakia, S. Vargas, T. Zadeh, and R. M. Vyas. Newly Identified Developmental Delays in a Large Population of Children With Nonsyndromic Cleft Lip and Palate. *Plastic and Reconstructive Surgery – Global Open*, 13(4):e6655, Apr. 2025. doi: 10.1097/GOX.0000000000006655.

[7] K. Austin, A. Turner, A. Maricar, H. Taimuri, and M. Mutalib. Functional gastrointestinal disorders in children with autism spectrum disorders: a systematic review of published literature. *Frontline Gastroenterology*, pages flgastro–2024–102870, Mar. 2025. ISSN 2041-4137, 2041-4145. doi: 10.1136/flgastro-2024-102870. URL `https://fg.bmj.com/lookup/doi/10.1136/flgastro-2024-102870`.

[8] C. Baeza-Velasco, J. Vergne, M. Poli, L. Kalisch, and R. Calati. Autism in the context of joint hypermobility, hypermobility spectrum disorders, and Ehlers–Danlos syndromes: A systematic review and prevalence meta-analyses. *Autism*, page 13623613251328059, Mar. 2025. ISSN 1362-3613. doi: 10.1177/13623613251328059. URL `https://doi.org/10.1177/13623613251328059`.

[9] L. Bishop, K. J. McLean, and E. Rubenstein. Epilepsy in adulthood: Prevalence, incidence, and associated antiepileptic drug use in autistic adults in a state Medicaid system. *Autism*, 25(3):831–839, Apr. 2021. ISSN 1362-3613. doi: 10.1177/1362361320942982. URL `https://doi.org/10.1177/1362361320942982`.

[10] L. Bishop-Fitzpatrick, A. Movaghar, J. S. Greenberg, D. Page, L. S. DaWalt, M. H. Brilliant, and M. R. Mailick. Using machine learning to identify patterns of lifetime health problems in decedents with autism spectrum disorder. *Autism Research*, 11(8):1120–1128, Aug. 2018. ISSN 1939-3792. doi: 10.1002/aur.1960. URL `https://onlinelibrary.wiley.com/doi/full/10.1002/aur.1960`.

[11] C. Bougeard, Françoise Picarel-Blanchot, Ramona Schmid, Rosanne Campbell, and Jan Buitelaar. Prevalence of Autism Spectrum Disorder and Co-morbidities in Children and Adolescents: A Systematic Literature Review. *ResearchGate*, Oct. 2021. doi: 10.3389/fpsyt.2021.744709.

[12] J. D. Brooks, S. E. Bronskill, L. Fu, F. E. Saxena, J. Arneja, V. B. Pinzaru, E. Anagnostou, K. Nylen, J. McLaughlin, and K. Tu. Identifying Children and Youth With Autism Spectrum Disorder in Electronic Medical Records: Examining Health System Utilization and Comorbidities. *Autism Research*, 14(2): 400–410, 2021. ISSN 1939-3806. doi: 10.1002/aur.2419. URL `https://onlinelibrary.wiley.com/doi/abs/10.1002/aur.2419`. _eprint: https://onlinelibrary.wiley.com/doi/pdf/10.1002/aur.2419.

[13] M. Butchart, J. J. Long, M. Brown, A. McMillan, J. Bain, and T. Karatzias. Autism and Visual Impairment: a Review of the Literature.

*Review Journal of Autism and Developmental Disorders*, 4(2):118–131, June 2017. ISSN 2195-7177, 2195-7185. doi: 10.1007/s40489-016-0101-1. URL `http://link.springer.com/10.1007/s40489-016-0101-1`.

[14] J. Cao, M. Liu, T. Zhang, H. Chen, and Z. Liu. Risk Factors for Psychiatric Disorders in Pediatric Patients with Tuberous Sclerosis Complex. *Neuropediatrics*, 56(03):172–179, June 2025. ISSN 0174-304X, 1439-1899. doi: 10.1055/a-2541-8540. URL `http://www.thieme-connect.de/DOI/DOI?10.1055/a-2541-8540`.

[15] J. K. Capal, M. E. Williams, D. A. Pearson, R. Kissinger, P. S. Horn, D. Murray, K. Currans, B. Kent, M. Bebin, H. Northrup, J. Y. Wu, M. Sahin, and D. A. Krueger. Profile of Autism Spectrum Disorder in Tuberous Sclerosis Complex: Results from a Longitudinal, Prospective, Multisite Study. *Annals of Neurology*, 90(6): 874–886, Dec. 2021. ISSN 1531-8249. doi: 10.1002/ana.26249. URL `https://onlinelibrary.wiley.com/doi/10.1002/ana.26249`.

[16] B. Carpita, B. Nardi, V. Tognini, F. Poli, G. Amatori, I. M. Cremone, S. Pini, and L. Dell'Osso. Autistic Traits and Somatic Symptom Disorders: What Is the Link? *Brain Sciences*, 14(3):274, Mar. 2024. ISSN 2076-3425. doi: 10.3390/brainsci14030274. URL `https://www.ncbi.nlm.nih.gov/pmc/articles/PMC10968945/`.

[17] D. Cawthorpe. Comprehensive Description of Comorbidity for Autism Spectrum Disorder in a General Population. *The Permanente Journal*, 21:16–088, Dec. 2016. ISSN 1552-5767. doi: 10.7812/TPP/16-088. URL `https://www.ncbi.nlm.nih.gov/pmc/articles/PMC5283790/`.

[18] D. Cawthorpe. A 16-Year Cohort Analysis of Autism Spectrum Disorder-Associated Morbidity in a Pediatric Population. *Frontiers in Psychiatry*, 9:635, Nov. 2018. ISSN 1664-0640. doi: 10.3389/fpsyt.2018.00635. URL `https://www.ncbi.nlm.nih.gov/pmc/articles/PMC6281889/`.

[19] L. Chen, X.-J. Shi, H. Liu, X. Mao, L.-N. Gui, H. Wang, and Y. Cheng. Oxidative stress marker aberrations in children with autism spectrum disorder: a systematic review and meta-analysis of 87 studies (N = 9109). *Translational Psychiatry*, 11(1):15, Jan. 2021. ISSN 2158-3188. doi: 10.1038/s41398-020-01135-3. URL `https://www.nature.com/articles/s41398-020-01135-3`.

[20] A. Cherskov, A. Pohl, C. Allison, H. Zhang, R. A. Payne, and S. Baron-Cohen. Polycystic ovary syndrome and autism: A test of the prenatal sex steroid theory. *Translational Psychiatry*, 8:136, Aug. 2018. ISSN 2158-3188. doi: 10.1038/s41398-018-0186-7. URL `https://www.ncbi.nlm.nih.gov/pmc/articles/PMC6068102/`.

[21] H.-L. Chiang, C.-J. Liu, Y.-W. Hu, S.-C. Chen, L.-Y. Hu, C.-C. Shen, C.-M. Yeh, T.-J. Chen, and S. S.-F. Gau. Risk of

Cancer in Children, Adolescents, and Young Adults with Autistic Disorder. *The Journal of Pediatrics*, 166(2):418–423.e1, Feb. 2015. ISSN 0022-3476. doi: 10.1016/j.jpeds.2014.10.029. URL https://www.sciencedirect.com/science/article/pii/S0022347614009743.

[22] A. Y. Chieh, B. M. Bryant, J. W. Kim, and L. Li. Systematic review investigating the relationship between autism spectrum disorder and metabolic dysfunction. *Research in autism spectrum disorders*, 86:101821, Aug. 2021. ISSN 1750-9467. doi: 10.1016/j.rasd.2021.101821. URL https://www.ncbi.nlm.nih.gov/pmc/articles/PMC9784428/.

[23] Y.-L. Chien, C.-S. Wu, and H.-J. Tsai. The Comorbidity of Schizophrenia Spectrum and Mood Disorders in Autism Spectrum Disorder. *Autism Research*, 14(3):571–581, 2021. ISSN 1939-3806. doi: 10.1002/aur.2451. URL https://onlinelibrary.wiley.com/doi/abs/10.1002/aur.2451. _eprint: https://onlinelibrary.wiley.com/doi/pdf/10.1002/aur.2451.

[24] B. Cook, S. Van Bockstaele, S. B. Crow, D. Sainsbury, S. Butterworth, and S. Filson. Neurodevelopmental disorders in children with cleft lip and palate: a systematic review. *European Child & Adolescent Psychiatry*, 34(6):1731–1738, June 2025. ISSN 1435-165X. doi: 10.1007/s00787-024-02636-y. URL https://doi.org/10.1007/s00787-024-02636-y.

[25] L. A. Croen, O. Zerbo, Y. Qian, M. L. Massolo, S. Rich, S. Sidney, and C. Kripke. The health status of adults on the autism spectrum. *Autism*, 19(7):814–823, Oct. 2015. ISSN 1362-3613, 1461-7005. doi: 10.1177/1362361315577517. URL https://journals.sagepub.com/doi/10.1177/1362361315577517.

[26] A. A. Danesh, D. Lang, W. Kaf, W. D. Andreassen, J. Scott, and A. A. Eshraghi. Tinnitus and hyperacusis in autism spectrum disorders with emphasis on high functioning individuals diagnosed with Asperger's Syndrome. *International Journal of Pediatric Otorhinolaryngology*, 79(10):1683–1688, Oct. 2015. ISSN 0165-5876. doi: 10.1016/j.ijporl.2015.07.024. URL https://www.sciencedirect.com/science/article/pii/S0165587615003596.

[27] E. Danzer, C. Hoffman, J. A. D'Agostino, J. S. Miller, L. N. Waqar, M. Gerdes, J. C. Bernbaum, H. Rosenthal, N. E. Rintoul, L. M. Herkert, W. H. Peranteau, A. W. Flake, N. S. Adzick, and H. L. Hedrick. Rate and Risk Factors Associated with Autism Spectrum Disorder in Congenital Diaphragmatic Hernia. *Journal of Autism and Developmental Disorders*, 48(6):2112–2121, June 2018. ISSN 1573-3432. doi: 10.1007/s10803-018-3472-6. URL https://doi.org/10.1007/s10803-018-3472-6.

[28] B. W. Darbro, R. Singh, M. B. Zimmerman, V. B. Mahajan, and A. G. Bassuk. Autism Linked to Increased Oncogene Mutations but Decreased Cancer Rate. *PLoS ONE*, 11(3):e0149041, Mar.

2016. ISSN 1932-6203. doi: 10.1371/journal.pone.0149041. URL https://www.ncbi.nlm.nih.gov/pmc/articles/PMC4774916/.

[29] M. N. Davignon, Y. Qian, M. Massolo, and L. A. Croen. Psychiatric and Medical Conditions in Transition-Aged Individuals With ASD. *Pediatrics*, 141(Supplement_4):S335–S345, Apr. 2018. ISSN 0031-4005. doi: 10.1542/peds.2016-4300K. URL https://doi.org/10.1542/peds.2016-4300K.

[30] L. S. DaWalt, J. L. Taylor, A. Movaghar, J. Hong, B. Kim, M. Brilliant, and M. R. Mailick. Health profiles of adults with ASD: Differences between women and men. *Autism research : official journal of the International Society for Autism Research*, 14(9):1896–1904, Sept. 2021. ISSN 1939-3792. doi: 10.1002/aur.2563. URL https://www.ncbi.nlm.nih.gov/pmc/articles/PMC8592037/.

[31] L. Delhey, E. N. Kilinc, L. Yin, J. Slattery, M. Tippett, R. Wynne, S. Rose, S. Kahler, S. Damle, A. Legido, M. J. Goldenthal, and R. E. Frye. Bioenergetic variation is related to autism symptomatology. *Metabolic Brain Disease*, 32(6):2021–2031, Dec. 2017. ISSN 1573-7365. doi: 10.1007/s11011-017-0087-0. URL https://doi.org/10.1007/s11011-017-0087-0.

[32] N. L. F. Diniz, E. Parlato-Oliveira, P. G. A. Pimenta, L. A. de Araújo, and E. R. Valadares. Autism and Down syndrome: early identification and diagnosis. *Arquivos de Neuro-Psiquiatria*, 80(6):620–630, Sept. 2021. ISSN 0004-282X. doi: 10.1590/0004-282X-ANP-2021-0156. URL https://www.ncbi.nlm.nih.gov/pmc/articles/PMC9387185/.

[33] Y. Dizitzer, G. Meiri, H. Flusser, A. Michaelovski, I. Dinstein, and I. Menashe. Comorbidity and health services' usage in children with autism spectrum disorder: a nested case–control study. *Epidemiology and Psychiatric Sciences*, 29:e95, Jan. 2020. ISSN 2045-7960, 2045-7979. doi: 10.1017/S2045796020000050. URL https://www.cambridge.org/core/journals/epidemiology-and-psychiatric-sciences/article/c

[34] B. Do, P. Lynch, E.-M. Macris, B. Smyth, S. Stavrinakis, S. Quinn, and P. A. Constable. Systematic review and meta-analysis of the association of Autism Spectrum Disorder in visually or hearing impaired children. *Ophthalmic and Physiological Optics*, 37(2): 212–224, 2017. ISSN 1475-1313. doi: 10.1111/opo.12350. URL https://onlinelibrary.wiley.com/doi/abs/10.1111/opo.12350. _eprint: https://onlinelibrary.wiley.com/doi/pdf/10.1111/opo.12350.

[35] E. M. Dykens, E. Roof, H. Hunt-Hawkins, N. Dankner, E. B. Lee, C. M. Shivers, C. Daniell, and S.-J. Kim. Diagnoses and characteristics of autism spectrum disorders in children with Prader-Willi syndrome. *Journal of Neurodevelopmental Disorders*, 9(1):18, June 2017. ISSN 1866-1955. doi: 10.1186/s11689-017-9200-2. URL https://doi.org/10.1186/s11689-017-9200-2.

[36] S. Eijk, S. E. Mous, G. C. Dieleman, B. Dierckx, A. B. Rietman, P. F. A. de Nijs, L. W. ten Hoopen, R. van Minkelen, Y. Elgersma, C. E. Catsman-Berrevoets, R. Oostenbrink, and J. S. Legerstee. Autism Spectrum Disorder in an Unselected Cohort of Children with Neurofibromatosis Type 1 (NF1). *Journal of Autism and Developmental Disorders*, 48(7):2278–2285, July 2018. ISSN 1573-3432. doi: 10.1007/s10803-018-3478-0. URL https://doi.org/10.1007/s10803-018-3478-0.

[37] E. Fazzi, S. Micheletti, J. Galli, A. Rossi, F. Gitti, and A. Molinaro. Autism in Children With Cerebral and Peripheral Visual Impairment: Fact or Artifact? *Seminars in Pediatric Neurology*, 31: 57–67, Oct. 2019. ISSN 1071-9091. doi: 10.1016/j.spen.2019.05.008. URL https://www.sciencedirect.com/science/article/pii/S1071909119300610.

[38] R. E. Frye, N. Rincon, P. J. McCarty, D. Brister, A. C. Scheck, and D. A. Rossignol. Biomarkers of mitochondrial dysfunction in autism spectrum disorder: A systematic review and meta-analysis. *Neurobiology of Disease*, 197:106520, July 2024. ISSN 0969-9961. doi: 10.1016/j.nbd.2024.106520. URL https://www.sciencedirect.com/science/article/pii/S0969996124001190.

[39] F. Fulceri, M. Morelli, E. Santocchi, H. Cena, T. D. Bianco, A. Narzisi, S. Calderoni, and F. Muratori. Gastrointestinal symptoms and behavioral problems in preschoolers with Autism Spectrum Disorder. *Digestive and Liver Disease*, 48(3):248–254, Mar. 2016. ISSN 1590-8658, 1878-3562. doi: 10.1016/j.dld.2015.11.026. URL https://www.dldjournalonline.com/article/S1590-8658(15)30030-X/fulltext.

[40] D. A. Geier and M. R. Geier. A Longitudinal Cohort Study of Precocious Puberty and Autism Spectrum Disorder. *Hormone Research in Paediatrics*, 94(5-6):219–228, 2021. ISSN 1663-2818, 1663-2826. doi: 10.1159/000519141. URL https://karger.com/article/doi/10.1159/000519141.

[41] M. R. Glans, N. Thelin, M. B. Humble, M. Elwin, and S. Bejerot. The Relationship Between Generalised Joint Hypermobility and Autism Spectrum Disorder in Adults: A Large, Cross-Sectional, Case Control Comparison. *Frontiers in Psychiatry*, 12:803334, Feb. 2022. ISSN 1664-0640. doi: 10.3389/fpsyt.2021.803334. URL https://www.ncbi.nlm.nih.gov/pmc/articles/PMC8861852/.

[42] M. J. Goldenthal, S. Damle, S. Sheth, N. Shah, J. Melvin, R. Jethva, H. Hardison, H. Marks, and A. Legido. Mitochondrial Enzyme Dysfunction in Autism Spectrum Disorders; A Novel Biomarker Revealed From Buccal Swab Analysis. *Biomarkers in Medicine*, 9(10):957–965, Oct. 2015. ISSN 1752-0363. doi: 10.2217/bmm.15.72. URL https://doi.org/10.2217/bmm.15.72. _eprint: https://doi.org/10.2217/bmm.15.72.

[43] T. Gong, C. Lundholm, S. Lundström, R. Kuja-Halkola, M. J. Taylor, and C. Almqvist. Understanding the relationship between asthma and autism spectrum disorder: a population-based family and twin study. *Psychological Medicine*, 53(7):3096–3104, 2023. ISSN 0033-2917. doi: 10.1017/S0033291721005158. URL `https://www.ncbi.nlm.nih.gov/pmc/articles/PMC10235668/`.

[44] S. Gu, A. Katyal, Q. Zhang, W. Chung, S. Franciosi, and S. Sanatani. The Association Between Congenital Heart Disease and Autism Spectrum Disorder: A Systematic Review and Meta-Analysis. *Pediatric Cardiology*, 44(5):1092–1107, June 2023. ISSN 1432-1971. doi: 10.1007/s00246-023-03146-5. URL `https://doi.org/10.1007/s00246-023-03146-5`.

[45] E. Haebig, A. Sterling, A. Barton-Hulsey, and L. Friedman. Rates and predictors of co-occurring autism spectrum disorder in boys with fragile X syndrome. *Autism & Developmental Language Impairments*, 5:2396941520905328, Jan. 2020. ISSN 2396-9415. doi: 10.1177/2396941520905328. URL `https://doi.org/10.1177/2396941520905328`.

[46] B. N. Hand, A. M. Angell, L. Harris, and L. A. Carpenter. Prevalence of physical and mental health conditions in Medicare-enrolled, autistic older adults. *Autism*, 24(3):755–764, Apr. 2020. ISSN 1362-3613. doi: 10.1177/1362361319890793. URL `https://www.ncbi.nlm.nih.gov/pmc/articles/PMC7433648/`.

[47] S. L. Hartley, A. C. Wheeler, M. R. Mailick, M. Raspa, I. Mihaila, E. Bishop, and D. B. Bailey. Autism Symptoms Across Adulthood in Men with Fragile X Syndrome: A Cross-Sectional Analysis. *Journal of Autism and Developmental Disorders*, 45(11):3668–3679, Nov. 2015. ISSN 1573-3432. doi: 10.1007/s10803-015-2513-7. URL `https://doi.org/10.1007/s10803-015-2513-7`.

[48] M. Hasler, U. S. Fideli, A. Susi, and E. Hisle-Gorman. Examining the relationship between autism spectrum disorder and neural tube defects. *Congenital Anomalies*, 63(4):100–108, 2023. ISSN 1741-4520. doi: 10.1111/cga.12516. URL `https://onlinelibrary.wiley.com/doi/abs/10.1111/cga.12516`. _eprint: https://onlinelibrary.wiley.com/doi/pdf/10.1111/cga.12516.

[49] H. Holmes, F. Sawer, and M. Clark. Autism spectrum disorders and epilepsy in children: A commentary on the occurrence of autism in epilepsy; how it can present differently and the challenges associated with diagnosis. *Epilepsy & Behavior*, 117:107813, Apr. 2021. ISSN 1525-5050. doi: 10.1016/j.yebeh.2021.107813. URL `https://www.sciencedirect.com/science/article/pii/S1525505021000470`.

[50] R. Houghton, R. C. Ong, and F. Bolognani. Psychiatric comorbidities and use of psychotropic medications in people with autism

spectrum disorder in the United States. *Autism Research*, 10(12): 2037–2047, 2017. ISSN 1939-3806. doi: 10.1002/aur.1848. URL https://onlinelibrary.wiley.com/doi/abs/10.1002/aur.1848. _eprint: https://onlinelibrary.wiley.com/doi/pdf/10.1002/aur.1848.

[51] J.-S. Huang, F.-C. Yang, W.-C. Chien, T.-C. Yeh, C.-H. Chung, C.-K. Tsai, S.-J. Tsai, S.-S. Yang, N.-S. Tzeng, M.-H. Chen, and C.-S. Liang. Risk of Substance Use Disorder and Its Associations With Comorbidities and Psychotropic Agents in Patients With Autism. *JAMA Pediatrics*, 175(2):e205371, Feb. 2021. ISSN 2168-6203. doi: 10.1001/jamapediatrics.2020.5371. URL https://pmc.ncbi.nlm.nih.gov/articles/PMC7783585/.

[52] M. M. Hughes, K. A. Shaw, M. E. Patrick, M. DiRienzo, A. V. Bakian, D. A. Bilder, M. S. Durkin, A. Hudson, M. H. Spivey, L. S. DaWalt, A. Salinas, Y. D. Schwenk, M. Lopez, T. M. Baroud, and M. J. Maenner. Adolescents With Autism Spectrum Disorder: Diagnostic Patterns, Co-occurring Conditions, and Transition Planning. *Journal of Adolescent Health*, 73(2):271–278, Aug. 2023. ISSN 1054-139X. doi: 10.1016/j.jadohealth.2022.12.010. URL https://www.sciencedirect.com/science/article/pii/S1054139X23000010.

[53] M. Hunt, J. F. G. Underwood, L. Hubbard, and J. Hall. Risk of physical health comorbidities in autistic adults: clinical nested cross-sectional study. *BJPsych Open*, 10(6):e182, Nov. 2024. ISSN 2056-4724. doi: 10.1192/bjo.2024.777. URL https://www.cambridge.org/core/journals/bjpsych-open/article/risk-of-physical-health-co[illegible]

[54] I. Ivanović. Psychiatric Comorbidities in Children With ASD: Autism Centre Experience. *Frontiers in Psychiatry*, 12, June 2021. ISSN 1664-0640. doi: 10.3389/fpsyt.2021.673169. URL https://www.frontiersin.org/journals/psychiatry/articles/10.3389/fpsyt.2021.673169/full[illegible]

[55] N. Jadav and V. H. Bal. Associations between co-occurring conditions and age of autism diagnosis: Implications for mental health training and adult autism research. *Autism Research*, 15(11): 2112–2125, 2022. ISSN 1939-3806. doi: 10.1002/aur.2808. URL https://onlinelibrary.wiley.com/doi/abs/10.1002/aur.2808. _eprint: https://onlinelibrary.wiley.com/doi/pdf/10.1002/aur.2808.

[56] E. Jenabi, S. Bashirian, F. Fariba, and B. Naghshtabrizi. The association between congenital heart disease and the risk of Autism spectrum disorders or attention-deficit/hyperactivity disorder among children: a meta-analysis. *The European Journal of Psychiatry*, 36(2): 71–76, Apr. 2022. ISSN 0213-6163. doi: 10.1016/j.ejpsy.2021.10.001. URL https://www.sciencedirect.com/science/article/pii/S0213616321000604.

[57] M. Junaid, L. Slack-Smith, K. Wong, J. Bourke, G. Baynam, H. Calache, and H. Leonard. Association between craniofacial anomalies, intellectual disability and autism spectrum disorder: Western Australian population-based study. *Pediatric Research*, 92(6):1795–1804, Dec. 2022. ISSN 1530-0447. doi: 10.1038/s41390-022-02024-9. URL `https://www.nature.com/articles/s41390-022-02024-9`.

[58] T. H. Kaas, R. K. Vinding, J. Stokholm, K. Bønnelykke, H. Bisgaard, and B. L. Chawes. Association between childhood asthma and attention deficit hyperactivity or autism spectrum disorders: A systematic review with meta-analysis. *Clinical and Experimental Allergy: Journal of the British Society for Allergy and Clinical Immunology*, 51(2):228–252, Feb. 2021. ISSN 1365-2222. doi: 10.1111/cea.13750.

[59] S. Kamionkowski, F. Shibli, S. Ganocy, and R. Fass. The relationship between gastroesophageal reflux disease and autism spectrum disorder in adult patients in the United States. *Neurogastroenterology & Motility*, 34(7):e14295, 2022. ISSN 1365-2982. doi: 10.1111/nmo.14295. URL `https://onlinelibrary.wiley.com/doi/abs/10.1111/nmo.14295`. _eprint: https://onlinelibrary.wiley.com/doi/pdf/10.1111/nmo.14295.

[60] A.-M. Kassa, C. A. Håkanson, and H. E. Lilja. The risk of autism spectrum disorder and intellectual disability but not attention deficit/hyperactivity disorder is increased in individuals with esophageal atresia. *Diseases of the Esophagus*, 36(7):doac097, Dec. 2022. ISSN 1120-8694. doi: 10.1093/dote/doac097. URL `https://www.ncbi.nlm.nih.gov/pmc/articles/PMC10317004/`.

[61] W. E. Kaufmann, S. A. Kidd, H. F. Andrews, D. B. Budimirovic, A. Esler, B. Haas-Givler, T. Stackhouse, C. Riley, G. Peacock, S. L. Sherman, W. T. Brown, and E. Berry-Kravis. Autism Spectrum Disorder in Fragile X Syndrome: Cooccurring Conditions and Current Treatment. *Pediatrics*, 139(Suppl 3):S194–S206, June 2017. ISSN 0031-4005. doi: 10.1542/peds.2016-1159F. URL `https://www.ncbi.nlm.nih.gov/pmc/articles/PMC5619699/`.

[62] C. H. Kennedy, T. B. Huedo-Medina, J. Twachtman-Bassett, L. Kalsner, R. Areny-Joval, and I.-M. Eigsti. Health Condition Co-Morbidities in Children with Autism and Their Association with Challenging Behavior. *Advances in Neurodevelopmental Disorders*, pages 1–7, 2024. URL `https://link.springer.com/article/10.1007/s41252-024-00395-0`.

[63] V. Khachadourian, B. Mahjani, S. Sandin, A. Kolevzon, J. D. Buxbaum, A. Reichenberg, and M. Janecka. Comorbidities in autism spectrum disorder and their etiologies. *Translational Psychiatry*, 13(1):71, Feb. 2023. ISSN 2158-3188. doi: 10.1038/s41398-023-02374-w. URL `https://www.nature.com/articles/s41398-023-02374-w`.

[64] K. Kielty, K. Luo, M. Siegel, and C. A. Mazefsky. Epilepsy in Autism: Prevalence and Associated Factors in a Large Inpatient Psychiatric Sample. *Journal of Autism and Developmental Disorders*, Nov. 2025. ISSN 1573-3432. doi: 10.1007/s10803-025-07140-z. URL https://doi.org/10.1007/s10803-025-07140-z.

[65] A. Koceski and V. Trajkovski. Health status of people with autism spectrum disorder. *Advances in Autism*, 8(3):252–263, June 2022. ISSN 2056-3868, 2056-3868. doi: 10.1108/AIA-01-2021-0005. URL http://www.emerald.com/aia/article/8/3/252-263/59892.

[66] B. Kutasy, C. Skoglund, A. Löf-Granström, E. Öst, B. Frenckner, and C. Mesas Burgos. Increased risk of clinically relevant neurodevelopmental disorders in survivors of congenital diaphragmatic hernia: a population-based study. *Pediatric Surgery International*, 40(1):304, Nov. 2024. ISSN 1437-9813. doi: 10.1007/s00383-024-05871-1. URL https://doi.org/10.1007/s00383-024-05871-1.

[67] K. Y. C. Lai, P. W. L. Leung, S. F. Hung, C. K. S. Shea, F. Mo, K. K. I. Che, C.-Y. Tse, F. L. F. Lau, S. L. Ma, J. C. Y. Wu, S. So, and M. R. Dadds. Gastrointestinal Problems in Chinese Children with Autism Spectrum Disorder. *Neuropsychiatric Disease and Treatment*, 16: 1807–1815, July 2020. ISSN 1176-6328. doi: 10.2147/NDT.S260654. URL https://www.ncbi.nlm.nih.gov/pmc/articles/PMC7386814/.

[68] I. Lasheras, M. Real-López, and J. Santabárbara. Prevalence of gastrointestinal symptoms in autism spectrum disorder: A meta-analysis. *Anales de Pediatría (English Edition)*, 99(2):102–110, Aug. 2023. ISSN 2341-2879. doi: 10.1016/j.anpede.2023.07.003. URL https://www.sciencedirect.com/science/article/pii/S234128792300159X.

[69] G. Leader, A. Hogan, J. L. Chen, L. Maher, K. Naughton, N. O'Rourke, M. Casburn, and A. Mannion. Age of Autism Spectrum Disorder Diagnosis and Comorbidity in Children and Adolescents with Autism Spectrum Disorder. *Developmental Neurorehabilitation*, 25(1):29–37, Jan. 2022. ISSN 1751-8431. doi: 10.1080/17518423.2021.1917717.

[70] M. Lee, G. E. Martin, E. Berry-Kravis, and M. Losh. A developmental, longitudinal investigation of autism phenotypic profiles in fragile X syndrome. *Journal of Neurodevelopmental Disorders*, 8(1):47, Dec. 2016. ISSN 1866-1955. doi: 10.1186/s11689-016-9179-0. URL https://doi.org/10.1186/s11689-016-9179-0.

[71] A. G. Lever and H. M. Geurts. Psychiatric Co-occurring Symptoms and Disorders in Young, Middle-Aged, and Older Adults with Autism Spectrum Disorder. *Journal of Autism and Developmental Disorders*, 46(6): 1916–1930, June 2016. ISSN 1573-3432. doi: 10.1007/s10803-016-2722-8. URL https://doi.org/10.1007/s10803-016-2722-8.

[72] H. Li, H. Liu, X. Chen, J. Zhang, G. Tong, and Y. Sun. Association of food hypersensitivity in children with the risk of autism spectrum disorder: a meta-analysis. *European Journal of Pediatrics*, 180(4):999–1008, Apr. 2021. ISSN 1432-1076. doi: 10.1007/s00431-020-03826-x. URL https://link-springer-com.mutex.gmu.edu/article/10.1007/s00431-020-03826-x. Company: Springer Distributor: Springer Institution: Springer Label: Springer Number: 4.

[73] Q. Liu, W. Yin, J. J. Meijsen, A. Reichenberg, J. R. Gådin, A. J. Schork, H.-O. Adami, A. Kolevzon, S. Sandin, and F. Fang. Cancer risk in individuals with autism spectrum disorder. *Annals of Oncology*, 33(7):713–719, July 2022. ISSN 0923-7534, 1569-8041. doi: 10.1016/j.annonc.2022.04.006. URL https://www.annalsofoncology.org/article/S0923-7534(22)00690-1/fulltext.

[74] S. Liu, H. Larsson, R. Kuja-Halkola, P. Lichtenstein, A. Butwicka, and M. J. Taylor. Age-related physical health of older autistic adults in Sweden: a longitudinal, retrospective, population-based cohort study. *The Lancet Healthy Longevity*, 4(7):e307–e315, July 2023. ISSN 2666-7568. doi: 10.1016/S2666-7568(23)00067-3. URL https://www.thelancet.com/journals/lanhl/article/PIIS2666-7568(23)00067-3/fulltext.

[75] A. Longo, D. Radford, and B. N. Hand. A US national update of health condition prevalence among privately-insured autistic adults. *Journal of Comparative Effectiveness Research*, 13 (3):e230051, Mar. 2024. doi: 10.57264/cer-2023-0051. URL https://becarispublishing.com/doi/full/10.57264/cer-2023-0051.

[76] S. Lukmanji, S. A. Manji, S. Kadhim, K. M. Sauro, E. C. Wirrell, C.-S. Kwon, and N. Jetté. The co-occurrence of epilepsy and autism: A systematic review. *Epilepsy & Behavior*, 98:238–248, Sept. 2019. ISSN 1525-5050. doi: 10.1016/j.yebeh.2019.07.037. URL https://linkinghub.elsevier.com/retrieve/pii/S1525505019304949.

[77] M. Marlborough, A. Welham, C. Jones, S. Reckless, and J. Moss. Autism spectrum disorder in females with fragile X syndrome: a systematic review and meta-analysis of prevalence. *Journal of Neurodevelopmental Disorders*, 13(1):28, July 2021. ISSN 1866-1955. doi: 10.1186/s11689-021-09362-5. URL https://doi.org/10.1186/s11689-021-09362-5.

[78] T. May, P. D. Pilkington, R. Younan, and K. Williams. Overlap of autism spectrum disorder and borderline personality disorder: A systematic review and meta-analysis. *Autism Research*, 14(12): 2688–2710, 2021. ISSN 1939-3806. doi: 10.1002/aur.2619. URL https://onlinelibrary.wiley.com/doi/abs/10.1002/aur.2619. _eprint: https://onlinelibrary.wiley.com/doi/pdf/10.1002/aur.2619.

[79] M. Micai, L. M. Fatta, L. Gila, A. Caruso, T. Salvitti, F. Fulceri, A. Ciaramella, R. D'Amico, C. Del Giovane, M. Bertelli, G. Romano, H. J.

Schünemann, and M. L. Scattoni. Prevalence of co-occurring conditions in children and adults with autism spectrum disorder: A systematic review and meta-analysis. *Neuroscience & Biobehavioral Reviews*, 155:105436, Dec. 2023. ISSN 0149-7634. doi: 10.1016/j.neubiorev.2023.105436. URL https://www.sciencedirect.com/science/article/pii/S0149763423004050.

[80] R. A. Mitchell, S. M. Barton, A. S. Harvey, A. M. Ure, and K. Williams. Factors associated with autism spectrum disorder in children with tuberous sclerosis complex: a systematic review and meta-analysis. *Developmental Medicine & Child Neurology*, 63(7):791–801, July 2021. ISSN 1469-8749. doi: 10.1111/dmcn.14787. URL https://onlinelibrary.wiley.com/doi/10.1111/dmcn.14787.

[81] C. Miyazaki, M. Koyama, E. Ota, T. Swa, R. M. Amiya, L. B. Mlunde, Y. Tachibana, K. Yamamoto-Hanada, and R. Mori. Allergies in Children with Autism Spectrum Disorder: a Systematic Review and Meta-analysis. *Review Journal of Autism and Developmental Disorders*, 2(4):374–401, Dec. 2015. ISSN 2195-7177, 2195-7185. doi: 10.1007/s40489-015-0059-4. URL http://link.springer.com/10.1007/s40489-015-0059-4.

[82] H. Morotti, S. Mastel, K. Keller, R. A. Barnard, T. Hall, B. J. O'Roak, and E. Fombonne. Autism and attention-deficit/hyperactivity disorders and symptoms in children with neurofibromatosis type 1. *Developmental Medicine & Child Neurology*, 63(2):226–232, 2021. ISSN 1469-8749. doi: 10.1111/dmcn.14558. URL https://onlinelibrary.wiley.com/doi/abs/10.1111/dmcn.14558. _eprint: https://onlinelibrary.wiley.com/doi/pdf/10.1111/dmcn.14558.

[83] J. Niemczyk, C. Wagner, and A. von Gontard. Incontinence in autism spectrum disorder: a systematic review. *European Child & Adolescent Psychiatry*, 27(12):1523–1537, Dec. 2018. ISSN 1435-165X. doi: 10.1007/s00787-017-1062-3. URL https://doi.org/10.1007/s00787-017-1062-3.

[84] E. O'Nions, J. Brown, J. E. J. Buckman, R. Charlton, C. Cooper, C. E. Baou, F. Happé, S. Hoare, D. Lewer, J. Manthorpe, D. G. J. McKechnie, M. Richards, R. Saunders, W. Mandy, and J. Stott. Diagnosis of common health conditions among autistic adults in the UK: evidence from a matched cohort study. *The Lancet Regional Health – Europe*, 41, June 2024. ISSN 2666-7762. doi: 10.1016/j.lanepe.2024.100907. URL https://www.thelancet.com/journals/lanepe/article/PIIS2666-7762(24)00073-5/fulltext.

[85] E. O'Nions, D. Lewer, I. Petersen, J. Brown, J. E. J. Buckman, R. Charlton, C. Cooper, C. E. Baou, F. Happé, J. Manthorpe, D. G. J. McKechnie, M. Richards, R. Saunders, C. Zanker, W. Mandy, and J. Stott. Estimating life expectancy and years of life lost for autistic people in the UK: a matched cohort study. *The Lancet Regional Health – Europe*, 36,

Jan. 2024. ISSN 2666-7762. doi: 10.1016/j.lanepe.2023.100776. URL https://www.thelancet.com/journals/lanepe/article/PIIS2666-7762(23)00195-3/fulltext.

[86] I. Pacheva, I. Ivanov, R. Yordanova, K. Gaberova, F. Galabova, M. Panova, A. Petkova, E. Timova, and I. Sotkova. Epilepsy in Children with Autistic Spectrum Disorder. *Children*, 6(2):15, Jan. 2019. ISSN 2227-9067. doi: 10.3390/children6020015. URL https://www.ncbi.nlm.nih.gov/pmc/articles/PMC6406948/.

[87] P.-Y. Pan, S. Bölte, P. Kaur, S. Jamil, and U. Jonsson. Neurological disorders in autism: A systematic review and meta-analysis. *Autism*, 25 (3):812–830, Apr. 2021. ISSN 1362-3613. doi: 10.1177/1362361320951370. URL https://doi.org/10.1177/1362361320951370.

[88] P. Phunsawat, W. Chiangjong, S. Chutipongtanate, O. Dumrongwongsiri, P. Thommachot, W. Butdawong, and J. Chuthapisith. Folate receptor alpha autoantibodies in children with autism spectrum disorder. *Biomarkers*, 27(8):715–719, Nov. 2022. ISSN 1354-750X. doi: 10.1080/1354750X.2022.2125579. URL https://doi.org/10.1080/1354750X.2022.2125579. _eprint: https://doi.org/10.1080/1354750X.2022.2125579.

[89] E. V. Quadros, J. M. Sequeira, W. T. Brown, C. Mevs, E. Marchi, M. Flory, E. C. Jenkins, M. T. Velinov, and I. L. Cohen. Folate receptor autoantibodies are prevalent in children diagnosed with autism spectrum disorder, their normal siblings and parents. *Autism Research*, 11(5):707–712, 2018. ISSN 1939-3806. doi: 10.1002/aur.1934. URL https://onlinelibrary.wiley.com/doi/abs/10.1002/aur.1934. _eprint: https://onlinelibrary.wiley.com/doi/pdf/10.1002/aur.1934.

[90] V. T. Ramaekers, J. M. Sequeira, M. DiDuca, G. Vrancken, A. Thomas, C. Philippe, M. Peters, A. Jadot, and E. V. Quadros. Improving Outcome in Infantile Autism with Folate Receptor Autoimmunity and Nutritional Derangements: A Self-Controlled Trial. *Autism Research and Treatment*, 2019:7486431, June 2019. ISSN 2090-1925. doi: 10.1155/2019/7486431. URL https://pmc.ncbi.nlm.nih.gov/articles/PMC6604479/.

[91] V. T. Ramaekers, J. M. Sequeira, B. Thöny, and E. V. Quadros. Oxidative Stress, Folate Receptor Autoimmunity, and CSF Findings in Severe Infantile Autism. *Autism Research and Treatment*, 2020(1): 9095284, 2020. ISSN 2090-1933. doi: 10.1155/2020/9095284. URL https://onlinelibrary.wiley.com/doi/abs/10.1155/2020/9095284. _eprint: https://onlinelibrary.wiley.com/doi/pdf/10.1155/2020/9095284.

[92] R. Ramani, B. Fatima, A. Hussain, U. Shahid, A. Kamani, S. M. A. Bakar, H. Naveed, T. Naveed, S. A. Aftab, and A. Z. Abbasi. The clinical interface of tuberous sclerosis complex and autism spectrum disorder: insights and future directions. *Neurological Sciences*, 46(6):2571–2580,

June 2025. ISSN 1590-3478. doi: 10.1007/s10072-025-08065-2. URL https://doi.org/10.1007/s10072-025-08065-2.

[93] J. E. Roberts, J. Bradshaw, E. Will, A. L. Hogan, S. McQuillin, and K. Hills. Emergence and rate of autism in fragile X syndrome across the first years of life. *Development and Psychopathology*, 32(4):1335–1352, Oct. 2020. ISSN 0954-5794, 1469-2198. doi: 10.1017/S0954579420000942.

[94] D. A. Rossignol and R. E. Frye. Cerebral Folate Deficiency, Folate Receptor Alpha Autoantibodies and Leucovorin (Folinic Acid) Treatment in Autism Spectrum Disorders: A Systematic Review and Meta-Analysis. *Journal of Personalized Medicine*, 11(11), Nov. 2021. ISSN 2075-4426. doi: 10.3390/jpm11111141. URL https://www.mdpi.com/2075-4426/11/11/1141. Company: Multidisciplinary Digital Publishing Institute Distributor: Multidisciplinary Digital Publishing Institute Institution: Multidisciplinary Digital Publishing Institute Label: Multidisciplinary Digital Publishing Institute.

[95] E. Rydzewska, L. A. Hughes-McCormack, C. Gillberg, A. Henderson, C. MacIntyre, J. Rintoul, and S.-A. Cooper. Prevalence of long-term health conditions in adults with autism: observational study of a whole country population. *BMJ Open*, 8(8):e023945, Sept. 2018. ISSN 2044-6055. doi: 10.1136/bmjopen-2018-023945. URL https://www.ncbi.nlm.nih.gov/pmc/articles/PMC6120653/.

[96] E. Rødgaard, K. Jensen, K. W. Miskowiak, and L. Mottron. Autism comorbidities show elevated female-to-male odds ratios and are associated with the age of first autism diagnosis. *Acta Psychiatrica Scandinavica*, 144 (5):475–486, Nov. 2021. ISSN 0001690X. doi: 10.1111/acps.13345.

[97] K. Saad, M. Ezzat, A. Elhoufey, M. E. Ahmed, A. A. Al-Atram, M. M. Fageeh, and Y. Hamed. Asthma and Atopic Diseases in Children with Autism Spectrum Disorders. *Journal of Pharmacy and Bioallied Sciences*, 16(Suppl 4):S3209–S3211, Dec. 2024. ISSN 0976-4879. URL https://www.ncbi.nlm.nih.gov/pmc/articles/PMC11805300/.

[98] M. Salehi, S. Jaka, A. Lotfi, A. Ahmad, M. Saeidi, and S. Gunturu. Prevalence, Socio-Demographic Characteristics, and Co-Morbidities of Autism Spectrum Disorder in US Children: Insights from the 2020–2021 National Survey of Children's Health. *Children*, 12(3):297, Feb. 2025. ISSN 2227-9067. doi: 10.3390/children12030297. URL https://www.ncbi.nlm.nih.gov/pmc/articles/PMC11941283/.

[99] D. E. Schendel, M. Overgaard, J. Christensen, L. Hjort, M. Jørgensen, M. Vestergaard, and E. T. Parner. Association of Psychiatric and Neurologic Comorbidity With Mortality Among Persons With Autism Spectrum Disorder in a Danish Population. *JAMA Pediatrics*, 170(3):243–250, Mar. 2016. ISSN 2168-6203. doi: 10.1001/jamapediatrics.2015.3935. URL https://doi.org/10.1001/jamapediatrics.2015.3935.

[100] W. Schott, S. Tao, and L. Shea. Co-occurring conditions and racial-ethnic disparities: Medicaid enrolled adults on the autism spectrum. *Autism Research*, 15(1):70–85, 2022. ISSN 1939-3806. doi: 10.1002/aur.2644. URL https://onlinelibrary.wiley.com/doi/abs/10.1002/aur.2644. _eprint: https://onlinelibrary.wiley.com/doi/pdf/10.1002/aur.2644.

[101] W. Schott, S. Tao, and L. Shea. Covid-19 Risk: Adult Medicaid Beneficiaries with Autism, Intellectual Disability, and Mental Health Conditions. *Autism : the international journal of research and practice*, 26(4):975–987, May 2022. ISSN 1362-3613. doi: 10.1177/13623613211039662. URL https://www.ncbi.nlm.nih.gov/pmc/articles/PMC8859009/.

[102] V. Semovski, C. King, N. Lapshina, and S. L. Stewart. A cross-sectional examination of service complexity in youths with co-occurring autism spectrum disorder and psychiatric or medical diagnoses across service sectors. *Frontiers in Psychology*, 13, Feb. 2023. ISSN 1664-1078. doi: 10.3389/fpsyg.2022.1027373. URL https://www.frontiersin.org/journals/psychology/articles/10.3389/fpsyg.2022.1027373/ful

[103] K. Shedlock, A. Susi, G. H. Gorman, E. Hisle-Gorman, C. R. Erdie-Lalena, and C. M. Nylund. Autism Spectrum Disorders and Metabolic Complications of Obesity. *The Journal of Pediatrics*, 178:183–187.e1, Nov. 2016. ISSN 0022-3476. doi: 10.1016/j.jpeds.2016.07.055. URL https://www.sciencedirect.com/science/article/pii/S0022347616306576.

[104] A. Shoaib, M. S. Cepeda, G. Murray, and R. Ochs-Ross. Autism: Comorbidities and Treatment Patterns in the Real World, a Retrospective Cohort Study Among Children, Adolescents and Adults Newly Diagnosed with Autism. *Journal of Autism and Developmental Disorders*, 52(10):4311–4320, Oct. 2022. ISSN 1573-3432. doi: 10.1007/s10803-021-05289-x. URL https://link-springer-com.mutex.gmu.edu/article/10.1007/s10803-021-05289-x. Company: Springer Distributor: Springer Institution: Springer Label: Springer Number: 10.

[105] E. R. Sigmon, M. Kelleman, A. Susi, C. M. Nylund, and M. E. Oster. Congenital Heart Disease and Autism: A Case-Control Study. *Pediatrics*, 144(5):e20184114, Nov. 2019. ISSN 0031-4005, 1098-4275. doi: 10.1542/peds.2018-4114. URL https://publications.aap.org/pediatrics/article/144/5/e20184114/38231/Congenital-Heart-

[106] G. N. Soke, M. J. Maenner, D. Christensen, M. Kurzius-Spencer, and L. A. Schieve. Prevalence of Co-occurring Medical and Behavioral Conditions/Symptoms Among 4- and 8-Year-Old Children with Autism Spectrum Disorder in Selected Areas of the United States in 2010. *Journal of Autism and Developmental Disorders*, 48(8):2663–2676, Aug. 2018. ISSN 1573-3432. doi: 10.1007/s10803-018-3521-1. URL https://link.springer.com/article/10.1007/s10803-018-3521-1.

Company: Springer Distributor: Springer Institution: Springer Label: Springer Number: 8.

[107] V. Stefanyshyn, R. Stetsyuk, O. Hrebeniuk, G. Ayoub, L. Fishchuk, Z. Rossokha, and N. Gorovenko. Analysis of the Association Between the SLC19A1 Genetic Variant (rs1051266) and Autism Spectrum Disorders, Cerebral Folate Deficiency, and Clinical and Laboratory Parameters. *Journal of Molecular Neuroscience*, 75(2):42, Mar. 2025. ISSN 1559-1166. doi: 10.1007/s12031-025-02338-3. URL https://doi.org/10.1007/s12031-025-02338-3.

[108] H.-C. Steinhausen, M. D. Villumsen, R. K. Støving, and N. Bilenberg. Complete Spectrum of Physical Comorbidities with Autism Spectrum Disorder in a Nationwide Cohort. *Journal of Autism and Developmental Disorders*, pages 1–9, July 2024. ISSN 1573-3432. doi: 10.1007/s10803-024-06476-2. URL https://link-springer-com.mutex.gmu.edu/article/10.1007/s10803-024-06476-2. Company: Springer Distributor: Springer Institution: Springer Label: Springer.

[109] L. Strasser, M. Downes, J. Kung, J. H. Cross, and M. De Haan. Prevalence and risk factors for autism spectrum disorder in epilepsy: a systematic review and meta-analysis. *Developmental Medicine & Child Neurology*, 60(1):19–29, 2018. ISSN 1469-8749. doi: 10.1111/dmcn.13598. URL https://onlinelibrary.wiley.com/doi/abs/10.1111/dmcn.13598. _eprint: https://onlinelibrary.wiley.com/doi/pdf/10.1111/dmcn.13598.

[110] Tamara May, Karen Lee Jing Yi, Kate L. Loveland, Beverley Vollenhoven, and Katrina Williams. Overlap of autism and conditions associated with atypical sex hormone levels or response: A systematic review and meta-analysis - ScienceDirect. *Research in Autism Spectrum Disorders*, 80, Feb. 2021. ISSN 1750-9467. doi: https://doi.org/10.1016/j.rasd.2020.101693.

[111] K. K. Tillman, M. Hakelius, J. Höijer, M. Ramklint, L. Ekselius, D. Nowinski, and F. C. Papadopoulos. Increased Risk for Neurodevelopmental Disorders in Children With Orofacial Clefts. *Journal of the American Academy of Child & Adolescent Psychiatry*, 57(11):876–883, Nov. 2018. ISSN 0890-8567. doi: 10.1016/j.jaac.2018.06.024. URL https://www.sciencedirect.com/science/article/pii/S0890856718313340.

[112] A. Tint, H. K. Brown, S. Chen, M.-C. Lai, L. A. Tarasoff, S. N. Vigod, S. Parish, S. M. Havercamp, and Y. Lunsky. Health Characteristics of Reproductive-Aged Autistic Women in Ontario: A Population-Based, Cross-Sectional Study. *Autism : the international journal of research and practice*, 25(4):1114–1124, May 2021. ISSN 1362-3613. doi: 10.1177/1362361320982819. URL https://www.ncbi.nlm.nih.gov/pmc/articles/PMC8089036/.

[113] A. Tint, H. Chung, M.-C. Lai, R. Balogh, E. Lin, A. Durbin, and Y. Lunsky. Health conditions and service use of autistic women and men: A retrospective population-based case–control study. *Autism*, 27(6):1641–1657, Aug. 2023. ISSN 1362-3613. doi: 10.1177/13623613221144353. URL `https://doi.org/10.1177/13623613221144353`.

[114] A. K. Toman, H. R. AbdulRasool, F. Lami, S. M. Jasim, O. A. Jaber, N. D. Nayeri, M. S. Sabet, and G. Al-Gburi. Exploring co-occurring conditions in Iraqi children with autism spectrum disorder: prevalence, characteristics, and potential risk factors. *Frontiers in Psychiatry*, 16, July 2025. ISSN 1664-0640. doi: 10.3389/fpsyt.2025.1592374. URL `https://www.frontiersin.org/journals/psychiatry/articles/10.3389/fpsyt.2025.1592374/ful`

[115] S. Tromans, G. Yao, R. Alexander, E. Mukaetova-Ladinska, R. Kiani, M. Al-Uzri, V. Chester, R. Carr, Z. Morgan, E. Vounzoulaki, and T. Brugha. The Prevalence of Diabetes in Autistic Persons: A Systematic Review. *Clinical Practice and Epidemiology in Mental Health : CP & EMH*, 16:212–225, Dec. 2020. ISSN 1745-0179. doi: 10.2174/1745017902016010212. URL `https://www.ncbi.nlm.nih.gov/pmc/articles/PMC7931154/`.

[116] S. Trudeau, S. Anne, T. Otteson, B. Hopkins, R. Georgopoulos, and C. Wentland. Diagnosis and patterns of hearing loss in children with severe developmental delay. *American Journal of Otolaryngology*, 42(3):102923, May 2021. ISSN 0196-0709. doi: 10.1016/j.amjoto.2021.102923. URL `https://www.sciencedirect.com/science/article/pii/S0196070921000247`.

[117] P.-C. Tsao, Y.-S. Lee, M.-J. Jeng, J.-W. Hsu, K.-L. Huang, S.-J. Tsai, M.-H. Chen, W.-J. Soong, and Y. R. Kou. Additive effect of congenital heart disease and early developmental disorders on attention-deficit/hyperactivity disorder and autism spectrum disorder: a nationwide population-based longitudinal study. *European Child & Adolescent Psychiatry*, 26(11):1351–1359, Nov. 2017. ISSN 1435-165X. doi: 10.1007/s00787-017-0989-8. URL `https://doi.org/10.1007/s00787-017-0989-8`.

[118] J. F. G. Underwood, M. DelPozo-Banos, A. Frizzati, D. Rai, A. John, and J. Hall. Neurological and psychiatric disorders among autistic adults: a population healthcare record study. *Psychological Medicine*, 53(12):5663–5673, Oct. 2022. ISSN 0033-2917. doi: 10.1017/S0033291722002884. URL `https://www.ncbi.nlm.nih.gov/pmc/articles/PMC10482712/`.

[119] A. Valiente-Pallejà, H. Torrell, G. Muntané, M. J. Cortés, R. Martínez-Leal, N. Abasolo, Y. Alonso, E. Vilella, and L. Martorell. Genetic and clinical evidence of mitochondrial dysfunction in autism spectrum disorder and intellectual disability | Human Molecular Genetics | Oxford Academic. *Human Molecular Genetics*, 27(5):891–900, Mar. 2018. doi: https://doi.org/10.1093/hmg/ddy009.

[120] N. Varga, K. Pentelényi, P. Balicza, A. Gézsi, V. Reményi, V. Hársfalvi, R. Bencsik, A. Illés, C. Prekop, and M. J. Molnár. Mitochondrial dysfunction and autism: comprehensive genetic analyses of children with autism and mtDNA deletion. *Behavioral and Brain Functions*, 14(1): 4, Feb. 2018. ISSN 1744-9081. doi: 10.1186/s12993-018-0135-x. URL https://doi.org/10.1186/s12993-018-0135-x.

[121] T. Vargason, D. L. McGuinness, and J. Hahn. Gastrointestinal Symptoms and Oral Antibiotic Use in Children with Autism Spectrum Disorder: Retrospective Analysis of a Privately Insured U.S. Population. *Journal of autism and developmental disorders*, 49(2):647–659, Feb. 2019. ISSN 0162-3257. doi: 10.1007/s10803-018-3743-2. URL https://www.ncbi.nlm.nih.gov/pmc/articles/PMC6365214/.

[122] L. Vetri. Autism and Migraine: An Unexplored Association? *Brain Sciences*, 10(9):615, Sept. 2020. ISSN 2076-3425. doi: 10.3390/brainsci10090615. URL https://www.mdpi.com/2076-3425/10/9/615. Number: 9.

[123] R. Vohra, S. Madhavan, and U. Sambamoorthi. Comorbidity prevalence, healthcare utilization, and expenditures of Medicaid enrolled adults with autism spectrum disorders. *Autism : the international journal of research and practice*, 21(8):995–1009, Nov. 2017. ISSN 1362-3613. doi: 10.1177/1362361316665222. URL https://www.ncbi.nlm.nih.gov/pmc/articles/PMC5517354/.

[124] J. Wang, B. Ma, J. Wang, Z. Zhang, and O. Chen. Global prevalence of autism spectrum disorder and its gastrointestinal symptoms: A systematic review and meta-analysis. *Frontiers in Psychiatry*, 13:963102, 2022.

[125] L. Wang, W. Shen, H. Yao, R. Zheng, W. Chen, and W. Zhang. Association between Autism Spectrum Disorder and Food Allergy: A Systematic Review and Meta-analysis. *Autism Research*, 14(1): 220–230, 2021. ISSN 1939-3806. doi: 10.1002/aur.2454. URL https://onlinelibrary.wiley.com/doi/abs/10.1002/aur.2454. _eprint: https://onlinelibrary.wiley.com/doi/pdf/10.1002/aur.2454.

[126] Y. Wang, M. Picard, and Z. Gu. Genetic Evidence for Elevated Pathogenicity of Mitochondrial DNA Heteroplasmy in Autism Spectrum Disorder. *PLoS Genetics*, 12(10):e1006391, Oct. 2016. ISSN 1553-7390. doi: 10.1371/journal.pgen.1006391. URL https://pmc.ncbi.nlm.nih.gov/articles/PMC5085253/.

[127] J. H. Ward, E. Weir, C. Allison, and S. Baron-Cohen. Increased rates of chronic physical health conditions across all organ systems in autistic adolescents and adults. *Molecular Autism*, 14:35, Sept. 2023. ISSN 2040-2392. doi: 10.1186/s13229-023-00565-2. URL https://www.ncbi.nlm.nih.gov/pmc/articles/PMC10510241/.

[128] E. Weir, C. Allison, V. Warrier, and S. Baron-Cohen. Increased prevalence of non-communicable physical health conditions among autistic adults. *Autism*, 25(3):681–694, Apr. 2021. ISSN 1362-3613. doi: 10.1177/1362361320953652. URL https://doi.org/10.1177/1362361320953652.

[129] J. A. Weiss, B. Isaacs, H. Diepstra, A. S. Wilton, H. K. Brown, C. McGarry, and Y. Lunsky. Health Concerns and Health Service Utilization in a Population Cohort of Young Adults with Autism Spectrum Disorder. *Journal of Autism and Developmental Disorders*, 48(1):36–44, 2018. ISSN 0162-3257. doi: 10.1007/s10803-017-3292-0. URL https://www.ncbi.nlm.nih.gov/pmc/articles/PMC5760600/.

[130] A. C. Wheeler, J. Mussey, A. Villagomez, E. Bishop, M. Raspa, A. Edwards, J. Bodfish, C. Bann, and D. B. Bailey. DSM-5 Changes and the Prevalence of Parent-Reported Autism Spectrum Symptoms in Fragile X Syndrome. *Journal of Autism and Developmental Disorders*, 45(3):816–829, Mar. 2015. ISSN 1573-3432. doi: 10.1007/s10803-014-2246-z. URL https://doi.org/10.1007/s10803-014-2246-z.

[131] G. Xu, L. G. Snetselaar, J. Jing, B. Liu, L. Strathearn, and W. Bao. Association of Food Allergy and Other Allergic Conditions With Autism Spectrum Disorder in Children. *JAMA Netw Open*, 1(2):e180279, June 2018. ISSN 2574-3805. doi: 10.1001/jamanetworkopen.2018.0279. URL https://www.ncbi.nlm.nih.gov/pubmed/30646068. Edition: 20180601.

[132] O. Zerbo, A. Leong, L. Barcellos, P. Bernal, B. Fireman, and L. A. Croen. Immune Mediated Conditions in Autism Spectrum Disorders. *Brain, behavior, and immunity*, 46:232–236, May 2015. ISSN 0889-1591. doi: 10.1016/j.bbi.2015.02.001. URL https://www.ncbi.nlm.nih.gov/pmc/articles/PMC4414798/.

[133] A. Zhang, J. Li, Y. Zhang, X. Jin, and J. Ma. Epilepsy and Autism Spectrum Disorder: An Epidemiological Study in Shanghai, China. *Frontiers in Psychiatry*, 10, Sept. 2019. ISSN 1664-0640. doi: 10.3389/fpsyt.2019.00658. URL https://www.frontiersin.org/journals/psychiatry/articles/10.3389/fpsyt.2019.00658/full.

[134] J. Zhou, A. Liu, F. He, Y. Jin, S. Zhou, R. Xu, H. Guo, W. Zhou, Q. Wei, and M. Wang. High prevalence of serum folate receptor autoantibodies in children with autism spectrum disorders. *Biomarkers*, 23(7):622–624, Oct. 2018. ISSN 1354-750X. doi: 10.1080/1354750X.2018.1458152. URL https://doi.org/10.1080/1354750X.2018.1458152. _eprint: https://doi.org/10.1080/1354750X.2018.1458152.
