# Supplementary material for: Somatic, Psychiatric and Neurodevelopmental Comorbidities in People with Autism Spectrum Disorder (ASD): A Narrative Review
Source: Healthcare (Basel). 2026 Aug 11;14(16):2492. doi: 10.3390/healthcare14162492 (PMC13512144; doi:10.3390/healthcare14162492)
Supplement: Supplementary file 1 [file healthcare-14-02492-s001.zip › 100e Suppl S2 Comorbidity_Papers___Psychiatric___Clean.pdf]

Supplement S2: Psychiatric

1 Psychiatric comorbidity

1.1 Anxiety Disorders

Table 1: Prevalence of anxiety in those with ASD, table 1.

Source	Sample	Prevalence	Other Notes
Croen et al. (2015) [23]	n=1507 (ASD), n=15070 (age, sex matches controls); Adults; Kaiser Permanente Northern California; 2008-12	Anxiety disorder: 29.13% (ASD), 9.10% (non-ASD)	Chi-square p value: <0.0001
Lever and Geurts (2016) [68]	n=138 (ASD), n=170 (non-ASD); Age: 19-79; Mental Health Institutions and online recruitment; Netherlands	Any anxiety disorder: 53.6% (ASD), 14.7% (non-ASD); Panic Disorder: 15.2% (ASD), 3.5% (non-ASD); Agoraphobia: 21% (ASD), 3.5% (non-ASD); Social Phobia: 15.2% (ASD), 4.7% (non-ASD); Specific Phobia: 11.6% (ASD), 4.7% (non-ASD); Generalized Anxiety Disorder: 15.9% (ASD), 2.9% (non-ASD)	P-value:≤0.001, ≤0.001, ≤0.001, ≤0.01, ≤0.05, ≤0.001
Vohra et al. (2017) [123]	n=1772 (ASD), n=5316 (non-ASD); Age: 22-64; three state Medicaid Analytic eXtract; 2000-08	Anxiety: 12.2% (ASD), 5.6% (TD)	aOR (95% CI): 2.34 (1.95, 2.81), p:<0.001
Houghton et al. (2017) [44]	n=46943 (ASD, commercial), Truven Health MarketScan Commercial Database, 2014; n=46696 (ASD, Medicaid), Truven Health MarketScan Multi-State Medicaid Database; Age: >3	Anxiety: 24.74% (commercial), 13.98% (medicaid)	
Brookman-Frazee et al. (2018) [16]	n=201 (ASD); Age: 4-14; data from community effectiveness trial; Southern California; 2012-15; MINI-KID-P	Any anxiety disorder: 56% (ASD); Specific phobia: 33%, Social phobia: 24%, Seperation anxiety disorder: 15%, Generalized anxiety disorder: 15%, Agoraphobia: 10%, OCD: 10%, Lifetime panic disorder: 10%	

Table 2: Prevalence of anxiety in those with ASD, table 2.

**Source**	**Sample**	**Prevalence**	**Other Notes**
Hollocks et al. (2019) [41]	Review, N=12	Social anxiety: 29% (current), 20% (lifetime)	
Hollocks et al. (2019) [41]	Review, N=7	Generalized anxiety disorder: 18% (current), 26% (lifetime)	
Hollocks et al. (2019) [41]	Review, N=8	Panic/agoraphobia: 15% (current), 18% (lifetime)	
Hollocks et al. (2019) [41]	Review, N=4	Specific phobia: 6% (current), 31% (lifetime)	
Hollocks et al. (2019) [41]	Review, N=1	Seperation anxiety: 3% (current), 21% (lifetime)	
Davignon, et al. (2018) [25]	n=4123 (ASD), n=20615 (non-ASD, non-ADHD); Age: 14-25; Kaiser Permanente Northern California, 2013-15	aOR (95% CI): 4.01 (3.57-4.50)	
Soke et al. (2018) [108]	n=783 (ASD, age: 4), n=1091 (ASD, age; 8), ADDMN - 5 sites (Arizona, Missouri, New Jersey, Utah, and Wisconsin), 2010	Anxiety: 4.73% (ASD, age: 4), 11.18% (ASD, age: 8)	Adjusted prevalence, 95% CI: 2.28 (1.57, 3.39) [8 vs 4 years] - adjusted for sex, ethnicity, maternal education, location
Hollocks et al. (2019) [41]	Review, N=30, n=26070	Pooled anxiety disorder: 27% (current), 42% (lifetime)	
Lai, et al. (2019) [65]	Meta-analysis, N=96	20%	
Margari et al. (2019) [77]	n=100 (ASD, male), n=59 (ASD, female)	Anxiety disorders: 18% (ASD, male), 25.4% (ASD, female)	p: 0.265
Mosner et al. (2019) [84]	n=67 (ASD); Age: 8-25; UNC Autism Research Registry; MINI-KID-P assessment and caregiver report	Anxiety: 34.3%, 25.7% (caregiver); Panic disorder: 2.9%, Agoraphobia: 2.9%, Separation anxiety: 2.9%, Social phobia: 17.1% , Specific phobia: 2.9%, GAD: 17.1%	

Table 3: Prevalence of anxiety in those with ASD, table 3.

Source	Sample	Prevalence	Other Notes
Hossain et al. (2020) [43]	Umbrella Review, N=8	Anxiety disorders: 1.47% - 54%	
Hand et al. (2020) [34]	n=4685 (ASD), n=46850 (non-ASD); Age: ≥65; Medicare; 2016-17	Anxiety disorders: 37.2% (ASD), 8.8% (non-ASD)	aOR: 6.2 (5.8-6.7)
Kirsch et al. (2020) [60]	n=1014 (ASD), n=2028 (non-ASD); Age: ≤30; USA; cohort study	Anxiety: 50.0% (ASD), 22.2% (non-ASD)	(HR, 3.45; 95% CI, 2.96-4.01), p-value:<0.001
Bowden et al. (2020) [15]	n=8955 (ASD), n=1551342 (non-ASD); Age: 0-24; IDI database, New Zealand	Anxiety: 17.1% (ASD), 1.8% (non-ASD)	aOR: 10.2 (95% CI: 9.8-10.6) - standardized for age, sex, ethnicity, deprivation, and urban/rural
Uljarević et al. (2020) [117]	n=255 (ASD), [n=77 (adolescence, age 15-19), n=97 (early adulthood, 20-39), n=70 (middle adulthood, 40-64), n=11 (older adulthood, 65+)]; Australia	Anxiety: 38.4% (ASD, all); 37.7% (ASD, age:15-19), 40.2% (age: 20-39), 38.6% (age: 40-64), 18.2% (age: 65+)	
Huang et al. (2021) [46]	n=6599 (ASD), n=26396 (non-ASD); controls age, sex, date matched 1:4; Taiwan National Health Insurance Research Database; ICD-9; 2000-15	Anxiety disorder: 30.3% (ASD), 5.1% (non-ASD)	p: <0.01
Bougeard et al. (2021) [14]	Review, N=13	Anxiety, Range: 0% - 82.20%	
Ivanović (2021) [51]	n=152 (ASD); Mean age: 8.02 (4.26); Autism Center, Montenegro	Anxiety: 7.89%	
Rødgaard et al. (2021) [100]	n=11,914 (ASD, men), n=4212 (ASD, women); Age ≤16; Danish National Patient Registry	Anxiety disorders: 10% (men with ASD), 19% (women with ASD)	
Brooks et al. (2021) [17]	n=1062 (ASD), n=79175 Age: 1-24; Canada; Electronic Medical Record Primary Care database	Anxiety, adjustment disorders: 2.9% (ASD), 1.3% (non-ASD)	Prevalence ratio: 2.30 (95% CI: 1.62, 3.26) - adjusted for age, sex, neighborhood income, geographic location

Table 4: Prevalence of anxiety in those with ASD, table 4.

Source	Sample	Prevalence	Other Notes
Angell et al. (2021) [5]	n=18355 (ASD, girls), n=65145 (ASD, boys); Age: 1-21; healthcare claims data; integrates ICD-9 and ICD-10 codes	Anxiety disorders: 31.95% (ASD, girls), 28.16% (ASD, boys)	aOR: 1.18 (95% CI: 1.14-1.23), p:<0.001 - adjusted for age, race, ethnicity, urbanicity, and insurance
Yule (2021) [4]	n=206 (ASD), n=156 (ADHD), n=156 (controls, age matched); age: $\geq$ 12; 2007-16; high functioning ASD - academic center	2+ anxiety disorders: 70% (ASD), 42% (ADHD), 12% (control)	p: <0.001 (ASD vs ADHD), p: <0.05 (ASD vs controls), p: <0.001 (ADHD vs controls)
Shoaib et al. (2022) [107]	n=29694 (ASD, medicaid), IBM Marketscan Multi-State Medicaid Database; n=6719 (ASD, Optum), Optum De-Identified Clinformatics Data Mart Database, Date of Death; US; 2015-16	Anxiety: 24.84% (Optum), 16.12% (Medicaid)	
Shoaib et al. (2022) [107]	n=29694 (ASD, medicaid), IBM Marketscan Multi-State Medicaid Database; n=6719 (ASD, Optum), Optum De-Identified Clinformatics Data Mart Database, Date of Death; US; 2015-16	Anxiety: 24.84% (Optum), 16.12% (Medicaid)	
Martini et al. (2022) [79]	n=20841 (ASD) [n=13712 (male, ASD), n=7129 (female, ASD)], n=1314912 (non-ASD) [n=671727 (male, non-ASD), n=643185 (female, non-ASD)]; Age: 16-24; Multiple national registers; Sweden; 2001-13	Anxiety disorders: 20.6% (ASD, total), 3.9% (non-ASD, total); 14.7% (ASD, male), 31.9% (ASD, female), 2.5% (non-ASD, male), 5.4% (non-ASD, female)	HR: 0.95 (95% CI: 0.81-1.10), p:0.47 [Male vs Female] - adjusted for birth year ADHD, ID
Jadav and Bal (2022) [52]	n=4657 (ASD); Adults; online survey, SPARK; 2022	65.8% (any form), 55.5% (any non-social or separation anxiety), 36.5% (social), 11.3% (separation)	

Table 5: Prevalence of anxiety in those with ASD, table 5.

Source	Sample	Prevalence	Other Notes
Mutluer et al. (2022) [85]	Review, N=12, population and EHR studies (age 6-18)	Pooled anxiety prevalence: 11.1%	
Schott et al. (2022) [105]	n=155,617 (ASD), n=466,851 (non-ASD); Adults; Medicaid; 2008–12	24.85% (ASD), 14.66% (non-ASD)	aOR: 1.71 (1.68 1.74)
Underwood et al. (2022) [118]	n=7943 (core ASD), n=25941 (control); Age: ≥18; UK; SAIL Databank, 2001-16	22.40% (ASD), 13.6% (non-ASD)	
Hughes et al. (2023) [48]	n=1846 (ASD); ≤16; ADDM Network (5 sites, USA); 2018	Anxiety: 16%	
Micai et al. (2023) [82]	Meta-analysis of observational studies, N=60 [N=48 (clinical studies from community samples), N=12 (population/registry based)], n=45,978	Pooled prevalence: 35%, Range: 30% - 39%	
Rim et al. (2023) [95]	n=381 (ASD), n=6179 (non-ASD); Age: 0-19; National Health Insurance System National Sample Cohort; South Korea; 2002–13	Anxiety and stress: 7.81% (ASD), 1.08% (non-ASD)	aOR: 6.22 (4.76,8.12)
Semovski et al. (2023) [106]	n=2020 (ASD); Age: 12-18; records + interviews; Canada	Anxiety disorders: 42.9% (ASD)	
Khachadourian et al. (2023) [58]	n=42569 (ASD), n=11389 (non-ASD siblings); SPARK; Age: <18 at time of registration; 1999-2019	Anxiety disorder, estimated: 17% (ASD), 7% (non-ASD); Separation anxiety, estimated: 6% (ASD), 2% (non-ASD); Social anxiety/phobia, estimated: 8% (ASD), 3% (non-ASD)	p: <0.05 for all
Longo et al. (2024) [71]	n=30258 (ASD), n=60516 (non-ASD); Age: 18-64; IBM Market Scan Commercial Claims and Encounters Database; 2019-20	Anxiety Disorders: 55.0% (ASD), 17.5% (non-ASD)	aOR: 5.9 (95% CI: 5.7, 6.1)

Table 6: Prevalence of anxiety in those with ASD, table 6.

Source	Sample	Prevalence	Other Notes
O'Nions et al. (2024) [88]	n=15675 (ASD, no ID), n=156750 (comparison), n=6437 (ASD+ID), n=64370 (comparison); Age: ≥18; UK; IQVIA Medical research data (electronic health records from multiple hospitals); comparison matched by age, sex, location	Anxiety: 20.86% (ASD, no ID), 6.11% (comparison), 13.34% (ASD+ID), 6.96% (comparison) [baseline]; crude incidence based on observation period: 195.89(182.05-210.50) (ASD, no ID), 102.08 (99.28-104.93) (comparison), 107.36(94.51-121.46) (ASD+ID), 92.53(88.83-96.35) (comparison)	p:<0.001 (ASD no ID vs comparison), p:<0.001 (ASD+ID vs comparison) [baseline]; ASD+ID vs comparison, incidence rate ratio: 1.14(1.00-1.30) (ASD+ID), 1.91(1.76-2.06) (ASD no ID)
Al-Ayadhi et al. (2025) [3]	n=363 [n=189 (ASD), n=174 (non-ASD)]; Online questionnaire (self or caregiver); Saudi Arabia; August 2022 - January 2023	32.3% (ASD), 5.7% (non-ASD)	p: 0.000
Salehi et al. (2025) [102]	n=6870 (ASD), n=2686160 (non-ASD); Age: 15-44; Administrative health data, Canada, 2017-18	45.7%	OR: 0.35, p:<0.001 [Female vs Male]
Diemer et al. (2025) [27]	n=1424 (ASD); Adult; SPARK	Anxiety: 69.87% (total), 68.16% (diagnosed <18), 70.74% (diagnosed >18)	p-value 0.314 (between age groups)
Lacroix et al. (2025) [64]	Narrative and Quantitative Review; N =12 , n= 52937 (ASD, female), n= 149478 (ASD, male); excluded papers with less than 12.5% females	Anxiety disorders, Pooled prevalence [95% CI]: 40.4% [31.4-50.2] (ASD, female), 29.7% [22.1-38.6] (ASD, male)	p:<0.001, OR: 1.63

1.1.1 OCD

Table 7: Prevalence of OCD in those with ASD, table 1.

Source	Sample	Prevalence	Other Notes
Croen et al. (2015) [23]	n=1507 (ASD), n=15070 (age, sex matches controls); Adults; Kaiser Permanente Northern California; 2008-12	7.63% (ASD), 0.49% (non-ASD)	Chi-square p value: <0.0001
Lever and Geurts (2016) [68]	n=138 (ASD), n=170 (non-ASD); Age: 19-79; Mental Health Institutions and online recruitment; Netherlands	OCD: 21.7% (ASD), 0.6% (non-ASD); Generalized Anxiety Disorder: 15.9% (ASD), 2.9% (non-ASD)	P-value:$\leq$0.001
Davignon, et al. (2018) [25]	n=4123 (ASD), n=20615 (non-ASD, non-ADHD); Age: 14-25; Kaiser Permanente Northern California, 2013-15	2.3% (ASD), 0.2% (non-ASD)	aOR (95% CI): 10.48 (7.21 - 15.24)
Hollocks et al. (2019) [41]	Review, N=15	24% (current), 22% (lifetime)	
Lai, et al. (2019) [65]	Meta-analysis, N=96	9%	
Margari et al. (2019) [77]	n=100 (ASD, male), n=59 (ASD, female)	OCD: 5% (ASD, male), 0% (ASD, female)	p: 0.081
Mosner et al. (2019) [84]	n=67 (ASD); Age: 8-25; UNC Autism Research Registry; MINI-KID-P assessment and caregiver report	OCD: 11.4% (MINI-KID-P), 17.1% (caregiver report)	
Hossain et al. (2020) [43]	Umbrella Review, N=3	OCD and related disorders: 9%-22%	
Martin et al. (2020) [78]	n=7922 (total), [n=335 (OCD+ASD), n=1010 (OCD), n=6577 (ASD)]; Age: 4-18; South London and Maudsley NHS Foundation Trust; 2007-16	OCD: 5% (ASD)	
Huang et al. (2021) [46]	n=6599 (ASD), n=26396 (non-ASD); controls age, sex, date matched 1:4; Taiwan National Health Insurance Research Database; ICD-9; 2000-15	OCD: 3.1% (ASD), 0.2% (non-ASD)	p: <0.01

Table 8: Prevalence of OCD in those with ASD, table 2.

Source	Sample	Prevalence	Other Notes
Chien et al. (2021) [22]	n=3837 (ASD), n=38370 (non-ASD); Children; Taiwan's claims database; 2000, 2005, 2010	3.9% (ASD), 0.2% (non-ASD)	
Rødgaard et al. (2021) [100]	n=11,914 (ASD, men), n=4212 (ASD, women); Age ≤16; Danish National Patient Registry	OCD: 5% (men with ASD), 8% (women with ASD)	
Martini et al. (2022) [79]	n=20841 (ASD) [n=13712 (male, ASD), n=7129 (female, ASD)], n=1314912 (non-ASD) [n=671727 (male, non-ASD), n=643185 (female, non-ASD)]; Age: 16-24; Multiple national registers; Sweden; 2001-13	OCD: 6.5% (ASD, total), 0.5% (non-ASD, total); 5.9% (ASD, male), 7.8% (ASD, female), 0.3% (non-ASD, male), 0.6% (non-ASD, female)	Male vs female: HR: 0.51 (95% CI: 0..38-0.67), p:<0.001 - adjusted for birth year ADHD, ID
Schott et al. (2022) [105]	n=155,617 (ASD), n=466,851 (non-ASD); Adults; Medicaid; 2008–12	7.94% (ASD), 1.92% (non-ASD)	aOR: 4.17 (4.02 4.33)
Jadav and Bal (2022) [52]	n=4657 (ASD); Adults; online survey, SPARK; 2022	24.2%	
Mutluer et al. (2022) [85]	Review, N=5, population and EHR studies (age 6-18)		
Underwood et al. (2022) [118]	n=7943 (core ASD), n=25941 (control); Age: ≥18; UK; SAIL Databank, 2001-16	3.02% (ASD), 0.33% (non-ASD)	
Hughes et al. (2023) [48]	n=1846 (ASD); ≤16; ADDM Network (5 sites, USA); 2018	OCD: 1%	

Table 9: Prevalence of OCD in those with ASD, table 3.

Source	Sample	Prevalence	Other Notes
Micai et al. (2023) [82]	Meta-analysis of observational studies, N=44 [N=38 (clinical studies from community samples), N=6 (population/registry based)], n=36467	Pooled prevalence: 9%, Range: 7% - 10%	
Khachadourian et al. (2023) [58]	n=42569 (ASD), n=11389 (non-ASD siblings); SPARK; Age: <18 at time of registration; 1999-2019	OCD, estimated: 8% (ASD), 2% (non-ASD)	p: <0.05
Diemer et al. (2025) [27]	n=1424 (ASD); Adult; SPARK	OCD: 25.49% (total), 26.12% (diagnosed <18), 25.19% (diagnosed >18)	p-value 0.701 (between age groups)
Beford et al. (2020) [9]	Narrative review	Lists OCD prevalence: 4% - 71%	
Lacroix et al. (2025) [64]	Narrative and Quantitative Review; N = 5, n= 15090 (ASD, female), n= 30958 (ASD, male); excluded papers with less than 12.5% females	OCD, Pooled prevalence [95% CI]: 12.7% [7.1-21.8] (ASD, female), 10.5% [5.6-18.6] (ASD, male)	p: 0.049, OR: 1.30

1.2 Mood Disorders

Table 10: Prevalence of mood/affective disorders in those with ASD, table 1.

Source	Sample	Prevalence	Other Notes
Lever and Geurts (2016) [68]	n=138 (ASD), n=170 (non-ASD); Age: 19-79; Mental Health Institutions and online recruitment; Netherlands	Any mood disorder: 57.2% (ASD), 18.2% (non-ASD)	p-value: $\leq$0.001
Schendel et al. (2016) [104]	n=20492 (ASD), 1892412 (non-ASD); Age: >1.5; Danish Civil Registration Service and the Danish Medical Birth Register; born 1980-2010	Any mood/affective disorder: 8.8% (ASD), 1.8% (non-ASD)	
Vohra et al. (2017) [123]	n=1772 (ASD), n=5316 (non-ASD); Age: 22-64; three state Medicaid Analytic eXtract; 2000-08	Mood Disorder: 14.4% (ASD), 15.8% (non-ASD)	aOR (95% CI): 0.90 (0.77, 1.05), p: NS
Brookman-Frazee et al. (2018) [16]	n=201 (ASD); Age: 4-14; data from community effectiveness trial; Southern California; 2012-15; MINI-KID-P	Any mood disorder: 30% (ASD)	
Soke et al. (2018) [108]	n=783 (ASD, age: 4), n=1091 (ASD, age; 8), ADDMN - 5 sites (Arizona, Missouri, New Jersey, Utah, and Wisconsin), 2010	Mood disorder: 56.0% (ASD, age: 4), 74.80% (ASD, age: 8)	Adjusted prevalence, 95% CI: 1.25 (1.16, 1.35) [8 vs 4 years] - adjusted for sex, ethnicity, maternal education, location
Bowden et al. (2020) [15]	n=8955 (ASD), n=1551342 (non-ASD); Age: 0-24; IDI database, New Zealand	Any emotional disorder: 27.5% (ASD), 5.1% (non-ASD)	aOR: 5.9 (95% CI: 5.8-6.1) - standardized for age, sex, ethnicity, deprivation, and urban/rural
Hand et al. (2020) [34]	n=4685 (ASD), n=46850 (non-ASD); Age: $\geq$65; Medicare; 2016-17	Mood disorders: 35.9% (ASD), 9.1% (non-ASD)	aOR: 5.6 (5.3-6.0)

Table 11: Prevalence of mood/affective disorders in those with ASD, table 2.

Source	Sample	Prevalence	Other Notes
Huang et al. (2021) [46]	n=6599 (ASD), n=26396 (non-ASD); controls age, sex, date matched 1:4; Taiwan National Health Insurance Research Database; ICD-9; 2000-15	Mood disorders: 24.1% (ASD), 1.4% (non-ASD)	p: <0.01
Tint et al. (2021) [113]	n=6870 (ASD), n=2686160 (non-ASD); Age: 15-44; Administrative health data, Canada, 2017-18	Mood and anxiety disorders: 31.8% (ASD), 13.4% (non-ASD)	Standard difference: 0.45
Brooks et al. (2021) [17]	n=1062 (ASD), n=79175 Age: 1-24; Canada; Electronic Medical Record Primary Care database	Mood/affective disorders: 1.8% (ASD), 0.9% (non-ASD)	Prevalence ratio: 2.15 (95% CI: 1.37, 3.37) - adjusted for age, sex, neighborhood income, geographic location
Rødgaard et al. (2021) [100]	n=11,914 (ASD, men), n=4212 (ASD, women); Age ≤16; Danish National Patient Registry	Affective disorders: 8% (men with ASD), 19% (women with ASD)	.
Angell et al. (2021) [5]	n=18355 (ASD, girls), n=65145 (ASD, boys); Age: 1-21; healthcare claims data; integrates ICD-9 and ICD-10 codes	Mood disorders: 21.45% (ASD, girls), 19.13% (ASD, boys)	aOR: 1.13 (95% CI: 1.08-1.18), p:<0.001 - adjusted for age, race, ethnicity, urbanicity, and insurance
Shoaib et al. (2022) [107]	n=29694 (ASD, medicaid), IBM Marketscan Multi-State Medicaid Database; n=6719 (ASD, Optum), Optum De-Identified Clinformatics Data Mart Database, Date of Death; US; 2015-16	Mood disorder: 17.47% (Optum), 16.56% (Medicaid)	
Micai et al. (2023) [82]	Meta-analysis of observational studies, N=22 [N=15 (clinical studies from community samples), N=7 (population/registry based)], n=16195	Pooled prevalence: 19%, Range: 11% - 28%	
Rim et al. (2023) [95]	n=381 (ASD), n=6179 (non-ASD); Age: 0-19; National Health Insurance System National Sample Cohort; South Korea; 2002–13	9.04% (ASD), 0.59% (non-ASD)	aOR: 13.62 (10.49,17.68)

Table 12: Prevalence of mood/affective disorders in those with ASD, table 3.

Source	Sample	Prevalence	Other Notes
Semovski et al. (2023) [106]	n=2020 (ASD); Age: 12-18; records + interviews; Canada	Mood disorder: 15.1% (ASD)	
Longo et al. (2024) [71]	n=30258 (ASD), n=60516 (non-ASD); Age: 18-64; IBM Market Scan Commercial Claims and Encounters Database; 2019-20	Mood disorders: 42.6% (ASD), 13.2% (non-ASD)	aOR: 4.9 (95% CI: 4.8, 5.1)
Lacroix et al. (2025) [64]	Narrative and Quantitative Review; N =5, n= 40867 (ASD, female), n= 125583 (ASD, male); excluded papers with less than 12.5% females	Mood disorders, Pooled prevalence [95% CI]: 31.1% [22.8-40.8] (ASD, female), 22.1% [13.3-34.4] (ASD, male)	p:0.007 , OR: 1.58

1.2.1 Bipolar

Table 13: Prevalence of bipolar disorder in those with ASD, table 1.

Source	Sample	Prevalence	Other Notes
Croen et al. (2015) [23]	n=1507 (ASD), n=15070 (age, sex matches controls); Adults; Kaiser Permanente Northern California; 2008-12	10.55% (ASD), 1.67% (non-ASD)	Chi-square p value: <0.0001
Houghton et al. (2017) [44]	n=46943 (ASD, commercial), Truven Health MarketScan Commercial Database, 2014; n=46696 (ASD, Medicaid), Truven Health MarketScan Multi-State Medicaid Database; Age: >3	Bipolar: 4.9% (commercial), 7.96% (medicaid)	
Davignon, et al. (2018) [25]	n=4123 (ASD), n=20615 (non-ASD, non-ADHD); Age: 14-25; Kaiser Permanente Northern California, 2013-15	Bipolar disorder: 6.4% (ASD), 0.6% (non-ASD)	aOR (95% CI): 9.58 (7.72 - 11.88)
Lai, et al. (2019) [65]	Meta-analysis, N=96	5%	
Margari et al. (2019) [77]	n=100 (ASD, male), n=59 (ASD, female)	Bipolar: 4% (ASD, male), 3.4% (ASD, female)	p: 0.845
Hossain et al. (2020) [43]	Umbrella Review, N=2	Bipolar: 5% - 21.4%	
Kirsch et al. (2020) [60]	n=1014 (ASD), n=2028 (non-ASD); Age: $\leq$30; USA; cohort study	Bipolar disorder: 7.3% (ASD), 0.9% (non-ASD)	(HR, 9.34; 95% CI, 4.57-19.06), p-value:<0.001
Bowden et al. (2020) [15]	n=8955 (ASD), n=1551342 (non-ASD); Age: 15-24; IDI database, New Zealand	Bipolar: 1.3% (ASD), 0.2% (non-ASD)	aOR: 8.9 (95% CI: 5.9-11.8) - standardized for age, sex, ethnicity, deprivation, and urban/rural

Table 14: Prevalence of bipolar disorder in those with ASD, table 2.

Source	Sample	Prevalence	Other Notes
Yule (2021) [4]	n=230 (ASD), n=219 (ADHD), n=230 (controls, age matched); age: ≥ 12; 2007-16; high functioning ASD - academic center	Bipolar: 22% (ASD), 14% (ADHD), <1% (control)	p: <0.05 (ASD vs ADHD), p: <0.001 (ASD vs controls), p: <0.001 (ADHD vs controls)
Chien et al. (2021) [22]	n=3837 (ASD), n=38370 (non-ASD); Children; Taiwan's claims database; 2000, 2005, 2010	7.0/1,000 person year (ASD), 0.4/1,000 person year (non-ASD)	Crude hazard ratio: 16.8 (13.9–20.2).
Oakley et al. (2021) [87]	Narrative review, N=1	Bipolar: 5% (ASD), <1% (non-ASD)	
Martini et al. (2022) [79]	n=20841 (ASD) [n=13712 (male, ASD), n=7129 (female, ASD)], n=1314912 (non-ASD) [n=671727 (male, non-ASD), n=643185 (female, non-ASD)]; Age: 16-24; Multiple national registers; Sweden; 2001-13	Bipolar disorders: 4.2% (ASD, total), 0.5% (non-ASD, total); 2.8% (ASD, male), 6.9% (ASD, female), 0.3% (non-ASD, male), 0.8% (non-ASD, female)	Male vs female: HR: 0.77 (95% CI: 0.58-1.03), p:0.08 - adjusted for birth year ADHD, ID
Jadav and Bal (2022) [52]	n=4657 (ASD); Adults; online survey, SPARK; 2022	Bipolar: 16.2%	
Mutluer et al. (2022) [85]	Review, N=6, population and EHR studies (age 6-18) (n=91052)	Bipolar: 2%	
Schott et al. (2022) [105]	n=155,617 (ASD), n=466,851 (non-ASD); Adults; Medicaid; 2008–12	25.02% (ASD), 14.83% (non-ASD)	aOR: 1.69 (1.66 1.72)
Varcin et al. (2022) [120]	Meta-analysis of 27 studies, n=31,739	Pooled prevalence: 7.5% (95% CI = 5.79, 9.53)	

Table 15: Prevalence of bipolar disorder in those with ASD, table 4.

Source	Sample	Prevalence	Other Notes
Underwood et al. (2022) [118]	n=7943 (core ASD), n=25941 (control); Age: ≥18; UK; SAIL Databank, 2001-16	2.50% (ASD), 2.00% (non-ASD)	
Micai et al. (2023) [82]	Meta-analysis of observational studies, N=25 [N=19 (clinical studies from community samples), N=6 (population/registry based)], n=108,224	Pooled prevalence: 7%, Range: 4% - 9%	
Khachadourian et al. (2023) [58]	n=42569 (ASD), n=11389 (non-ASD siblings); SPARK; Age: <18 at registration; 1999-2019	Bipolar disorder, estimated: 2% (ASD), 1% (non-ASD)	p: <0.05
Hsu et al. (2024) [45]	n=22698 (ASD), [n=19191 (male), n=3507 (female)]; Age: 2-19; Taiwan's National Health Insurance Research Database; 2001-11; ICD-9	Bipolar disorder: 1.5% (ASD, total); 1.3% (ASD, male), 2.5% (ASD, female)	RR: 0.58 (95% CI: 0.45-0.75) [Male vs female (ref)] - adjusted for age, income, residence, and parental psychiatric disorders. RR: 0.85 (95% CI: 0.60-1.21) [Parental psychiatric disorders - Male vs female (ref)] - adjusted for age, income, residence
Diemer et al. (2025) [27]	n=1424 (ASD); Adult; SPARK	Bipolar: 18.26% (total), 19.59% (diagnosed <18), 17.47% (diagnosed >18)	p-value 0.324 (between age groups)
Lacroix et al. (2025) [64]	Narrative and Quantitative Review; N =7, n= 31423 (ASD, female), n= 86154 (ASD, male); excluded papers with less than 12.5% females	Bipolar disorders, Pooled prevalence [95% CI]: 9.3% [5.9-14.3] (ASD, female), 5.9% [3.4-10.2] (ASD, male)	p: <0.001, OR: 1.62

1.2.2 Depression

Table 16: Prevalence of depression in those with ASD, table 1.

Source	Sample	Prevalence	Other Notes
Croen et al. (2015) [23]	n=1507 (ASD), n=15070 (age, sex matches controls); Adults; Kaiser Permanente Northern California; 2008-12	25.75% (ASD), 9.89% (non-ASD)	Chi-square p value: <0.0001
Greenlee et al. (2016) [32]	n=1272 (ASD); Age: 6-17; Autism Speaks Autism Treatment Network consortium	Depression: 7% (ASD, total); 4.8% (age: 6-12), 20.2% (age: 13-17)	
Lever and Geurts (2016) [68]	n=138 (ASD), n=170 (non-ASD); Age: 19-79; Mental Health Institutions and online recruitment; Netherlands	Depression: 53.6% (ASD), 16.5% (non-ASD); Dysthymia: 18.1% (ASD), 2.9% (non-ASD)	p:≤0.001, p:≤0.001
Houghton et al. (2017) [44]	n=46943 (ASD, commercial), Truven Health MarketScan Commercial Database, 2014; n=46696 (ASD, Medicaid), Truven Health MarketScan Multi-State Medicaid Database; Age: >3	Depression: 10.15% (commercial), 9.19% (medicaid)	
Davignon, et al. (2018) [25]	n=4123 (ASD), n=20615 (non-ASD, non-ADHD); Age: 14-25; Kaiser Permanente Northern California, 2013-15	9.9% (ASD), 4.0% (non-ASD)	aOR (95% CI): 2.51 (2.21 - 2.85)
Brookman-Frazee et al. (2018) [16]	n=201 (ASD); Age: 4-14; data from community effectiveness trial; Southern California; 2012-15; MINI-KID-P	Major depressive disorder (lifetime): 22% (ASD); Dysthymia: 8%	

Table 17: Prevalence of depression in those with ASD, table 2.

Source	Sample	Prevalence	Other Notes
Hudson et al. (2019) [47]	Meta-Analysis, N=66 (lifetime: 44, current: 31)	Depression: 14.1% (95% CI: 10.3-19.8) [lifetime], 12.3% (95% CI: 9.7-15.5%) [current]	
Hollocks et al. (2019) [41]	Review, N=29, n=26117	Pooled estimation: 23% (current), 37% (lifetime)	
Lai, et al. (2019) [65]	Meta-analysis, N=96	Depressive disorders: 11%	
Margari et al. (2019) [77]	n=100 (ASD, male), n=59 (ASD, female)	Depressive disorders: 6% (ASD, male), 13.5% (ASD, female)	p: 0.104
Mosner et al. (2019) [84]	n=67 (ASD); Age: 8-25; UNC Autism Research Registry; MINI-KID-P assessment and caregiver report	Depression: 28.6% (MINI-KID-P), 8.6% (caregiver)	
Hossain et al. (2020) [43]	Umbrella Review, N=6	Depressive disorders: 2.5%-47.1%	
Kirsch et al. (2020) [60]	n=1014 (ASD), n=2028 (non-ASD); Age: $\leq$30; USA; cohort study	Depression: 54.1% (ASD), 28.9% (non-ASD)	(HR, 2.81; 95% CI, 2.45-3.22), p-value:$<$0.001
Bowden et al. (2020) [15]	n=8955 (ASD), n=1551342 (non-ASD); Age: 0-24; IDI database, New Zealand	Depression: 4.8% (ASD), 1.6% (non-ASD)	aOR: 4.0 (95% CI: 3.7-4.4) - standardized for age, sex, ethnicity, deprivation, and urban/rural
Uljarević et al. (2020) [117]	n=255 (ASD), [n=77 (adolescence, age 15-19), n=97 (early adulthood, 20-39), n=70 (middle adulthood, 40-64), n=11 (older adulthood, 65+)]; Australia	Depression: 38.0% (ASD, all); 33.8% (ASD, age:15-19), 36.1% (age: 20-39), 48.6% (age: 40-64), 27.3% (age: 65+)	
Bougeard et al. (2021) [14]	Review: N=12	Range: 0% - 74.80%	

Table 18: Prevalence of depression in those with ASD, table 3.

Source	Sample	Prevalence	Other Notes
Hosozawa et al. (2021) [42]	n=396 (ASD) [n=61 (age 5), n=65 (age 7), n=138 (age 11), n=132 (age 14)], n=11307 (non-ASD); UK Millennium Cohort Study; questionnaire and self-report; born 2000-02	Depression: 16.6% (ASD, diagnosed age 5), 18.2% (ASD, diagnosed age 7), 22.0% (ASD, diagnosed age 11), 32.8% (ASD, diagnosed age 14), 16% (non-ASD)	p: 0.03 (linear trend, unadjusted occurrence); SexaOR, 95% CI: 1 (ref) [no ASD], 1.05 (0.4-2.76) [ASD, diagnosed age 5], 1.17 (0.49-2.78) [ASD, diagnosed age 7], 1.49 (0.91-2.44) [ASD, diagnosed age 11], 2.57 (1.62-4.09) [ASD, diagnosed age 14]
Chien et al. (2021) [22]	n=3837 (ASD), n=38370 (non-ASD); Children; Taiwan's claims database; 2000, 2005, 2010	Major depressive disorder: 3.2/1,000 person year (ASD), 0.4/1,000 person year (non-ASD)	Crude hazard ratio: 7.4 (5.9–9.2)
Ivanović (2021) [51]	n=152 (ASD); Mean age: 8.02 (4.26); Autism Center, Montenegro	1.97%	
Oakley et al. (2021) [87]	Narrative review, N=4	depression: 10%-50% (ASD), 7% (non-ASD)	s
Yule (2021) [4]	n=230 (ASD), n=219 (ADHD), n=230 (controls, age matched); age: ≥ 12; 2007-16; high functioning ASD - academic center	Major depressive disorder: 61% (ASD), 63% (ADHD), 22% (control)	p: ns (ASD vs ADHD), p: <0.001 (ASD vs controls), p: <0.001 (ADHD vs controls)
Martini et al. (2022) [79]	n=20841 (ASD) [n=13712 (male, ASD), n=7129 (female, ASD)], n=1314912 (non-ASD) [n=671727 (male, non-ASD), n=643185 (female, non-ASD)]; Age: 16-24; Multiple national registers; Sweden; 2001-13	depressive disorders: 22.7% (ASD, total), 3.9% (non-ASD, total); 18.4% (ASD, male), 31.0% (ASD, female), 2.6% (non-ASD, male), 5.2% (non-ASD, female)	Male vs female: HR: 0.82 (95% CI: 0.71-0.95), p:0.008 - adjusted for birth year ADHD, ID

Table 19: Prevalence of depression in those with ASD, table 4.

Source	Sample	Prevalence	Other Notes
Jadav and Bal (2022) [52]	n=4657 (ASD); Adults; online survey, SPARK; 2022	52.9%	
Mutluer et al. (2022) [85]	Review, N=6, population and EHR studies (age 6-18)	Pooled prevalence: 2.7%	
Schott et al. (2022) [105]	n=155,617 (ASD), n=466,851 (non-ASD); Adults; Medicaid; 2008–12	27.45% (ASD), 23.33% (non-ASD)	aOR: 1.09 (1.08 1.11)
Underwood et al. (2022) [118]	n=7943 (core ASD), n=25941 (control); Age: ≥18; UK; SAIL Databank, 2001-16	25.90% (ASD), 17.90% (non-ASD)	
Hughes et al. (2023) [48]	n=1846 (ASD); ≤16; ADDM Network (5 sites, USA); 2018	2%	
Micai et al. (2023) [82]	Meta-analysis of observational studies, N=55 [N=46 (clinical studies from community samples), N=9 (population/registry based)], n=41,923	Depressive disorder: Pooled prevalence: 18%, Range: 15% - 21%	
Rim et al. (2023) [95]	n=381 (ASD), n=6179 (non-ASD); Age: 0-19; National Health Insurance System National Sample Cohort; South Korea; 2002–13	5.49% (ASD), 0.45% (non-ASD)	aOR: 10.56 (7.65,14.57)
Khachadourian et al. (2023) [58]	n=42569 (ASD), n=11389 (non-ASD siblings); SPARK; Age: <18 at time of registration; 1999-2019	Depression or dysthymia, estimated: 6% (ASD), 4% (non-ASD)	p: <0.05

Table 20: Prevalence of depression in those with ASD, table 5.

Source	Sample	Prevalence	Other Notes
O'Nions et al. (2024) [88]	n=15675 (ASD, no ID), n=156750 (comparison), n=6437 (ASD+ID), n=64370 (comparison); Age: ≥18; UK; IQVIA Medical research data (electronic health records from multiple hospitals); comparison matched by age, sex, location	Depression: 23.35% (ASD, no ID), 7.64% (comparison), 12.85% (ASD+ID), 9.80% (comparison) [baseline]; crude incidence based on observation period: 263.27(246.78-280.58) (ASD, no ID), 144.42(141.05-147.86) (comparison), 106.15(93.29-120.27) (ASD+ID), 143.67(138.92-148.54) (comparison)	p: <0.001 (ASD no ID vs comparison), p:<0.001 (ASD+ID vs comparison) [baseline]; ASD+ID vs comparison, incidence rate ratio: 0.73 (0.64-0.83) (ASD+ID), 1.79(1.67-1.92) (ASD no ID)
Al-Ayadhi et al. (2025) [3]	n=363 [n=189 (ASD), n=174 (non-ASD)]; Online questionnaire (self or caregiver); Saudi Arabia; August 2022 - January 2023	22.8% (ASD), 3.4% (non-ASD)	p: 0.000
Salehi et al. (2025) [102]	n=6870 (ASD), n=2686160 (non-ASD); Age: 15-44; Administrative health data, Canada, 2017-18	16.7%	OR: 1.6, p <0.001 [Female vs Male]
Diemer et al. (2025) [27]	n=1424 (ASD); Adult; SPARK	Depression: 61.79% (total), 56.94% (diagnosed <18), 64.42% (diagnosed >18)	p-value 0.006 (between age groups)
Lacroix et al. (2025) [64]	Narrative and Quantitative Review; N =12, n= 38492 (ASD, female), n= 117343 (ASD, male); excluded papers with less than 12.5% females	Depressive disorders, Pooled prevalence [95% CI]: 19.2% [11.3-30.6] (ASD, female), 12.9% [7.7-21.1] (ASD, male)	p:<0.001, OR: 1.63

1.2.3 Other Conditions

Table 21: Prevalence of depression in those with ASD, table 5.

Source	Sample	Prevalence	Other Notes
Lever and Geurts (2016) [68]	n=138 (ASD), n=170 (non-ASD); Age: 19-79; Mental Health Institutions and online recruitment; Netherlands	Premenstrual Dysphoric Disorder: 20.9% (ASD), 2.7% (non-ASD)	p-value:$\leq$0.001
Brookman-Frazee et al. (2018) [16]	n=201 (ASD); Age: 4-14; data from community effectiveness trial; Southern California; 2012-15; MINI-KID-P	Manic episode (lifetime): 5% (ASD); Hypomaniac episode (lifetime): 3%	

1.3 Schizophrenia Spectrum Disorders

Psychosis/Psychotic Disorders

Table 22: Prevalence of psychosis and psychotic disorders in those with ASD, table 1.

Source	Sample	Prevalence	Other Notes
Croen et al. (2015) [23]	n=1507 (ASD), n=15070 (age, sex matches controls); Adults; Kaiser Permanente Northern California; 2008-12	Psychosis: 6.30% (ASD), 0.55% (non-ASD)	Chi-square p value: <0.0001
Davignon, et al. (2018) [25]	n=4123 (ASD), n=20615 (non-ASD, non-ADHD); Age: 14-25; Kaiser Permanente Northern California, 2013-15	1.7% (ASD), 0.4% (non-ASD)	aOR (95% CI): 4.83 (3.46 - 6.73)
Hand et al. (2020) [34]	n=4685 (ASD), n=46850 (non-ASD); Age: ≥65; Medicare; 2016-17	Schizophrenia and psychotic disorders: 17.8% (ASD), 0.8% (non-ASD)	aOR: 25.3(22.4-28.7)
Bowden et al. (2020) [15]	n=8955 (ASD), n=1551342 (non-ASD); Age: 10-24; IDI database, New Zealand	Psychosis: 5.1% (ASD), 0.4% (non-ASD)	aOR: 15.9 (95% CI: 14.1-17.8) - standardized for age, sex, ethnicity, deprivation, and urban/rural
Tint et al. (2021) [113]	n=6870 (ASD), n=2686160 (non-ASD); Age: 15-44; Administrative health data, Canada, 2017-18	Psychotic mental illness: 9.1% (ASD), 1.1% (non-ASD)	Standard difference: 0.37
Rødgaard et al. (2021) [100]	n=11,914 (ASD, men), n=4212 (ASD, women); Age ≤16; Danish National Patient Registry	Psychotic disorder: 4% (men with ASD), 8% (women with ASD)	
Martini et al. (2022) [79]	n=20841 (ASD) [n=13712 (male, ASD), n=7129 (female, ASD)], n=1314912 (non-ASD) [n=671727 (male, non-ASD), n=643185 (female, non-ASD)]; Age: 16-24; Multiple national registers; Sweden; 2001-13	Psychotic disorders: 4.3% (ASD, total), 0.3% (non-ASD, total); 4.0% (ASD, male), 4.8% (ASD, female), 0.3% (non-ASD, male), 0.3% (non-ASD, female)	Male vs female: HR: 1.18 (95% CI: 0.80-1.75), p:0.40 - adjusted for birth year ADHD, ID

Table 23: Prevalence of psychosis and psychotic disorders in those with ASD, table 2.

Source	Sample	Prevalence	Other Notes
Mutluer et al. (2022) [85]	Review - 4 population and EHR studies (age 6-18)	Psychotic Disorders: 0.6% (overall), 1.1% (adolescents)	
Schott et al. (2022) [105]	n=155,617 (ASD), n=466,851 (non-ASD); Adults; Medicaid; 2008–12	Other psychoses: 12.33% (ASD), 7.29% (non-ASD)	aOR: 1.75 (1.70 1.79)
Varcin et al. (2022) [120]	Meta-analysis, N=45, n=63,657	Pooled prevalence: 9.4% (95% CI = 7.52, 11.72)	
Underwood et al. (2022) [118]	n=7943 (core ASD), n=25941 (control); Age: ≥18; UK; SAIL Databank, 2001-16	18.30% (ASD), 2.30% (non-ASD)	
Tint et al. (2023) [114]	n=3220 (ASD, women), n=12920 (no DD, women), n=3217 (other DD, women), n=7426 (ASD, men), n=29687 (no DD, men), n=7398 (other DD, men); Canada; administrative and social services data - ICES	Psychotic psychiatric diagnosis: 12.7% (ASD, women), 0.7% (other DD, women), 9.1% (no DD, women), 13.7% (ASD, men), 1.0% (no DD, men), 10.5% (other DD, men)	aRR: 18.28 (95% 14.60-22.90) [ASD vs no DD, women], aRR: 1.46 (95% 1.27-1.69) [ASD vs other DD, women], aRR: 13.07 (95% 11.54-14.80) [ASD vs no DD, men], aRR: 1.35 (95% 1.24-1.48) [ASD vs other DD, men]
Lopez-Arvizu et al. (2025) [72]	n=41230 (ASD, ID, or both), n=29001 (ASD), n=27054 (ID); Age: 5-24; Medicaid (6+ months, Midwestern states); 2010-20	Psychosis or other thought disorders: 17.2% (ASD), 17.2% (ID), 14.6% (combined)	
Vaquerizo-Serrano et al. (2022) [119]	N=4, Meta-analysis	ASD: 11.6% (clinical high-risk for psychosis)	

Schizophrenia

Table 24: Prevalence of schizophrenia in those with ASD, table 1.

Source	Sample	Prevalence	Other Notes
Croen et al. (2015) [23]	n=1507 (ASD), n=15070 (age, sex matches controls); Adults; Kaiser Permanente Northern California; 2008-12	7.83% (ASD), 0.37% (non-ASD)	Chi-square p value: <0.0001
Schendel et al. (2016) [104]	n=20492 (ASD), 1892412 (non-ASD); Age: >1.5; Danish Civil Registration Service and the Danish Medical Birth Register; born 1980-2010	Schizophrenia/schizotypal/or other delusional disorder: 5.6% (ASD), 0.7% (non-ASD)	
Vohra et al. (2017) [123]	n=1772 (ASD), n=5316 (non-ASD); Age: 22-64; three state Medicaid Analytic eXtract; 2000-08	Schizophrenia: 16.6% (ASD), 11.1% (non-ASD)	aOR (95% CI): 1.61 (1.38, 1.88), p:<0.001
Houghton et al. (2017) [44]	n=46943 (ASD, commercial), Truven Health MarketScan Commercial Database, 2014; n=46696 (ASD, Medicaid), Truven Health MarketScan Multi-State Medicaid Database; Age: >3	Schizophrenia: 1.09% (commercial), 2.29% (medicaid)	
Zheng et al. (2018) [130]	Meta-analysis; N=10 (Schizophrenia in ASD), N=6 (ASD in schizophrenia); n=14,945 (ASD), n=1,950,113 (non-ASD)	Schizophrenia: [odds ratio = 3.55, 95% CI: 2.08–6.05, P <.001]	
Lai, et al. (2019) [65]	Meta-analysis, N=96	4%	

Table 25: Prevalence of schizophrenia in those with ASD, table 2.

Source	Sample	Prevalence	Other Notes
Hossain et al. (2020) [43]	Umbrella Review - N=8	Schizophrenia spectrum and other psychotic disorders: 4% - 67%	
Chien et al. (2021) [22]	n=3837 (ASD), n=38370 (non-ASD); Children; Taiwan's claims database; 2000, 2005, 2010	9.7/1,000 person year (ASD), 0.3/1,000 person year (non-ASD)	Crude hazard ratio: 30.7 (25.2–37.3)
Jadav and Bal (2022) [52]	n=4657 (ASD); Adults; online survey, SPARK; 2022	Schizophrenia, other psychosis or schizoaffective disorder: 4.5%	
Schott et al. (2022) [105]	n=155,617 (ASD), n=466,851 (non-ASD); Adults; Medicaid; 2008–12	11.23% (ASD), 9.3% (non-ASD)	aOR: 1.21 (1.18 1.24)
Underwood et al. (2022) [118]	n=7943 (core ASD), n=25941 (control); Age: $\geq$18; UK; SAIL Databank, 2001-16	5.20% (ASD), 0.70% (non-ASD)	
Micai et al. (2023) [82]	Meta-analysis of observational studies, N=29 [N=20 (clinical studies from community samples), N=9 (population/registry based)], n=65841	Pooled prevalence: 10%, Range: 7% - 13%	
Rim et al. (2023) [95]	n=381 (ASD), n=6179 (non-ASD); Age: 0-19; National Health Insurance System National Sample Cohort; South Korea; 2002–13	2.69% (ASD), 0.06% (non-ASD)	aOR: 32.05 (19.51,52.66)
Semovski et al. (2023) [106]	n=2020 (ASD); Age: 12-18; records + interviews; Canada	Schizophrenia or other psychotic disorders: 1.3% (ASD)	

Table 26: Prevalence of schizophrenia in those with ASD, table 4.

Source	Sample	Prevalence	Other Notes
Longo et al. (2024) [71]	n=30258 (ASD), n=60516 (non-ASD); Age: 18-64; IBM Market Scan Commercial Claims and Encounters Database; 2019-20	Schizophrenia and psychotic disorders: 6.8% (ASD), 0.9% (non-ASD)	aOR: 7.4 (95% CI: 6.7, 8.2)
Hsu et al. (2024) [45]	n=22698 (ASD), [n=19191 (male), n=3507 (female)]; Age: 2-19; Taiwan's National Health Insurance Research Database; 2001-11; ICD-9	Schizophrenia: 1.8% (ASD, total); 1.4% (ASD, male), 3.5% (ASD, female)	RR: 0.45 (95% CI: 0.36-0.57) [Male vs female (ref)] - adjusted for age, income, residence, and parental psychiatric disorders. RR: 0.66 (95% CI: 0.49-0.89) [Parental psychiatric disorders - Male vs female (ref)] - adjusted for age, income, residence
Diemer et al. (2025) [27]	n=1424 (ASD); Adult; SPARK	Schizophrenia: 3.79% (total), 3.67% (diagnosed <18), 3.85% (diagnosed >18)	p-value 0.862 (between age groups)
Lacroix et al. (2025) [64]	Narrative and Quantitative Review; N = 9, n= 56329 (ASD, female), n= 155068 (ASD, male);excluded papers with less than 12.5% females	Schizophrenia spectrum and other psychotic disorders, Pooled prevalence [95% CI]: 8.5% [5.6-12.7] (ASD, female), 6.3% [3.8-10.3] (ASD, male)	p: 0.004, OR: 1.37

Table 27: Prevalence of ASD in those with schizophrenia.

Source	Sample	Prevalence	Other Notes
Zheng et al. (2018) [130]	Meta-analysis; N=6 (ASD in schizophrenia); n=930	ASD: ASD: 3.4 to 52%	

1.4 Personality Disorders

Table 28: Prevalence of personality disorders in those with ASD

Source	Sample	Prevalence	Other Notes
Schendel et al. (2016) [104]	n=20492 (ASD), 1892412 (non-ASD); Age: >1.5; Danish Civil Registration Service and Medical Birth Register; born 1980-2010	Disorders of adult personality and behavior: 3.4% (ASD), 1.2% (non-ASD)	
Vohra et al. (2017) [123]	n=1772 (ASD), n=5316 (non-ASD); Age: 22-64; three state Medicaid Analytic eXtract; 2000-08	Personality disorders: 2.5% (ASD), 2.0% (TD)	aOR (95% CI): 1.26 (0.89, 1.81), p: ns
Hand et al. (2020) [34]	n=4685 (ASD), n=46850 (non-ASD); Age: ≥65; Medicare; 2016-17	Personality disorders: 3.1% (ASD), 0.1% (non-ASD)	aOR: 24.1(17.8-32.5)
Bowden et al. (2020) [15]	n=8955 (ASD), n=1551342 (non-ASD); Age: 18-24; IDI database, New Zealand	Personality: 2.4% (ASD), 0.3% (non-ASD)	aOR: 9.0 (95% CI: 6.3-11.8) - age, sex, ethnicity, deprivation, and urban/rural standardized
May et al. (2021) [81]	N=12	BPD: 4%	[95% CI 0%–9%]. ASD in BPD, 3% [95% CI 1%–8%]
Micai et al. (2023) [82]	Meta-analysis of observational studies, N=14 [N=10 (clinical studies from community samples), N=4 (population/registry based)], n=8306	Personality disorders: Pooled prevalence: 7%, Range: 4% - 10%	
Longo et al. (2024) [71]	n=30258 (ASD), n=60516 (non-ASD); Age: 18-64; IBM Market Scan Commercial Claims and Encounters Database; 2019-20	Personality disorders: 3.2% (ASD), 0.5% (non-ASD)	aOR: 6.3 (95% CI: 5.5, 7.2)
Diemer et al. (2025) [27]	n=1424 (ASD); Adult; SPARK	Personality disorder: 12.35% (total), 11.63% (diagnosed <18), 12.65% (diagnosed >18)	p-value 0.58 (between age groups)
Lacroix et al. (2025) [64]	N =5, n= 40828 (ASD, female), n= 106579 (ASD, male); Narrative and Quantitative Review; excluded papers with less than 12.5% females	Personality disorders, Pooled prevalence [95% CI]: 6.8% [3.4-13.1] (ASD, female), 4.5% [2.0-9.9] (ASD, male)	p:0.003, OR: 1.55

1.5 Substance and Addiction Related Disorders

Table 29: Prevalence of behavioral addictions in those with ASD.

Source	Sample	Prevalence	Other Notes
Kervin et al. (2021) [57]	N=30; Systematic Review	N=27 (more common), N=1 (significantly less common), N=3 (no significant difference)	

Table 30: Prevalence of substance abuse issues in those with ASD, table 1

Source	Sample	Prevalence	Other Notes
Arnevik and Helverschou (2016) [6]	Systematic Review; N=1; Helverschou et al, 2015 (n=48)	Substance abuse: 15%	
Lever and Geurts (2016) [68]	n=138 (ASD), n=170 (non-ASD); Age: 19-79; Mental Health Institutions and online recruitment; Netherlands	Substance-related disorder: 15.9% (ASD), 25.3% (non-ASD)	p-value:≤0.5
Butwicka et al. (2017) [18]	n=26986 (ASD), n=1349300 (non-ASD); Sweden; Population Register; Born: January 1, 1973 - December 31, 2009	Substance problem: 4.0% (ASD), 1.3% (non-ASD); Substance use disorder: 3.6% (ASD), 0.8% (non-ASD)	Univariate crude OR: 3.3 (3.1-3.6); 5.2 (4.9-5.6)
Weiss, et al. (2017) [125]	n=5085 (ASD), n=393,263 (no developmental disorder); Age: 18-24; Administrative health data, Canada	Substance related and addictive disorder diagnosis: 5.16% (ASD), 3.17% (non-ASD)	aOR: 1.58 (1.39-1.79)
Vohra et al. (2017) [123]	n=1772 (ASD), n=5316 (non-ASD); Age: 22-64; three state Medicaid Analytic eXtract; 2000-08	Alcohol or substance abuse: 2.0% (ASD), 17.8% (non-ASD)	0.09 (0.07, 0.13), p:<0.001
Hossain et al. (2020) [43]	Umbrella Review - N=2	Substance use disorder: 0.7% - 36%	
Hand et al. (2020) [34]	n=4685 (ASD), n=46850 (non-ASD); Age: ≥65; Medicare; 2016-17	Substance use disorder: 9.2% (ASD), 9.4% (non-ASD)	aOR: 0.9(0.9-1.0)
Ressel et al. (2020) [93]	N=26, systematic review and qualitative synthesis	Substance abuse: 1.3% - 36%	
Tint et al. (2021) [113]	n=6870 (ASD), n=2686160 (non-ASD); Age: 15-44; Administrative health data, Canada, 2017-18	Substance use disorder: 2.4% (ASD), 1.1% (non-ASD)	Standard difference: 0.10 t
Brooks et al. (2021) [17]	n=1062 (ASD), n=79175 Age: 1-24; Canada; Electronic Medical Record Primary Care database	Substance-related disorders: 0.8% (ASD), 0.8% (non-ASD)	Prevalence ratio: 1.08 (95% CI: 0.55, 2.16) - adjusted for age, sex, neighborhood income, geographic location

Table 31: Prevalence of substance abuse issues in those with ASD, table 2

Source	Sample	Prevalence	Other Notes
Yule (2021) [4]	n=230 (ASD), n=219 (ADHD), n=230 (controls, age matched); age: $\geq$ 12; 2007-16; high functioning ASD - academic center	Substance abuse, any (lifetime): 13% (ASD), 36% (ADHD), 19% (control)	p: <0.001 (ASD vs ADHD), p: $\geq$0.05 (ASD vs controls)
Huang et al. (2021) [46]	n=6599 (ASD), n=26396 (non-ASD); controls age, sex, date matched 1:4; Taiwan National Health Insurance Research Database; ICD-9; 2000-15	SUD: 1.94% (ASD), 1.55% (non-ASD); 723 per 100-000 person-years (ASD), 350 per 100000 person-years (non-ASD)	aHR: 2.33 (95% CI: 1.89-2.87). Adjusted based on psychotherapy, age, sex, education, martial status, psychiatric comorbidities, CCI score, season of diagnosis, levels of care, hospitalizations, length of admission, residence, income, and follow up period
Roux et al. (2022) [96]	n=209795 (ASD), n=790719 (ID), n=123705 (ASD+ID), n=1768499 (control); Age: 11-64; Medicaid, all states + Washington DC; ICD-9; 2008-12	SUD, any: 3.08% (ASD), 3.01% (ASD+ID), 5.70% (ID), 7.52% (control)	
Micai et al. (2023) [82]	Meta-analysis of observational studies, N=18 [N=13 (clinical studies from community samples), N=5 (population/registry based)], n=20311	Substance use disorder: Pooled prevalence: 5%, Range: 2% - 11%	
Longo et al. (2024) [71]	n=30258 (ASD), n=60516 (non-ASD); Age: 18-64; IBM Market Scan Commercial Claims and Encounters Database; 2019-20	Substance Abuse Disorders: 6.7% (ASD), 6.7% (non-ASD)	aOR: 1.0 (95% CI: 1.0, 1.1)
Lushin et al. (2025) [73]	n=388426 (ASD), n=745699 (non-ASD); Age 11-64; Medicare Claims data; USA; 2008-16	Any substance abuse disorder: 6.52% (ASD), 16.31% (non-ASD)	aOR: 0.28, (95% CI: 0.27, 0.28), p-value:<0.0001 - includes alcohol, cannibis, opiods, hallucinogens, and drugs

Table 32: Prevalence of substance abuse issues in those with ASD, table 3

Source	Sample	Prevalence	Other Notes
Diemer et al. (2025) [27]	n=1424 (ASD); Adult; SPARK	Substance abuse: 8.85% (total), 6.94% (diagnosed <18), 9.86% (diagnosed >18)	p-value 0.065 (between age groups)
Garcia et al. (2025) [31]	n=22911 (ASD), n=647029 (non-ASD); Child and Caregiver Linked Utilization and Outcomes Database (CCOULD) [children in both Medicaid and child welfare system in Florida (January 2017–June 2021) and Kentucky (January 2017–January 2020)]	Any SUD: 1.7% (ASD), 3.8% (non-ASD);	OR (vs autism): 0.45, 95% CI [.41-0.50], p: <0.001
Lopez-Arvizu et al. (2025) [72]	n=41230 (ASD, ID, or both), n=29001 (ASD), n=27054 (ID); Age: 5-24; Medicaid (6+ months, Midwestern states); 2010-20	Substance abuse: 5.6% (ASD), 7.3% (ID), 6.2% (combined)	

1.5.1 Alcohol

Table 33: Prevalence of alcohol use issues in those with ASD, table 1

Source	Sample	Prevalence	Other Notes
Croen et al. (2015) [23]	n=1507 (ASD), n=15070 (age, sex matches controls); Adults; Kaiser Permanente Northern California; 2008-12	Alcohol abuse: 2.19% (ASD), 3.92% (non-ASD)	Chi-square p value: 0.0008
Croen et al. (2015) [23]	n=1507 (ASD), n=15070 (age, sex matches controls); Adults; Kaiser Permanente Northern California; 2008-12	Alcohol dependence: 1.06% (ASD), 1.96% (non-ASD)	Chi-square p value: 0.014
Butwicka et al. (2017) [18]	n=26986 (ASD), n=1349300 (non-ASD); Sweden; Population Register; Born: January 1, 1973 - December 31, 2009	Alcohol problem: 2.1% (ASD), 0.6% (non-ASD)	Univariate crude OR: 4.0 (3.7-4.4)
Yule (2021) [4]	n=230 (ASD), n=219 (ADHD), n=230 (controls, age matched); age: $\geq$ 12; 2007-16; high functioning ASD - academic center	Substance abuse, alcohol (lifetime): 11% (ASD), 29% (ADHD), 16% (control)	p: <0.001 (ASD vs ADHD), p: $\geq$0.05 (ASD vs controls)
Huang et al. (2021) [46]	n=6599 (ASD), n=26396 (non-ASD); controls age, sex, date matched 1:4; Taiwan National Health Insurance Research Database; ICD-9; 2000-15	Alcohol use disorder: 1.27% (ASD), 1.13% (non-ASD); 474 per 100-000 person-years (ASD), 256 per 100000 person-years (non-ASD)	aHR: 2.07 (95% CI: 1.60-2.63). Adjusted based on psychotherapy, age, sex, education, martial status, psychiatric comorbidities, CCI score, season of diagnosis, levels of care, hospitalizations, length of admission, residence, income, and follow up period

Table 34: Prevalence of alcohol use issues in those with ASD, table 2

Source	Sample	Prevalence	Other Notes
Weir et al. (2021) [124]	n=1182 (ASD), n=1202 (non-ASD); Age: 16-90; survey - Autism and Physical Health Survey; Feb 2018- Aug 2019	Consumes alcohol $\geq$3 days per week: 15.99% (ASD), 22.21% (non-ASD); Consumes $\geq$5 alcoholic beverages per session: 3.81% (ASD), 8.15% (non-ASD)	OR (95% CI) - adjusted for age, sex, ethnicity, education, country of residence: 0.69 (0.55-0.86), p: 0.0022 [days per week]; 0.38 (0.26-0.56), p:<0.0001 [beverages per session]
Kaltenegger et al. (2021) [55]	Age 15: n=250 (ASD), n=9800 (non-ASD); Age: 18 n=163 (ASD), n=7768 (non-ASD); Age: 24 n=83 (ASD), n=2799 (non-ASD); Study is longitudinal and the age groups are not exclusive nor a continuous group 82-85% of population retained through sampling points; Child and Adolescent Twin Study in Sweden; phone interview;	Hazardous/harmful alcohol use: 3.60% (ASD, age 15, all), 5.38% (non-ASD, age 15, all), 9.20% (ASD, age 18, all), 27.19% (non-ASD, age 18, all), 13.25% (ASD, age 24, all), 27.26% (non-ASD, age 24, all); By sex: 1.76% (ASD, age 15, male), 7.50% (ASD, age 15, female), 4.42% (non-ASD, age 15, male), 6.17% (non-ASD, age 15, male), 9.35% (ASD, age 18, male), 8.93% (ASD, age 18, female), 20.23% (non-ASD, age 18, male), 32.17% (non-ASD, age 18, male), 14.58% (ASD, age 24, male), 11.43% (ASD, age 24, female), 24.65% (non-ASD, age 24, male), 29.23% (non-ASD, age 24, male)	p: 0.275 (Age 15, ASD vs non, all sex), p: <0.001 (Age 18, ASD vs non, all sex), p: 0.007 (Age 24, ASD vs non, all sex)
Martini et al. (2022) [79]	n=20841 (ASD) [n=13712 (male, ASD), n=7129 (female, ASD)], n=1314912 (non-ASD) [n=671727 (male, non-ASD), n=643185 (female, non-ASD)]; Age: 16-24; Multiple national registers; Sweden; 2001-13	Alcohol use disorders: 2.6% (ASD, total), 0.8% (non-ASD, total); 2.3% (ASD, male), 3.2% (ASD, female), 0.8% (non-ASD, male), 0.8% (non-ASD, female)	Male vs female: HR: 0.72 (95% CI: 0.42-1.21), p:0.21 - adjusted for birth year ADHD, ID

Table 35: Prevalence of alcohol use issues in those with ASD, table 3

**Source**	**Sample**	**Prevalence**	**Other Notes**
Schott et al. (2022) [105]	n=155,617 (ASD), n=466,851 (non-ASD); Adults; Medicaid; 2008–12	Alcohol abuse/dependence: 2.41% (ASD), 5.21% (non-ASD)	aOR: 0.42 (0.41 0.44)
Underwood et al. (2022) [118]	n=7943 (core ASD), n=25941 (control); Age: ≥18; UK; SAIL Databank, 2001-16	Alcohol Misuse: 3.9% (ASD), 3.2% (non-ASD)	
Barber et al. (2025) [8]	Review	Alcohol use disorder: Clinical: 16.1% (pooled), 6.7%-39% (range); Population: 1.6% (pooled), 0.7%-9.5% (range)	
Lushin et al. (2025) [73]	n=388426 (ASD), n=745699 (non-ASD); Age 11-64; Medicare Claims data; USA; 2008-16	Alcohol use disorder: 1.82% (ASD), 3.50% (non-ASD)	aOR: 0.40, (95% CI: 0.39, 0.42), p-value:<0.0001
Garcia et al. (2025) [31]	n=22911 (ASD), n=647029 (non-ASD); Child and Caregiver Linked Utilization and Outcomes Database [children in both Medicaid and child welfare system in Florida (Jan 2017–June 2021) and Kentucky (Jan 2017–Jan 2020)]	Alcohol SUB: 0.4% (ASD), 0.9% (non-ASD)	OR: 0.43 [CI: 0.35-0.53], p: <0.001

1.5.2 Drugs

Table 36: Prevalence of drug use issues in those with ASD, table 1

Source	Sample	Prevalence	Other Notes
Croen et al. (2015) [23]	n=1507 (ASD), n=15070 (age, sex matches controls); Adults; Kaiser Permanente Northern California; 2008-12	Drug abuse: 2.59% (ASD), 2.77% (non-ASD)	Chi-square p value: 0.67
Croen et al. (2015) [23]	n=1507 (ASD), n=15070 (age, sex matches controls); Adults; Kaiser Permanente Northern California; 2008-12	Drug dependence: 1.79% (ASD), 2.16% (non-ASD)	Chi-square p value: 0.35
Butwicka et al. (2017) [18]	n=26986 (ASD), n=1349300 (non-ASD); Sweden; Population Register; Born: January 1, 1973 - December 31, 2009	Drug problems: 2.1% (ASD), 0.3% (non-ASD)	Univariate crude OR: 8.5 (7.7-9.3)
Davignon, et al. (2018) [25]	n=4123 (ASD), n=20615 (non-ASD, non-ADHD); Age: 14-25; Kaiser Permanente Northern California, 2013-15	Drug abuse/dependence: 1.5% (ASD), 2.2% (non-ASD)	aOR (95% CI): 0.65 (0.50 - 0.85)
Yule (2021) [4]	n=230 (ASD), n=219 (ADHD), n=230 (controls, age matched); age: $\geq$ 12; 2007-16; high functioning ASD - academic center	Substance abuse, drugs (lifetime): 6% (ASD), 24% (ADHD), 8% (control)	p: <0.05 (ASD vs ADHD), p: $\geq$(ASD vs controls), p: 0.32 (ADHD vs controls)
Huang et al. (2021) [46]	n=6599 (ASD), n=26396 (non-ASD); controls age, sex, date matched 1:4; Taiwan National Health Insurance Research Database; ICD-9; 2000-15	Drug use disorder: 0.67% (ASD), 0.42% (non-ASD); 248 per 100-000 person-years (ASD), 95 per 100000 person-years (non-ASD)	aHR: 2.33 (95% CI: 1.89-2.87). Adjusted based on psychotherapy, age, sex, education, martial status, psychiatric comorbidities, CCI score, season of diagnosis, levels of care, hospitalizations, length of admission, residence, income, and follow up period

Table 37: Prevalence of drug use issues in those with ASD, table 2

Source	Sample	Prevalence	Other Notes
Weir et al. (2021) [124]	n=1182 (ASD) [n=746 (female), n=437 (male)], n=1202 (non-ASD) [n=824 (female), n=378 (male)]; Age: 16-90; survey - Autism and Physical Health Survey; Feb 2018- Aug 2019	Ever used drugs: 37.58% (ASD, female), 35.77% (non-ASD, female), 35.40% (ASD, male), 52.65% (non-ASD, male)	OR (95% CI) - adjusted for age, sex, ethnicity, education, country of residence: 1.11 (0.82-1.50), p: 1.00 [Ever, female], 0.53 (0.35-0.80), p: 0.0022 [Ever, male]
Schott et al. (2022) [105]	n=155,617 (ASD), n=466,851 (non-ASD); Adults; Medicaid; 2008–12	Drug abuse/dependence: 3.89% (ASD), 7.55% (non-ASD)	aOR: 0.42 (0.40 0.43)
Underwood et al. (2022) [118]	n=7943 (core ASD), n=25941 (control); Age: ≥18; UK; SAIL Databank, 2001-16	Drug Misuse; 4.10% (ASD), 2.80% (non-ASD)	
Lushin et al. (2025) [73]	n=388426 (ASD), n=745699 (non-ASD); Age 11-64; Medicare Claims data; USA; 2008-16	Opioids Use Disorder: 0.61% (ASD), 2.77% (non-ASD)	aOR: 0.19, (95% CI: 0.19, 0.2), p-value:<0.0001
Lushin et al. (2025) [73]	n=388426 (ASD), n=745699 (non-ASD); Age 11-64; Medicare Claims data; USA; 2008-16	Hallucinogens abuse: 0.07% (ASD), 0.08% (non-ASD)	aOR: 0.50, (95% CI: 0.42, 0.59), p-value:<0.0001
Lushin et al. (2025) [73]	n=388426 (ASD), n=745699 (non-ASD); Age 11-64; Medicare Claims data; USA; 2008-16	Drug use disorder: 2.98% (ASD), 6.59% (non-ASD)	aOR: 0.29, (95% CI: 0.28, 0.29), p-value:<0.0001
Garcia et al. (2025) [31]	n=22911 (ASD), n=647029 (non-ASD); Child and Caregiver Linked Utilization and Outcomes Database (CCOULD) [children in both Medicaid and child welfare system in Florida (January 2017–June 2021) and Kentucky (January 2017–January 2020)]	Opioid SUB: 0.1% (ASD), 1.1% (non-ASD); Sedative SUB: 0.2% (ASD), 0.3% (non-ASD); Cocaine SUB: 0.1% (ASD), 0.6% (non-ASD); Other stimulants SUB: 0.3% (ASD), 0.6% (non-ASD); Hallucinogen SUB: 0.1% (ASD), 0.1% (non-ASD); Other psychoactive SUB: 0.1% (ASD), 0.1% (non-ASD)	OR: 0.12 [CI: .08-.17], p: <0.001; OR: 0.58 [CI: .43-.80], p: <0.001; OR: 0.22 [CI: .16-.31], p: <0.001; OR: 0.40 [CI: .31-.52], p: <0.001; OR: 1.40 [CI: .89-2.19], p: ns; OR: 0.38 [CI: .31-.47], p: <0.001

1.5.3 Smoking

Table 38: Prevalence of smoking among those with ASD.

Source	Sample	Prevalence	Other Notes
Butwicka et al. (2017) [18]	n=26986 (ASD), n=1349300 (non-ASD); Sweden; Population Register; Born: January 1, 1973 - December 31, 2009	Tobacco problem: 0.1% (ASD), 0.0% (non-ASD)	Univariate crude OR: 6.4 (3.8-10.5)
Davignon, et al. (2018) [25]	n=4123 (ASD), n=20615 (non-ASD, non-ADHD); Age: 14-25; Kaiser Permanente Northern California, 2013-15	Smoking: 5.7% (ASD), 12.0% (non-ASD)	aOR (95% CI): 0.40 (0.35-0.47)
Weir et al. (2021) [124]	n=1182 (ASD) [n=746 (female), n=437 (male)], n=1202 (non-ASD) [n=824 (female), n=378 (male)]; Age: 16-90; survey - Autism and Physical Health Survey; Feb 2018- Aug 2019	Ever smoked: 49.20% (ASD, female), 54.25% (non-ASD, female), 50.80% (ASD, male), 64.55% (non-ASD, male); Smokes weekly: 31.64% (ASD, female), 32.89% (non-ASD, female), 32.72% (ASD, male), 38.62% (non-ASD, male)	OR (95% CI) - adjusted for age, sex, ethnicity, education, country of residence: 0.81 (0.60-1.09), p: 0.47 [Ever, female], 0.50 (0.32-0.76), p: 0.0022 [Ever, male], 0.93 (0.68-1.29), p: 1.00 [Weekly, female], 0.64 (0.41-1.00), p: 0.077 [Weekly, male]
Yule (2021) [4]	n=230 (ASD), n=154 (ADHD), n=163 (controls, age matched); age: $\geq$ 12; 2007-16; high functioning ASD - academic center	Substance abuse, cigarette smoking (lifetime): 6% (ASD), 31% (ADHD), 9% (control)	p: <0.001 (ASD vs ADHD), p: $\geq$0.05 (ASD vs controls)
Lushin et al. (2025) [73]	n=388426 (ASD), n=745699 (non-ASD); Age 11-64; Medicare Claims data; USA; 2008-16	Cannabis Abuse: 2.02% (ASD), 2.78% (non-ASD)	aOR: 0.34, (95% CI: 0.33,0.35), p-value:<0.0001
Garcia et al. (2025) [31]	n=22911 (ASD), n=647029 (non-ASD); Child and Caregiver Linked Utilization and Outcomes Database [children in both Medicaid and Florida welfare system (Jan 2017–June 2021) and Kentucky (Jan 2017–Jan 2020)]	Cannabis SUB: 1.3% (ASD), 2.2% (non-ASD)	OR: 0.57 [CI: 0.51-0.64], p: <0.001

1.6 Trauma and Stressor Related Conditions

Table 39: Prevalence of trauma and trauma related conditions in those with ASD.

Source	Sample	Prevalence	Other Notes
Lever and Geurts (2016) [68]	n=138 (ASD), n=170 (non-ASD); Age: 19-79; Mental Health Institutions and online recruitment; Netherlands	PTSS: 2.9% (ASD), 0.6% (non-ASD)	P-value: ≤0.05
Hollocks et al. (2019) [41]	Review - N=5	PTSD: 1% (current), 5% (lifetime)	
Rumball (2019) [97]	Review, N=12 (group studies), n=703	PTSD: 0-17.4%	
Haruvi-Lamdan (2020) [36]	n=25 (ASD), n=25 (non-ASD); Age: 18-35; Online survey and in person interview	PTSD: 32% (ASD), 4% (non-ASD)	
Hoch and Youssef (2020) [39]	n=7695; Children and adolescents; community mental health provider; Minneapolis, Minnesota; 2013-18	PTSD: 4.25% (ASD), 14.83% (DD), 29.81% (mental health comparison group); Reactive Attachment Disorders or Acute Stress Disorders: 0.34% (ASD), 2.54% (DD), 1.73% (comparison group)	Trauma diagnoses - OR: 0.15 CI(0.12, 0.17), p: <0.001 [ASD vs mental health comparison group]
Rumball et al. (2020) [98]	n=59 (ASD), Age: 19-67; Online survey	PTSD: 45% (after DSM-5 trauma), 43% (after non-DSM-5 trauma)	
Rumball et al. (2021) [99]	n=38 (ASD), n=44 (TD); Age: 18-68; Online Survey	Traumatic evnts: 1+: 94.7% (ASD), 90.9% (non-ASD), 4+s: 63.2% (ASD), 34.1% (non-ASD); PTSD symptomatology after: 47% (ASD), 5% (non-ASD); PTSD: 45% (ASD), 4.5% (non-ASD)	
Micai et al. (2023) [82]	Meta-analysis of observational studies, N=12 [N=9 (clinical studies from community samples), N=3 (population/registry based)], n=21162	Trauma Stress Related Disorders: Pooled prevalence: 4%, Range: 0% - 10%	

Table 40: Prevalence of trauma and trauma related conditions in those with ASD, table 2.

Source	Sample	Prevalence	Other Notes
Junewicz et al. (2024) [54]	n=289 (ASD), n=2439 (non-ASD); Age: 5-17; Psychiatric emergency department, NYC; 2019-21		Prevalence ratio: 0.58, 95% CI 0.41-0.80
Lopez-Arvizu et al. (2025) [72]	n=41230 (ASD, ID, or both), n=29001 (ASD), n=27054 (ID); Age: 5-24; Medicaid (6+ months, Midwestern states); 2010-20	Abuse/neglect: 6.6% (ASD), 8.1% (ID), 7.1% (combined)	
Mansour et al. (2025) [76]	Review, N=17 (children and young people (CYP)), N=13 (adults)	PTSD: 1.11% (95% CI: 0.32-2.38) (ASD, CYP), 2.06% (2.54-2.90) (ASD, adults)	

Attachment Disorder

Table 41: Prevalence of attachment disorder in those with ASD.

Source	Sample	Prevalence	Other Notes
Minnis et al. (2020) [83]	n=3349 (general population) Age: 5-6; growing up in Scotland database	Overlap: n=61 (1.8%); high RAD score: 43.9% (high ASD score), high ASD: 39.6% (high RAD score)	
Semovski et al. (2023) [106]	n=2020 (ASD); Age: 12-18; records + interviews; Canada	Reactive Attachment Disorder: 1.67% (ASD)	

1.7 Feeding and Eating Disorder

Nutrition

Table 42: Prevalence of nutrition issues in those with ASD, table 1.

Source	Sample	Prevalence	Other Notes
Croen et al. (2015) [23]	n=1507 (ASD), n=15070 (age, sex matches controls); Adults; Kaiser Permanente Northern California; 2008-12	Nutrition conditions, any: 37.23% (ASD), 18.72% (non-ASD)	Chi-square p value: <0.001
Croen et al. (2015) [23]	n=1507 (ASD), n=15070 (age, sex matches controls); Adults; Kaiser Permanente Northern California; 2008-12	Symptom concerning nutrition metabolism and development: 34.77% (ASD), 17.31% (non-ASD)	Chi-square p value: <0.001
Croen et al. (2015) [23]	n=1507 (ASD), n=15070 (age, sex matches controls); Adults; Kaiser Permanente Northern California; 2008-12	Vitamin deficiency: 4.98% (ASD), 2.28% (non-ASD)	Chi-square p value: <0.001
Vohra et al. (2017) [123]	n=1772 (ASD), n=5316 (non-ASD); Age: 22-64; three state Medicaid Analytic eXtract; 2000-08	Nutritional deficiency: 0.8% (ASD), 0.8% (non-ASD)	aOR (95% CI): 1.07 (0.59, 1.94), p: NS
Vohra et al. (2017) [123]	n=1772 (ASD), n=5316 (non-ASD); Age: 22-64; three state Medicaid Analytic eXtract; 2000-08	fluid or electrolyte dysfunction: 4.7% (ASD), 3.2% (non-ASD)	aOR (95% CI): 1.53 (1.17, 2.01), $0.001 \leq p < 0.01$
Bishop-Fitzpatrick et al. (2018) [11]	n=91 (ASD), n=6186 (sex, birth year matched controls); Age: 0-89; EHR from Marshfield clinic - decedents - Wisconsin	Fluid or electrolyte disorder: 61.5% (ASD), 37.3% (non-ASD)	aOR: 2.79 (adjusted for age and sex), p: <0.001
Davignon, et al. (2018) [25]	n=4123 (ASD), n=20615 (non-ASD, non-ADHD); Age: 14-25; Kaiser Permanente Northern California, 2013-15	Nutrition: 5.2% (ASD), 1.9% (non-ASD)	aOR (95% CI): 2.84 (2.38-3.38)

Table 43: Prevalence of nutrition issues in those with ASD, table 2.

Source	Sample	Prevalence	Other Notes
Smith DaWalt et al. (2021) [26]	n=2187 (ASD) [n=1716 (male), n=471 (female)], n=21870 (non-ASD, age and sex matched) [n=17160 (male), n=4710 (female)]; Age:>18; EHRs - Marshfield Clinic - Wisconsin; ICD-9 codes	Nutrition conditions: 0.66 (female, ASD), 0.57 (male,ASD), 0.21 (female, control), 0.16 (male, control) [predicted probability]	OR: 7.02 (95% CI: 6.32-7.79), p:<0.001 [ASD status], OR: 1.03 (95% CI: 0.82-1.29) [ASD X Sex]
Schott et al. (2022) [105]	n=155,617 (ASD), n=466,851 (non-ASD); Adults; Medicaid; 2008–12	Nutrition condition: 35.23% (ASD), 26.49% (non-ASD)	aOR: 1.38 (1.35 1.40)
Schott et al. (2022) [105]	n=155,617 (ASD), n=466,851 (non-ASD); Adults; Medicaid; 2008–12	Symptom concerning nutrition metabolism: 32.03% (ASD), 23.46% (non-ASD)	aOR: 1.38 (1.36 1.41)
Schott et al. (2022) [105]	n=155,617 (ASD), n=466,851 (non-ASD); Adults; Medicaid; 2008–12	Vitamin deficiency: 8.80% (ASD), 7.60% (non-ASD)	aOR: 1.13 (1.10 1.16)
Micai et al. (2023) [82]	Meta-analysis of observational studies, N=3 [N=2 (clinical studies from community samples), N=1 (population/registry based)], n=6038	Organic Nutrition Disorder: Pooled prevalence: 22%, Range: 0% - 76%	
Agebjörn et al. (2025) [1]	n=63 (ASD or subthreshold ASD), n=27 (non-ASD); Age: 18-66; Psychiatric outpatients; MINI to evaluate PTSD; 2019-20	PTSD: 21% (ASD), 4% (non-ASD)	p: 0.056

Table 44: Prevalence of ASD in those nutrition issues.

Source	Sample	Prevalence	Other Notes
Reikersdorfer et al. (2024) [92]	n=265; Age: <18; National Inpatient Sample database; 2016-20	ASD: 62.2% (Scurvy)	

Eating

Table 45: Prevalence of eating disorders, general and specific, in those with ASD, table 1.

Source	Sample	Prevalence	Other Notes
Koch et al. (2015) [62]	n= 12606 (ASD), n=5006 (non-ASD), n=1724189 (total); Denmark population study; study end: 2012	0.8% (Anorexia in ASD), 1.8% (ASD in Anorexia)	Primary anorexia nervosa, risk of secondary ASD HR (95% CI): 15.08 (12.23-18.58) (all), 22.04 (15.39-31.58) (males), 12.82 (9.94-16.53) (females); Primary ASD, risk of secondary anorexia nervosa: 5.39 (4.37-6.64) (all). Other probands examined and details available
Lever and Geurts (2016) [68]	n=138 (ASD), n=170 (non-ASD); Age: 19-79; Mental Health Institutions and online recruitment; Netherlands	Eating disorders: 5.8% (ASD), 0.6% (non-ASD)	p-value:$\leq$0.5
Margari et al. (2019) [77]	n=100 (ASD, male), n=59 (ASD, female)	Anorexia nervosa: 1% (ASD, male), 6.8% (ASD, female)	p: 0.004
Hossain et al. (2020) [43]	Umbrella Review - N=2	Eating disorders: 1.4% - 7.9%	
Bowden et al. (2020) [15]	n=8955 (ASD), n=1551342 (non-ASD); Age: 0-24; IDI database, New Zealand	Eating: 0.8% (ASD), 0.2% (non-ASD)	aOR: 6.5 (95% CI: 5.0-7.9) - standardized for age, sex, ethnicity, deprivation, and urban/rural
Rødgaard et al. (2021) [100]	n=11,914 (ASD, men), n=4212 (ASD, women); Age $\leq$16; Danish National Patient Registry	Eating disorders 1% (men with ASD), 7% (women with ASD)	
Martini et al. (2022) [79]	n=20841 (ASD) [n=13712 (male, ASD), n=7129 (female, ASD)], n=1314912 (non-ASD) [n=671727 (male, non-ASD), n=643185 (female, non-ASD)]; Age: 16-24; Multiple national registers; Sweden; 2001-13	Anorexia nervosa: 1.1% (ASD, total), 0.4% (non-ASD, total); 0.2% (ASD, male), 2.8% (ASD, female), 0.0% (non-ASD, male), 0.8% (non-ASD, female)	Male vs female: HR: 0.48 (95% CI: 0.19-1.22), p:.12 - adjusted for birth year ADHD, ID

Table 46: Prevalence of eating disorders, general and specific, in those with ASD, table 2.

Source	Sample	Prevalence	Other Notes
Martini et al. (2022) [79]	n=20841 (ASD) [n=13712 (male, ASD), n=7129 (female, ASD)], n=1314912 (non-ASD) [n=671727 (male, non-ASD), n=643185 (female, non-ASD)]; Age: 16-24; Multiple national registers; Sweden; 2001-13	Bulimia nervosa: 0.4% (ASD, total), 0.2% (non-ASD, total); 0.1% (ASD, male), 1.0% (ASD, female), 0.0% (non-ASD, male), 0.3% (non-ASD, female)	Male vs female: HR: 0.34 (95% CI: 0.04-2.92), p:0.33 - adjusted for birth year ADHD, ID
Martini et al. (2022) [79]	n=20841 (ASD) [n=13712 (male, ASD), n=7129 (female, ASD)], n=1314912 (non-ASD) [n=671727 (male, non-ASD), n=643185 (female, non-ASD)]; Age: 16-24; Multiple national registers; Sweden; 2001-13	Other eating disorders: 2.3% (ASD, total), 0.7% (non-ASD, total); 0.6% (ASD, male), 5.6% (ASD, female), 0.1% (non-ASD, male), 1.3% (non-ASD, female)	Male vs female: HR: 0.47 (95% CI: 0.26-0.83), p:0.01 - adjusted for birth year ADHD, ID
Jadav and Bal (2022) [52]	n=4657 (ASD); Adults; Online survey, SPARK; 2022	Eating disorder: 10.3%	
Micai et al. (2023) [82]	Meta-analysis of observational studies, N=32 [N=26 (clinical studies from community samples), N=6 (population/registry based)], n=19,233	Feeding and Eating Disorders: Pooled prevalence: 32%, Range: 20% - 36%	
Micai et al. (2023) [82]	Meta-analysis of observational studies, N=14 [N=13 (clinical studies from community samples), N=1 (population/registry based)], n=12,721	Anorexia nervosa, bulimia nervosa, binge eating disorder: Pooled prevalence: 5%, Range: 2% - 10%	
Semovski et al. (2023) [106]	n=2020 (ASD); Age: 12-18; records + interviews; Canada	Eating disorders: 1.1% (ASD)	
Casati et al. (2024) [20]	Narrative Review	ARFID: 20-30%; (ASD); ASD: 8.2%-42% (ARFID)	

Table 47: Prevalence of ASD in those with eating disorders, general and specific.

Source	Sample	Prevalence	Other Notes
Sader et al. (2025) [101]	n=5734 (ASD), n=1708 (ARFID); Meta-analysis of N=21 (N=18 ARFID, N=3 ASD); 2010-24	ARFID: 11.41%	(95% CI = 2.89%–35.76%)
Diemer et al. (2025) [27]	n=1424 (ASD); Adult; SPARK	Eating disorder: 17.28% (total), 13.27% (diagnosed <18), 19.39% (diagnosed >18)	p-value 0.004 (between age groups)
Westwood et al. (2017) [126]	n=60 (anorexia nervosa, women), Age: >18	ADOS-2 clinical cutoff: 23.3%	
Carpita et al. (2020) [19]	N=10 , Review, 1992-2019	Range: 4% - 52.5%	
Boltri and Sapuppo (2021) [13]	Review, N=13	Autistic symptoms: 17.8% - 35% (acute Anorexia Nervosa); 24% - 24.5% (recovered Anorexia Nervosa)	
Sader et al. (2025) [101]	n=5734 (ASD), n=1708 (ARFID); Meta-analysis of N=21 (N=18 ARFID, N=3 ASD); 2010-24	ASD: 16.27%	(95% CI = 8.64%–28.53%)
Lacroix et al. (2025) [64]	Narrative and Quantitative Review; N =4, n= 14594 (ASD, female), n= 28049 (ASD, male); excluded papers with less than 12.5% females	Eating disorders, Pooled prevalence [95% CI]: 9.1% [6.5-12.6] (ASD, female), 1.6% [0.8-3.4] (ASD, male)	p: <0.001, OR: 6.10

1.8 Sleep-Wake Disorders

Table 48: Prevalence of sleep issues in those with ASD, table 1.

Source	Sample	Prevalence	Other Notes
Croen et al. (2015) [23]	n=1507 (ASD), n=15070 (age, sex matches controls); Adults; Kaiser Permanente Northern California; 2008-12	Sleep disorder, any: 17.58% (ASD), 9.60% (non-ASD)	Chi-square p value: <0.001
Elrod et al. (2016) [29]	n=48726 (ASD), n=243810 (non-ASD, age and sex controlled); Age:2-18; TRICARE database; ICD-9; 2000-13	Any sleep disorder: 30.66% (ASD), 13.92% (non-ASD)	p:0.28, RR: 1.97 (95% CI: 1.91-2.02)
Malow et al. (2016) [75]	1518 children age 4-10, 2008-2013, ATN registry, children's sleep habits questionnaire	Sleep diagnoses: 30%, Score $\geq$ 41: 71%	
Davignon, et al. (2018) [25]	n=4123 (ASD), n=20615 (non-ASD, non-ADHD); Age: 14-25; Kaiser Permanente Northern California, 2013-15	Sleep disorder: 6.2% (ASD), 2.0% (non-ASD)	3.13 (2.66-3.68)
Houghton et al. (2017) [44]	n=46943 (ASD, commercial), Truven Health MarketScan Commercial Database, 2014; n=46696 (ASD, Medicaid), Truven Health MarketScan Multi-State Medicaid Database; Age: >3	Sleep disturbances: 5.73% (commercial), 9.42% (medicaid)	
Lai, et al. (2019) [65]	Meta-analysis, N=96	Sleep-wake disorders: 13%	

Table 49: Prevalence of sleep issues in those with ASD, table 2.

Source	Sample	Prevalence	Other Notes
Won et al. (2019) [128]	n=440 (ASD), n=484 (ADHD), n=145 (ASD+ADHD); Age: <18; DBPNet Study; 2011-12	Diagnosed sleep disorder: 12.0% (ASD), 15.3% (ADHD), 16.6% (ASD+ADHD); Sleep disorder or PSG: 13.0% (ASD), 16.1% (ADHD), 18.6% (ASD+ADHD); Sleep disorder, PSG, or sleep medication: 15.5% (ASD), 18.0% (ADHD), 20.7% (ASD+ADHD); Sleep disorder, PSG, sleep medication, or Clonidine: 18.0% (ASD), 19.8% (ADHD), 24.8% (ASD+ADHD)	
Hand et al. (2020) [34]	n=4685 (ASD), n=46850 (non-ASD); Age: $\geq$65; Medicare; 2016-17	Sleep: 1.4% (ASD), 0.7% (non-ASD)	aOR: 2.2(1.7-2.8)
Bowden et al. (2020) [15]	n=8955 (ASD), n=1551342 (non-ASD); Age: 0-24; IDI database, New Zealand	Sleeping: 2.8% (ASD), 1.6% (non-ASD)	aOR: 2.5 (95% CI: 2.2-2.8) - standardized for age, sex, ethnicity, deprivation, and urban/rural
Bougeard et al. (2021) [14]	Review: N=7	Sleep disorder, Range: 2.08% - 72.50%	
Rødgaard et al. (2021) [100]	n=11,914 (ASD, men), n=4212 (ASD, women); Age $\leq$16; Danish National Patient Registry	Sleep disorder: 2% (men with ASD), 2% (women with ASD)	
Smith DaWalt et al. (2021) [26]	n=2187 (ASD) [n=1716 (male), n=471 (female)], n=21870 (non-ASD, age and sex matched) [n=17160 (male), n=4710 (female)]; Age:>18; EHRs - Marshfield Clinic - Wisconsin; ICD-9 codes	Sleep disorder: 0.20 (female, ASD), 0.22 (male,ASD), 0.07 (female, control), 0.07 (male, control) [predicted probability]	OR: 4.12 (95% CI: 3.62-4.68), p:<0.001 [ASD status], OR: 0.75 (95% CI: 0.57-1.00), p: <0.10 [ASD X Sex]

Table 50: Prevalence of sleep issues in those with ASD, table 3.

Source	Sample	Prevalence	Other Notes
Angell et al. (2021) [5]	n=18355 (ASD, girls), n=65145 (ASD, boys); Age: 1-21; healthcare claims data; integrates ICD-9 and ICD-10 codes	Sleep disorders: 31.39% (ASD, girls), 28.04% (ASD, boys)	aOR: 1.17 (95% CI: 1.13-1.21), p:<0.001 - adjusted for age, race, ethnicity, urbanicity, and insurance
Martini et al. (2022) [79]	n=20841 (ASD) [n=13712 (male, ASD), n=7129 (female, ASD)], n=1314912 (non-ASD) [n=671727 (male, non-ASD), n=643185 (female, non-ASD)]; Age: 16-24; Multiple national registers; Sweden; 2001-13	Sleep disorders: 30.7% (ASD, total), 5.1% (non-ASD, total); 27.0% (ASD, male), 37.8% (ASD, female), 4.2% (non-ASD, male), 6.1% (non-ASD, female)	Male vs female: HR: 0.88 (95% CI: 0.79-0.99), p:0.03 - adjusted for birth year ADHD, ID
Jadav and Bal (2022) [52]	n=4657 (ASD); Adults; online survey, SPARK; 2022	Sleep disorder: 26.6%	
Mutluer et al. (2022) [85]	Review - 8 population and EHR studies (age 6-18)	Sleep disorders: 19.7%	
Schott et al. (2022) [105]	n=155,617 (ASD), n=466,851 (non-ASD); Adults; Medicaid; 2008–12	Sleep disorders: 11.92% (ASD), 10.73% (non-ASD)	aOR: 1.00 (0.97 1.02)
Koceski and Trajkovski (2022) [61]	n=72 (ASD), n=75 (non-ASD); Age: 3-24; Rochester Health Status Survey IV, Macedonia; 2019	Sleep problems: 18% (ASD), 0% (non-ASD)	p:0.000
Masi et al. (2022) [80]	n=969 (ASD), n=188 (siblings), n=111 (non-ASD, non-related); Age: 2-17; caregiver filled out CSHQ	CSHQ above clinical cutoff: 73.4% (ASD), 54.3% (siblings), 43.2% (non-ASD, non-related)	
Micai et al. (2023) [82]	Meta-analysis of observational studies, N=29 [N=16 (clinical studies from community samples), N=13 (population/registry based)], n=203287	Sleep-wake disorder: Pooled prevalence: 25%, Range: 18% - 34%	

Table 51: Prevalence of sleep issues in those with ASD, table 4.

Source	Sample	Prevalence	Other Notes
Micai et al. (2023) [82]	Meta-analysis of observational studies, N=26 [N=18 (clinical studies from community samples), N=8 (population/registry based)], n=18482	Sleep-wake problem: Pooled prevalence: 43%, Range: 36% - 50%	
Estes et al. (2023) [30]	age: 6–12, n=250 (ASD), n=114 (non-ASD)	Sleep problems: 84.4% (ASD, females), 65.8% (ASD, males), 44.8% (non-ASD, females), 42.4% (non-ASD, males)	
Semovski et al. (2023) [106]	n=2020 (ASD); Age: 12-18; records + interviews; Canada	Sleep disorders: 1.9% (ASD)	
Khachadourian et al. (2023) [58]	n=42569 (ASD), n=11389 (non-ASD siblings); SPARK; Age: <18 at time of registration; 1999-2019	Sleep disorders, estimated: 21% (ASD), 3% (non-ASD)	p: <0.05
Kennedy et al. (2024) [56]	n=246 (ASD); Connecticut Children's Medical Center ASD Assessment Program electronic records; 2018-19	Sleep dysregulation: 35.4%	
Longo et al. (2024) [71]	n=30258 (ASD), n=60516 (non-ASD); Age: 18-64; IBM Market Scan Commercial Claims and Encounters Database; 2019-20	Sleep disorders: 3.3% (ASD), 1.0% (non-ASD)	aOR: 3.5 (95% CI: 3.2, 3.9)
Diemer et al. (2025) [27]	n=1424 (ASD); Adult; SPARK	Sleep diagnosis: 31.81% (total), 33.47% (diagnosed <18), 30.87% (diagnosed >18)	p-value 0.317 (between age groups)

Table 52: Prevalence of sleep issues in those with ASD, table 5.

Source	Sample	Prevalence	Other Notes
Toman et al. (2025) [115]	n=240 (ASD); Al-Subtain Academy, Imam Hussein Centre, Baghdad's National Centre; 2024	≥1 sleep issue: 74.2%; Difficulty falling asleep: 40.4%, Sleepwalking: 10.8%, Waking up during the early morning: 31.3%, Problems arising during the morning: 14.2%, Day-time sleepiness: 7.1%, Sleep-onset associated problems (ie co-sleeping, etc): 32.5%; Difficulty breathing or snoring during sleep: 16.7%, Teeth grinding: 12.1%, Confusional arousal: 14.2%, Behavioral disorders during sleep: 19.2%	
Soke et al. (2018) [108]	n=783 (ASD, age: 4), n=1091 (ASD, age; 8), ADDMN - 5 sites (Arizona, Missouri, New Jersey, Utah, and Wisconsin), 2010	Sleep abnormalities: 26.95% (ASD, age: 4), 37.12% (ASD, age: 8)	Adjusted prevalence, 95% CI: 1.34 (1.15, 1.56) [8 vs 4 years] - adjusted for sex, ethnicity, maternal education, location

1.8.1 Dyssomnia

Table 53: Prevalence of dyssomnia in those with ASD.

Source	Sample	Prevalence	Other Notes
Croen et al. (2015) [23]	n=1507 (ASD), n=15070 (age, sex matches controls); Adults; Kaiser Permanente Northern California; 2008-12	Dyssomnia: 15.66% (ASD), 7.85% (non-ASD)	Chi-square p value: <0.001
Davignon, et al. (2018) [25]	n=4123 (ASD), n=20615 (non-ASD, non-ADHD); Age: 14-25; Kaiser Permanente Northern California, 2013-15	Dyssomnia: 5.2% (ASD), 1.7% (non-ASD)	aOR (95% CI): 2.98 (2.50-3.56)
Schott et al. (2022) [105]	n=155,617 (ASD), n=466,851 (non-ASD); Adults; Medicaid; 2008–12	Dyssomnia: 10.18% (ASD), 8.65% (non-ASD)	aOR: 1.05 (1.02 1.07)

1.8.2 Sleep-Disordered Breathing

Table 54: Prevalence of issues breathing during sleep in those with ASD.

Source	Sample	Prevalence	Other Notes
Croen et al. (2015) [23]	n=1507 (ASD), n=15070 (age, sex matches controls); Adults; Kaiser Permanente Northern California; 2008-12	Organic sleep apnea: 4.25% (ASD), 2.68% (non-ASD)	Chi-square p value: 0.005
Elrod et al. (2016) [29]	n=48726 (ASD), n=243810 (non-ASD, age and sex controlled); Age:2-18; TRICARE database; ICD-9; 2000-13	Sleep-disordered breathing: 9.26% (ASD), 4.07% (non-ASD)	p: <0.01, RR: 1.96 (95% CI: 1.88-2.05)
Tomkies et al. [116]	n=45 (ASD); Age: 2-18; EHR from Children's Medical Center Sleep Disorders Center of Dallas; 2009-15	Obstructive sleep apnea: 58%	
Dizitzer et al. (2020) [28]	n=459 (ASD), n=2285 (non-ASD); Children, southern Israel, Negev Autism Center database, born 2009-16, controls age, sex, ethnicity matched	Sleep apnea: 1.1% (ASD), 0.8% (non-ASD)	OR: 1.39 (95% CI: 0.51-3.76), p:0.518
Chen et al. (2021) [21]	n=1310 (ASD), n=1158 (non-ASD); Age: 2-7; China Multi-Center Pre-school Autism Project; 2018-19; Children's sleep habits questionnaire (CSHQ)	Sleep Disordered Breathing: 5.3% (ASD), 1.3% (non-ASD)	p:<0.001
Schott et al. (2022) [105]	n=155,617 (ASD), n=466,851 (non-ASD); Adults; Medicaid; 2008–12	Organic sleep apnea: 5.25% (ASD), 5.99% (non-ASD)	aOR: 0.80 (0.77 0.82)
Scarpelli et al. (2024) [103]	n=242 (ASD); Age 2-17; Child and Adolescent Neuropsychiatry Unit of the Bambino Gesù Children's Hospital; Rome; 2021-22; Sleep Disturbance Scale for Children	Sleep breathing disorders: 14.9% (ASD)	

1.8.3 Other Sleep Disorders

Table 55: Prevalence of other sleep issues in those with ASD, table 1.

Source	Sample	Prevalence	Other Notes
Elrod et al. (2016) [29]	n=48726 (ASD), n=243810 (non-ASD, age and sex controlled); Age:2-18; TRICARE database; ICD-9; 2000-13	Circadian rhythm disorder: 0.62% (ASD), 0.09% (non-ASD)	p:<0.01, RR: 5.92 (95% CI: 4.94-7.10)
Elrod et al. (2016) [29]	n=48726 (ASD), n=243810 (non-ASD, age and sex controlled); Age:2-18; TRICARE database; ICD-9; 2000-13	Hypersomnia: 0.75% (ASD), 0.17% (non-ASD)	p: 0.69, RR: 3.96 (95% CI: 3.42-4.59)
Elrod et al. (2016) [29]	n=48726 (ASD), n=243810 (non-ASD, age and sex controlled); Age:2-18; TRICARE database; ICD-9; 2000-13	Narcolepsy: 0.22% (ASD), 0.04% (non-ASD)	p: 0.49, RR: 4.82 (95% CI: 3.62-6.40)
Elrod et al. (2016) [29]	n=48726 (ASD), n=243810 (non-ASD, age and sex controlled); Age:2-18; TRICARE database; ICD-9; 2000-13	Parasomnias: 1.52% (ASD), 0.51% (non-ASD)	p: 0.99, RR: 2.56 (95% CI: 2.33-2.82)
Chen et al. (2021) [21]	n=1310 (ASD), n=1158 (non-ASD); Age: 2-7; China Multi-Center Pre-school Autism Project; 2018-19; Children's sleep habits questionnaire (CSHQ)	Parasomnia: 7.4% (ASD), 4% (non-ASD)	p:<0.001
Scarpelli et al. (2024) [103]	n=242 (ASD); Age 2-17; Child and Adolescent Neuropsychiatry Unit of the Bambino Gesù Children's Hospital; Rome; 2021-22; Sleep Disturbance Scale for Children	Parasomnias: 20.2%	

Table 56: Prevalence of other sleep issues in those with ASD, table 2.

Source	Sample	Prevalence	Other Notes
Elrod et al. (2016) [29]	n=48726 (ASD), n=243810 (non-ASD, age and sex controlled); Age:2-18; TRICARE database; ICD-9; 2000-13	Restless limb syndrome/periodic limb movement disorder: 0.69% (ASD), 0.13% (non-ASD)	p: 0.20, RR: 4.73 (95% CI: 4.02-5.56)
Elrod et al. (2016) [29]	n=48726 (ASD), n=243810 (non-ASD, age and sex controlled); Age:2-18; TRICARE database; ICD-9; 2000-13	Sleep disorder, not otherwise specified: 21.72% (ASD), 10.77% (non-ASD)	p: 0.22, RR: 1.78 (95% CI: 1.73-1.84)
Elrod et al. (2016) [29]	n=48726 (ASD), n=243810 (non-ASD, age and sex controlled); Age:2-18; TRICARE database; ICD-9; 2000-13	Insomnia: 9.52% (ASD), 1.58% (non-ASD)	p:<0.01, RR: 5.40 (95% CI: 5.14-5.67)
Ivanović (2021) [51]	n=152 (ASD); Mean age: 8.02 (4.26); Autism Center, Montenegro	Insomnia: 7.89%	
Berloco et al. (2024) [10]	n=270 (ASD); Age: 1.8-9.6; Child and Adolescent Neuropsychiatry Unit of Bambino Gesu`	Chronic insomnia: 32%	
Chen et al. (2021) [21]	n=1310 (ASD), n=1158 (non-ASD); Age: 2-7; China Multi-Center Pre-school Autism Project; 2018-19; Children's sleep habits questionnaire (CSHQ)	Bedtime Resistance: 25.6% (ASD), 15.9% (non-ASD)	p:<0.001
Chen et al. (2021) [21]	n=1310 (ASD), n=1158 (non-ASD); Age: 2-7; China Multi-Center Pre-school Autism Project; 2018-19; Children's sleep habits questionnaire (CSHQ)	Sleep Onset Delay: 17.9% (ASD), 12.8% (non-ASD)	p:<0.001

Table 57: Prevalence of other sleep issues in those with ASD, table 3.

**Source**	**Sample**	**Prevalence**	**Other Notes**
Chen et al. (2021) [21]	n=1310 (ASD), n=1158 (non-ASD); Age: 2-7; China Multi-Center Pre-school Autism Project; 2018-19; Children's sleep habits questionnaire (CSHQ)	Sleep Duration: 9.4% (ASD), 6% (non-ASD)	p: <0.001
Chen et al. (2021) [21]	n=1310 (ASD), n=1158 (non-ASD); Age: 2-7; China Multi-Center Pre-school Autism Project; 2018-19; Children's sleep habits questionnaire (CSHQ)	Sleep Anxiety: 22.7% (ASD), 15.7% (non-ASD)	p:<0.001
Chen et al. (2021) [21]	n=1310 (ASD), n=1158 (non-ASD); Age: 2-7; China Multi-Center Pre-school Autism Project; 2018-19; Children's sleep habits questionnaire (CSHQ)	Night Waking: 5% (ASD), 1.5% (non-ASD)	p:<0.001
Chen et al. (2021) [21]	n=1310 (ASD), n=1158 (non-ASD); Age: 2-7; China Multi-Center Pre-school Autism Project; 2018-19; Children's sleep habits questionnaire (CSHQ)	Daytime Sleepiness: 14.7% (ASD), 6% (non-ASD)	p:<0.001

Table 58: Prevalence of other sleep issues in those with ASD, table 4.

Source	Sample	Prevalence	Other Notes
Scarpelli et al. (2024) [103]	n=242 (ASD); Age 2-17; Child and Adolescent Neuropsychiatry Unit of the Bambino Gesù Children's Hospital; Rome; 2021-22; Sleep Disturbance Scale for Children	Excessive somnolence: 22.7%	
Scarpelli et al. (2024) [103]	n=242 (ASD); Age 2-17; Child and Adolescent Neuropsychiatry Unit of the Bambino Gesù Children's Hospital; Rome; 2021-22; Sleep Disturbance Scale for Children	Hyperhidrosis: 24.0%	
Scarpelli et al. (2024) [103]	n=242 (ASD); Age 2-17; Child and Adolescent Neuropsychiatry Unit of the Bambino Gesù Children's Hospital; Rome; 2021-22; Sleep Disturbance Scale for Children	Disorders of initiating and maintaining sleep: 41.7%	
Gupta et al. (2023) [33]	Review, N=65	Sleep disturbances: 64%-93%	

1.9 Self-harm Related Conditions

Self-Injurious Behaviors/Self-Harm

Table 59: Prevalence of self-harm and SIB in those with ASD, table 1.

Source	Sample	Prevalence	Other Notes
Rattaz et al. (2015) [91]	n=152 (ASD); Age: recruited 3-7 - followed 10 yrs; subset of French EpiTED cohort; 1997-2009	SIB: 35.8%	Main risk: ASD symptom severity (P=0.04). Main protective factor: cognitive development (P=0.03) [childhood], communicative abilities (P=0.04) [adolescence]
Akram et al. (2017) [2]	n=83 (ASD), age 8-18, 5 special schools, Lahore City (Pakistan), parental report	Non-suicidal self-injury: 33%	
Richards et al. (2017) [94]	n=208 (ASD, children), n=216 (ASD, adults); Age: 6-61; Questionairre data; NAS schools and adult services; UK	SIB: 45.7% (ASD, child), 49.1% (ASD, adult)	p: 0.513
Soke et al. (2018) [108]	n=783 (ASD, age: 4), n=1091 (ASD, age; 8), ADDMN - 5 sites (Arizona, Missouri, New Jersey, Utah, and Wisconsin), 2010	Self-injurious behaviors: 24.52% (ASD, age: 4), 25.02% (ASD, age: 8)	Adjusted prevalence, 95% CI: 0.94 (0.79, 1.12) [8 vs 4 years] - adjusted for sex, ethnicity, maternal education, location
Culpin et al. (2018) [24]	n=5031 (ASD); age: 16; UK; Avon Longitudinal Study of Parents and Children	Self-harm without suicidal intent: 11.9%	
Hunsche et al. (2020) [50]	n=178 (ASD); Age: 7-11; parental report - child behavior checklist	Suicidal and/or self-injurious behavior: 14.6%	
Laverty et al. (2020) [67]	n=67 (ASD), mean age at start: 13.4 years; 10 year longitudinal study; questionnaire filled out by carer; National Autistic Society	Persistent self-injurious behavior: 44%	
Licence et al. (2020) [69]	n=83 (ASD); age: 4-45; questionnaire filled out by parents; recruited via National Autistic Society	Self-harm: 24.10% (ASD)	

Table 60: Prevalence of self-harm and SIB in those with ASD, table 2.

Source	Sample	Prevalence	Other Notes
Steenfeldt-Kristensen et al. (2020) [110]	N=37, n=14379; meta-analysis	Self-injury: 42% (0.38-0.47)	
Blanchard et al. (2021) [12]	Meta-analysis, N=17	Self-injurious behaviors: not reported	OR, range: 1.21-18.76, OR, pooled: 3.18 (95% CI: 2.45-4.12); Children: 2.99 (1.93-4.64); Adults: 3.38 (2.54-4.50)
Hosozawa et al. (2021) [42]	n=396 (ASD) [n=61 (age 5), n=65 (age 7), n=138 (age 11), n=132 (age 14)], n=11307 (non-ASD); UK Millennium Cohort Study; questionnaire and self-report; born 2000-02	Self-harming behavior: 15.2% (ASD, diagnosed age 5), 23.8% (ASD, diagnosed age 7), 14.8% (ASD, diagnosed age 11), 29% (ASD, diagnosed age 14), 15% (non-ASD)	p: 0.03 (linear trend, unadjusted occurrence); SexaOR, 95% CI: 1 (ref) [no ASD], 1.8 (0.76-4.22) [ASD, diagnosed age 5], 2.58 (1.20-5.59) [ASD, diagnosed age 7], 1.45 (0.80-2.64) [ASD, diagnosed age 11], 3.33 (1.92-5.77) [ASD, diagnosed age 14]
Jokiranta-Olkoniemi et al. (2021) [53]	n=4695 (ASD), n=18450 (non-ASD); matched 1:4 by age, sex, location; The Finnish Prenatal Study of Autism Spectrum disorders (FIPS-A) register study; born: 1987-2005 - diagnosed with ASD by 2007	Intentional self-harm: 0.4% (ASD, total), 0.2% (non-ASD, total); 0.3% (ASD, male), 0.1% (non-ASD, male), 0.7% (ASD, female), 0.2% (non-ASD, female); 0.4% (no ID,ASD), 0.2% (no ID, non-ASD), NS% (ID, ASD), NS% (ID, non-ASD)	Unadjusted: HR: 2.7 (95% CI: 1.5-5.1), p:0.0015 [ASD vs non-ASD]; HR: 2.4 (95% CI: 1.1-5.1), p:0.016 [Male, ASD vs non-ASD]; HR: 3.1 (95% CI: 1.1-8.3), p:0.0255 [Female, ASD vs non-ASD]; HR: 2.8 (95% CI: 1.5-5.2), p:0.0012 [No ID, ASD vs non-ASD]; HR: 1.3 (95% CI: 0.1-12.8), p:0.8050 [ID, ASD vs non-ASD]
Malhi and Sankhyan (2021) [74]	n=1252 (ASD); Age: <18; Diagnosed ASD 1997-2018	SIB: 22.1%	

Table 61: Prevalence of self-harm and SIB in those with ASD, table 3.

Source	Sample	Prevalence	Other Notes
Widnall et al. (2022) [127]	n=2026 (ASD, male), n=437 (ASD, female), n=54552 (non-ASD, male), n=56271 (non-ASD, female); Age: 11-17; South London; National Pupil Database; January 2009-March 2013	Self-harm presentation to ED: 0.5% (ASD, male), <2.3% (ASD, female), 0.2% (non-ASD, male), 1.1% (non-ASD, female)	
Stark et al. (2022) [109]	n=9070 (ASD, all) [n=6753 (ASD, no ID), n=2317 (ASD, ID)], n=402,662 (non-ASD); Stockholm Youth Cohort	Any admission for self-harm: 2.97 % (ASD, all), 0.76% (non-ASD); 3.55% (ASD, no ID), 1.25% (ASD, ID)	RR: 5.88 (95% CI 5.20-6.64) [All ASD], 7.08 (95% CI: 6.23-8.04) [ASD no ID), 2.22 (95% CI: 1.55-3.19) [ASD with ID]; Age and sex adjusted
Lai et al. (2023) [66]	n=379630 (all), n=19800 (ASD, female), n=56126 (ASD, male), n=79200 (non-ASD, female), n=224504 (non-ASD, male); age and sex matched; Emergency health care records - multiple databases; Canada; April 2005-December 2020		Crude hazard ratio 1.83 (95% CI 1.61-2.08) [females, ASD vs non-ASD], 1.47 (95% CI 1.28-1.69) [males, ASD vs non-ASD]
Stewart et al. (2023) [111]	n=276 (AS Traits) [only 7.6% ASD diagnosis], n=10495 (controls, age/sex matched); Age: 50-81; PROTECT study; UK	Self harm, with or without suicidal intent: 19.9% (AS Traits), 4.7% (non-ASD); Self-injury: 9.7% (AS Traits), 2.3% (non-ASD); Ingesting medicine in excess of normal dose: 12.7% (AS Traits), 2.7% (non-ASD); Abusing alcohol or non-prescription drugs: 4.0% (AS Traits), 0.5% (non-ASD); Swallowing dangerous objects/substances: 0.7% (AS Traits), 0.1% (non-ASD); Stopping medication without medical advice: 2.2% (AS Traits), 0.2% (non-ASD)	p:<0.001; p:<0.001; p:<0.001; p:<0.001; p:<0.001; p:<0.001

Table 62: Prevalence of self-harm and self-injurious behavior in those with ASD, table 4.

Source	Sample	Prevalence	Other Notes
Stewart et al. (2023) [111]	n=276 (AS Traits) [only 7.6% ASD diagnosis], n=10495 (controls, age/sex matched); Age: 50-81; PROTECT study; UK	Number of self-harm behaviors: 82.5% (AS Traits), 95.5% (non-ASD) [None], 9.5% (AS Traits), 3.5% (non-ASD) [One], 5.8% (AS Traits), 0.7% (non-ASD) [Two], 2.2% (AS Traits), 0.3% (non-ASD) [3+]	
Hull et al. (2024) [49]	n=85143 (ASD) [n=31288 female, n=53855 male], n=2628382 (non-ASD) [n=1286481 female, n=1341901 male]; Age: 12-37; Sweden; Population registrar; age 12 - December 2021	Self-harm: 6.2% (ASD), 1.4% (non-ASD)	
Lopez-Arvizu et al. (2025) [72]	n=41230 (ASD, ID, or both), n=29001 (ASD), n=27054 (ID); Age: 5-24; Medicaid (6+ months, Midwestern states); 2010-20	Deliberate self-harm: 2.6% (ASD), 2.7% (ID), % (combined)	ASD: HR 1.44 [95% CI 1.23-1.69] (female vs male), older age (vs 5-9 years; 10-14 years: HR 3.71 [95% CI 2.74-5.03]; 15-19 years: HR 4.80 [95% CI 3.50-6.59]; 20-24 years: HR 3.26 [95% CI 2.27-4.71] - other demographics available. ID: HR 1.61 [95% CI 1.38-1.88] (female vs male) and older age (vs 5-9 years: 10-14 years: HR 3.17 [95% CI 2.28-4.41]; 15-19 years: HR 3.98 [95% CI 2.83-5.58]; 20-24 years: HR 2.94 [95% CI 2.02-4.27]

Suicide

Table 63: Prevalence of suicidal thoughts and behaviors in those with ASD, table 1.

Source	Sample	Prevalence	Other Notes
Croen et al. (2015) [23]	n=1507 (ASD), n=15070 (age, sex matches controls); Adults; Kaiser Permanente Northern California; 2008-12	Suicide attempts: 1.79% (ASD), 0.32% (non-ASD)	Chi-square p value: <0.0001
Hirvikoski et al. (2016) [37]	n=27122 (ASD), n=2672185 (non-ASD) [n=6240 (low functioning), n=20882 (high-functioning)];National Patient Register; Sweden; diagnosed: 1987-2009; ICD-10 conversion	Suicide: 0.31% (ASD, all), 0.04% (non-ASD); 0.11% (low-functioning ASD), 0.36% (high-functioning ASD)	OR (95% CI): 7.55 (6.04-9.44) [ASD all vs non-ASD], 2.41 (1.14-5.11) [low-functioning ASD], 9.40 (7.43-11.90) [high-functioning ASD]
Culpin et al. (2018) [24]	n=5031 (ASD); age: 16; UK; Avon Longitudinal Study of Parents and Children	Self-harm with suicidal intent: 6.9%; Suicidal thoughts: 15.7%; Suicidal plans: 4.5%	
Kirby et al. (2019) [59]	n=5202 (ASD), n=1928484 (non-ASD) [1998-2002], n=8722 (ASD), n=2126249 (non-ASD) [03-07], n=13890 (ASD), n=2391862 (non-ASD) [08-12], n=16907 (ASD), n=2630221 (non-ASD) [13-17]; URADD statewide autism surveillance data, statewide suicide surveillance data - Utah Office of the Medical Examiner, UPDB, IBIS-PH; Utah; 1998-2017	Cases/Total: 2/5202 (ASD), 1617/1928484 (non-ASD) [1998-2002], 5/8722 (ASD), 1841/2126249 (non-ASD) [03-07], 14/13890 (ASD), 2529/2391862 (non-ASD) [08-12], 28/16907 (ASD), 2791/2630221 (non-ASD) [13-17]; Incidence (95% CI): 0.04% (0.01-0.15) (ASD), 0.08% (0.08-0.09) (non-ASD) [1998-2002], 0.06% (0.02-0.14) (ASD), 0.09% (0.08-0.09) (non-ASD) [03-07], 0.10% (0.06-0.17) (ASD), 0.11% (0.10-0.11) (non-ASD) [08-12], 0.17% (0.11-0.24) (ASD), 0.11% (0.10-0.11) (non-ASD) [13-17]	ASD vs non-ASD RR (95% CI): 0.46 (0.11-1.83) [1998-2002], 0.66 (0.28-1.59) [03-07], 0.95 (0.56-1.61) [08-12], 1.56 (1.08-2.26) [13-17]l

Table 64: Prevalence of suicidal thoughts and behaviors in those with ASD, table 2.

Source	Sample	Prevalence	Other Notes
Hossain et al. (2020) [43]	Umbrella Review - N=5	Ideation: 10.9%-66%, attempts: 1% - 35%	
Hand et al. (2020) [34]	n=4685 (ASD), n=46850 (non-ASD); Age: $\geq$65; Medicare; 2016-17	Suicide or intentional self injury: 3.6% (ASD), 0.3% (non-ASD)	aOR: 11.1(8.9-13.8)
Hand et al. (2020) [35]	n=21792 (ASD); Age: 18-59; US; Medicare, National; U.S. Medicare Limited Data Set Standard Analytic Files (SAF); 2012-17	Suicidal ideation: 4.32%; Suicide attempt/Self-inflicted injury: 4.14%	
Holden et al. (2020) [40]	n=680 (ASD); Age: 13-18; South London and Maudsley NHS Foundation; 2008-13; longitudinal study - followed until age 18 or June 2016	Suicidality: 19% (ASD)	
Hunsche et al. (2020) [50]	n=178 (ASD); Age: 7-11; parental report - child behavior checklist	Suicidal ideation: 9.6%	
Pelton et al. (2020) [90]	n=350 (ASD),n=339 (non-ASD); Age: 18-90; online survey	Past suicidal thoughts: 16.1% (ASD), 35.9% (non-ASD); Past suicidal plans: 40.9% (ASD), 21.9% (non-ASD); Past suicide attempt: 38.3% (ASD), 10.5% (non-ASD)	

Table 65: Prevalence of suicidal thoughts and behaviors in those with ASD, table 3.

Source	Sample	Prevalence	Other Notes
Hirvikoski et al. (2020) [38]	n=24535 (ASD, no ID, no ADHD), n=122675 (non-ASD, no ID, no ADHD), n=19035 (ASD, no ID, ADHD), n=95175 (non-ASD, no ID, ADHD), n=7704 (ASD+ID), n=38520 (non-ASD+ID), n=2894 (ASD+ID+ADHD), n=14470 (non-ASD+ID+ADHD); Swedish national registers; 1987-2013; diagnoses converted to ICD-10	Attempted suicide (all): 8.42% (ASD, no ID, no ADHD), 2.17% (non-ASD, no ID, no ADHD), 12.59% (ASD, no ID, ADHD), 2.05% (non-ASD, no ID, ADHD), 5.88% (ASD, ID, no ADHD), 2.38% (non-ASD, ID, no ADHD), 9.57% (ASD, ID, ADHD), 1.91% (non-ASD, ID, ADHD); Male: 5.80% (ASD, no ID, no ADHD), 1.98% (non-ASD, no ID, no ADHD), 8.95% (ASD, no ID, ADHD), 1.78% (non-ASD, no ID, ADHD), 5.03% (ASD, ID, no ADHD), 2.19% (non-ASD, ID, no ADHD), 7.56% (ASD, ID, ADHD), 1.71% (non-ASD, ID, ADHD); Female: 13.98% (ASD, no ID, no ADHD), 2.57% (non-ASD, no ID, no ADHD), 20.39% (ASD, no ID, ADHD), 2.62% (non-ASD, no ID, ADHD), 7.45% (ASD, ID, no ADHD), 2.74% (non-ASD, ID, no ADHD), 14.34% (ASD, ID, ADHD), 2.40% (non-ASD, ID, ADHD)	OR (95% CI) - adjusted for depression, anxiety, SUD: 1.70 (1.57-1.84) [All, ASD - no ID, no ADHD vs control], 2.31 (2.11-2.53) [All, ASD+ADHD vs controls], 2.00 (1.76-2.27) [All, ASD+ID vs controls], 2.90 (2.36-3.57) [All, ASD+ID+ADHD vs controls]; 1.47 (1.32-1.63) [Male, ASD - no ID, no ADHD vs control], 2.03 (1.80-2.29) [Male, ASD+ADHD vs controls], 2.03 (1.72-2.40) [Male, ASD+ID vs controls], 3.20 (2.47-4.14) [Male, ASD+ID+ADHD vs controls]; 2.08 (1.84-2.36) [Female, ASD - no ID, no ADHD vs control], 2.83 (2.45-3.26) [Female, ASD+ADHD vs controls], 1.97 (1.61-2.40) [Female, ASD+ID vs controls], 2.46 (1.73-3.49) [Female, ASD+ID+ADHD vs controls]

Table 66: Prevalence of suicidal thoughts and behaviors in those with ASD, table 4.

Source	Sample	Prevalence	Other Notes
Hirvikoski et al. (2020) [38]	n=24535 (ASD, no ID, no ADHD), n=122675 (non-ASD, no ID, no ADHD), n=19035 (ASD, no ID, ADHD), n=95175 (non-ASD, no ID, ADHD), n=7704 (ASD+ID), n=38520 (non-ASD+ID), n=2894 (ASD+ID+ADHD), n=14470 (non-ASD+ID+ADHD); Swedish national registers; 1987-2013; diagnoses converted to ICD-10	Death by suicide: 0.59% (ASD, no ID, no ADHD), 0.07% (non-ASD, no ID, no ADHD), 0.40% (ASD, no ID, ADHD), 0.03% (non-ASD, no ID, ADHD), 0.16% (ASD, ID, no ADHD), 0.07% (non-ASD, ID, no ADHD); Male: 0.62% (ASD, no ID, no ADHD), 0.09% (non-ASD, no ID, no ADHD), 0.43% (ASD, no ID, ADHD), 0.03% (non-ASD, no ID, ADHD), 0.18% (ASD, ID, no ADHD), 0.09% (non-ASD, ID, no ADHD); Female: 0.52% (ASD, no ID, no ADHD), 0.04% (non-ASD, no ID, no ADHD), 0.33% (ASD, no ID, ADHD), 0.02% (non-ASD, no ID, ADHD)	OR (95% CI) - adjusted for depression, anxiety, SUD: 4.46 (3.24-6.13) [All, ASD - no ID, no ADHD vs control], 3.61 (1.94-6.71) [All, ASD+ADHD vs controls], 1.44 (0.70-2.97) [All, ASD+ID vs controls]; 4.68 (3.28-6.67) [Male, ASD - no ID, no ADHD vs control], 3.91 (1.87-8.19) [Male, ASD+ADHD vs controls], 1.32 (0.58-3.03) [Male, ASD+ID vs controls]; 3.85 (1.88-7.91) [Female, ASD - no ID, no ADHD vs control], 3.15 (0.98-10.11) [Female, ASD+ADHD vs controls]
Blanchard et al. (2021) [12]	Meta-analysis, N=16	Suicidality: not reported	OR, range: 0.86-11.10, OR, pooled: 3.32 (95% CI: 2.60-4.24); Children: 2.53 (1.70-3.76); Adults: 3.84 (2.78-5.30)
Kõlves et al. (2021) [63]	n=35020 (ASD), n=6524246 (non-ASD); Age: >10; Denmark; 1995-2016	Suicide attempts: 587/35020 (ASD), 63522/6524246 (non-ASD); Suicide: 53/35020 (ASD), 14144/6524246 (non-ASD)	ASD vs non-ASD, aRR (95% CI): 3.19 (2.93-3.46) [attempt], 3.75 (2.85-4.92) [suicide]. Women vs men - suicide attempt: 4.41 (3.74-5.19) (ASD), 1.41 (1.39-1.43) (non-ASD). ASD only diagnosis, suicide attempt: 1.33 (0.99-1.78). ASD+co-occurring vs those without psychiatric, suicide attempt: 9.27 (8.51-10.10)

Table 67: Prevalence of suicidal thoughts and behaviors in those with ASD, table 5.

Source	Sample	Prevalence	Other Notes
Bal et al. (2022) [7]	3 samples: [KP: n=86 (ASD, diagnosed), n=864 ("ASP", screened ASD, no diagnostic evaluation), n=13473 (non-ASD); Age: 7-12; metropolitan Seoul, South Korea; 2006-09], [CHEER: n=223 (ASP), n=3702 (non-ASD); school aged; cities in South Korea], [S: n=205 (ASP), n=4623 (non-ASD); elementary schools; Sooncheon, South Korea]	Suicidal ideation: 14.0% (ASD, KP), 19.8% (ASP, KP), 5.4% (non-ASD, KP); 27.4% (ASP, CHEER), 6.9% (non-ASD, CHEER); 16.6% (ASP, S), 3.4% (non-ASD, S)	OR (95% CI) - adjusted for demographics, peer victimization, depression, behavior problems [Model 3]: 1.59 (1.29-1.98) p:0.000 (KP: ASP vs non-ASD), 1.95 (1.28-2.97) p:0.002 [CHEER: ASP vs non-ASD], 1.98 (1.22-3.19) p: 0.005 [S: ASP vs non-ASD]
Schott et al. (2022) [105]	n=155,617 (ASD), n=466,851 (non-ASD); Adults; Medicaid; 2008–12	Suicide attempts: 0.89% (ASD), 0.68% (non-ASD)	aOR: 1.10 (1.01 1.20)
Lai et al. (2023) [66]	n=379630 (all), n=17982 (ASD, female), n=48956 (ASD, male), n=71928 (non-ASD, female), n=195824 (non-ASD, male); age and sex matched 1:4 with non-ASD; Emergency health care records - multiple databases; Canada; April 2005-December 2020	Not listed	Crude hazard ratio: 1.98 (95% CI 1.11-3.56) [females, ASD vs non-ASD], 1.34 (95% CI 0.99-1.82) [males, ASD vs non-ASD]
Newell et al. (2023) [86]	N=36, n=48186 (ASD); Systematic review and meta-analysis; No co-occurring intellectual disability	Suicidal ideation: 34.2% (95% CI: 27.9-40.5), Suicide plans: 21.9% (13.4-30.4), Suicidal attempts and behaviors: 24.3% (18.9-29.6)	
Stewart et al. (2023) [111]	n=276 (AS Traits) [only 7.6% ASD diagnosis], n=10495 (controls, age/sex matched); Age: 50-81; PROTECT study; UK	Suicide attempt: 12.7% (AS Traits), 2.5% (non-ASD)	p:<0.001

Table 68: Prevalence of suicidal thoughts and behaviors in those with ASD, table 6.

Source	Sample	Prevalence	Other Notes
Liu et al. (2023) [70]	n=5291 (ASD), n=4195596 (non-ASD); Age: >45; Swedish nationwide Total Population Register and National Patient Register; born 1932-67; cumulative incidence uses 1:10 sex and birth year matched cohort	Intentional self-injury: 4.6% (ASD), 0.7% (non-ASD) [prevalence over follow up period]; 6.1% (ASD), 0.9% (non-ASD) [estimated cumulative incidence by age 70]	aOR: 7.08 (95% CI: 6.24-8.03), p:<0.0001 (ASD vs non-ASD); aOR: 8.09 (95% CI: 7.07-9.25), p:<0.0001 (ASD without ID vs non-ASD);aOR: 3.48 (2.37-5.11), p:<0.0001 (ASD+ID vs non-ASD); p:<0.0001 (ASD without ID vs ASD+ID); aOR: 6.87 (5.81-8.13), p:<0.0001 (men with ASD vs men without ASD);aOR: 7.45 (95% CI: 6.14-.03), p:<0.0001 (women with ASD vs women without ASD); p: 0.54 (men with ASD vs women with ASD) [AORs from cumulative incidence; adjusted for sex and birth year]
Hull et al. (2024) [49]	n=85143 (ASD) [n=31288 female, n=53855 male], n=2628382 (non-ASD) [n=1286481 female, n=1341901 male]; Age: 12-37; Sweden; Population registrar; age 12 - December 2021	Suicide: 0.4% (ASD), 0.13% (non-ASD)	
O'Nions et al. (2024) [89]	n=13,172 (ASD, men, no ID), n=3958 (ASD, women, no ID) n=4850 (ASD+ID, men) n=1600 (ASD+ID, women); comparison are 1:10 matched by age, sex, ID status, general practice registered to; mortality study; UK; 1989-2019	Self harm/suicide: Men: 6.89% (ASD, no ID); 1.93% (comparison). Women: 17.43% (ASD, no ID); 4.68% (comparison). Men: 5.46% (ASD + ID); 2.38% (comparison). Women: 10.00% (ASD + ID); 5.20% (comparison)	

Table 69: Prevalence of suicidal thoughts and behaviors in those with ASD, table 7.

Source	Sample	Prevalence	Other Notes
Longo et al. (2024) [71]	n=30258 (ASD), n=60516 (non-ASD); Age: 18-64; IBM Market Scan Commercial Claims and Encounters Database; 2019-20	Suicide or intentional self injury: 6.5% (ASD), 1.2% (non-ASD)	aOR: 5.7 (95% CI: 5.2, 6.2)

1.10 Neurocognitive Disorders

Parkinson's

Table 70: Prevalence of Parkinsons in those with ASD.

Source	Sample	Prevalence	Other Notes
Croen et al. (2015) [23]	n=1507 (ASD), n=15070 (age, sex matches controls); Adults; Kaiser Permanente Northern California; 2008-12	Parkinson's: 0.93% (ASD), 0.03% (non-ASD)	Chi-square p value: <0.001
Hand et al. (2020) [34]	n=4685 (ASD), n=46850 (non-ASD); Age: $\geq$65; Medicare; 2016-17	Parkinson's: 6.6% (ASD), 1.2% (non-ASD)	aOR: 6.1 (5.3 - 7.0)
Schott et al. (2022) [105]	n=155,617 (ASD), n=466,851 (non-ASD); Adults; Medicaid; 2008–12	Parkinson disease and spectrum: 0.50% (ASD), 0.34% (non-ASD)	aOR: 1.54 (1.37 1.72)
Yin et al. (2025) [129]	n=2278565, Nationwide, population, Sweden, all individuals born 1974-99, Age 20 until end of 2022	Parkinson disease: 0.05% [3.9 cases/100,000 person-years] (ASD), 0.02% [1.3 cases/100,000 person-years] (non-ASD)	RR 4.43 (95% CI, 2.92-6.72)

Dementia

Table 71: Prevalence of dementia in those with ASD.

Source	Sample	Prevalence	Other Notes
Croen et al. (2015) [23]	n=1507 (ASD), n=15070 (age, sex matches controls); Adults; Kaiser Permanente Northern California; 2008-12	Dementia: 2.26% (ASD), 0.50% (non-ASD)	Chi-square p value: <0.0001
Vivanti et al. (2021) [121]	Medicaid, age 30-64, n=38,816 (ASD, no ID), n=798,828 (no ASD, no ID)	Early onset dementia: 4.04% (ASD), 0.97% (no ASD, no ID)	adjusted hazard ratio, 1.96; 95% CI, (1.69–2.28)
Schott et al. (2022) [105]	n=155,617 (ASD), n=466,851 (non-ASD); Adults; Medicaid; 2008–12	Dementia: 1.52% (ASD), 1.17% (non-ASD)	aOR: 1.34 (1.26 1.43)
Tewolde et al. (2025) [112]	n=90,229 (ASD); $\geq$ 30 years; Medicaid and Medicare data	Alzheimer's disease and related dementia: 8.11%	(95% CI: 7.92%, 8.30%)
Vivanti et al. (2025) [122]	n=46877 (ASD), n=67705 (ASD+ID); Medicare and Medicaid data; 2014-16; excluded Down Syndrome	Dementia: 8.03% (ASD), 8.88% (ASD+ID)	

Other

Table 72: Prevalence of other neurocognitive disorders in those with ASD.

Source	Sample	Prevalence	Other Notes
Soke et al. (2018) [108]	n=783 (ASD, age: 4), n=1091 (ASD, age; 8), ADDMN - 5 sites (Arizona, Missouri, New Jersey, Utah, and Wisconsin), 2010	Developmental regression: 19.67% (ASD, age: 4), 19.34% (ASD, age: 8)	Adjusted prevalence, 95% CI: 0.98 (0.80, 1.20) [8 vs 4 years] - adjusted for sex, ethnicity, maternal education, location

References

[1] J. Agebjörn, C. Gillberg, J. Eberhard, E. Billstedt, and J. Nyrenius. Association Between Autism and PTSD Among Adult Psychiatric Outpatients. *Journal of Autism and Developmental Disorders*, 55(9):3343–3353, Sept. 2025. ISSN 1573-3432. doi: 10.1007/s10803-024-06439-7. URL https://doi.org/10.1007/s10803-024-06439-7.

[2] B. Akram, M. Batool, Z. Rafi, and A. Akram. Prevalence and Predictors of Non-Suicidal Self-Injury among Children with Autism Spectrum Disorder. *Pakistan Journal of Medical Sciences*, 33(5):1225–1229, 2017. ISSN 1682-024X. doi: 10.12669/pjms.335.12931. URL https://pmc.ncbi.nlm.nih.gov/articles/PMC5673738/.

[3] L. Y. Al-Ayadhi, N. E. Elamin, A. Madani, F. Al-Ghamdi, H. A. Al-Ghamdi, and D. M. Halepoto. Examining the Prevalence, Characteristics, and Potential Links Between Skin Disorders and Autism Spectrum Disorder (ASD). *Journal of Clinical Medicine*, 14(2):469, Jan. 2025. ISSN 2077-0383. doi: 10.3390/jcm14020469. URL https://www.ncbi.nlm.nih.gov/pmc/articles/PMC11765873/.

[4] Amy Yule, Maura DiSalvo, Joseph Biederman, Timothy E Wilens, Nina T Dallenbach, Daria Taubin, and Gagan Joshi. Decreased Risk for Substance Use Disorders in Individuals with High-functioning Autism Spectrum Disorder - PMC. *European Child & Adolescent Psychiatry*, 32(2):257–265, Aug. 2021. doi: 10.1007/s00787-021-01852-0. URL https://pmc.ncbi.nlm.nih.gov/articles/PMC8936975/.

[5] A. M. Angell, A. Deavenport-Saman, L. Yin, B. Zou, C. Bai, D. Varma, and O. Solomon. Sex Differences in Co-occurring Conditions among Autistic Children and Youth in Florida: A Retrospective Cohort Study (2012-2019). *Journal of autism and developmental disorders*, 51(10):3759–3765, Oct. 2021. ISSN 0162-3257. doi: 10.1007/s10803-020-04841-5. URL https://www.ncbi.nlm.nih.gov/pmc/articles/PMC8254818/.

[6] E. A. Arnevik and S. B. Helverschou. Autism Spectrum Disorder and Co-occurring Substance Use Disorder - A Systematic Review. *Substance Abuse: Research and Treatment*, 10:69–75, 2016. ISSN 1178-2218. doi: 10.4137/SART.S39921.

[7] V. H. Bal, B. L. Leventhal, G. Carter, C. Child, H. Kim, Y.-J. Koh, M. Ha, H.-J. Kwon, P. Hong, and Y. S. Kim. Parent-reported suicidal ideation in three population-based samples of school-aged Korean children with Autism Spectrum Disorder and Autism Spectrum Screening Questionnaire Screen Positivity. *Archives of suicide research : official journal of the International Academy for Suicide Research*, 26(3):1232–1249, 2022. ISSN 1381-1118. doi: 10.1080/13811118.2020.1868367. URL https://pmc.ncbi.nlm.nih.gov/articles/PMC9058040/.

[8] W. Barber, B. Aslan, T. Meynen, J. Marsden, S. R. Chamberlain, V. Paleri, and J. M. A. Sinclair. Alcohol use among populations with autism spectrum disorder: narrative systematic review. *BJPsych Open*, 11(1):e15, Jan. 2025. ISSN 2056-4724. doi: 10.1192/bjo.2024.824. URL `https://www.cambridge.org/core/journals/bjpsych-open/article/alcohol-use-among-populati`

[9] S. A. Bedford, M. C. Hunsche, and C. M. Kerns. Co-occurrence, Assessment and Treatment of Obsessive Compulsive Disorder in Children and Adults With Autism Spectrum Disorder. *Current Psychiatry Reports*, 22(10):53, Aug. 2020. ISSN 1535-1645. doi: 10.1007/s11920-020-01176-x. URL `https://link.springer.com/article/10.1007/s11920-020-01176-x`.

[10] B. Bianca, G. Silvia, F. Elisa, M. Deny, V. Giovanni, N. Lino, and V. Stefano. Insomnia in Children with Autism Spectrum Disorder: A Cross-Sectional Study on Clinical Correlates and Parental Stress. *Journal of Autism and Developmental Disorders*, 54(1):46–55, Jan. 2024. ISSN 1573-3432. doi: 10.1007/s10803-022-05793-8. URL `https://doi.org/10.1007/s10803-022-05793-8`.

[11] L. Bishop-Fitzpatrick, A. Movaghar, J. S. Greenberg, D. Page, L. S. DaWalt, M. H. Brilliant, and M. R. Mailick. Using machine learning to identify patterns of lifetime health problems in decedents with autism spectrum disorder. *Autism Research*, 11(8):1120–1128, Aug. 2018. ISSN 1939-3792. doi: 10.1002/aur.1960. URL `https://onlinelibrary.wiley.com/doi/full/10.1002/aur.1960`.

[12] A. Blanchard, S. Chihuri, C. G. DiGuiseppi, and G. Li. Risk of Self-harm in Children and Adults With Autism Spectrum Disorder: A Systematic Review and Meta-analysis. *JAMA Network Open*, 4(10):e2130272, Oct. 2021. ISSN 2574-3805. doi: 10.1001/jamanetworkopen.2021.30272. URL `https://doi.org/10.1001/jamanetworkopen.2021.30272`.

[13] M. Boltri and W. Sapuppo. Anorexia Nervosa and Autism Spectrum Disorder: A Systematic Review. *Psychiatry Research*, 306:114271, Dec. 2021. ISSN 0165-1781. doi: 10.1016/j.psychres.2021.114271. URL `https://www.sciencedirect.com/science/article/pii/S0165178121005667`.

[14] C. Bougeard, Françoise Picarel-Blanchot, Ramona Schmid, Rosanne Campbell, and Jan Buitelaar. Prevalence of Autism Spectrum Disorder and Co-morbidities in Children and Adolescents: A Systematic Literature Review. *ResearchGate*, Oct. 2021. doi: 10.3389/fpsyt.2021.744709.

[15] N. Bowden, H. Thabrew, J. Kokaua, R. Audas, B. Milne, K. Smiler, H. Stace, B. Taylor, and S. Gibb. Autism spectrum disorder/Takiwātanga: An Integrated Data Infrastructure-based approach to autism spectrum disorder research in New Zealand - Nicholas Bowden, Hiran Thabrew, Jesse Kokaua, Richard Audas, Barry Milne, Kirsten Smiler,

Hilary Stace, Barry Taylor, Sheree Gibb, 2020. *Autism*, 24(8): 2213–2227, 2020. doi: https://doi.org/10.1177/1362361320939329. URL https://journals.sagepub.com/doi/full/10.1177/1362361320939329.

[16] L. Brookman-Frazee, N. Stadnick, C. Chlebowski, M. Baker-Ericzén, and W. Ganger. Characterizing psychiatric comorbidity in children with autism spectrum disorder receiving publicly funded mental health services. *Autism*, 22(8):938–952, Nov. 2018. ISSN 1362-3613, 1461-7005. doi: 10.1177/1362361317712650. URL https://journals.sagepub.com/doi/10.1177/1362361317712650.

[17] J. D. Brooks, S. E. Bronskill, L. Fu, F. E. Saxena, J. Arneja, V. B. Pinzaru, E. Anagnostou, K. Nylen, J. McLaughlin, and K. Tu. Identifying Children and Youth With Autism Spectrum Disorder in Electronic Medical Records: Examining Health System Utilization and Comorbidities. *Autism Research*, 14(2): 400–410, 2021. ISSN 1939-3806. doi: 10.1002/aur.2419. URL https://onlinelibrary.wiley.com/doi/abs/10.1002/aur.2419. _eprint: https://onlinelibrary.wiley.com/doi/pdf/10.1002/aur.2419.

[18] A. Butwicka, N. Långström, H. Larsson, S. Lundström, E. Serlachius, C. Almqvist, L. Frisén, and P. Lichtenstein. Increased Risk for Substance Use-Related Problems in Autism Spectrum Disorders: A Population-Based Cohort Study. *Journal of Autism and Developmental Disorders*, 47(1):80–89, Jan. 2017. ISSN 1573-3432. doi: 10.1007/s10803-016-2914-2. URL https://doi.org/10.1007/s10803-016-2914-2.

[19] B. Carpita, D. Muti, I. M. Cremone, A. Fagiolini, and L. Dell'Osso. Eating disorders and autism spectrum: links and risks. *CNS Spectrums*, 27(3):272–280, June 2022. ISSN 1092-8529, 2165-6509. doi: 10.1017/S1092852920002011. URL https://www.cambridge.org/core/journals/cns-spectrums/article/abs/eating-disorders-and-

[20] L. Casati, T. Prodi, A. Vedani, C. Caruso, C. Gesi, and B. Dell'Osso. The Challenges of Avoidant/Restrictive Food Intake Disorder and Autism Spectrum Disorder Comorbidity: A Narrative Update of Course and Outcomes. *Current Treatment Options in Psychiatry*, 11(4):358–365, Dec. 2024. ISSN 2196-3061. doi: 10.1007/s40501-024-00336-7. URL https://doi.org/10.1007/s40501-024-00336-7.

[21] H. Chen, T. Yang, J. Chen, L. Chen, Y. Dai, J. Zhang, L. Li, F. Jia, L. Wu, Y. Hao, X. Ke, M. Yi, Q. Hong, J. Chen, S. Fang, Y. Wang, Q. Wang, C. Jin, and T. Li. Sleep problems in children with autism spectrum disorder: a multicenter survey. *BMC Psychiatry*, 21:406, Aug. 2021. ISSN 1471-244X. doi: 10.1186/s12888-021-03405-w. URL https://www.ncbi.nlm.nih.gov/pmc/articles/PMC8365936/.

[22] Y.-L. Chien, C.-S. Wu, and H.-J. Tsai. The Comorbidity of Schizophrenia Spectrum and Mood Disorders in Autism Spectrum Disorder. *Autism Research*, 14(3):571–581, 2021. ISSN 1939-3806. doi: 10.1002/aur.2451. URL https://onlinelibrary.wiley.com/doi/abs/10.1002/aur.2451. _eprint: https://onlinelibrary.wiley.com/doi/pdf/10.1002/aur.2451.

[23] L. A. Croen, O. Zerbo, Y. Qian, M. L. Massolo, S. Rich, S. Sidney, and C. Kripke. The health status of adults on the autism spectrum. *Autism*, 19(7):814–823, Oct. 2015. ISSN 1362-3613, 1461-7005. doi: 10.1177/1362361315577517. URL https://journals.sagepub.com/doi/10.1177/1362361315577517.

[24] I. Culpin, B. Mars, R. M. Pearson, J. Golding, J. Heron, I. Bubak, P. Carpenter, C. Magnusson, D. Gunnell, and D. Rai. Autistic Traits and Suicidal Thoughts, Plans, and Self-Harm in Late Adolescence: Population-Based Cohort Study. *Journal of the American Academy of Child & Adolescent Psychiatry*, 57(5):313–320.e6, May 2018. ISSN 0890-8567. doi: 10.1016/j.jaac.2018.01.023. URL https://www.sciencedirect.com/science/article/pii/S0890856718301047.

[25] M. N. Davignon, Y. Qian, M. Massolo, and L. A. Croen. Psychiatric and Medical Conditions in Transition-Aged Individuals With ASD. *Pediatrics*, 141(Supplement_4):S335–S345, Apr. 2018. ISSN 0031-4005. doi: 10.1542/peds.2016-4300K. URL https://doi.org/10.1542/peds.2016-4300K.

[26] L. S. DaWalt, J. L. Taylor, A. Movaghar, J. Hong, B. Kim, M. Brilliant, and M. R. Mailick. Health profiles of adults with ASD: Differences between women and men. *Autism research : official journal of the International Society for Autism Research*, 14(9):1896–1904, Sept. 2021. ISSN 1939-3792. doi: 10.1002/aur.2563. URL https://www.ncbi.nlm.nih.gov/pmc/articles/PMC8592037/.

[27] M. C. Diemer, R. Ros-Demarize, C. C. Bradley, S. Kanne, S. H. Kim, J. Parish-Morris, L. G. Snyder, E. Wodka, S. Consortium, and L. A. Carpenter. Comparative Analysis of Autistic Women Across the Lifespan: Childhood vs. Adulthood Diagnosis. *Autism Research*, n/a(n/a), June 2025. ISSN 1939-3806. doi: 10.1002/aur.70073. URL https://onlinelibrary.wiley.com/doi/abs/10.1002/aur.70073. _eprint: https://onlinelibrary.wiley.com/doi/pdf/10.1002/aur.70073.

[28] Y. Dizitzer, G. Meiri, H. Flusser, A. Michaelovski, I. Dinstein, and I. Menashe. Comorbidity and health services' usage in children with autism spectrum disorder: a nested case–control study. *Epidemiology and Psychiatric Sciences*, 29:e95, Jan. 2020. ISSN 2045-7960, 2045-7979. doi: 10.1017/S2045796020000050. URL https://www.cambridge.org/core/journals/epidemiology-and-psychiatric-sciences/article/c

[29] M. G. Elrod, C. M. Nylund, A. L. Susi, G. H. Gorman, E. Hisle-Gorman, D. J. Rogers, and C. Erdie-Lalena. Prevalence of Diagnosed Sleep Disorders and Related Diagnostic and Surgical Procedures in Children with Autism Spectrum Disorders. *Journal of Developmental & Behavioral Pediatrics*, 37(5):377–384, June 2016. ISSN 0196-206X. doi: 10.1097/DBP.0000000000000248. URL https://journals.lww.com/00004703-201606000-00004.

[30] A. Estes, J. Munson, T. St. John, R. Finlayson, J. Pandey, B. Gottlieb, J. Herrington, and R. T. Schultz. Sleep problems in autism: Sex differences in the school-age population. *Autism research : official journal of the International Society for Autism Research*, 16(1): 164–173, Jan. 2023. ISSN 1939-3792. doi: 10.1002/aur.2848. URL https://www.ncbi.nlm.nih.gov/pmc/articles/PMC9839593/.

[31] J. M. Garcia, K. A. Anderson, J. R. Moore, T. Renn, and J. E. Rast. Prevalence of Substance Use Disorder Among Autistic Youth With and Without Co-Occurring Mental Health Conditions and a History of Trauma. *Autism Research*, 18(8):1704–1713, 2025. ISSN 1939-3806. doi: 10.1002/aur.70081. URL https://onlinelibrary.wiley.com/doi/abs/10.1002/aur.70081. _eprint: https://onlinelibrary.wiley.com/doi/pdf/10.1002/aur.70081.

[32] J. L. Greenlee, A. S. Mosley, A. M. Shui, J. Veenstra-VanderWeele, and K. O. Gotham. Medical and Behavioral Correlates of Depression History in Children and Adolescents With Autism Spectrum Disorder. *Pediatrics*, 137(Supplement_2):S105–S114, Feb. 2016. ISSN 0031-4005, 1098-4275. doi: 10.1542/peds.2015-2851I.

[33] M. Gupta, N. Gupta, Y. Fradkin, and T. Petti. Sleep Disturbances in Children and Adolescents with AutismSpectrum Disorder: An Overview for Clinicians. *Adolescent Psychiatry*, 13(1):1–24, Apr. 2023. ISSN 22106766. doi: 10.2174/2210676613666230126115646. URL https://www.eurekaselect.com/213178/article.

[34] B. N. Hand, A. M. Angell, L. Harris, and L. A. Carpenter. Prevalence of physical and mental health conditions in Medicare-enrolled, autistic older adults. *Autism*, 24(3):755–764, Apr. 2020. ISSN 1362-3613. doi: 10.1177/1362361319890793. URL https://www.ncbi.nlm.nih.gov/pmc/articles/PMC7433648/.

[35] B. N. Hand, T. W. Benevides, and H. J. Carretta. Suicidal Ideation and Self-inflicted Injury in Medicare Enrolled Autistic Adults With and Without Co-occurring Intellectual Disability. *Journal of Autism and Developmental Disorders*, 50(10):3489–3495, Oct. 2020. ISSN 1573-3432. doi: 10.1007/s10803-019-04345-x. URL https://doi.org/10.1007/s10803-019-04345-x.

[36] N. Haruvi-Lamdan, D. Horesh, S. Zohar, M. Kraus, and O. Golan. Autism Spectrum Disorder and Post-Traumatic Stress Disorder: An unexplored co-occurrence of conditions. *Autism*, 24(4):884–898, May 2020. ISSN 1362-3613, 1461-7005. doi: 10.1177/1362361320912143. URL `https://journals.sagepub.com/doi/10.1177/1362361320912143`.

[37] T. Hirvikoski, E. Mittendorfer-Rutz, M. Boman, H. Larsson, P. Lichtenstein, and S. Bölte. Premature mortality in autism spectrum disorder. *The British Journal of Psychiatry*, 208(3):232–238, Mar. 2016. ISSN 0007-1250, 1472-1465. doi: 10.1192/bjp.bp.114.160192. URL `https://www.cambridge.org/core/journals/the-british-journal-of-psychiatry/article/prema`

[38] T. Hirvikoski, M. Boman, Q. Chen, B. M. D'Onofrio, E. Mittendorfer-Rutz, P. Lichtenstein, S. Bölte, and H. Larsson. Individual risk and familial liability for suicide attempt and suicide in autism: a population-based study. *Psychological Medicine*, 50(9):1463–1474, July 2020. ISSN 0033-2917, 1469-8978. doi: 10.1017/S0033291719001405.

[39] J. D. Hoch and A. M. Youssef. Predictors of Trauma Exposure and Trauma Diagnoses for Children with Autism and Developmental Disorders Served in a Community Mental Health Clinic. *Journal of Autism and Developmental Disorders*, 50(2):634–649, 2020. ISSN 0162-3257. doi: 10.1007/s10803-019-04331-3. URL `https://pmc.ncbi.nlm.nih.gov/articles/PMC6994449/`.

[40] R. Holden, J. Mueller, J. McGowan, J. Sanyal, M. Kikoler, E. Simonoff, S. Velupillai, and J. Downs. Investigating Bullying as a Predictor of Suicidality in a Clinical Sample of Adolescents with Autism Spectrum Disorder. *Autism Research*, 13(6):988–997, June 2020. ISSN 1939-3792. doi: 10.1002/aur.2292. URL `https://pmc.ncbi.nlm.nih.gov/articles/PMC8647922/`.

[41] M. J. Hollocks, J. W. Lerh, I. Magiati, R. Meiser-Stedman, and T. S. Brugha. Anxiety and depression in adults with autism spectrum disorder: a systematic review and meta-analysis. *Psychological Medicine*, 49(4):559–572, Mar. 2019. ISSN 0033-2917, 1469-8978. doi: 10.1017/S0033291718002283. URL `https://www.cambridge.org/core/journals/psychological-medicine/article/anxiety-and-depr`

[42] M. Hosozawa, A. Sacker, and N. Cable. Timing of diagnosis, depression and self-harm in adolescents with autism spectrum disorder. *Autism*, 25 (1):70–78, Jan. 2021. ISSN 1362-3613. doi: 10.1177/1362361320945540. URL `https://doi.org/10.1177/1362361320945540`.

[43] M. M. Hossain, N. Khan, A. Sultana, P. Ma, E. L. J. McKyer, H. U. Ahmed, and N. Purohit. Prevalence of comorbid psychiatric disorders among people with autism spectrum disorder: An umbrella review of systematic reviews and meta-analyses. *Psychiatry Research*, 287:112922,

May 2020. ISSN 0165-1781. doi: 10.1016/j.psychres.2020.112922. URL https://www.sciencedirect.com/science/article/pii/S0165178120300093.

[44] R. Houghton, R. C. Ong, and F. Bolognani. Psychiatric comorbidities and use of psychotropic medications in people with autism spectrum disorder in the United States. *Autism Research*, 10(12): 2037–2047, 2017. ISSN 1939-3806. doi: 10.1002/aur.1848. URL https://onlinelibrary.wiley.com/doi/abs/10.1002/aur.1848. _eprint: https://onlinelibrary.wiley.com/doi/pdf/10.1002/aur.1848.

[45] J.-W. Hsu, L.-C. Chen, K.-L. Huang, S.-J. Tsai, Y.-M. Bai, T.-P. Su, T.-J. Chen, and M.-H. Chen. The influence of sex on major psychiatric comorbidities and parental psychiatric disorders in 22,698 children and adolescents with ICD-9-CM-based autism spectrum disorder. *European Archives of Psychiatry and Clinical Neuroscience*, 274(1):19–25, Feb. 2024. ISSN 1433-8491. doi: 10.1007/s00406-022-01529-y. URL https://doi.org/10.1007/s00406-022-01529-y.

[46] J.-S. Huang, F.-C. Yang, W.-C. Chien, T.-C. Yeh, C.-H. Chung, C.-K. Tsai, S.-J. Tsai, S.-S. Yang, N.-S. Tzeng, M.-H. Chen, and C.-S. Liang. Risk of Substance Use Disorder and Its Associations With Comorbidities and Psychotropic Agents in Patients With Autism. *JAMA Pediatrics*, 175(2):e205371, Feb. 2021. ISSN 2168-6203. doi: 10.1001/jamapediatrics.2020.5371. URL https://pmc.ncbi.nlm.nih.gov/articles/PMC7783585/.

[47] C. C. Hudson, L. Hall, and K. L. Harkness. Prevalence of Depressive Disorders in Individuals with Autism Spectrum Disorder: a Meta-Analysis. *Journal of Abnormal Child Psychology*, 47(1):165–175, Jan. 2019. ISSN 1573-2835. doi: 10.1007/s10802-018-0402-1. URL https://doi.org/10.1007/s10802-018-0402-1.

[48] M. M. Hughes, K. A. Shaw, M. E. Patrick, M. DiRienzo, A. V. Bakian, D. A. Bilder, M. S. Durkin, A. Hudson, M. H. Spivey, L. S. DaWalt, A. Salinas, Y. D. Schwenk, M. Lopez, T. M. Baroud, and M. J. Maenner. Adolescents With Autism Spectrum Disorder: Diagnostic Patterns, Co-occurring Conditions, and Transition Planning. *Journal of Adolescent Health*, 73(2):271–278, Aug. 2023. ISSN 1054-139X. doi: 10.1016/j.jadohealth.2022.12.010. URL https://www.sciencedirect.com/science/article/pii/S1054139X23000010.

[49] L. Hull, I. Stark, M. Lundberg, V. H. Ahlqvist, S. I. Nordström, A. Ohlis, G. Hadlaczky, D. Rai, and C. Magnusson. Sex differences in self-harm and suicide in young autistic adults. *Acta Psychiatrica Scandinavica*, 150(4):223–233, 2024. ISSN 1600-0447. doi: 10.1111/acps.13736. URL https://onlinelibrary.wiley.com/doi/abs/10.1111/acps.13736. _eprint: https://onlinelibrary.wiley.com/doi/pdf/10.1111/acps.13736.

[50] M. C. Hunsche, S. Saqui, P. Mirenda, A. Zaidman-Zait, T. Bennett, E. Duku, M. Elsabbagh, S. Georgiades, I. M. Smith, P. Szatmari, W. J. Ungar, T. Vaillancourt, C. Waddell, L. Zwaigenbaum, and C. M. Kerns. Parent-Reported Rates and Clinical Correlates of Suicidality in Children with Autism Spectrum Disorder: A Longitudinal Study. *Journal of Autism and Developmental Disorders*, 50(10):3496–3509, Oct. 2020. ISSN 1573-3432. doi: 10.1007/s10803-020-04373-y. URL https://doi.org/10.1007/s10803-020-04373-y.

[51] I. Ivanović. Psychiatric Comorbidities in Children With ASD: Autism Centre Experience. *Frontiers in Psychiatry*, 12, June 2021. ISSN 1664-0640. doi: 10.3389/fpsyt.2021.673169. URL https://www.frontiersin.org/journals/psychiatry/articles/10.3389/fpsyt.2021.673169/full

[52] N. Jadav and V. H. Bal. Associations between co-occurring conditions and age of autism diagnosis: Implications for mental health training and adult autism research. *Autism Research*, 15(11): 2112–2125, 2022. ISSN 1939-3806. doi: 10.1002/aur.2808. URL https://onlinelibrary.wiley.com/doi/abs/10.1002/aur.2808. _eprint: https://onlinelibrary.wiley.com/doi/pdf/10.1002/aur.2808.

[53] E. Jokiranta-Olkoniemi, D. Gyllenberg, D. Sucksdorff, A. Suominen, K. Kronström, R. Chudal, and A. Sourander. Risk for Premature Mortality and Intentional Self-harm in Autism Spectrum Disorders. *Journal of Autism and Developmental Disorders*, 51(9):3098–3108, Sept. 2021. ISSN 1573-3432. doi: 10.1007/s10803-020-04768-x. URL https://doi.org/10.1007/s10803-020-04768-x.

[54] A. Junewicz, S. Dhir, F. Guo, Y. Song, C. R. Stein, and A. Baroni. Trauma diagnoses during emergency psychiatric evaluation among youth with and without autism spectrum disorder. *Autism*, 28(12):3201–3207, Dec. 2024. ISSN 1362-3613, 1461-7005. doi: 10.1177/13623613241274832. URL https://journals.sagepub.com/doi/10.1177/13623613241274832.

[55] H. C. Kaltenegger, S. Doering, C. Gillberg, P. Wennberg, and S. Lundström. Low prevalence of risk drinking in adolescents and young adults with autism spectrum problems. *Addictive Behaviors*, 113:106671, Feb. 2021. ISSN 0306-4603. doi: 10.1016/j.addbeh.2020.106671. URL https://www.sciencedirect.com/science/article/pii/S0306460320308017.

[56] C. H. Kennedy, T. B. Huedo-Medina, J. Twachtman-Bassett, L. Kalsner, R. Areny-Joval, and I.-M. Eigsti. Health Condition Co-Morbidities in Children with Autism and Their Association with Challenging Behavior. *Advances in Neurodevelopmental Disorders*, pages 1–7, 2024. URL https://link.springer.com/article/10.1007/s41252-024-00395-0.

[57] R. Kervin, Chelsea Berger, Sun Jae Moon, Harrison Hill, Dongchan Park, and Jung Won Kim. Behavioral addiction and autism spectrum disorder: A systematic review. *Research in Developmental Disabilities*, 117:

104033, Oct. 2021. ISSN 0891-4222. doi: 10.1016/j.ridd.2021.104033. URL https://www.sciencedirect.com/science/article/pii/S0891422221001827.

[58] V. Khachadourian, B. Mahjani, S. Sandin, A. Kolevzon, J. D. Buxbaum, A. Reichenberg, and M. Janecka. Comorbidities in autism spectrum disorder and their etiologies. *Translational Psychiatry*, 13(1):71, Feb. 2023. ISSN 2158-3188. doi: 10.1038/s41398-023-02374-w. URL https://www.nature.com/articles/s41398-023-02374-w.

[59] A. V. Kirby, A. V. Bakian, Y. Zhang, D. A. Bilder, B. R. Keeshin, and H. Coon. A 20-year study of suicide death in a statewide autism population. *Autism Research*, 12(4):658–666, Apr. 2019. ISSN 1939-3792. doi: 10.1002/aur.2076. URL https://pmc.ncbi.nlm.nih.gov/articles/PMC6457664/.

[60] A. C. Kirsch, A. R. S. Huebner, S. Q. Mehta, F. R. Howie, A. L. Weaver, S. M. Myers, R. G. Voigt, and S. K. Katusic. Association of Comorbid Mood and Anxiety Disorders With Autism Spectrum Disorder. *JAMA Pediatrics*, 174(1):63–70, Jan. 2020. ISSN 2168-6203. doi: 10.1001/jamapediatrics.2019.4368. URL https://doi.org/10.1001/jamapediatrics.2019.4368.

[61] A. Koceski and V. Trajkovski. Health status of people with autism spectrum disorder. *Advances in Autism*, 8(3):252–263, June 2022. ISSN 2056-3868, 2056-3868. doi: 10.1108/AIA-01-2021-0005. URL http://www.emerald.com/aia/article/8/3/252-263/59892.

[62] S. V. Koch, J. T. Larsen, S. E. Mouridsen, M. Bentz, L. Petersen, C. Bulik, P. B. Mortensen, and K. J. Plessen. Autism spectrum disorder in individuals with anorexia nervosa and in their first- and second-degree relatives: Danish nationwide register-based cohort-study. *The British Journal of Psychiatry*, 206(5):401–407, May 2015. ISSN 0007-1250, 1472-1465. doi: 10.1192/bjp.bp.114.153221. URL https://www.cambridge.org/core/journals/the-british-journal-of-psychiatry/article/autis

[63] K. Kõlves, C. Fitzgerald, M. Nordentoft, S. J. Wood, and A. Erlangsen. Assessment of Suicidal Behaviors Among Individuals With Autism Spectrum Disorder in Denmark. *JAMA Network Open*, 4(1):e2033565, Jan. 2021. ISSN 2574-3805. doi: 10.1001/jamanetworkopen.2020.33565. URL https://pmc.ncbi.nlm.nih.gov/articles/PMC12578491/.

[64] A. Lacroix, C.-C. Tzang, J. X. Yu, B. K. Jacob, M. Alexandrovsky, A. Winge-Breen, T. Rodak, and M.-C. Lai. Disproportionate mental health risks in autistic females: A rapid review with quantitative and narrative syntheses. *Frontiers in Neuroendocrinology*, page 101229, Dec. 2025. ISSN 0091-3022. doi: 10.1016/j.yfrne.2025.101229. URL https://www.sciencedirect.com/science/article/pii/S009130222500055X.

[65] M.-C. Lai, C. Kassee, R. Besney, S. Bonato, W. Mandy, P. Szatmari, and S. Ameis. Prevalence of co-occurring mental health diagnoses in the autism population: a systematic review and meta-analysis. *The Lancet Psychiatry*, 6(10):819–829, Oct. 2019. ISSN 2215-0366. doi: 10.1016/S2215-0366(19)30289-5. URL `https://www.sciencedirect.com/science/article/abs/pii/S2215036619302895`.

[66] M.-C. Lai, N. R. Saunders, A. Huang, A. Artani, A. S. Wilton, J. Zaheer, S. H. Ameis, H. K. Brown, and Y. Lunsky. Self-Harm Events and Suicide Deaths Among Autistic Individuals in Ontario, Canada. *JAMA Network Open*, 6(8):e2327415, Aug. 2023. ISSN 2574-3805. doi: 10.1001/jamanetworkopen.2023.27415. URL `https://doi.org/10.1001/jamanetworkopen.2023.27415`.

[67] C. Laverty, C. Oliver, J. Moss, L. Nelson, and C. Richards. Persistence and predictors of self-injurious behaviour in autism: a ten-year prospective cohort study. *Molecular Autism*, 11(1):8, Jan. 2020. ISSN 2040-2392. doi: 10.1186/s13229-019-0307-z. URL `https://doi.org/10.1186/s13229-019-0307-z`.

[68] A. G. Lever and H. M. Geurts. Psychiatric Co-occurring Symptoms and Disorders in Young, Middle-Aged, and Older Adults with Autism Spectrum Disorder. *Journal of Autism and Developmental Disorders*, 46(6): 1916–1930, June 2016. ISSN 1573-3432. doi: 10.1007/s10803-016-2722-8. URL `https://doi.org/10.1007/s10803-016-2722-8`.

[69] L. Licence, C. Oliver, J. Moss, and C. Richards. Prevalence and Risk-Markers of Self-Harm in Autistic Children and Adults. *Journal of Autism and Developmental Disorders*, 50(10):3561–3574, Oct. 2020. ISSN 1573-3432. doi: 10.1007/s10803-019-04260-1. URL `https://doi.org/10.1007/s10803-019-04260-1`.

[70] S. Liu, H. Larsson, R. Kuja-Halkola, P. Lichtenstein, A. Butwicka, and M. J. Taylor. Age-related physical health of older autistic adults in Sweden: a longitudinal, retrospective, population-based cohort study. *The Lancet Healthy Longevity*, 4(7):e307–e315, July 2023. ISSN 2666-7568. doi: 10.1016/S2666-7568(23)00067-3. URL `https://www.thelancet.com/journals/lanhl/article/PIIS2666-7568(23)00067-3/fulltext`.

[71] A. Longo, D. Radford, and B. N. Hand. A US national update of health condition prevalence among privately-insured autistic adults. *Journal of Comparative Effectiveness Research*, 13 (3):e230051, Mar. 2024. doi: 10.57264/cer-2023-0051. URL `https://becarispublishing.com/doi/full/10.57264/cer-2023-0051`.

[72] C. Lopez-Arvizu, D. L. Steelesmith, B. N. Hand, R. Huang, A. J. Thompson, E. N. Llamocca, B. A. Quinn, C. A. Fontanella, and J. V. Campo. Correlates of Deliberate Self-Harm in Youth With

Autism and/or Intellectual Disability. *JAACAP Open*, 3(3):477–484, Sept. 2025. ISSN 2949-7329. doi: 10.1016/j.jaacop.2024.09.012. URL https://www.sciencedirect.com/science/article/pii/S2949732925000043.

[73] V. Lushin, S. Marcus, S. Tao, M. Engstrom, A. Roux, and L. Shea. Comparing the prevalence of substance use disorders between persons with and without autism spectrum disorders. *Autism*, 29(7):1674–1687, July 2025. ISSN 1362-3613. doi: 10.1177/13623613251325282. URL https://doi.org/10.1177/13623613251325282.

[74] P. Malhi and N. Sankhyan. Intentional Self Harm in Children with Autism. *The Indian Journal of Pediatrics*, 88(2):158–160, Feb. 2021. ISSN 0973-7693. doi: 10.1007/s12098-020-03276-1. URL https://doi.org/10.1007/s12098-020-03276-1.

[75] B. A. Malow, T. Katz, A. M. Reynolds, A. Shui, M. Carno, H. V. Connolly, D. Coury, and A. E. Bennett. Sleep Difficulties and Medications in Children With Autism Spectrum Disorders: A Registry Study. *Pediatrics*, 137(Supplement_2):S98–S104, Feb. 2016. ISSN 0031-4005. doi: 10.1542/peds.2015-2851H. URL https://doi.org/10.1542/peds.2015-2851H.

[76] H. Mansour, S. Kurana, A. Eshetu, S. Hoare, C. El Baou, I. Arnold, C. Halys, G. R. Stewart, R. Desai, A. John, W. Mandy, E. O'Nions, and J. Stott. Prevalence of Post-Traumatic Stress Disorder (PTSD) in autistic children or young people (CYP) and adults: A systematic review and meta-analysis. *Clinical Psychology Review*, 120:102621, Aug. 2025. ISSN 0272-7358. doi: 10.1016/j.cpr.2025.102621. URL https://www.sciencedirect.com/science/article/pii/S0272735825000881.

[77] L. Margari, R. Palumbi, A. Peschechera, F. Craig, C. de Giambattista, P. Ventura, and F. Margari. Sex-Gender Comparisons in Comorbidities of Children and Adolescents With High-Functioning Autism Spectrum Disorder. *Frontiers in Psychiatry*, 10:159, Mar. 2019. ISSN 1664-0640. doi: 10.3389/fpsyt.2019.00159. URL https://www.ncbi.nlm.nih.gov/pmc/articles/PMC6445051/.

[78] A. F. Martin, A. Jassi, A. E. Cullen, M. Broadbent, J. Downs, and G. Krebs. Co-occurring obsessive–compulsive disorder and autism spectrum disorder in young people: prevalence, clinical characteristics and outcomes. *European Child & Adolescent Psychiatry*, 29(11):1603–1611, Nov. 2020. ISSN 1018-8827. doi: 10.1007/s00787-020-01478-8. URL https://research.ebsco.com/linkprocessor/plink?id=7e1803ff-7936-374b-bfef-308be7614b35.

[79] M. I. Martini, R. Kuja-Halkola, A. Butwicka, E. Du Rietz, B. M. D'Onofrio, F. Happé, A. Kanina, H. Larsson, S. Lundström, J. Martin, M. A. Rosenqvist, P. Lichtenstein, and M. J. Taylor. Sex Differences in Mental Health Problems and Psychiatric Hospitalization

in Autistic Young Adults. *JAMA Psychiatry*, 79(12):1188–1198, Dec. 2022. ISSN 2168-622X. doi: 10.1001/jamapsychiatry.2022.3475. URL https://doi.org/10.1001/jamapsychiatry.2022.3475.

[80] A. Masi, M. A. Moni, S. I. Azim, B. Choi, H. Heussler, P.-I. Lin, A. M. Diaz, and V. Eapen. Clinical and behavioral attributes leading to sleep disorders in children on the autism spectrum. *Autism Research*, 15 (7):1274–1287, 2022. ISSN 1939-3806. doi: 10.1002/aur.2745. URL https://onlinelibrary.wiley.com/doi/abs/10.1002/aur.2745. _eprint: https://onlinelibrary.wiley.com/doi/pdf/10.1002/aur.2745.

[81] T. May, P. D. Pilkington, R. Younan, and K. Williams. Overlap of autism spectrum disorder and borderline personality disorder: A systematic review and meta-analysis. *Autism Research*, 14(12): 2688–2710, 2021. ISSN 1939-3806. doi: 10.1002/aur.2619. URL https://onlinelibrary.wiley.com/doi/abs/10.1002/aur.2619. _eprint: https://onlinelibrary.wiley.com/doi/pdf/10.1002/aur.2619.

[82] M. Micai, L. M. Fatta, L. Gila, A. Caruso, T. Salvitti, F. Fulceri, A. Ciaramella, R. D'Amico, C. Del Giovane, M. Bertelli, G. Romano, H. J. Schünemann, and M. L. Scattoni. Prevalence of co-occurring conditions in children and adults with autism spectrum disorder: A systematic review and meta-analysis. *Neuroscience & Biobehavioral Reviews*, 155:105436, Dec. 2023. ISSN 0149-7634. doi: 10.1016/j.neubiorev.2023.105436. URL https://www.sciencedirect.com/science/article/pii/S0149763423004050.

[83] H. Minnis, C.-M. Messow, A. McConnachie, P. Bradshaw, A. Briggs, P. Wilson, and C. Gillberg. Autism and attachment disorder symptoms in the general population: Prevalence, overlap, and burden. *Developmental Child Welfare*, 2(1):37–51, Mar. 2020. ISSN 2516-1032. doi: 10.1177/2516103220902778. URL https://doi.org/10.1177/2516103220902778.

[84] M. G. Mosner, J. L. Kinard, J. S. Shah, S. McWeeny, R. K. Greene, S. C. Lowery, C. A. Mazefsky, and G. S. Dichter. Rates of Co-occurring Psychiatric Disorders in Autism Spectrum Disorder using the Mini International Neuropsychiatric Interview. *Journal of autism and developmental disorders*, 49(9):3819–3832, Sept. 2019. ISSN 0162-3257. doi: 10.1007/s10803-019-04090-1. URL https://www.ncbi.nlm.nih.gov/pmc/articles/PMC6669096/.

[85] T. Mutluer, H. Aslan Genç, A. Özcan Morey, H. Yapici Eser, B. Ertinmaz, M. Can, and K. Munir. Population-Based Psychiatric Comorbidity in Children and Adolescents With Autism Spectrum Disorder: A Meta-Analysis. *Frontiers in Psychiatry*, 13, May 2022. ISSN 1664-0640. doi: 10.3389/fpsyt.2022.856208. URL https://www.frontiersin.org/journals/psychiatry/articles/10.3389/fpsyt.2022.856208/full

[86] V. Newell, L. Phillips, C. Jones, E. Townsend, C. Richards, and S. Cassidy. A systematic review and meta-analysis of suicidality in autistic and possibly autistic people without co-occurring intellectual disability. *Molecular Autism*, 14(1):12, Mar. 2023. ISSN 2040-2392. doi: 10.1186/s13229-023-00544-7. URL `https://doi.org/10.1186/s13229-023-00544-7`.

[87] B. Oakley, E. Loth, and D. G. Murphy. Autism and mood disorders. *International Review of Psychiatry*, 33(3):280–299, Apr. 2021. ISSN 0954-0261. doi: 10.1080/09540261.2021.1872506. URL `https://doi.org/10.1080/09540261.2021.1872506`. _eprint: https://doi.org/10.1080/09540261.2021.1872506.

[88] E. O'Nions, J. Brown, J. E. J. Buckman, R. Charlton, C. Cooper, C. E. Baou, F. Happé, S. Hoare, D. Lewer, J. Manthorpe, D. G. J. McKechnie, M. Richards, R. Saunders, W. Mandy, and J. Stott. Diagnosis of common health conditions among autistic adults in the UK: evidence from a matched cohort study. *The Lancet Regional Health – Europe*, 41, June 2024. ISSN 2666-7762. doi: 10.1016/j.lanepe.2024.100907. URL `https://www.thelancet.com/journals/lanepe/article/PIIS2666-7762(24)00073-5/fulltext`.

[89] E. O'Nions, D. Lewer, I. Petersen, J. Brown, J. E. J. Buckman, R. Charlton, C. Cooper, C. E. Baou, F. Happé, J. Manthorpe, D. G. J. McKechnie, M. Richards, R. Saunders, C. Zanker, W. Mandy, and J. Stott. Estimating life expectancy and years of life lost for autistic people in the UK: a matched cohort study. *The Lancet Regional Health – Europe*, 36, Jan. 2024. ISSN 2666-7762. doi: 10.1016/j.lanepe.2023.100776. URL `https://www.thelancet.com/journals/lanepe/article/PIIS2666-7762(23)00195-3/fulltext`.

[90] M. K. Pelton, H. Crawford, A. E. Robertson, J. Rodgers, S. Baron-Cohen, and S. Cassidy. Understanding Suicide Risk in Autistic Adults: Comparing the Interpersonal Theory of Suicide in Autistic and Non-autistic Samples. *Journal of Autism and Developmental Disorders*, 50(10):3620–3637, Oct. 2020. ISSN 1573-3432. doi: 10.1007/s10803-020-04393-8. URL `https://doi.org/10.1007/s10803-020-04393-8`.

[91] C. Rattaz, C. Michelon, and A. Baghdadli. Symptom severity as a risk factor for self-injurious behaviours in adolescents with autism spectrum disorders. *Journal of Intellectual Disability Research*, 59 (8):730–741, 2015. ISSN 1365-2788. doi: 10.1111/jir.12177. URL `https://onlinelibrary.wiley.com/doi/abs/10.1111/jir.12177`. _eprint: https://onlinelibrary.wiley.com/doi/pdf/10.1111/jir.12177.

[92] K. N. Reikersdorfer, A. Singh, J. D. Young, M. B. Batty, A. E. Steele, L. C. Yuen, D. A. Momtaz, J. N. Weissert, D. S. Liu, and G. D. Hogue. The Troubling Rise of Scurvy: A Review and National Analysis of Incidence, Associated Risk Factors, and Clinical Manifestations. *JAAOS Global Research & Reviews*, 8(7):e24.00162, July 2024. doi: 10.5435/JAAOSGlobal-D-24-00162.

[93] M. Ressel, B. Thompson, M.-H. Poulin, C. L. Normand, M. H. Fisher, G. Couture, and G. Iarocci. Systematic review of risk and protective factors associated with substance use and abuse in individuals with autism spectrum disorders. *Autism*, 24(4):899–918, May 2020. ISSN 1362-3613, 1461-7005. doi: 10.1177/1362361320910963. URL https://journals.sagepub.com/doi/10.1177/1362361320910963.

[94] C. Richards, L. Davies, and C. Oliver. Predictors of Self-Injurious Behavior and Self-Restraint in Autism Spectrum Disorder: Towards a Hypothesis of Impaired Behavioral Control. *Journal of Autism and Developmental Disorders*, 47(3):701–713, Mar. 2017. ISSN 1573-3432. doi: 10.1007/s10803-016-3000-5. URL https://doi.org/10.1007/s10803-016-3000-5.

[95] S. J. Rim, K. Kwak, and S. Park. Risk of psychiatric comorbidity with autism spectrum disorder and its association with diagnosis timing using a nationally representative cohort. *Research in Autism Spectrum Disorders*, 104:102134, June 2023. ISSN 17509467. doi: 10.1016/j.rasd.2023.102134. URL https://linkinghub.elsevier.com/retrieve/pii/S175094672300034X.

[96] A. M. Roux, S. Tao, S. Marcus, V. Lushin, and L. L. Shea. A national profile of substance use disorder among Medicaid enrollees on the autism spectrum or with intellectual disability. *Disability and health journal*, 15(2 Suppl):101289, June 2022. ISSN 1936-6574. doi: 10.1016/j.dhjo.2022.101289. URL https://pmc.ncbi.nlm.nih.gov/articles/PMC9232987/.

[97] F. Rumball. A Systematic Review of the Assessment and Treatment of Posttraumatic Stress Disorder in Individuals with Autism Spectrum Disorders. *Review Journal of Autism and Developmental Disorders*, 6(3): 294–324, Sept. 2019. ISSN 2195-7185. doi: 10.1007/s40489-018-0133-9. URL https://doi.org/10.1007/s40489-018-0133-9.

[98] F. Rumball, F. Happé, and N. Grey. Experience of Trauma and PTSD Symptoms in Autistic Adults: Risk of PTSD Development Following DSM-5 and Non-DSM-5 Traumatic Life Events. *Autism Research*, 13 (12):2122–2132, Dec. 2020. ISSN 1939-3806. doi: 10.1002/aur.2306. URL https://onlinelibrary.wiley.com/doi/10.1002/aur.2306.

[99] F. Rumball, L. Brook, F. Happé, and A. Karl. Heightened risk of posttraumatic stress disorder in adults with autism spectrum disorder: The role of cumulative trauma and memory deficits. *Research in Developmental Disabilities*, 110:103848, Mar. 2021. ISSN 0891-4222. doi: 10.1016/j.ridd.2020.103848. URL https://www.sciencedirect.com/science/article/pii/S0891422220302808.

[100] E. Rødgaard, K. Jensen, K. W. Miskowiak, and L. Mottron. Autism comorbidities show elevated female-to-male odds ratios and are associated

with the age of first autism diagnosis. *Acta Psychiatrica Scandinavica*, 144 (5):475–486, Nov. 2021. ISSN 0001690X. doi: 10.1111/acps.13345.

[101] M. Sader, A. Weston, K. Buchan, J. Kerr-Gaffney, K. Gillespie-Smith, H. Sharpe, and F. Duffy. The Co-Occurrence of Autism and Avoidant/Restrictive Food Intake Disorder (ARFID): A Prevalence-Based Meta-Analysis. *International Journal of Eating Disorders*, 58 (3):473–488, 2025. ISSN 1098-108X. doi: 10.1002/eat.24369. URL https://onlinelibrary.wiley.com/doi/abs/10.1002/eat.24369. _eprint: https://onlinelibrary.wiley.com/doi/pdf/10.1002/eat.24369.

[102] M. Salehi, S. Jaka, A. Lotfi, A. Ahmad, M. Saeidi, and S. Gunturu. Prevalence, Socio-Demographic Characteristics, and Co-Morbidities of Autism Spectrum Disorder in US Children: Insights from the 2020–2021 National Survey of Children's Health. *Children*, 12(3):297, Feb. 2025. ISSN 2227-9067. doi: 10.3390/children12030297. URL https://www.ncbi.nlm.nih.gov/pmc/articles/PMC11941283/.

[103] S. Scarpelli, D. Menghini, V. Alfonsi, F. Giumello, L. Annarumma, M. Gorgoni, G. Valeri, M. Pazzaglia, L. De Gennaro, and S. Vicari. Sleep Disturbances and Co-sleeping in Italian Children and Adolescents with Autism Spectrum Disorder. *Journal of Autism and Developmental Disorders*, Aug. 2024. ISSN 1573-3432. doi: 10.1007/s10803-024-06507-y. URL https://doi.org/10.1007/s10803-024-06507-y.

[104] D. E. Schendel, M. Overgaard, J. Christensen, L. Hjort, M. Jørgensen, M. Vestergaard, and E. T. Parner. Association of Psychiatric and Neurologic Comorbidity With Mortality Among Persons With Autism Spectrum Disorder in a Danish Population. *JAMA Pediatrics*, 170(3):243–250, Mar. 2016. ISSN 2168-6203. doi: 10.1001/jamapediatrics.2015.3935. URL https://doi.org/10.1001/jamapediatrics.2015.3935.

[105] W. Schott, S. Tao, and L. Shea. Co-occurring conditions and racial-ethnic disparities: Medicaid enrolled adults on the autism spectrum. *Autism Research*, 15(1):70–85, 2022. ISSN 1939-3806. doi: 10.1002/aur.2644. URL https://onlinelibrary.wiley.com/doi/abs/10.1002/aur.2644. _eprint: https://onlinelibrary.wiley.com/doi/pdf/10.1002/aur.2644.

[106] V. Semovski, C. King, N. Lapshina, and S. L. Stewart. A cross-sectional examination of service complexity in youths with co-occurring autism spectrum disorder and psychiatric or medical diagnoses across service sectors. *Frontiers in Psychology*, 13, Feb. 2023. ISSN 1664-1078. doi: 10.3389/fpsyg.2022.1027373. URL https://www.frontiersin.org/journals/psychology/articles/10.3389/fpsyg.2022.1027373/ful

[107] A. Shoaib, M. S. Cepeda, G. Murray, and R. Ochs-Ross. Autism: Comorbidities and Treatment Patterns in the Real World, a Retrospective Cohort Study Among Children, Adolescents and Adults Newly Diagnosed with

Autism. *Journal of Autism and Developmental Disorders*, 52(10):4311–4320, Oct. 2022. ISSN 1573-3432. doi: 10.1007/s10803-021-05289-x. URL https://link-springer-com.mutex.gmu.edu/article/10.1007/s10803-021-05289-x. Company: Springer Distributor: Springer Institution: Springer Label: Springer Number: 10.

[108] G. N. Soke, M. J. Maenner, D. Christensen, M. Kurzius-Spencer, and L. A. Schieve. Prevalence of Co-occurring Medical and Behavioral Conditions/Symptoms Among 4- and 8-Year-Old Children with Autism Spectrum Disorder in Selected Areas of the United States in 2010. *Journal of Autism and Developmental Disorders*, 48(8):2663–2676, Aug. 2018. ISSN 1573-3432. doi: 10.1007/s10803-018-3521-1. URL https://link.springer.com/article/10.1007/s10803-018-3521-1. Company: Springer Distributor: Springer Institution: Springer Label: Springer Number: 8.

[109] I. Stark, D. Rai, M. Lundberg, I. Culpin, S. I. Nordström, A. Ohlis, and C. Magnusson. Autism and self-harm: A population-based and discordant sibling study of young individuals. *Acta Psychiatrica Scandinavica*, 146 (5):468–477, Nov. 2022. ISSN 0001-690X. doi: 10.1111/acps.13479. URL https://onlinelibrary.wiley.com/doi/full/10.1111/acps.13479.

[110] C. Steenfeldt-Kristensen, C. A. Jones, and C. Richards. The Prevalence of Self-injurious Behaviour in Autism: A Meta-analytic Study. *Journal of Autism and Developmental Disorders*, 50(11):3857–3873, Nov. 2020. ISSN 1573-3432. doi: 10.1007/s10803-020-04443-1. URL https://doi.org/10.1007/s10803-020-04443-1.

[111] G. R. Stewart, A. Corbett, C. Ballard, B. Creese, D. Aarsland, A. Hampshire, R. A. Charlton, and F. Happé. Self-harm and Suicidality Experiences of Middle-Age and Older Adults With vs. Without High Autistic Traits. *Journal of Autism and Developmental Disorders*, 53(8):3034–3046, 2023. ISSN 0162-3257. doi: 10.1007/s10803-022-05595-y. URL https://pmc.ncbi.nlm.nih.gov/articles/PMC10313542/.

[112] S. Tewolde, S. B. Rosenberg, J. A. G. Estrada, M. P. Jimenez, A. Scott, A. Higgins, and E. Rubenstein. Epidemiology of Alzheimer Disease and Related Dementia Among Medicare and Medicaid Enrolled Autistic Adults, 2011–2019. *Autism Research*, 18(5):1077–1086, 2025. ISSN 1939-3806. doi: 10.1002/aur.70035. URL https://onlinelibrary.wiley.com/doi/abs/10.1002/aur.70035. _eprint: https://onlinelibrary.wiley.com/doi/pdf/10.1002/aur.70035.

[113] A. Tint, H. K. Brown, S. Chen, M.-C. Lai, L. A. Tarasoff, S. N. Vigod, S. Parish, S. M. Havercamp, and Y. Lunsky. Health Characteristics of Reproductive-Aged Autistic Women in Ontario: A Population-Based, Cross-Sectional Study. *Autism : the international journal of research and practice*, 25(4):1114–1124, May

2021. ISSN 1362-3613. doi: 10.1177/1362361320982819. URL `https://www.ncbi.nlm.nih.gov/pmc/articles/PMC8089036/`.

[114] A. Tint, H. Chung, M.-C. Lai, R. Balogh, E. Lin, A. Durbin, and Y. Lunsky. Health conditions and service use of autistic women and men: A retrospective population-based case–control study. *Autism*, 27(6):1641–1657, Aug. 2023. ISSN 1362-3613. doi: 10.1177/13623613221144353. URL `https://doi.org/10.1177/13623613221144353`.

[115] A. K. Toman, H. R. AbdulRasool, F. Lami, S. M. Jasim, O. A. Jaber, N. D. Nayeri, M. S. Sabet, and G. Al-Gburi. Exploring co-occurring conditions in Iraqi children with autism spectrum disorder: prevalence, characteristics, and potential risk factors. *Frontiers in Psychiatry*, 16, July 2025. ISSN 1664-0640. doi: 10.3389/fpsyt.2025.1592374. URL `https://www.frontiersin.org/journals/psychiatry/articles/10.3389/fpsyt.2025.1592374/ful`

[116] A. Tomkies, R. F. Johnson, G. Shah, M. Caraballo, P. Evans, and R. B. Mitchell. Obstructive Sleep Apnea in Children With Autism. *Journal of Clinical Sleep Medicine : JCSM : Official Publication of the American Academy of Sleep Medicine*, 15(10):1469–1476, Oct. 2019. ISSN 1550-9389. doi: 10.5664/jcsm.7978. URL `https://www.ncbi.nlm.nih.gov/pmc/articles/PMC6778353/`.

[117] M. Uljarević, D. Hedley, K. Rose-Foley, I. Magiati, R. Y. Cai, C. Dissanayake, A. Richdale, and J. Trollor. Anxiety and Depression from Adolescence to Old Age in Autism Spectrum Disorder. *Journal of Autism and Developmental Disorders*, 50(9):3155–3165, Sept. 2020. ISSN 1573-3432. doi: 10.1007/s10803-019-04084-z. URL `https://doi.org/10.1007/s10803-019-04084-z`.

[118] J. F. G. Underwood, M. DelPozo-Banos, A. Frizzati, D. Rai, A. John, and J. Hall. Neurological and psychiatric disorders among autistic adults: a population healthcare record study. *Psychological Medicine*, 53(12):5663–5673, Oct. 2022. ISSN 0033-2917. doi: 10.1017/S0033291722002884. URL `https://www.ncbi.nlm.nih.gov/pmc/articles/PMC10482712/`.

[119] J. Vaquerizo-Serrano, G. Salazar de Pablo, J. Singh, and P. Santosh. Autism Spectrum Disorder and Clinical High Risk for Psychosis: A Systematic Review and Meta-analysis. *Journal of Autism and Developmental Disorders*, 52(4):1568–1586, Apr. 2022. ISSN 1573-3432. doi: 10.1007/s10803-021-05046-0. URL `https://doi.org/10.1007/s10803-021-05046-0`.

[120] K. J. Varcin, S. E. Herniman, A. Lin, Y. Chen, Y. Perry, C. Pugh, K. Chisholm, A. J. O. Whitehouse, and S. J. Wood. Occurrence of psychosis and bipolar disorder in adults with autism: A systematic review and meta-analysis. *Neuroscience & Biobehavioral Reviews*, 134:104543, Mar. 2022. ISSN 0149-7634. doi: 10.1016/j.neubiorev.2022.104543. URL `https://www.sciencedirect.com/science/article/pii/S014976342200029X`.

[121] G. Vivanti, S. Tao, K. Lyall, D. L. Robins, and L. L. Shea. The prevalence and incidence of early-onset dementia among adults with Autism Spectrum Disorder. *Autism research : official journal of the International Society for Autism Research*, 14(10):2189–2199, Oct. 2021. ISSN 1939-3792. doi: 10.1002/aur.2590. URL `https://www.ncbi.nlm.nih.gov/pmc/articles/PMC8487995/`.

[122] G. Vivanti, W.-L. Lee, J. Ventimiglia, S. Tao, K. Lyall, and L. L. Shea. Prevalence of Dementia Among US Adults With Autism Spectrum Disorder. *JAMA Network Open*, 8(1):e2453691–e2453691, Jan. 2025. doi: 10.1001/jamanetworkopen.2024.53691. URL `https://jamanetwork.com/journals/jamanetworkopen/fullarticle/2828643`.

[123] R. Vohra, S. Madhavan, and U. Sambamoorthi. Comorbidity prevalence, healthcare utilization, and expenditures of Medicaid enrolled adults with autism spectrum disorders. *Autism : the international journal of research and practice*, 21(8):995–1009, Nov. 2017. ISSN 1362-3613. doi: 10.1177/1362361316665222. URL `https://www.ncbi.nlm.nih.gov/pmc/articles/PMC5517354/`.

[124] E. Weir, C. Allison, and S. Baron-Cohen. Understanding the substance use of autistic adolescents and adults: a mixed-methods approach - PMC. *Lancet Psychiatry*, 8(8):673–685, Aug. 2021. doi: 10.1016/S2215-0366(21)00160-7.

[125] J. A. Weiss, B. Isaacs, H. Diepstra, A. S. Wilton, H. K. Brown, C. McGarry, and Y. Lunsky. Health Concerns and Health Service Utilization in a Population Cohort of Young Adults with Autism Spectrum Disorder. *Journal of Autism and Developmental Disorders*, 48(1):36–44, 2018. ISSN 0162-3257. doi: 10.1007/s10803-017-3292-0. URL `https://www.ncbi.nlm.nih.gov/pmc/articles/PMC5760600/`.

[126] H. Westwood, W. Mandy, and K. Tchanturia. Clinical evaluation of autistic symptoms in women with anorexia nervosa. *Molecular Autism*, 8, Mar. 2017. doi: 10.1186/s13229-017-0128-x.

[127] E. Widnall, S. Epstein, C. Polling, S. Velupillai, A. Jewell, R. Dutta, E. Simonoff, R. Stewart, R. Gilbert, T. Ford, M. Hotopf, R. D. Hayes, and J. Downs. Autism spectrum disorders as a risk factor for adolescent self-harm: a retrospective cohort study of 113,286 young people in the UK. *BMC Medicine*, 20(1):137, Apr. 2022. ISSN 1741-7015. doi: 10.1186/s12916-022-02329-w. URL `https://doi.org/10.1186/s12916-022-02329-w`.

[128] D. C. Won, H. M. Feldman, and L. C. Huffman. Sleep Problem Detection and Documentation in Children With Autism Spectrum Disorder and Attention-Deficit/Hyperactivity Disorder by Developmental-Behavioral Pediatricians: A DBPNet Study. *Journal of Developmental & Behavioral Pediatrics*, 40(1):20–31, Jan.

2019. ISSN 0196-206X. doi: 10.1097/DBP.0000000000000624. URL https://journals.lww.com/00004703-201901000-00003.

[129] W. Yin, A. Reichenberg, M. Schnaider Beeri, S. Z. Levine, J. F. Ludvigsson, M. Figee, and S. Sandin. Risk of Parkinson Disease in Individuals With Autism Spectrum Disorder. *JAMA Neurology*, May 2025. ISSN 2168-6149. doi: 10.1001/jamaneurol.2025.1284. URL https://jamanetwork.com/journals/jamaneurology/fullarticle/2834684.

[130] Z. Zheng, P. Zheng, and X. Zou. Association between schizophrenia and autism spectrum disorder: A systematic review and meta-analysis. *Autism Research*, 11(8):1110–1119, 2018. ISSN 1939-3806. doi: 10.1002/aur.1977. URL https://onlinelibrary.wiley.com/doi/abs/10.1002/aur.1977. _eprint: https://onlinelibrary.wiley.com/doi/pdf/10.1002/aur.1977.
