# Supplementary material for: Somatic, Psychiatric and Neurodevelopmental Comorbidities in People with Autism Spectrum Disorder (ASD): A Narrative Review
Source: Healthcare (Basel). 2026 Aug 11;14(16):2492. doi: 10.3390/healthcare14162492 (PMC13512144; doi:10.3390/healthcare14162492)
Supplement: Supplementary file 1 [file healthcare-14-02492-s001.zip › 100f Suppl S3 Comorbidity_Papers___Behavioral_and_Neurodevelopmental___Clean.pdf]

Supplement S3: Behavioral and Neurodevelopmental

1 Behavioral and/or Neurodevelopmental Comorbidity

1.1 Cognitive and Learning (Intellectual) Disorders

Cognitive Disorders

Table 1: Prevalence of cognitive disorders in those with ASD.

Source	Sample	Prevalence	Other Notes
Hand et al. (2020) [14]	n=4685 (ASD), n=46850 (non-ASD); Age: ≥65; Medicare; 2016-17	Cognitive disorder: 25.2% (ASD), 4.9% (non-ASD)	aOR: 8.4(7.7-9.1)
Longo et al. (2024) [28]	n=30258 (ASD), n=60516 (non-ASD); Age: 18-64; IBM Market Scan Commercial Claims and Encounters Database; 2019-20	Cognitive disorder: 1.5% (ASD), 0.3% (non-ASD)	aOR: 3.9 (95% CI: 3.3, 4.7)

Intellectual Disability

Table 2: Prevalence of intellectual disabilities in those with ASD, table 1.

Source	Sample	Prevalence	Other Notes
Schendel et al. (2016) [42]	n=20492 (ASD), 1892412 (non-ASD); Age: >1.5; Danish Civil Registration Service and the Danish Medical Birth Register; born 1980-2010	Mental retardation: 16.0% (ASD), 0.4% (non-ASD)	
Houghton et al. (2017) [16]	n=46943 (ASD, commercial), Truven Health MarketScan Commercial Database, 2014; n=46696 (ASD, Medicaid), Truven Health MarketScan Multi-State Medicaid Database; Age: >3	5.03% (commercial), 23.36% (medicaid)	
Kurzius-Spencer et al. (2018) [25]	ADDM (2006, 2008, 2010 - 14 study sites). 8 yr old.	ASD and ID: 35.1%, ASD only: 64.9%	
Rydzewska et al. (2018) [39]	n=6649 (ASD), n=3739935 (non-ASD); Age: ≥25; Scotland Census data; 2011	29.4% (ASD), 0.4% (non-ASD)	
Bishop-Fitzpatrick et al. (2018) [4]	n=91 (ASD), n=6186 (sex, birth year matched controls); Age: 0-89; EHR from Marshfield clinic - decedents - Wisconsin	Intellectual disability: 75.8% (ASD), 0% (non-ASD)	p:<0.001
Bowden et al. (2020) [6]	n=8955 (ASD), n=1551342 (non-ASD); Age: 0-24; IDI database, New Zealand	Intellectual disability: 29.9% (ASD), 0.4% (non-ASD)	aOR: 61.9 (95% CI: 59.2-64.2) - standardized for age, sex, ethnicity, deprivation, and urban/rural

Table 3: Prevalence of intellectual disabilities in those with ASD, table 2.

Source	Sample	Prevalence	Other Notes
Bougeard et al. (2021) [5]	Review: N=13	ID, Range: 0% - 91.70%	
Chien et al. (2021) [9]	n=3837 (ASD), n=38370 (non-ASD); Children; Taiwan's claims database; 2000, 2005, 2010	Mental retardation: 27.5% (ASD), 1.3% (non-ASD)	
Rødgaard et al. (2021) [40]	n=11,914 (ASD, men), n=4212 (ASD, women); Age ≤16; Danish National Patient Registry	Intellectual disability: 17% (men with ASD), 17% (women with ASD)	
Angell et al. (2021) [2]	n=18355 (ASD, girls), n=65145 (ASD, boys); Age: 1-21; healthcare claims data; integrates ICD-9 and ICD-10 codes	Intellectual disability: 15.11% (ASD, girls), 10.74% (ASD, boys)	aOR: 1.49 (95% CI: 1.42-1.56), p:<0.001 - adjusted for age, race, ethnicity, urbanicity, and insurance
Huang et al. (2021) [18]	n=6599 (ASD), n=26396 (non-ASD); controls age, sex, date matched 1:4; All ages, mean: 5.1 (ASD), 5.8 (non-ASD); Taiwan National Health Insurance Research Database; ICD-9; 2000-15	Intellectual disability: 22.2% (ASD), 0.9% (non-ASD)	p: <0.01
Mutluer et al. (2022) [35]	Review, N=19; population and EHR studies (age 6-18)	Pooled prevalence: 22.9%	
Schott et al. (2022) [43]	n=166,952 (ASD), n=500,856 (non-ASD); Adults; Medicaid; 2008–12	23.53% (ASD), 26.77% (non-ASD)	
Hughes et al. (2023) [19]	n=1846 (ASD); ADDM Network (5 sites, USA); 2018	32% (before age 8), 36.6% (9-16)	
Micai et al. (2023) [32]	Meta-analysis of observational studies, N=27 [N=11 (clinical studies from community samples), N=16 (population/registry based)], n=162,997	Pooled prevalence: 33%, Range: 26% - 41%	

Table 4: Prevalence of intellectual disabilities in those with ASD, table 3.

Source	Sample	Prevalence	Other Notes
Rim et al. (2023) [38]	n=381 (ASD), n=6179 (non-ASD); Age: 0-19; National Health Insurance System National Sample Cohort; South Korea; 2002–13	21.89% (ASD), 0.29% (non-ASD)	aOR: 78.9 (64.45,96.6)
Semovski et al. (2023) [44]	n=2020 (ASD); Age: 12-18; records + interviews; Canada	Intellectual disability: 24.6% (ASD)	
Khachadourian et al. (2023) [23]	n=42569 (ASD), n=11389 (non-ASD siblings); SPARK; Age: <18; 1999-2019	Intellectual disability: 21.7% (ASD), 2% (non-ASD, estimated)	p: <0.05
Hsu et al. (2024) [17]	n=22698 (ASD), [n=19191 (male), n=3507 (female)]; Age: 2-19; Taiwan's National Health Insurance Research Database; 2001-11; ICD-9	Intelligence disability: 26.9% (ASD, total); 24.8% (ASD, male), 38.4% (ASD, female)	RR: 0.54 (95% CI: 0.50-0.58) [Male vs female (ref)] - adjusted for age, income, residence, and parental psychiatric disorders. RR: 0.34 (95% CI: 0.19-0.61) [Parental psychiatric disorders - Male vs female (ref)] - adjusted for age, income, residence
Salehi et al. (2025) [41]	n=2568 (ASD); Age: 3-17; The National Survey of Children's Health; USA; 2020-21	16.1%	OR: 1.5, p = 0.001 [Female vs male]
Lopez-Arvizu et al. (2025) [29]	n=41230 (ASD, ID, or both), n=29001 (ASD), n=27054 (ID); Age: 5-24; Medicaid (6+ months, Midwestern states); 2010-20	Intellectual disability (any): 51.1% (ASD), 100% (ID), 65.6% (combined); Mild: 4.3% (ASD), 7.9% (ID), 5.2% (combined); Moderate: 2.0% (ASD), 7.3% (ID), 4.8% (combined); Severe: 0.8% (ASD), 2.5% (ID), 1.6% (combined); Profound: 0.2% (ASD), 0.9% (ID), 0.6% (combined)	

Developmental Delay/Disability

Table 5: Prevalence of developmental disabilities in those with ASD, table 1.

Source	Sample	Prevalence	Other Notes
Vohra et al. (2017) [48]	n=1772 (ASD), n=5316 (non-ASD); Age: 22-64; three state Medicaid Analytic eXtract; 2000-08	Developmental disorders: 69.5% (ASD), 5.1% (non-ASD)	aOR (95% CI): 44.60 (37.9, 52.4), p:<0.001
Soke et al. (2018) [46]	n=783 (ASD, age: 4), n=1091 (ASD, age; 8), ADDMN - 5 sites (Arizona, Missouri, New Jersey, Utah, and Wisconsin), 2010 survey, 4 and 8 year olds	Developmental Disability - cognitive: 16.73% (ASD, age: 4), 14.57% (ASD, age: 8)	Adjusted prevalence, 95% CI: 0.83 (0.67, 1.05) [8 vs 4 years] - adjusted for sex, ethnicity, maternal education, location
Soke et al. (2018) [46]	n=783 (ASD, age: 4), n=1091 (ASD, age; 8), ADDMN - 5 sites (Arizona, Missouri, New Jersey, Utah, and Wisconsin), 2010	Developmental disability - adaptive: 20.31% (ASD, age: 4), 20.81% (ASD, age: 8)	Adjusted prevalence, 95% CI: 1.13 (0.96, 1.36) [8 vs 4 years] - adjusted for sex, ethnicity, maternal education, location
Dizitzer et al. (2020) [13]	n=459 (ASD), n=2285 (non-ASD); Children, southern Israel, Negev Autism Center database, born 2009-16, controls age, sex, ethnicity matched	Developmental disorder: 12.9% (ASD), 1% (non-ASD)	OR: 14.51 (95% CI: 8.86-23.76), p:<0.001
Angell et al. (2021) [2]	n=18355 (ASD, girls), n=65145 (ASD, boys); Age: 1-21; healthcare claims data; integrates ICD-9 and ICD-10 codes	Developmental disabilities: 78.27% (ASD, girls), 76.66% (ASD, boys)	aOR: 1.16 (95% CI: 1.11-1.22), p:<0.001 - adjusted for age, race, ethnicity, urbanicity, and insurance
Jadav and Bal (2022) [21]	n=4657 (ASD); Adults; online survey, SPARK; 2022	Any developmental disorder: 30.3%	

Table 6: Prevalence of developmental disabilities in those with ASD, table 2.

Source	Sample	Prevalence	Other Notes
Shoaib et al. (2022) [45]	n=29694 (ASD, medicaid), IBM Marketscan Multi-State Medicaid Database; n=6719 (ASD, Optum), Optum De-Identified Clinformatics Data Mart Database, Date of Death; US; 2015-16	Developmental delay: 13.6% (Optum), 14.91% (Medicaid)	
Salehi et al. (2025) [41]	n=2568 (ASD); Age: 3-17; The National Survey of Children's Health; USA; 2020-21	Developmental delay: 64.0%	
Jadav and Bal (2022) [21]	n=4657 (ASD); Adults; online survey, SPARK; 2022	Intellectual disability, cognitive impairment, global developmental delay, or borderline intellectual functioning: 6.1%	
Davignon, et al. (2018) [11]	n=4123 (ASD), n=20615 (non-ASD, non-ADHD); Age: 14-25; Kaiser Permanente Northern California, 2013-15	Global Developmental Delay: 1.9% (ASD), 0.05% (non-ASD)	aOR (95% CI): 42.81 (27.56-66.50)

Table 7: Prevalence of developmental disabilities in those with ASD, table 3.

**Source**	**Sample**	**Prevalence**	**Other Notes**
Davignon, et al. (2018) [11]	n=4123 (ASD), n=20615 (non-ASD, non-ADHD); Age: 14-25; Kaiser Permanente Northern California, 2013-15	Learning disorder: 0.6% (ASD), 0.04% (non-ASD)	aOR (95% CI): 16.16 (7.45-35.03)
Bhat et al. (2020) [3]	n=11814 (ASD); SPARK; Children; DCD questionnaire	Learning disability: 17.9% (diagnosed)	
Chien et al. (2021) [9]	n=3837 (ASD), n=38370 (non-ASD); Children; Taiwan's claims database; 2000, 2005, 2010	Learning disorder: 61.0% (ASD), 6.0% (non-ASD)	
Micai et al. (2023) [32]	Meta-analysis of observational studies, N=1, n=122	Specific learning disorder: Pooled prevalence: 13%, Range: 8% - 20%	
Khachadourian et al. (2023) [23]	n=42569 (ASD), n=11389 (non-ASD siblings); SPARK; Age: <18 at time of registration; 1999-2019	Learning disability: 23.5% (ASD), 4% (non-ASD, estimatedly)	p: <0.05
Semovski et al. (2023) [44]	n=2020 (ASD); Age: 12-18; records + interviews; Canada	Learning or communication disorder: 37.3% (ASD)	

1.2 Disorders of Attentional Controls

1.2.1 ADD/ADHD

Table 8: Prevalence of ADHD in those with ASD, table 1.

Source	Sample	Prevalence	Other Notes
Croen et al. (2015) [10]	n=1507 (ASD), n=15070 (age, sex matches controls); Adults; Kaiser Permanente Northern California; 2008-12	ADD: 11.08% (ASD), 1.95% (non-ASD)	Chi-square p value: <0.0001
Schendel et al. (2016) [42]	n=20492 (ASD), 1892412 (non-ASD); Age: >1.5; Danish Civil Registration Service and the Danish Medical Birth Register; born 1980-2010	ADHD: 27.6% (ASD), 1.5% (non-ASD)	
Vohra et al. (2017) [48]	n=1772 (ASD), n=5316 (non-ASD); Age: 22-64; three state Medicaid Analytic eXtract; 2000-08	ADHD: 8.2% (ASD), 0.7% (non-ASD)	aOR (95% CI): 12.89 (8.94, 18.5), p:<0.001
Houghton et al. (2017) [16]	n=46943 (ASD, commercial), Truven Health MarketScan Commercial Database, 2014; n=46696 (ASD, Medicaid), Truven Health MarketScan Multi-State Medicaid Database; Age: >3	ADHD: 37.82% (commercial), 40.49% (medicaid)	
Soke et al. (2018) [46]	n=783 (ASD, age: 4), n=1091 (ASD, age; 8), ADDMN - 5 sites (Arizona, Missouri, New Jersey, Utah, and Wisconsin), 2010	ADHD: 5.49% (ASD, age: 4), 26.05% (ASD, age: 8)	Adjusted prevalence, 95% CI: 4.78 (3.40, 6.73) [8 vs 4 years] - adjusted for sex, ethnicity, maternal education, location
Lai, et al. (2019) [26]	Meta-analysis, N=96	28%	
Margari et al. (2019) [30]	n=100 (ASD, male), n=59 (ASD, female); Children and adolescents, mean: 9.91 (ASD, male), mean: 10.97 (ASD, female); Child and Adolescent Neuropsychiatry Unit; 2016-18	ADHD: 32% (ASD, male), 42.7% (ASD, female)	p: 0.156
Mosner et al. (2019) [34]	n=67 (ASD); Age: 8-25; UNC Autism Research Registry; MINI-KID-P assessment and caregiver report	Any ADHD: 71.4% (MINI-KID-P), 34.3% (caregiver)	
Hossain et al. (2020) [15]	Umbrella Review, N=3	ADHD; 25.7% - 65%	

Table 9: Prevalence of ADHD in those with ASD, table 2.

Source	Sample	Prevalence	Other Notes
Hand et al. (2020) [14]	n=4685 (ASD), n=46850 (non-ASD); Age: ≥65; Medicare; 2016-17	Attention deficit disorders: 2.5% (ASD), 0.1% (non-ASD)	aOR: 22.4 (16.2-31.0)
Bhat et al. (2020) [3]	n=11814 (ASD); SPARK; Children; DCD questionnaire	ADHD: 40.8%	
Bowden et al. (2020) [6]	n=8955 (ASD), n=1551342 (non-ASD); Age: 0-24; IDI database, New Zealand	ADHD: 26.8% (ASD), 1.2% (non-ASD)	aOR: 13.8 (95% CI: 13.42-14.3) - standardized for age, sex, ethnicity, deprivation, and urban/rural
Bougeard et al. (2021) [5]	Review: N=17	ADHD, Range: 0% - 86%	
Chien et al. (2021) [9]	n=3837 (ASD), n=38370 (non-ASD); Children; Taiwan's claims database; 2000, 2005, 2010	54.6% (ASD), 6.2% (non-ASD)	
Rødgaard et al. (2021) [40]	n=11,914 (ASD, men), n=4212 (ASD, women); Age ≤16; Danish National Patient Registry	ADHD: 35% (men with ASD), 26% (women with ASD)	
Ivanović (2021) [20]	n=152 (ASD); Mean age: 8.02 (4.26); Autism Center, Montenegro	ADHD: 17.76%	
Angell et al. (2021) [2]	n=18355 (ASD, girls), n=65145 (ASD, boys); Age: 1-21; healthcare claims data; integrates ICD-9 and ICD-10 codes	ADHD: 55.15% (ASD, girls), 61.03% (ASD, boys)	aOR: 0.76 (95% CI: 0.74-0.79), p:<0.001 - adjusted for age, race, ethnicity, urbanicity, and insurance
Huang et al. (2021) [18]	n=6599 (ASD), n=26396 (non-ASD); controls age, sex, date matched 1:4; All ages, mean: 5.1 (ASD), 5.8 (non-ASD); Taiwan National Health Insurance Research Database; ICD-9; 2000-15	ADHD: 23.1% (ASD), 1.0% (non-ASD)	p: <0.01

Table 10: Prevalence of ADHD in those with ASD, table 3.

Source	Sample	Prevalence	Other Notes
Shoaib et al. (2022) [45]	n=29694 (ASD, medicaid), IBM Marketscan Multi-State Medicaid Database; n=6719 (ASD, Optum), Optum De-Identified Clinformatics Data Mart Database, Date of Death; US; 2015-16	ADHD: 44.16% (Optum), 50.49% (Medicaid)	
Jadav and Bal (2022) [21]	n=4657 (ASD); Adults; online survey, SPARK; 2022	ADD/ADHD: 44%	
Mutluer et al. (2022) [35]	Review, N=15; population and EHR studies (age 6-18)	Prevalence in studies: 28% - 83%	
Schott et al. (2022) [43]	n=155,617 (ASD), n=466,851 (non-ASD); Adults; Medicaid; 2008–12	Attention deficit disorder: 20.14% (ASD), 8.32% (non-ASD)	aOR: 2.02 (1.98 2.07)
Underwood et al. (2022) [47]	n=7943 (core ASD), n=25941 (control); Age: $\geq$18; UK; SAIL Databank, 2001-16	7.0% (ASD), 0.7% (non-ASD)	
Koceski and Trajkovski (2022) [24]	n=72 (ASD), n=75 (non-ASD); Age: 3-24; Rochester Health Status Survey IV, Macedonia; 2019	ADD/ADHD: 19.4% (ASD), 0% (non-ASD)	p: 0.000
Hughes et al.(2023) [19]	n=1846 (ASD); ADDM Network (5 sites, USA); 2018	28% had ADHD	
Micai et al. (2023) [32]	Meta-analysis of observational studies, N=72 [N=49 (clinical studies from community samples), N=23 (population/registry based)], n=208,789	Pooled prevalence: 37%, Range: 28% - 46%	
Rim et al. (2023) [38]	n=381 (ASD), n=6179 (non-ASD); Age: 0-19; National Health Insurance System National Sample Cohort; South Korea; 2002–13	23.81% (ASD), 1.79% (non-ASD)	aOR: 13.03 (10.94,15.52)

Table 11: Prevalence of ADHD in those with ASD, table 4.

Source	Sample	Prevalence	Other Notes
Semovski et al. (2023) [44]	n=2020 (ASD); Age: 12-18; records + interviews; Canada	ADHD: 48.9% (ASD)	
Khachadourian et al. (2023) [23]	n=42569 (ASD), n=11389 (non-ASD siblings); SPARK; Age: <18 at time of registration; 1999-2019	ADHD: 35.3% (ASD), 16.8% (non-ASD)	p: <0.05
O'Nions et al. (2024) [36]	n=13,172 (ASD, men, no ID), n=3958 (ASD, women, no ID) n=4850 (ASD+ID, men) n=1600 (ASD+ID, women); comparison are 1:10 matched by age, sex, ID status, general practice registered to; mortality study; UK; 1989-2019	ADHD: Men: 15.55% (ASD, no ID); 1.91% (comparison). Women: 9.42% (ASD, no ID); 0.43% (comparison). Men: 12.62% (ASD + ID); 1.49% (comparison). Women: 7.88% (ASD + ID); 0.26% (comparison).	
Longo et al. (2024) [28]	n=30258 (ASD), n=60516 (non-ASD); Age: 18-64; IBM Market Scan Commercial Claims and Encounters Database; 2019-20	ADHD: 34.1% (ASD), 7.4% (non-ASD)	aOR: 6.5 (95% CI: 6.3, 6.8)
Hsu et al. (2024) [17]	n=22698 (ASD), [n=19191 (male), n=3507 (female)]; Age: 2-19; Taiwan's National Health Insurance Research Database; 2001-11; ICD-9	ADHD: 46.9% (ASD, total); 48.6% (ASD, male), 37.1% (ASD, female)	RR: 1.63 (95% CI: 1.52-1.76) [Male vs female (ref)] - adjusted for age, income, residence, and parental psychiatric disorders.
Salehi et al. (2025) [41]	n=2568 (ASD); Age: 3-17; The National Survey of Children's Health; USA; 2020-21	44.3%	OR: 0.8, p = 0.007 [Female vs Male]
Diemer et al. (2025) [12]	n=1424 (ASD); Adult; SPARK	ADHD: 46.07% (total), 46.73% (diagnosed <18), 45.65% (diagnosed >18)	p-value 0.699 (between age groups)

1.2.2 Conduct and Oppositional Disorders

Conduct, Behavior, and Disruptive Disorders

Table 12: Prevalence of combined, overlapping, and unspecified conduct, behavior, and disruptive disorders in those with ASD.

Source	Sample	Prevalence	Other Notes
Schendel et al. (2016) [42]	n=20492 (ASD), 1892412 (non-ASD); Age: >1.5; Danish Civil Registration Service and Medical Birth Register; born 1980-2010	Behavioral and emotional disorders with juvenile onset: 39.4% (ASD), 2.4% (non-ASD)	
Davignon, et al. (2018) [11]	n=4123 (ASD), n=20615 (non-ASD, non-ADHD); Age: 14-25; Kaiser Permanente Northern California, 2013-15	Disruptive, impulse, and conduct disorders: 3.5% (ASD), 0.3% (non-ASD)	aOR (95% CI): 11.93 (8.75 - 16.26)
Lai, et al. (2019) [26]	Meta-analysis, N=96	Disruptive/ impulsive/ conduct disorder: 12%	
Hossain et al. (2020) [15]	Umbrella Review, N=3	Disruptive, impulse-control, and conduct disorders: 12% - 48%	
Huang et al. (2021) [18]	n=6599 (ASD), n=26396 (non-ASD); controls age, sex, date matched 1:4; All ages, mean: 5.1 (ASD), 5.8 (non-ASD); Taiwan National Health Insurance Research Database; ICD-9; 2000-15	Impulse control disorder: 3.4% (ASD), 0.5% (non-ASD)	p: <0.01
Jadav and Bal (2022) [21]	n=4657 (ASD); Adults; online survey, SPARK; 2022	Disruptive disorder: 45.5%	
Rim et al. (2023) [38]	n=381 (ASD), n=6179 (non-ASD); Age: 0-19; National Health Insurance System National Sample Cohort; South Korea; 2002–13	Behavioral and emotional disorders: 31.99% (ASD), 3.33% (non-ASD)	aOR: 10.82 (9.25,12.65)
Salehi et al. (2025) [41]	n=2568 (ASD); Age: 3-17; The National Survey of Children's Health; USA; 2020-21	Behavioral and Conduct Problems: 57.8%	

Conduct Disorders

Table 13: Prevalence of conduct disorders in those with ASD.

Source	Sample	Prevalence	Other Notes
Lever and Geurts (2016) [27]	n=138 (ASD), n=170 (non-ASD); Age: 19-79; Mental Health Institutions and online recruitment; Netherlands	Conduct disorder: 2.2% (ASD), 0.6% (non-ASD)	
Houghton et al. (2017) [16]	n=46943 (ASD, commercial), Truven Health MarketScan Commercial Database, 2014; n=46696 (ASD, Medicaid), Truven Health MarketScan Multi-State Medicaid Database; Age: >3	Conduct disorder: 11.82% (commercial), 19.34% (medicaid)	
Mosner et al. (2019) [34]	n=67 (ASD); Age: 8-25; UNC Autism Research Registry; MINI-KID-P assessment and caregiver report	Conduct disorder: 5.7% (MINI-KID-P), 0% (caregiver report)	
Ivanović (2021) [20]	n=152 (ASD); Mean age: 8.02 (4.26); Autism Center, Montenegro	Conduct disorder: 13.10%	
Bowden et al. (2020) [6]	n=8955 (ASD), n=1551342 (non-ASD); Age: 0-24; IDI database, New Zealand	Conduct: 1.2% (ASD), 0.1% (non-ASD)	aOR: 7.2 (95% CI: 5.8-8.6) - standardized: age, sex, ethnicity, deprivation, urban/rural
Rødgaard et al. (2021) [40]	n=11,914 (ASD, men), n=4212 (ASD, women); Age: $\leq$16; Danish National Patient Registry	Conduct disorder; 5% (men with ASD), 3% (women with ASD)	
Yule (2021) [1]	n=206 (ASD), n=156 (ADHD), n=156 (controls, age matched); age: $\geq$ 12; 2007-16; high functioning ASD - academic center	Conduct disorders: 12% (ASD), 22% (ADHD), 3% (control)	p: (ASD vs ADHD), p: <0.05 (ASD vs controls), p: <0.001 (ADHD vs controls)
Khachadourian et al. (2023) [23]	n=42569 (ASD), n=11389 (non-ASD siblings); SPARK; 1999-2019; Age: <18 (registration)	Conduct Disorder, estimatedly: 3% (ASD), 1% (non-ASD)	p: <0.05
Diemer et al. (2025) [12]	n=1424 (ASD); Adult; SPARK	Conduct: 1.05% (total), 2.04% (diagnosed <18), 0.54% (diagnosed >18)	p-value 0.008 (between age groups)

Disruptive Disorder

Table 14: Prevalence of disruptive disorders in those with ASD, table 2.

Source	Sample	Prevalence	Other Notes
Brookman-Frazee et al. (2018) [7]	n=201 (ASD); Age: 4-14; data from community effectiveness trial; Southern California; 2012-15; MINI-KID-P	Oppositional defiant disorder: 58% (ASD)	
Soke et al. (2018) [46]	n=783 (ASD, age: 4), n=1091 (ASD, age; 8), ADDMN - 5 sites (Arizona, Missouri, New Jersey, Utah, and Wisconsin), 2010	ODD: 0.89% (ASD, age: 4), 4.03% (ASD, age: 8)	Adjusted prevalence, 95% CI: 4.13 (1.75, 9.75) [8 vs 4 years] - adjusted for sex, ethnicity, maternal education, location
Mosner et al. (2019) [34]	n=67 (ASD); Age: 8-25; UNC Autism Research Registry; MINI-KID-P assessment and caregiver report	ODD: 40.0% (MINI-KID-P), 0% (caregiver report)	
Bowden et al. (2020) [6]	n=8955 (ASD), n=1551342 (non-ASD); Age: 0-24; IDI database, New Zealand	ODD: 5.1% (ASD), 0.2% (non-ASD)	aOR: 17.8 (95% CI: 16.0-19.6) - standardized for age, sex, ethnicity, deprivation, and urban/rural
Ivanović (2021) [20]	n=152 (ASD); Autism Center, Montenegro	Disruptive mood dysregulation disorder: 9.87%	
Yule (2021) [1]	n=206 (ASD), n=156 (ADHD), n=156 (controls, age matched); age: $\geq$ 12; 2007-16; high functioning ASD - academic center	ODD/Antisocial personality disorder: 52% (ASD), 54% (ADHD), 9% (control)	p: ns (ASD vs ADHD), p: <0.001 (ASD vs controls), p: <0.001 (ADHD vs controls)
Mutluer et al. (2022) [35]	Review, N=10, population and EHR studies (age 6-18)	Disruptive Behavior Disorder: 12% - 48%	
Micai et al. (2023) [32]	Meta-analysis of observational studies, N=24 [N=18 (clinical studies from community samples), N=6 (population/registry based)], n=18,842	Disruptive Behavior: Pooled prevalence: 28%, Range: 21% - 36%	

Table 15: Prevalence of disruptive disorders in those with ASD, table 2.

Source	Sample	Prevalence	Other Notes
Micai et al. (2023) [32]	Meta-analysis of observational studies, N=42 [N=26 (clinical studies from community samples), N=16 (population/registry based)], n=163,225	Disruptive Impulse Control Disorder: Pooled prevalence: 17%, Range: 13% - 22%	
Semovski et al. (2023) [44]	n=2020 (ASD); Age: 12-18; records + interviews; Canada	Disruptive behavior disorder: 19.5% (ASD)	
Khachadourian et al. (2023) [23]	n=42569 (ASD), n=11389 (non-ASD siblings); SPARK; Age: <18 at time of registration; 1999-2019	Disruptive mood dysregulation disorder, estimated: 3% (ASD), 1% (non-ASD)	p: <0.05
Khachadourian et al. (2023) [23]	n=42569 (ASD), n=11389 (non-ASD siblings); SPARK; Age: <18 at time of registration; 1999-2019	ODD, estimated: 7% (ASD), 3% (non-ASD)	p: <0.05
Hsu et al. (2024) [17]	n=22698 (ASD), [n=19191 (male), n=3507 (female)]; Age: 2-19; Taiwan's National Health Insurance Research Database; 2001-11; ICD-9	Disruptive behavior disorder: 6.4% (ASD, total); 6.6% (ASD, male), 5.1% (ASD, female)	RR: 1.38 (95% CI: 1.17-1.62) [Male vs female (ref)] - adjusted for age, income, residence, and parental psychiatric disorders.
Mayes et al. (2024) [31]	n=2400 (ASD) [n = 1,077 Autism+ADHDC, 120 Autism+ADHD-I, 152 Autism Only, 743 ADHD-C Only, and 308 ADHD-I only]; Age: 3-17; [ADHD-Combined presentation (ADHD-C) (i.e., children who have hyperactivity, impulsivity, and inattention); ADHD-Inattentive presentation (ADHD-I).]; Parent completed Pediatric Behavior Scale	ODD: 23.7% (ASD, only), 61.9% (ASD+ADHD-C), 27.5% (ASD+ADHD-I), 53.3% (ADHD-C, only), 14.3% (ADHD-I, only)	
Diemer et al. (2025) [12]	n=1424 (ASD); Adult; SPARK	ODD: 4.85% (total), 8.57% (diagnosed <18), 2.79% (diagnosed >18)	p-value <0.001 (between age groups)

1.3 Speech and Language Disorders

Table 16: Prevalence of speech and language disorders in those with ASD.

Source	Sample	Prevalence	Other Notes
Davignon, et al. (2018) [11]	n=4123 (ASD), n=20615 (non-ASD, non-ADHD); Age: 14-25; Kaiser Permanente Northern California, 2013-15	Language Disorder: 1.8% (ASD), 0.01% (non-ASD)	aOR (95% CI): 194.78 (47.62-796.57)
Soke et al. (2018) [46]	n=783 (ASD, age: 4), n=1091 (ASD, age; 8), ADDMN - 5 sites (Arizona, Missouri, New Jersey, Utah, and Wisconsin), 2010	Language disorder: 26.05% (ASD, age: 4), 34.83% (ASD, age: 8)	Adjusted prevalence, 95% CI: 1.39 (1.19, 1.62) [8 vs 4 years] - adjusted for sex, ethnicity, maternal education, location
Dizitzer et al. (2020) [13]	n=459 (ASD), n=2285 (non-ASD); Children, southern Israel, Negev Autism Center database, born 2009-16, controls age, sex, ethnicity matched	Developmental disorder; speech/language: 9.2% (ASD), 0.7% (non-ASD)	OR: 13.44 (95% CI: 7.58-23.83), p:<0.001
Micai et al. (2023) [32]	Meta-analysis of observational studies, N=4 [N=1 (clinical studies from community samples), N=3 (population/registry based)], n=8979	Language disorder: Pooled prevalence: 16%, Range: 0% - 53%	
Khachadourian et al. (2023) [23]	n=42569 (ASD), n=11389 (non-ASD siblings); SPARK; Age: <18 at time of registration; 1999-2019	Social communication disorder, estimated: 19% (ASD), 2% (non-ASD)	p: <0.05

1.4 Motor disorders

Tic and Tourettes

Table 17: Prevalence of movement disorders in those with ASD, table 1.

Source	Sample	Prevalence	Other Notes
Kalyva et al. (2016) [22]	Review - N=20	Tourette syndrome: 4% - 5%, Tic Disorder: 9-12%	
Davignon, et al. (2018) [11]	n=4123 (ASD), n=20615 (non-ASD, non-ADHD); Age: 14-25; Kaiser Permanente Northern California, 2013-15	Tic Disorder: 1.48% (ASD), 0.02% (non-ASD)	59.36 (23.69 - 148.73)
Mosner et al. (2019) [34]	n=67 (ASD); Age: 8-25; UNC Autism Research Registry; MINI-KID-P assessment and caregiver report	Tourette's Disorder: 5.7% (MINI-KID-P), 2.9% (caregiver report)	
Chien et al. (2021) [9]	n=3837 (ASD), n=38370 (non-ASD); Children; Taiwan's claims database; 2000, 2005, 2010	Tic disorder: 5.9% (ASD), 1.3% (non-ASD)	
Rødgaard et al. (2021) [40]	n=11,914 (ASD, men), n=4212 (ASD, women); Age $\leq$16; Danish National Patient Registry	Tic disorder: 9% (men with ASD), 4% (women with ASD)	
Huang et al. (2021) [18]	n=6599 (ASD), n=26396 (non-ASD); controls age, sex, date matched 1:4; All ages, mean: 5.1 (ASD), 5.8 (non-ASD); Taiwan National Health Insurance Research Database; ICD-9; 2000-15	Tic Disorder: 16.9% (ASD), 0.8% (non-ASD)	p: <0.01
Jadav and Bal (2022) [21]	n=4657 (ASD); Adults; online survey, SPARK; 2022	Tourette's or tic disorder: 4.1%	
Koceski and Trajkovski (2022) [24]	n=72 (ASD), n=75 (non-ASD); Age: 3-24; Rochester Health Status Survey IV, Macedonia; 2019	Tics: 6.9% (ASD), 2.6% (non-ASD)	p:0.25
Micai et al. (2023) [32]	Meta-analysis of observational studies, N=38 [N=30 (clinical studies from community samples), N=8	Tic disorder: Pooled prevalence: 10%, Range: 8% - 13%	

Table 18: Prevalence of movement disorders in those with ASD, table 2.

Source	Sample	Prevalence	Other Notes
Rim et al. (2023) [38]	n=381 (ASD), n=6179 (non-ASD); Age: 0-19; National Health Insurance System National Sample Cohort; South Korea; 2002–13	Tic disorder: 3.05% (ASD), 0.52% (non-ASD)	aOR: 4.02 (2.66,6.05)
Khachadourian et al. (2023) [23]	n=42569 (ASD), n=11389 (non-ASD siblings); SPARK; Age: <18 at time of registration; 1999-2019	Tourette syndrome or tic disorder, estimated: 3% (ASD), 1% (non-ASD)	p: <0.05
Salehi et al. (2025) [41]	n=2568 (ASD); Age: 3-17; The National Survey of Children's Health; USA; 2020-21	Tourette syndrome: 1.7%	

Cerebral Palsy

Table 19: Prevalence of cerebral palsy in those with ASD.

Source	Sample	Prevalence	Other Notes
Croen et al. (2015) [10]	n=1507 (ASD), n=15070 (age, sex matches controls); Adults; Kaiser Permanente Northern California; 2008-12	3.58% (ASD), 0.21% (non-ASD)	Chi-square p value: <0.001
Davignon, et al. (2018) [11]	n=4123 (ASD), n=20615 (non-ASD, non-ADHD); Age: 14-25; Kaiser Permanente Northern California, 2013-15	Cerebral palsy and other paralytics syndromes: 2.3% (ASD), 0.3% (non-ASD)	aOR (95% CI): 7.37 (5.29 - 10.29)
Chien et al. (2021) [9]	n=3837 (ASD), n=38370 (non-ASD); Children; Taiwan's claims database; 2000, 2005, 2010	Cerebral Palsy: 7.5% (ASD), 0.5% (non-ASD)	
Pan et al. (2021) [37]	Review, N=17 [N=7 (case control), N=10 (prevalence)], n=39692	Cerebral Palsy: Range: 0.8% - 13.9%	Pooled estimate of proportion: 2.8 (1.6-4.2)
Schott et al. (2022) [43]	n=155,617 (ASD), n=466,851 (non-ASD); Adults; Medicaid; 2008–12	Cerebral palsy: 6.96% (ASD), 10.72% (non-ASD)	aOR: 0.61 (0.59 0.63)
Salehi et al. (2025) [41]	n=2568 (ASD); Age: 3-17; The National Survey of Children's Health; USA; 2020-21	Cerebral Palsy: 1.2%	OR: 2.2, p = 0.042 [Female vs Male]

Table 20: Prevalence of ASD in those with cerebral palsy.

Source	Sample	Prevalence	Other Notes
Casseus et al. (2024) [8]	n=994 (cerebral palsy); Age: 3-26; electronic health records; 2016-21	ASD: 8.2% (CP)	aOR: 2.17 (1.22-3.84), p=0.0080 [Male vs Female]; aOR: 0.53 (95% CI: 0.29-0.99), p=0.0455 [spastic -bilateral vs non-spastic]

Coordination

Table 21: Prevalence of coordination disorders in those with ASD.

Source	Sample	Prevalence	Other Notes
Soke et al. (2018) [46]	n=783 (ASD, age: 4), n=1091 (ASD, age; 8), ADDMN - 5 sites (Arizona, Missouri, New Jersey, Utah, and Wisconsin), 2010	Developmental disability - motor: 21.58% (ASD, age: 4), 26.86% (ASD, age: 8)	Adjusted prevalence, 95% CI: 1.30 (1.08, 1.56) [8 vs 4 years] - adjusted for sex, ethnicity, maternal education, location
Miller et al. (2021) [33]	n=43 (ASD), n=18 (Developmental coordination disorder); Age: 5-19; community sampled recruited via fliers and internet notices from 2 projects	Motor coordination: Very low: 46.5% (ASD), 55.6% (DCD), Low: 14% (ASD), 22.2% (DCD), Below average: 20.9% (ASD), 16.7% (DCD), Average: 16.3% (ASD), 5.6% (DCD)	
Bhat et al. (2020) [3]	n=11814 (ASD); SPARK; Children; DCD questionnaire	Developmental coordination disorder: 15.1% (diagnosed); Motor impairment - at risk for DCD: 86.9% (based on DCDQ scores)	
Micai et al. (2023) [32]	Meta-analysis of observational studies, N=2 [N=2 (clinical studies from community samples), N=0 (population/registry based)], n=11857	Developmental Coordination Disorder: Pooled prevalence: 87%, Range: 87% - 88%	
Micai et al. (2023) [32]	Meta-analysis of observational studies, N=12 [N=6 (clinical studies from community samples), N=6 (population/registry based)], n=25945	Motor Problem: Pooled prevalence: 40%, Range: 19% - 64%	
Khachadourian et al. (2023) [23]	n=42569 (ASD), n=11389 (non-ASD siblings); SPARK; Age: <18 at start; 1999-2019	Motor delay, estimated: 18% (ASD), 3% (non-ASD)	p: <0.05

References

[1] Amy Yule, Maura DiSalvo, Joseph Biederman, Timothy E Wilens, Nina T Dallenbach, Daria Taubin, and Gagan Joshi. Decreased Risk for Substance Use Disorders in Individuals with High-functioning Autism Spectrum Disorder - PMC. *European Child & Adolescent Psychiatry*, 32(2):257–265, Aug. 2021. doi: 10.1007/s00787-021-01852-0. URL https://pmc.ncbi.nlm.nih.gov/articles/PMC8936975/.

[2] A. M. Angell, A. Deavenport-Saman, L. Yin, B. Zou, C. Bai, D. Varma, and O. Solomon. Sex Differences in Co-occurring Conditions among Autistic Children and Youth in Florida: A Retrospective Cohort Study (2012-2019). *Journal of autism and developmental disorders*, 51(10):3759–3765, Oct. 2021. ISSN 0162-3257. doi: 10.1007/s10803-020-04841-5. URL https://www.ncbi.nlm.nih.gov/pmc/articles/PMC8254818/.

[3] A. N. Bhat. Is Motor Impairment in Autism Spectrum Disorder Distinct From Developmental Coordination Disorder? A Report From the SPARK Study. *Physical Therapy*, 100(4):633–644, Apr. 2020. ISSN 1538-6724. doi: 10.1093/ptj/pzz190.

[4] L. Bishop-Fitzpatrick, A. Movaghar, J. S. Greenberg, D. Page, L. S. DaWalt, M. H. Brilliant, and M. R. Mailick. Using machine learning to identify patterns of lifetime health problems in decedents with autism spectrum disorder. *Autism Research*, 11(8):1120–1128, Aug. 2018. ISSN 1939-3792. doi: 10.1002/aur.1960. URL https://onlinelibrary.wiley.com/doi/full/10.1002/aur.1960.

[5] C. Bougeard, Françoise Picarel-Blanchot, Ramona Schmid, Rosanne Campbell, and Jan Buitelaar. Prevalence of Autism Spectrum Disorder and Comorbidities in Children and Adolescents: A Systematic Literature Review. *ResearchGate*, Oct. 2021. doi: 10.3389/fpsyt.2021.744709.

[6] N. Bowden, H. Thabrew, J. Kokaua, R. Audas, B. Milne, K. Smiler, H. Stace, B. Taylor, and S. Gibb. Autism spectrum disorder/Takiwātanga: An Integrated Data Infrastructure-based approach to autism spectrum disorder research in New Zealand - Nicholas Bowden, Hiran Thabrew, Jesse Kokaua, Richard Audas, Barry Milne, Kirsten Smiler, Hilary Stace, Barry Taylor, Sheree Gibb, 2020. *Autism*, 24(8):2213–2227, 2020. doi: https://doi.org/10.1177/1362361320939329. URL https://journals.sagepub.com/doi/full/10.1177/1362361320939329.

[7] L. Brookman-Frazee, N. Stadnick, C. Chlebowski, M. Baker-Ericzén, and W. Ganger. Characterizing psychiatric comorbidity in children with autism spectrum disorder receiving publicly funded mental health services. *Autism*, 22(8):938–952, Nov. 2018. ISSN 1362-3613, 1461-7005. doi: 10.1177/1362361317712650. URL https://journals.sagepub.com/doi/10.1177/1362361317712650.

[8] M. Casseus, H. A. Shoval, A. J. Erasmus, and J. Cheng. Clinical and functional characteristics of co-occurring cerebral palsy and autism spectrum disorder among children and young adults. *Research in Autism Spectrum Disorders*, 118:102490, Oct. 2024. ISSN 1750-9467. doi: 10.1016/j.rasd.2024.102490. URL `https://www.sciencedirect.com/science/article/pii/S175094672400165X`.

[9] Y.-L. Chien, C.-S. Wu, and H.-J. Tsai. The Comorbidity of Schizophrenia Spectrum and Mood Disorders in Autism Spectrum Disorder. *Autism Research*, 14(3):571–581, 2021. ISSN 1939-3806. doi: 10.1002/aur.2451. URL `https://onlinelibrary.wiley.com/doi/abs/10.1002/aur.2451`. _eprint: https://onlinelibrary.wiley.com/doi/pdf/10.1002/aur.2451.

[10] L. A. Croen, O. Zerbo, Y. Qian, M. L. Massolo, S. Rich, S. Sidney, and C. Kripke. The health status of adults on the autism spectrum. *Autism*, 19(7):814–823, Oct. 2015. ISSN 1362-3613, 1461-7005. doi: 10.1177/1362361315577517. URL `https://journals.sagepub.com/doi/10.1177/1362361315577517`.

[11] M. N. Davignon, Y. Qian, M. Massolo, and L. A. Croen. Psychiatric and Medical Conditions in Transition-Aged Individuals With ASD. *Pediatrics*, 141(Supplement_4):S335–S345, Apr. 2018. ISSN 0031-4005. doi: 10.1542/peds.2016-4300K. URL `https://doi.org/10.1542/peds.2016-4300K`.

[12] M. C. Diemer, R. Ros-Demarize, C. C. Bradley, S. Kanne, S. H. Kim, J. Parish-Morris, L. G. Snyder, E. Wodka, S. Consortium, and L. A. Carpenter. Comparative Analysis of Autistic Women Across the Lifespan: Childhood vs. Adulthood Diagnosis. *Autism Research*, n/a (n/a), June 2025. ISSN 1939-3806. doi: 10.1002/aur.70073. URL `https://onlinelibrary.wiley.com/doi/abs/10.1002/aur.70073`. _eprint: https://onlinelibrary.wiley.com/doi/pdf/10.1002/aur.70073.

[13] Y. Dizitzer, G. Meiri, H. Flusser, A. Michaelovski, I. Dinstein, and I. Menashe. Comorbidity and health services' usage in children with autism spectrum disorder: a nested case–control study. *Epidemiology and Psychiatric Sciences*, 29:e95, Jan. 2020. ISSN 2045-7960, 2045-7979. doi: 10.1017/S2045796020000050. URL `https://www.cambridge.org/core/journals/epidemiology-and-psychiatric-sciences/article/co`

[14] B. N. Hand, A. M. Angell, L. Harris, and L. A. Carpenter. Prevalence of physical and mental health conditions in Medicare-enrolled, autistic older adults. *Autism*, 24(3):755–764, Apr. 2020. ISSN 1362-3613. doi: 10.1177/1362361319890793. URL `https://www.ncbi.nlm.nih.gov/pmc/articles/PMC7433648/`.

[15] M. M. Hossain, N. Khan, A. Sultana, P. Ma, E. L. J. McKyer, H. U. Ahmed, and N. Purohit. Prevalence of comorbid psychiatric disorders

among people with autism spectrum disorder: An umbrella review of systematic reviews and meta-analyses. *Psychiatry Research*, 287:112922, May 2020. ISSN 0165-1781. doi: 10.1016/j.psychres.2020.112922. URL https://www.sciencedirect.com/science/article/pii/S0165178120300093.

[16] R. Houghton, R. C. Ong, and F. Bolognani. Psychiatric comorbidities and use of psychotropic medications in people with autism spectrum disorder in the United States. *Autism Research*, 10 (12):2037–2047, 2017. ISSN 1939-3806. doi: 10.1002/aur.1848. URL https://onlinelibrary.wiley.com/doi/abs/10.1002/aur.1848. _eprint: https://onlinelibrary.wiley.com/doi/pdf/10.1002/aur.1848.

[17] J.-W. Hsu, L.-C. Chen, K.-L. Huang, S.-J. Tsai, Y.-M. Bai, T.-P. Su, T.-J. Chen, and M.-H. Chen. The influence of sex on major psychiatric comorbidities and parental psychiatric disorders in 22,698 children and adolescents with ICD-9-CM-based autism spectrum disorder. *European Archives of Psychiatry and Clinical Neuroscience*, 274(1):19–25, Feb. 2024. ISSN 1433-8491. doi: 10.1007/s00406-022-01529-y. URL https://doi.org/10.1007/s00406-022-01529-y.

[18] J.-S. Huang, F.-C. Yang, W.-C. Chien, T.-C. Yeh, C.-H. Chung, C.-K. Tsai, S.-J. Tsai, S.-S. Yang, N.-S. Tzeng, M.-H. Chen, and C.-S. Liang. Risk of Substance Use Disorder and Its Associations With Comorbidities and Psychotropic Agents in Patients With Autism. *JAMA Pediatrics*, 175(2):e205371, Feb. 2021. ISSN 2168-6203. doi: 10.1001/jamapediatrics.2020.5371. URL https://pmc.ncbi.nlm.nih.gov/articles/PMC7783585/.

[19] M. M. Hughes, K. A. Shaw, M. E. Patrick, M. DiRienzo, A. V. Bakian, D. A. Bilder, M. S. Durkin, A. Hudson, M. H. Spivey, L. S. DaWalt, A. Salinas, Y. D. Schwenk, M. Lopez, T. M. Baroud, and M. J. Maenner. Adolescents With Autism Spectrum Disorder: Diagnostic Patterns, Co-occurring Conditions, and Transition Planning. *Journal of Adolescent Health*, 73(2):271–278, Aug. 2023. ISSN 1054-139X. doi: 10.1016/j.jadohealth.2022.12.010. URL https://www.sciencedirect.com/science/article/pii/S1054139X23000010.

[20] I. Ivanović. Psychiatric Comorbidities in Children With ASD: Autism Centre Experience. *Frontiers in Psychiatry*, 12, June 2021. ISSN 1664-0640. doi: 10.3389/fpsyt.2021.673169. URL https://www.frontiersin.org/journals/psychiatry/articles/10.3389/fpsyt.2021.673169/full.

[21] N. Jadav and V. H. Bal. Associations between co-occurring conditions and age of autism diagnosis: Implications for mental health training and adult autism research. *Autism Research*, 15 (11):2112–2125, 2022. ISSN 1939-3806. doi: 10.1002/aur.2808. URL https://onlinelibrary.wiley.com/doi/abs/10.1002/aur.2808. _eprint: https://onlinelibrary.wiley.com/doi/pdf/10.1002/aur.2808.

[22] E. Kalyva, M. Kyriazi, E. Vargiami, and D. I. Zafeiriou. A review of co-occurrence of autism spectrum disorder and Tourette syndrome. *Research in Autism Spectrum Disorders*, 24:39–51, Apr. 2016. ISSN 1750-9467. doi: 10.1016/j.rasd.2016.01.007. URL https://www.sciencedirect.com/science/article/pii/S1750946716300071.

[23] V. Khachadourian, B. Mahjani, S. Sandin, A. Kolevzon, J. D. Buxbaum, A. Reichenberg, and M. Janecka. Comorbidities in autism spectrum disorder and their etiologies. *Translational Psychiatry*, 13(1):71, Feb. 2023. ISSN 2158-3188. doi: 10.1038/s41398-023-02374-w. URL https://www.nature.com/articles/s41398-023-02374-w.

[24] A. Koceski and V. Trajkovski. Health status of people with autism spectrum disorder. *Advances in Autism*, 8(3):252–263, June 2022. ISSN 2056-3868, 2056-3868. doi: 10.1108/AIA-01-2021-0005. URL http://www.emerald.com/aia/article/8/3/252-263/59892.

[25] M. Kurzius-Spencer, S. Pettygrove, D. Christensen, A. L. Pedersen, C. Cunniff, F. J. Meaney, G. N. Soke, R. A. Harrington, M. Durkin, and S. Rice. Behavioral problems in children with autism spectrum disorder with and without co-occurring intellectual disability. *Research in Autism Spectrum Disorders*, 56:61–71, Dec. 2018. ISSN 1750-9467. doi: 10.1016/j.rasd.2018.09.002. URL https://www.sciencedirect.com/science/article/pii/S1750946718301363.

[26] M.-C. Lai, C. Kassee, R. Besney, S. Bonato, W. Mandy, P. Szatmari, and S. Ameis. Prevalence of co-occurring mental health diagnoses in the autism population: a systematic review and meta-analysis. *The Lancet Psychiatry*, 6(10):819–829, Oct. 2019. ISSN 2215-0366. doi: 10.1016/S2215-0366(19)30289-5. URL https://www.sciencedirect.com/science/article/abs/pii/S2215036619302895.

[27] A. G. Lever and H. M. Geurts. Psychiatric Co-occurring Symptoms and Disorders in Young, Middle-Aged, and Older Adults with Autism Spectrum Disorder. *Journal of Autism and Developmental Disorders*, 46(6):1916–1930, June 2016. ISSN 1573-3432. doi: 10.1007/s10803-016-2722-8. URL https://doi.org/10.1007/s10803-016-2722-8.

[28] A. Longo, D. Radford, and B. N. Hand. A US national update of health condition prevalence among privately-insured autistic adults. *Journal of Comparative Effectiveness Research*, 13 (3):e230051, Mar. 2024. doi: 10.57264/cer-2023-0051. URL https://becarispublishing.com/doi/full/10.57264/cer-2023-0051.

[29] C. Lopez-Arvizu, D. L. Steelesmith, B. N. Hand, R. Huang, A. J. Thompson, E. N. Llamocca, B. A. Quinn, C. A. Fontanella, and J. V. Campo. Correlates of Deliberate Self-Harm in Youth With Autism and/or Intellectual Disability. *JAACAP Open*, 3(3):477–484,

Sept. 2025. ISSN 2949-7329. doi: 10.1016/j.jaacop.2024.09.012. URL https://www.sciencedirect.com/science/article/pii/S2949732925000043.

[30] L. Margari, R. Palumbi, A. Peschechera, F. Craig, C. de Giambattista, P. Ventura, and F. Margari. Sex-Gender Comparisons in Comorbidities of Children and Adolescents With High-Functioning Autism Spectrum Disorder. *Frontiers in Psychiatry*, 10:159, Mar. 2019. ISSN 1664-0640. doi: 10.3389/fpsyt.2019.00159. URL https://www.ncbi.nlm.nih.gov/pmc/articles/PMC6445051/.

[31] S. D. Mayes, S. K. Pardej, and D. A. Waschbusch. Oppositional Defiant Disorder in Autism and ADHD. *Journal of Autism and Developmental Disorders*, July 2024. ISSN 1573-3432. doi: 10.1007/s10803-024-06437-9. URL https://doi.org/10.1007/s10803-024-06437-9.

[32] M. Micai, L. M. Fatta, L. Gila, A. Caruso, T. Salvitti, F. Fulceri, A. Ciaramella, R. D'Amico, C. Del Giovane, M. Bertelli, G. Romano, H. J. Schünemann, and M. L. Scattoni. Prevalence of co-occurring conditions in children and adults with autism spectrum disorder: A systematic review and meta-analysis. *Neuroscience & Biobehavioral Reviews*, 155:105436, Dec. 2023. ISSN 0149-7634. doi: 10.1016/j.neubiorev.2023.105436. URL https://www.sciencedirect.com/science/article/pii/S0149763423004050.

[33] H. L. Miller, G. M. Sherrod, J. E. Mauk, N. E. Fears, L. S. Hynan, and P. M. Tamplain. Shared Features or Co-occurrence? Evaluating Symptoms of Developmental Coordination Disorder in Children and Adolescents with Autism Spectrum Disorder. *Journal of Autism and Developmental Disorders*, 51(10):3443–3455, Oct. 2021. ISSN 1573-3432. doi: 10.1007/s10803-020-04766-z. URL https://doi.org/10.1007/s10803-020-04766-z.

[34] M. G. Mosner, J. L. Kinard, J. S. Shah, S. McWeeny, R. K. Greene, S. C. Lowery, C. A. Mazefsky, and G. S. Dichter. Rates of Co-occurring Psychiatric Disorders in Autism Spectrum Disorder using the Mini International Neuropsychiatric Interview. *Journal of autism and developmental disorders*, 49(9):3819–3832, Sept. 2019. ISSN 0162-3257. doi: 10.1007/s10803-019-04090-1. URL https://www.ncbi.nlm.nih.gov/pmc/articles/PMC6669096/.

[35] T. Mutluer, H. Aslan Genç, A. Özcan Morey, H. Yapici Eser, B. Ertinmaz, M. Can, and K. Munir. Population-Based Psychiatric Comorbidity in Children and Adolescents With Autism Spectrum Disorder: A Meta-Analysis. *Frontiers in Psychiatry*, 13, May 2022. ISSN 1664-0640. doi: 10.3389/fpsyt.2022.856208. URL https://www.frontiersin.org/journals/psychiatry/articles/10.3389/fpsyt.2022.856208/full.

[36] E. O'Nions, D. Lewer, I. Petersen, J. Brown, J. E. J. Buckman, R. Charlton, C. Cooper, C. E. Baou, F. Happé, J. Manthorpe, D. G. J. McKechnie, M. Richards, R. Saunders, C. Zanker, W. Mandy, and J. Stott. Estimating life expectancy and years of life lost for autistic people in the

UK: a matched cohort study. *The Lancet Regional Health – Europe*, 36, Jan. 2024. ISSN 2666-7762. doi: 10.1016/j.lanepe.2023.100776. URL https://www.thelancet.com/journals/lanepe/article/PIIS2666-7762(23)00195-3/fulltext.

[37] P.-Y. Pan, S. Bölte, P. Kaur, S. Jamil, and U. Jonsson. Neurological disorders in autism: A systematic review and meta-analysis. *Autism*, 25(3): 812–830, Apr. 2021. ISSN 1362-3613. doi: 10.1177/1362361320951370. URL https://doi.org/10.1177/1362361320951370.

[38] S. J. Rim, K. Kwak, and S. Park. Risk of psychiatric comorbidity with autism spectrum disorder and its association with diagnosis timing using a nationally representative cohort. *Research in Autism Spectrum Disorders*, 104:102134, June 2023. ISSN 17509467. doi: 10.1016/j.rasd.2023.102134. URL https://linkinghub.elsevier.com/retrieve/pii/S175094672300034X.

[39] E. Rydzewska, L. A. Hughes-McCormack, C. Gillberg, A. Henderson, C. MacIntyre, J. Rintoul, and S.-A. Cooper. Prevalence of long-term health conditions in adults with autism: observational study of a whole country population. *BMJ Open*, 8(8):e023945, Sept. 2018. ISSN 2044-6055. doi: 10.1136/bmjopen-2018-023945. URL https://www.ncbi.nlm.nih.gov/pmc/articles/PMC6120653/.

[40] E. Rødgaard, K. Jensen, K. W. Miskowiak, and L. Mottron. Autism comorbidities show elevated female-to-male odds ratios and are associated with the age of first autism diagnosis. *Acta Psychiatrica Scandinavica*, 144 (5):475–486, Nov. 2021. ISSN 0001690X. doi: 10.1111/acps.13345.

[41] M. Salehi, S. Jaka, A. Lotfi, A. Ahmad, M. Saeidi, and S. Gunturu. Prevalence, Socio-Demographic Characteristics, and Co-Morbidities of Autism Spectrum Disorder in US Children: Insights from the 2020–2021 National Survey of Children's Health. *Children*, 12(3):297, Feb. 2025. ISSN 2227-9067. doi: 10.3390/children12030297. URL https://www.ncbi.nlm.nih.gov/pmc/articles/PMC11941283/.

[42] D. E. Schendel, M. Overgaard, J. Christensen, L. Hjort, M. Jørgensen, M. Vestergaard, and E. T. Parner. Association of Psychiatric and Neurologic Comorbidity With Mortality Among Persons With Autism Spectrum Disorder in a Danish Population. *JAMA Pediatrics*, 170(3):243–250, Mar. 2016. ISSN 2168-6203. doi: 10.1001/jamapediatrics.2015.3935. URL https://doi.org/10.1001/jamapediatrics.2015.3935.

[43] W. Schott, S. Tao, and L. Shea. Co-occurring conditions and racial-ethnic disparities: Medicaid enrolled adults on the autism spectrum. *Autism Research*, 15(1):70–85, 2022. ISSN 1939-3806. doi: 10.1002/aur.2644. URL https://onlinelibrary.wiley.com/doi/abs/10.1002/aur.2644. _eprint: https://onlinelibrary.wiley.com/doi/pdf/10.1002/aur.2644.

[44] V. Semovski, C. King, N. Lapshina, and S. L. Stewart. A cross-sectional examination of service complexity in youths with co-occurring autism spectrum disorder and psychiatric or medical diagnoses across service sectors. *Frontiers in Psychology*, 13, Feb. 2023. ISSN 1664-1078. doi: 10.3389/fpsyg.2022.1027373. URL `https://www.frontiersin.org/journals/psychology/articles/10.3389/fpsyg.2022.1027373/full`

[45] A. Shoaib, M. S. Cepeda, G. Murray, and R. Ochs-Ross. Autism: Comorbidities and Treatment Patterns in the Real World, a Retrospective Cohort Study Among Children, Adolescents and Adults Newly Diagnosed with Autism. *Journal of Autism and Developmental Disorders*, 52(10):4311–4320, Oct. 2022. ISSN 1573-3432. doi: 10.1007/s10803-021-05289-x. URL `https://link-springer-com.mutex.gmu.edu/article/10.1007/s10803-021-05289-x`. Company: Springer Distributor: Springer Institution: Springer Label: Springer Number: 10.

[46] G. N. Soke, M. J. Maenner, D. Christensen, M. Kurzius-Spencer, and L. A. Schieve. Prevalence of Co-occurring Medical and Behavioral Conditions/Symptoms Among 4- and 8-Year-Old Children with Autism Spectrum Disorder in Selected Areas of the United States in 2010. *Journal of Autism and Developmental Disorders*, 48(8):2663–2676, Aug. 2018. ISSN 1573-3432. doi: 10.1007/s10803-018-3521-1. URL `https://link.springer.com/article/10.1007/s10803-018-3521-1`. Company: Springer Distributor: Springer Institution: Springer Label: Springer Number: 8.

[47] J. F. G. Underwood, M. DelPozo-Banos, A. Frizzati, D. Rai, A. John, and J. Hall. Neurological and psychiatric disorders among autistic adults: a population healthcare record study. *Psychological Medicine*, 53(12):5663–5673, Oct. 2022. ISSN 0033-2917. doi: 10.1017/S0033291722002884. URL `https://www.ncbi.nlm.nih.gov/pmc/articles/PMC10482712/`.

[48] R. Vohra, S. Madhavan, and U. Sambamoorthi. Comorbidity prevalence, healthcare utilization, and expenditures of Medicaid enrolled adults with autism spectrum disorders. *Autism : the international journal of research and practice*, 21(8):995–1009, Nov. 2017. ISSN 1362-3613. doi: 10.1177/1362361316665222. URL `https://www.ncbi.nlm.nih.gov/pmc/articles/PMC5517354/`.
